# Burden of stroke in North Africa and Middle East, 1990 to 2019: a systematic analysis for the global burden of disease study 2019

**DOI:** 10.1186/s12883-022-02793-0

**Published:** 2022-07-27

**Authors:** Ataollah Shahbandi, Parnian Shobeiri, Sina Azadnajafabad, Sahar Saeedi Moghaddam, Yeganeh Sharifnejad Tehrani, Narges Ebrahimi, Nazila Rezaei, Mohammad-Mahdi Rashidi, Seyyed-Hadi Ghamari, Mohsen Abbasi-Kangevari, Sogol Koolaji, Rosa Haghshenas, Negar Rezaei, Bagher Larijani, Farshad Farzadfar

**Affiliations:** 1 Non-Communicable Diseases Research Center, Endocrinology and Metabolism Population Sciences Institute, Tehran University of Medical Sciences, No. 10, Jalal Al-e-Ahmad Highway, Tehran, 1411713119 Iran; 2 Endocrinology and Metabolism Research Center, Endocrinology and Metabolism Clinical Sciences Institute, Tehran University of Medical Sciences, Tehran, Iran

**Keywords:** Stroke, Global burden of disease, DALYs, Risk factors

## Abstract

**Background:**

While several studies investigated the epidemiology and burden of stroke in the North Africa and Middle East region, no study has comprehensively evaluated the age-standardized attributable burden to all stroke subtypes and their risk factors yet.

**Objective:**

The aim of the present study is to explore the regional distribution of the burden of stroke, including ischemic stroke, subarachnoid hemorrhage, and intracerebral hemorrhage, and the attributable burden to its risk factors in 2019 among the 21 countries of North Africa and Middle East super-region.

**Methods:**

The data of the Global Burden of Disease Study (GBD) 2019 on stroke incidence, prevalence, death, disability-adjusted life years (DALYs), years of life lost (YLLs), years lived with disability (YLDs) rates, and attributed deaths, DALYs, YLLs, and YLDs to stroke risk factors were used for the present study.

**Results:**

The age-standardized deaths, DALYs, and YLLs rates were diminished statistically significant by 27.8, 32.0, and 35.1% from 1990 to 2019, respectively. Attributed deaths, DALYs, and YLLs to stroke risk factors, including high systolic blood pressure, high body-mass index, and high fasting plasma glucose shrank statistically significant by 24.9, 25.8, and 28.8%, respectively.

**Conclusion:**

While the age-standardized stroke burden has reduced during these 30 years, it is still a concerning issue due to its increased burden in all-age numbers. Well-developed primary prevention, timely diagnosis and management of the stroke and its risk factors might be appreciated for further decreasing the burden of stroke and its risk factors and reaching Sustainable Development Goal 3.4 target for reducing premature mortality from non-communicable diseases.

**Supplementary Information:**

The online version contains supplementary material available at 10.1186/s12883-022-02793-0.

## Introduction

Stroke is defined as “A group of pathological conditions characterized by sudden, non-convulsive loss of neurological function due to brain ischemia or intracranial hemorrhages” by the National Library of Medicine [1]. It is classified either as hemorrhagic or ischemic, with the former being categorized as subarachnoid or intracerebral hemorrhage [2–4]. While the etiologies, risk factors, diagnosis, and management of strokes have been comprehensively studied in recent decades, it is still one of the main contributors to the burden of diseases worldwide [5, 6], putting ischemic and hemorrhagic stroke as two prominent diseases of Sustainable Development Goal target 3.4 (SDG 3.4) by the United Nations [7].

As a region that experiences rapid economic growth and consequent lifestyle modification [8, 9], North Africa and the Middle East super-region is a zone of particular interest to investigate the pattern of change in the stroke burden and attributable burden to its risk factors. The impact of lifestyle and behavioral parameters on stroke is well established, including tobacco smoking, diet, and physical activity [10, 11]. On the other hand, stroke was always a major health problem in this region during the last decades due to the high prevalence of risk factors such as hypertension and indoor air pollution [12–14]. While several studies investigated the epidemiology and burden of stroke in the countries located in this region [12, 13, 15–18], no study was found to comprehensively evaluate the age-standardized attributable burden to all stroke subtypes and their risk factors, making it difficult to monitor the effectiveness of health interventions to achieve the SDG 3.4 target: “by 2030 reduce by one third [relative to 2015 levels] premature mortality from non-communicable diseases (NCDs) through prevention and treatment and promote mental health and well-being” [7].

The aim of the present study is to explore the regional distribution of the burden of stroke and attributable burden to its risk factors based on age, sex, and stroke subtypes and compare these parameters in 2019 with 1990 among the 21 countries of North Africa and Middle East super-region. The results of the present study can be utilized to monitor the latest state and trends of ischemic and hemorrhagic stroke as the components of SDG 3.4 [7].

## Methods

The data of the Global Burden of Disease (GBD) Study 2019 on stroke incidence, prevalence, death, disability-adjusted life years (DALYs), years of life lost (YLLs), years lived with disability (YLDs), and attributed deaths, DALYs, YLLs, and YLDs to stroke risk factors were used for the present study [6, 19]. North Africa and the Middle East are one of the 7 GBD super-regions and 21 GBD regions. The stroke data was collected for the 21 countries of this super-region by sex and age groups. This region includes Afghanistan, Algeria, Bahrain, Egypt, Iran (Islamic Republic of), Iraq, Jordan, Kuwait, Lebanon, Libya, Morocco, Oman, Palestine, Qatar, Saudi Arabia, Sudan, Syrian Arab Republic (SAR), Tunisia, Turkey, United Arab Emirates (UAE), and Yemen. It is noteworthy that Sudan was partitioned into Sudan and South Sudan in 2011.

Stroke and its subtypes (ischemic, intracerebral hemorrhage (ICH), and subarachnoid hemorrhage (AH)) were defined using codes of the 10th revision of the International Classification of Diseases (ICD-10) of the World Health Organization (WHO) (Supplementary File 1). Yielded data from various surveys, reports, scientific literature, modeled data, and administrative data were utilized to define these conditions [20].

The age categories included under 5, 5 to 9, 10 to 14, …, 75 to 79, and 80+ years of age in addition to all-ages and age-standardized. Furthermore, the data was available for female, male, and both sex populations. All of the extracted data is publicly available via the allocated website (http://ghdx.healthdata.org/gbd-results-tool). The GBD stroke data was complete for all of the regional countries for the 30-year span of the study (1990–2019).

GBD introduced a four-level hierarchical system to categorize risk factors and assess specific and combinatorial risk factors [19]. The level four included alcohol use, ambient particulate matter pollution, diet high in red meat, diet high in sodium, diet low in fiber, diet low in fruits, diet low in vegetables, diet low in whole grains, high body−mass index (BMI), high fasting plasma glucose (FPG), high LDL cholesterol, high systolic blood pressure (SBP), high temperature, household air pollution from solid fuels, kidney dysfunction, lead exposure, low physical activity, low temperature, secondhand smoke, and smoking. The attributed burden to each risk factor was estimated by the Comparative Risk Assessment (CRA) framework in six steps [19]. Socio-demographic Index (SDI) was computed using income per capita, educational attainment, and fertility rates among females of 25 years of age or younger in order to stratify the socioeconomic status of different countries [6]. Uncertainty intervals (UIs) were calculated for each quantitative data on the basis of 25th and 975th ordered values of 1000 draws of the posterior distribution using the Bayesian approach [6]. R version 3.5.0, Stata version 13, and Python version 3.6.2 were used for the statistical analyses and visualizations in this study. The detailed method for calculating each epidemiologic parameter is described in other papers [6, 19].

## Results

During the 30-year time-span, all-ages stroke incidence, prevalence, death, DALY, YLL, and YLD rates have grown statistically significant by 130.7% (95% UI 124.4 to 137.7), 142.1% (137.8 to 146.3), 75.5% (56.2 to 98.8), 43.3% (27.2 to 61.4), 34.5% (18.0 to 53.6), and 140.2% (135.3 to 145.3), respectively (Table 1).

**Table 1:**
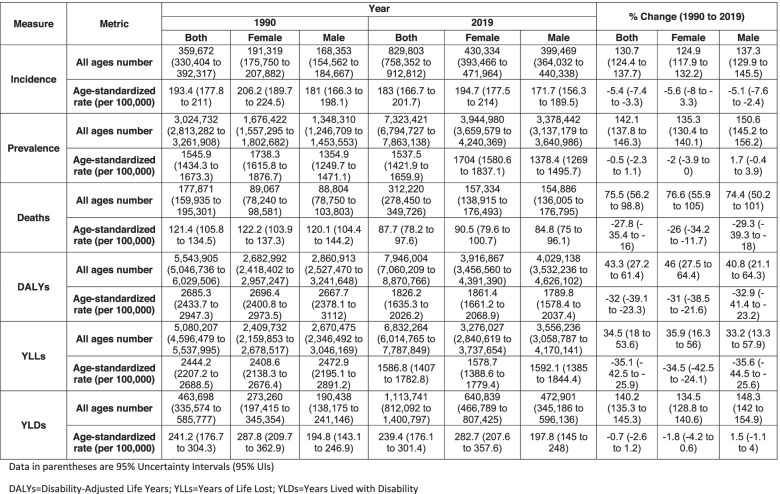
All ages number and age-standardized burden of stroke by sex in 1990 and 2019 at the super-region with percent changes

The age-standardized stroke incidence, death, DALY, YLL, and YLD rates declined by 5.4% (− 7.4 to − 3.3), 27.8% (− 35.4 to − 16.0), 32.0 (− 39.1 to − 23.3), 35.1 (− 42.5 to − 25.9), and 0.7 (− 2.6 to 1.2), respectively, from 1990 to 2019. Age-standardized stroke incidence and DALY rates in female population have decreased by 5.6% (− 8.0 to − 3.3) and 31.0% (− 38.5 to − 21.6), respectively. In the male population, age-standardized stroke incidence and DALY rates diminished by 5.1% (− 7.6 to − 2.4) and 32.9% (− 41.4 to − 23.2), respectively (Table 1).

Attributed age-standardized DALY rates to stroke risk factors have declined by 25.4% (− 33.9 to − 12.3) and 26.1% (− 36.8 to − 13.5) during this 30-year period in the female and male population, respectively (Supplementary Table 1). The stroke incidence and DALY rates increased steadily with older age groups both in 1990 and 2019, with the exception of unexpectedly high DALY rates of stroke in the under-five age group in both time points (Supplementary Fig. 1).

**Fig. 1:**
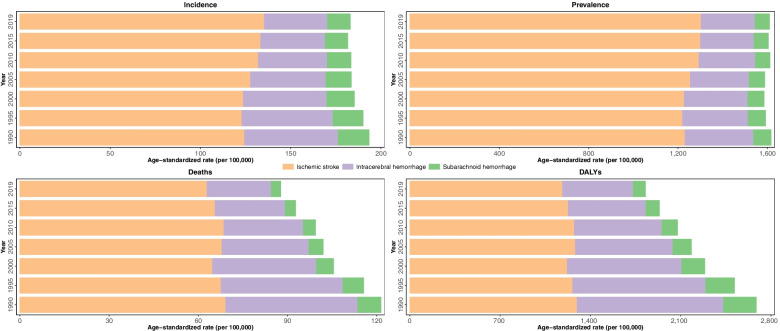
Burden of stroke by each subtype, 1990 to 2019

Stroke was classified into the ischemic stroke, ICH, and SAH. Age-standardized ICH and SAH incidence rates consistently subsided in the super-region by 32.5% (− 34.4 to − 30.5) and 26.3% (− 29.2 to − 23.2), respectively (Fig. 1). On the other hand, ischemic stroke incidence rates escalated by 8.8 (6.3 to 11.4) (Fig. 1). Age-standardized death rates of ICH, SAH, and ischemic stroke dropped by 51.3% (− 58.1 to − 41.2), 59.0% (− 70.6 to − 31.5), and 9.1% (− 20.6 to 4.0) during the 30-year time-span.

**Fig. 2:**
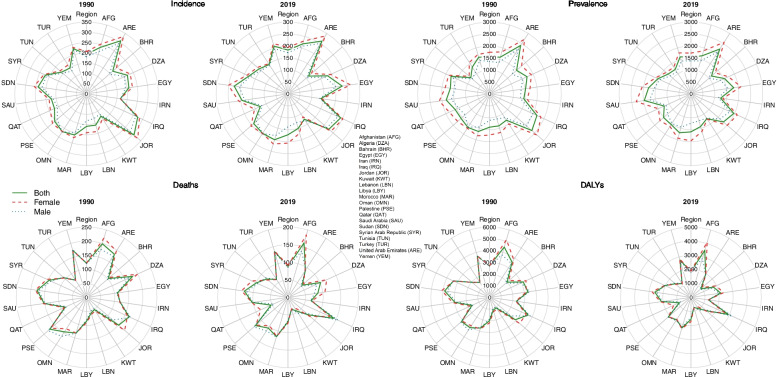
Burden of stroke in the super-region and its 21 countries by sex, 1990 compared to 2019

Age-standardized incidence, prevalence, death, and DALY rates per 100,000 population exhibited a relatively homogenous geographical pattern in both 1990 and 2019 (Fig. 2). Among the 21 countries of the North Africa and Middle East super-region, Bahrain had the most decrease in stroke incidence rate by 32.8% (− 36.8 to − 28.7) (Supplementary Table 2). The stroke incidence rate was reduced in 19 countries and increased only in Egypt and Libya by 9.6% (3.5 to 16.5) and 7.9% (2.1 to 14.6), respectively. During this 30-year period, the death, DALY, and YLL rates have dropped in all countries of this super-region. UAE was the most successful country in lessening the stroke death rate by 50% (− 62 to − 34.9), while Kuwait had the slightest decrease by 9.6% (− 24.2 to 7.7). At the same time, Bahrain dwindled the DALY and YLL rates more than other countries in the region, by 52.5% (− 61.2 to − 42.5) and 56.1% (− 65.0 to − 45.0), respectively.

**Fig. 3:**
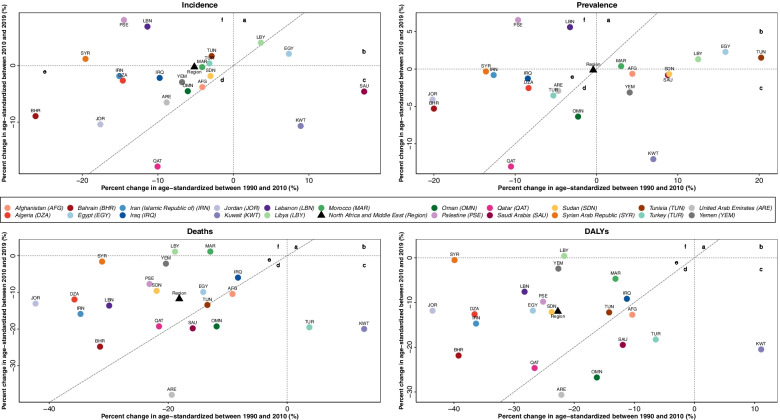
Percent change in the stroke burden from 1990 to 2019 in the super-region and its 21 countries; a, rates increased more rapidly after vs before 2010; b, rates increased more slowly after vs before 2010; c, rates increased before 2010 but decreased after 2010; d, rates decreased more rapidly after than before 2010; e, rates decreased more slowly after than before 2010; f, rates decreased before 2010 but increased after 2010

This 30-year period can be divided into two time spans: 1990–2010 and 2010–2019. Taken into account, Libya is the only country in the super-region that its age-standardized stroke incidence rate increased more rapidly after vs before 2010 (Fig. 3). While the stroke incidence rate also elevated in Egypt, its increase occurred more slowly after vs before 2010. Kuwait and Saudi Arabia were the two countries with increased stroke incidence rates before and decreased rates after 2010. Qatar was the only country in the super-region with a more rapid decrease in stroke incidence rate after vs before 2010. Looking at the DALYs age-standardized percentage changes at the two time spans, Kuwait faced an upward trend before and a downward trend after 2010. Surprisingly, Libya was the only country with a DALYs rate that decreased before 2010 but increased after 2010. Other countries of the region demonstrated a sustained decrease in DALYs rate during both time windows.

**Fig. 4:**
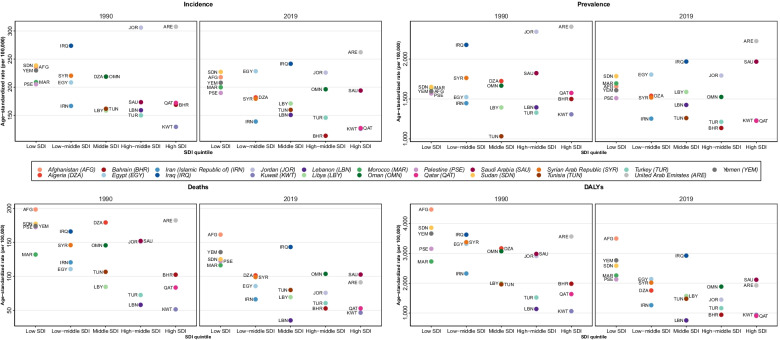
Stroke burden in the countries of the super-region stratified by SDI indexes, 1990 compared to 2019

The North Africa and Middle East super-region countries can be divided into five subgroups of SDI: low, low-middle, middle, high-middle, and high SDI (Fig. 4). UAE, Jordan, Oman, Iraq, Egypt, and Sudan had the highest age-standardized stroke rates in 2019 in their respective SDI subgroups. Furthermore, Saudi Arabia, Oman, Iraq, Egypt, and Afghanistan sustained the highest DALYs rate in their respective subgroup. A remarkable feature seen in the DALYs diagram was the overall comparable status of DALYs rate of low-middle, middle, high-middle, and high SDI countries, which is much less than low SDI countries.

During the 1990–2019 time-span, attributed death, DALY, and YLL rates to stroke risk factors shrank by 24.9% (− 33.5 to − 11.6), 25.8% (− 34.1 to − 14.0), and 28.8% (− 37.9 to − 15.8), respectively (Supplementary Table 1). The aforementioned parameters demonstrated a descending trend in all 21 countries, with UAE having the most decline in attributed deaths rate of 50.2% (− 62.3 to − 35.4), and Bahrain with the highest drop rate in DALYs and YLLs by 52.2% (− 61 to − 41.9) and 55.7% (− 65 to − 44.4), respectively (Supplementary Table 2).

High SBP stayed in the first rank of attributed DALYs rate in both 1990 and 2019 by far in the super-region among all of the level four risk factors of the stroke (Supplementary Table 3). However, its attributed age-standardized DALYs rate has substantially decreased from over 1250 life years to below 1000 life years per 100,000 population (− 24.9%). High BMI came in second in both years, and its attributed DALYs have minimally changed (− 6.2%). High FPG has surpassed ambient particulate matter pollution, becoming the third risk factor with the highest attributed DALYs rate. On the other hand, attributed DALYs rate to household air pollution from solid fuels has contracted to less than one-fourth of its original value from 1990 to 2019 (80.9%).

It was estimated that 875,610 new stroke cases would be estimated during 2019, considering population growth and aging (Table 2). While the number of new cases rose by 130.7% in 2019 compared to 1990, the number of new cases was 5.2% less than the expected new cases, staying at 829,803. Population growth, age structure change, and incidence rate change contributed to this growth by 76.4, 67.0%, and − 12.7%, respectively. Overall, UAE had the highest rise in the number of new cases by 710.5% (Supplementary Table 4). On the other hand, the SAR sustained the lowest elevation in the number of new cases by 61.4%.

**Table 2:**
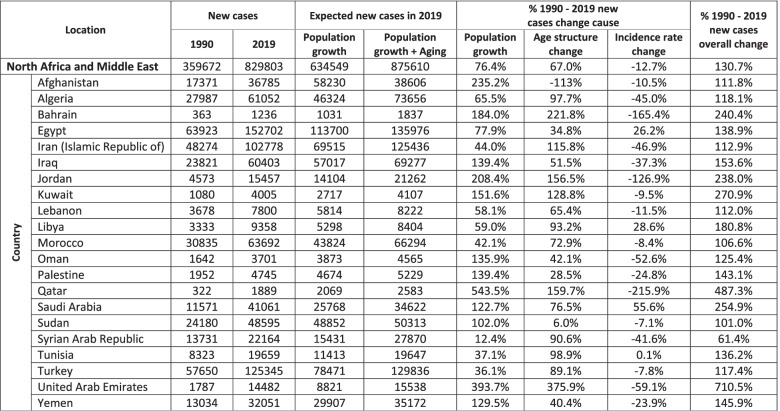
Decomposition of the predictors of expected new cases in 2019 at the super-region and its 21 countries

## Discussion

In the present study, the available relevant data were extracted from the GBD 2019 public database and were assessed for any remarkable trends and features regarding the burden of the stroke and the burden attributable to stroke risk factors in North Africa and Middle East super-region.

While the same public dataset was used by Jaberinezhad et al., there are several important differences between the two research endeavors [21]. The current study has performed the decomposition analysis on the GBD outputs of stroke incidence, and also reported the six health estimates (i.e., prevalence, incidence, death, YLL, YLD, and DALY rates) for each age group, sex, and country. On the other hand, the risk factors were explored in this study in the most meticulous manner possible by investigating the 20 risk factors of the level-four GBD hierarchy. While Jaberinezhad et al. reported the estimates for various age groups, the grouping and its aim differ from the present work. Furthermore, providing YLD and YLL rates, a comparison of 1990 health estimates to 2019, and assessing 1990–2019 trends of the stroke’s burden and its three subtypes were the unique features of the current paper.

Another point that might cause confusion for the readers of the two papers is the seemingly different reports using the same dataset regarding the overlapping health metrics reported in both articles. Regarding the attributable burden to stroke risk factors, the present paper reported age-standardized health metrics, unlike Jabarinezhad et al.’s. As a consequence, while the two risk factors with the most attributable burden were the same in both studies (i.e., high SBP and high BMI), the third risk factor differed as it was identified as ambient particulate matter pollution in this study and high FPG in the former study. Besides the differences in the yielded metrics caused by reporting either all-ages or age-standardized estimates, units of measurement differed on several occasions. As an instance, attributed DALY rates to risk factors were reported in both articles. However, it was reported in 100,000 of the population and by percentage in our and Jaberinezhad et al. studies. The discussed points were the main reasons that caused the differences in reporting the same health metrics using the mentioned dataset [21].

The upward trend of all-ages stroke burden was found in this paper, and it is consistent with the previous epidemiological studies of stroke burden in this super-region [12–14]. Albeit population growth and the change of population structure were the major contributors to the ongoing trend, inadequate lifestyle modification programs and education, leading to a high prevalence of risk factors (e. g., diabetes mellitus, tobacco smoking), and lackluster preventive strategies such as underdiagnosis of atrial fibrillation might particularly play a role in exacerbating the current trends in the super-region [14, 22].

Another evidence of the insufficient preventive strategies in the present study was the change in the pattern of stroke risk factors in the super-region. As a result of urbanization, attributed DALY rates to metabolic risk factors had an even greater share in 2019 than in 1990 in the region as well as globally [15, 23–26]. Public health efforts resulted in a reduced attributable burden to high SBP. However, much more enhancement in primary prevention strategies must be made to reduce the burden of high SBP even further, especially among young individuals and rural areas [27–30]. The same rule applies to high BMI, which its age-standardized attributed DALY rates remained almost the same during the period [24, 25]. As a result of lifestyle changes, the attributable burden to high FPG raised considerably compared to other parts of the world, implicating that the prevention strategies were inadequate during the 1990–2019 time span, and more work should be done for primary prevention and timely diagnosis and treatment of high FPG in population-wide scale [26, 31].

A global effort has been made to diminish the premature mortality caused by NCDs since the inception of SDG 3.4 [7, 32]. While each country of the super-region has a unique status regarding the prevalence of the attributable risk factors, all of these countries can appraise how the most successful countries such as Luxembourg, New Zealand, and South Korea are reaching the SDG 3.4 by decreasing the incidence of stroke using optimal prevention strategies [32]. By combining tested-and-approved prevention strategies with additional preventive measures required based on each country’s specific profile, a well-suited set of interventions would be created to decrease the stroke burden in the region further. In addition to components of each national preventive strategy, the pace of implementing changes cannot be the same as countries differ regarding their available healthcare resources and how much the premature mortality rates are required to mitigate in order to achieve SDG 3.4 goals [32].

While almost all countries in the region were moving toward lessened stroke incidence rates, Egypt and Libya faced an ascending trend in the age-standardized incidence rates of stroke. One possible explanation might be insufficient primary prevention. Another possible explanation might be the failure to allocate adequate resources to control the burden of stroke due to the damaged healthcare infrastructure secondary to domestic conflicts.

The overall attributed DALYs to high SBP decreased in the super-region in this 29-year period. However, this parameter experienced minimal changes in Egypt. On the other hand, the attributed DALYs rate to high FPG has almost doubled, at a much more rapid pace than the overall trend in the super-region. Libya also failed to make any substantial improvements to the attributed DALYs rate to stroke risk factors at the same time, suggesting that both countries still have more room for optimization of primary prevention strategies, along with a faster implementation pace. However, Libya’s status might be more concerning due to a faster increase in incidence and DALY rates after vs before 2010, requiring prompt actions. While the overall incidence rates increased in Egypt, it happened more slowly after vs before 2010, which might imply that good primary prevention strategies were adopted and started to show their impact. Recent studies also confirmed the increased prevalence of stroke risk factors such as hypertension, high BMI and metabolic syndrome in Egypt [15, 23] and Libya [33].

On the other end of the spectrum, Bahrain decreased the age-standardized incidence, DALY, and YLL rates and attributed DALYs and YLLs to stroke risk factors more than any other country. Attributed DALYs to high SBP, high FPG, high BMI, smoking, and ambient particulate matter pollution were all strikingly decreased in 2019 compared to 1990 in Bahrain, demonstrating the country’s extraordinary performance in the primary prevention of stroke and its consequent burden. Regarding stroke death and attributed death to risk factors rates, UAE outperformed other countries of the super-region, which can be possibly explained by population-wide screening programs for risk factor identification (e.g., “Weqaya”) and better access to care in this high SDI country [34]. In particular, screening programs might be a crucial step toward primary prevention of stroke as the country ranked first in the age-standardized stroke incidence rates in the super-region with a seven-fold increase.

The announcement of the global health agenda greatly impacted the policymaking and health service provision worldwide in 2011 [35]. Among the countries of the region, Kuwait and Saudi Arabia managed to turn the tides and diminish the stroke incidence rates only after 2010, probably due to enhanced prevention strategies and risk factor modification subsequent to the 2011 global health agenda [35]. However, Saudi Arabia was still at the top of regional high-SDI countries, showing much more room for improvement. The available evidence demonstrated the same trend for Iran, although the time point of the trend breakout was 2001–2002 in the case of this country [18].

While the preventive strategies were not satisfactory in the region, the therapeutic interventions were much more successful when looking into the trends. During the study time span, the age-standardized death, DALY, and YLL rates of stroke were considerably mitigated, while the incidence and YLD rates remained almost unchanged. This finding implicates the reduced regional burden of stroke and its risk factors and a transition from burden caused by mortality to burden asserted by morbidity. The latter transformation has also occurred globally [5, 6]. The underlying explanation for this shift might be the higher chance of survival in stroke patients for two reasons: less severe strokes because of primary prevention strategies and better short-term and long-term stroke management, treatment, and care in the recent years [5, 36, 37]. This transition is likely to progress further with the more widespread application of these novel therapies and the development of more efficacious and state-of-the-art therapeutic approaches (e.g., mechanical thrombectomy devices) [38, 39].

However, adhering merely to the therapeutic strategies is not the most efficient route to reaching the SDG 3.4 targets, particularly for low-SDI countries. A concerning finding of the present study is the divergence of stroke DALY rates trends between low-SDI and other countries of the super-region. This finding might not be surprising since stroke care and management costs continuously grew and tend to increase more in the future [5]. High-SDI countries of the super-region can easily sustain these increased costs due to their higher gross domestic product (GDP) per capita [40]. Moreover, lower levels of educational attainment in these countries might lead to less individualized risk factor modification and primary prevention of stroke [30]. This divergence might escalate even further if the stroke costs fail to be contained by effective measures such as thrombolytic therapy and early supportive discharge [5, 41].

A novel finding of the current paper is the classification of stroke subtypes’ prevalence, incidence, and burden in the super-region. Age-standardized non-traumatic SAH incidence rates continuously dropped during the 30-year span of the study. Lowered attributed DALYs rates can mediate this phenomenon to ambient particulate matter pollution, smoking, secondhand smoke, high SBP, and alcohol use in 2019 compared to 1990, all known as SAH and ICH risk factors [42–48]. Furthermore, better access to neurocritical care services secondary to economic development might have led to better management of these conditions, such as treating ruptured intracranial aneurysms and surgical hematoma evacuation, leading to lower death rates [43–45, 47]. The age-standardized death rates of ischemic stroke are also considerably less in 2019 than in 1990, probably due to better access to specialized treatment such as thrombolysis therapy, mechanical thrombectomy, and decompressive craniectomy [49, 50]. However, the incidence rates of ischemic stroke have grown since 1990, unlike hemorrhagic stroke. This finding suggests that specific risk factors for ischemic stroke were not sufficiently contained during this period. While attributed DALY rates to high SBP, ambient particulate matter pollution, smoking, secondhand smoke, and alcohol abuse reduced, DALY rates of high FPG are considerably elevated as one of the most important modifiable risk factors of ischemic stroke [51, 52].

The present study had several limitations. The relevant stroke data and its risk factors were available for every country of the region during 1990–2019. However, the quality of the data may differ significantly between the countries as epidemiological studies might be scarce in some of the low-SDI and politically unstable countries such as Sudan and Libya. This lack of primary epidemiologic data is even more pronounced for stroke subtypes, which would undeniably affect the certainty of the GBD estimates built upon these primary statistics. Further endeavors should be directed toward investigating the unfortunate consequences of conflicts for these countries regarding the stroke burden, as these upheavals took a huge toll on healthcare infrastructure in countries like Sudan and Libya. The primary epidemiological studies also might be considerably heterogeneous regarding stroke definition or measurement methods. While the GBD workgroup has developed a unique estimation method as UIs, more work is needed to enhance the representation of the intervals, especially for countries with more sparse stroke data. Moreover, stroke severity distribution is still lacking in the GBD reports, which might be measured via standardized, widely-accepted tools such as the National Institute of Health Stroke Scale (NIHSS) [53]. Finally, the burden of more similar conditions such as transient ischemic attacks (TIA) and risk factors (e.g., atrial fibrillation) can be added to the GBD database for future research.

## Conclusion

Stroke is consistently among the main drivers of the burden of the disease worldwide as one of the more emphasized counterparts of SDG 3.4. With the countries of North Africa and Middle East super-region confronting the urbanized lifestyle secondary to economic development, investigating the attributable burden to stroke subtypes and their risk factors is the first step in designing suitable primary prevention and timely management strategies in order to prevent premature mortality and consequently, fulfilling SDG 3.4 target. The present study utilized the 2019 GBD data to explore the stroke burden and the attributed burden to stroke risk factors. While the age-standardized stroke burden and attributable burden to stroke risk factors have decreased during 1990–2019, it is still unacceptably high, particularly in low-SDI countries. Well-developed primary prevention programs and timely diagnosis and management of the stroke and its risk factors in well-equipped healthcare centers may be needed to reduce the significant burden of stroke and attributed burden to stroke risk factors.

## Supplementary Information

**Additional file 1: Supplementary Fig. 1.** Burden of stroke in the super-region for different age groups by sex, 1990 compared to 2019

**Additional file 2: Supplementary Table 1.** All ages number and age-standardized attributable burden to stroke risk factors at the super-region

**Additional file 3: Supplementary Table 2.** Age-standardized stroke burden in 1990 and 2019 at each country of the super-region

**Additional file 4: Supplementary Table 3.** Age-standardized attributed burden to stroke risk factors in 1990 and 2019 at the super-region and its 21 countries

**Additional file 5: Supplementary Table 4.** Decomposition of the predictors of expected new cases in 2019 by sex at the super-region and its 21 countries

**Additional file 6: Supplementary File 1.** GBD 2019 cause list mapped to ICD-10 codes used in hospital/claim analyses and causes of death due to stroke and its subtypes

## Data Availability

The datasets analyzed during the current study are available in the Global Health Data Exchange repository, https://ghdx.healthdata.org/gbd-results-tool.
