# Supplementary figures and images for: Burden of stroke in North Africa and Middle East, 1990 to 2019: a systematic analysis for the global burden of disease study 2019
Source: BMC Neurol. 2022 Jul 27;22:279. doi: 10.1186/s12883-022-02793-0 (PMC9327376; doi:10.1186/s12883-022-02793-0)

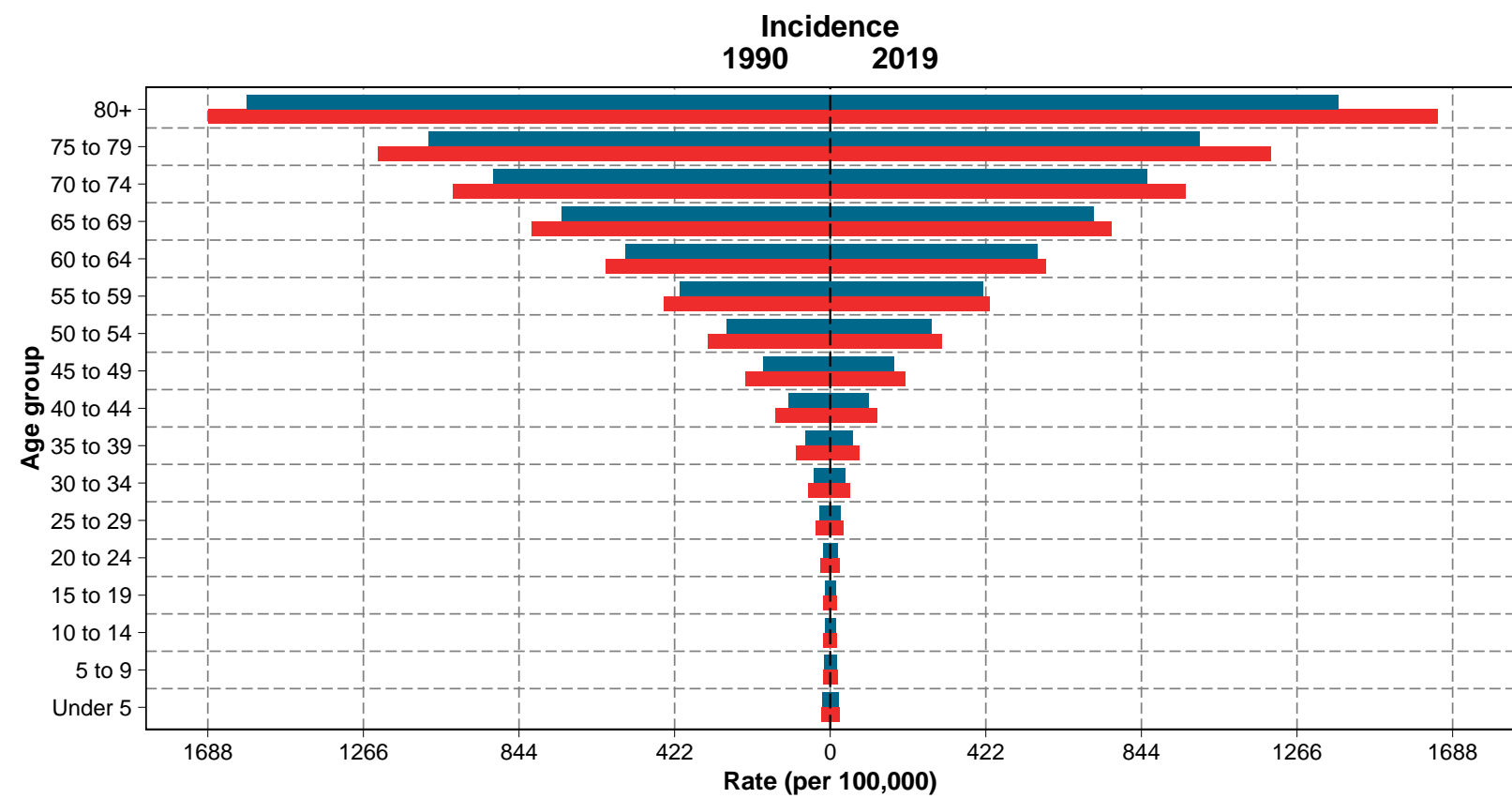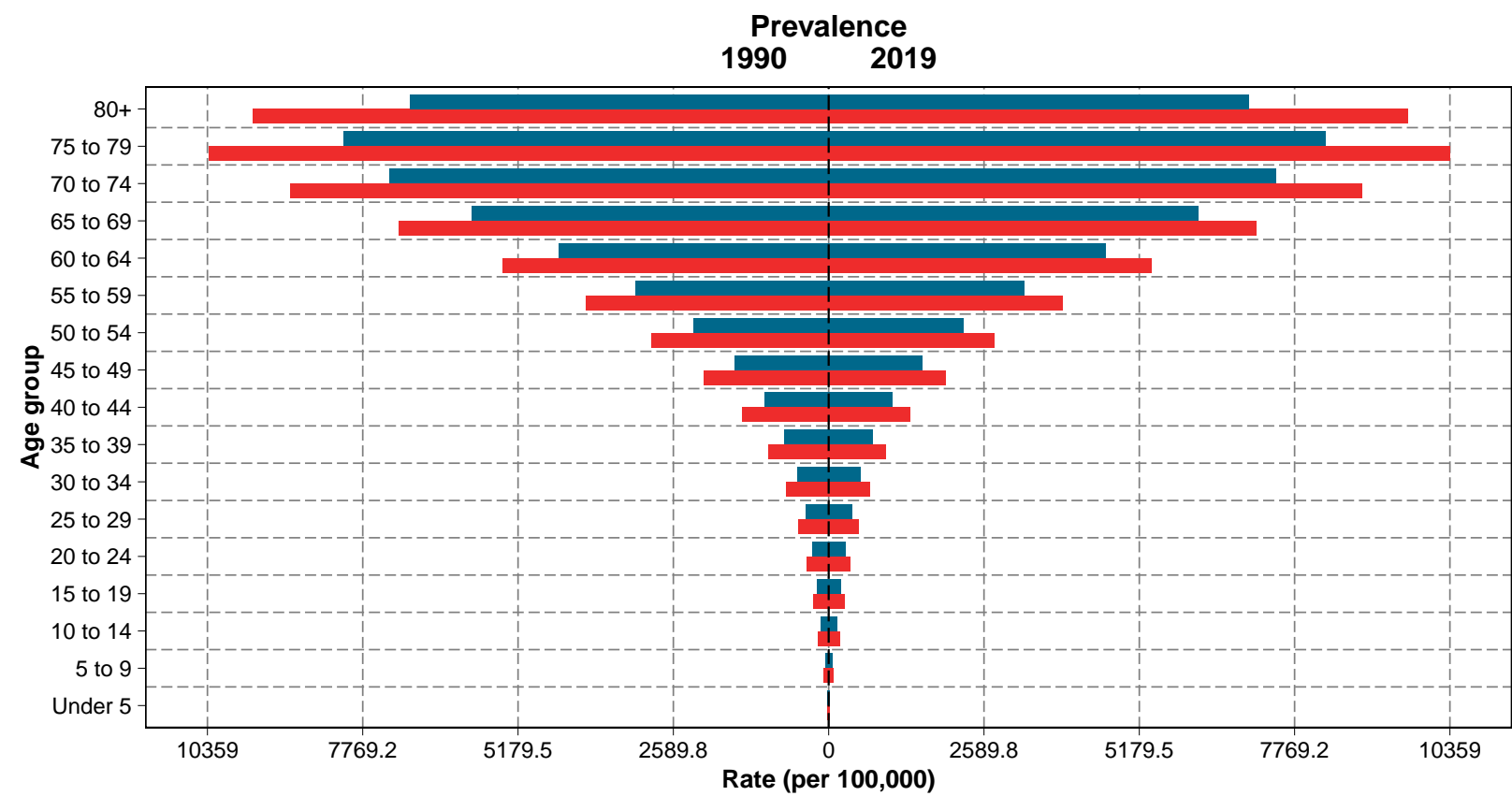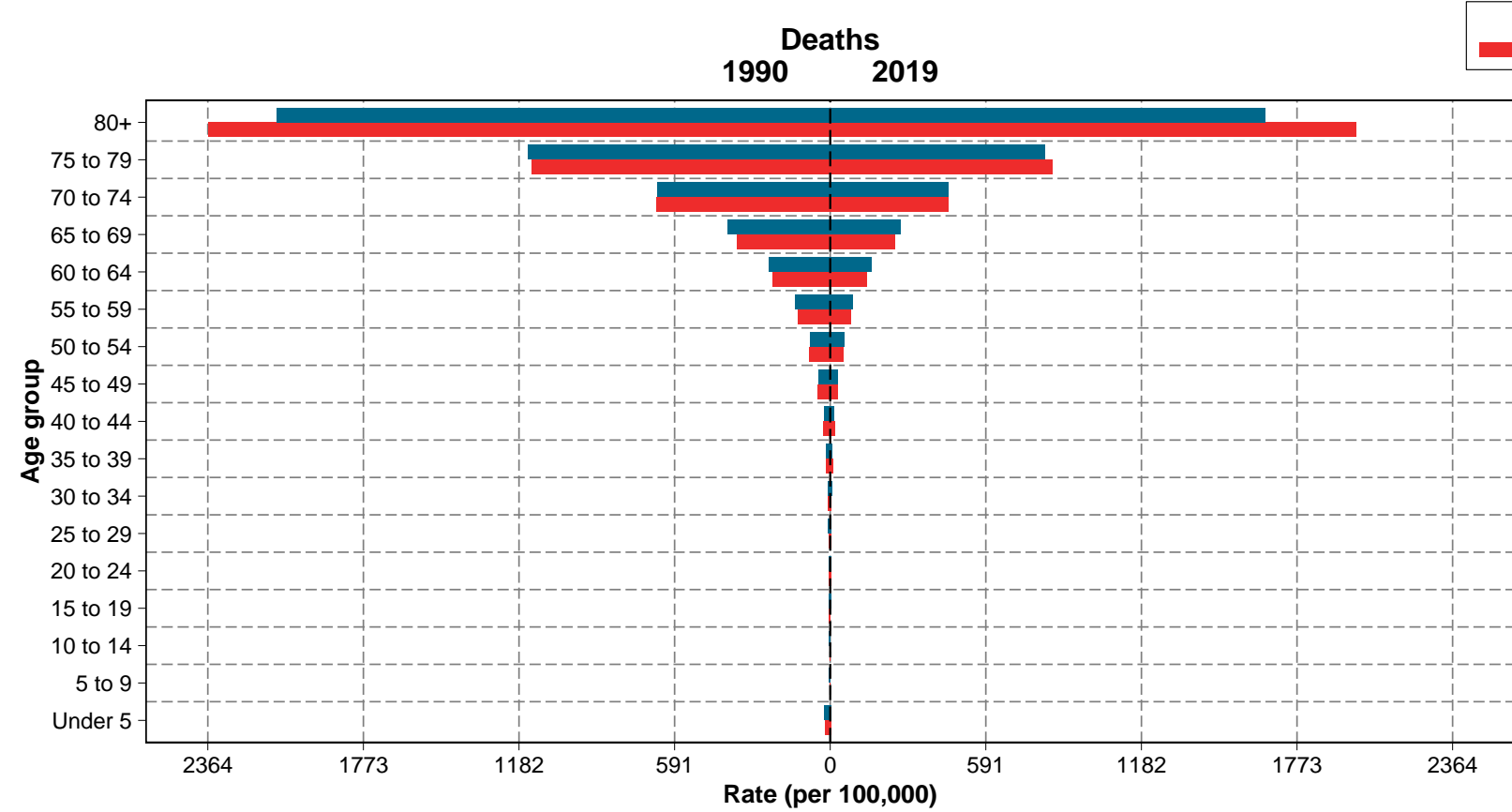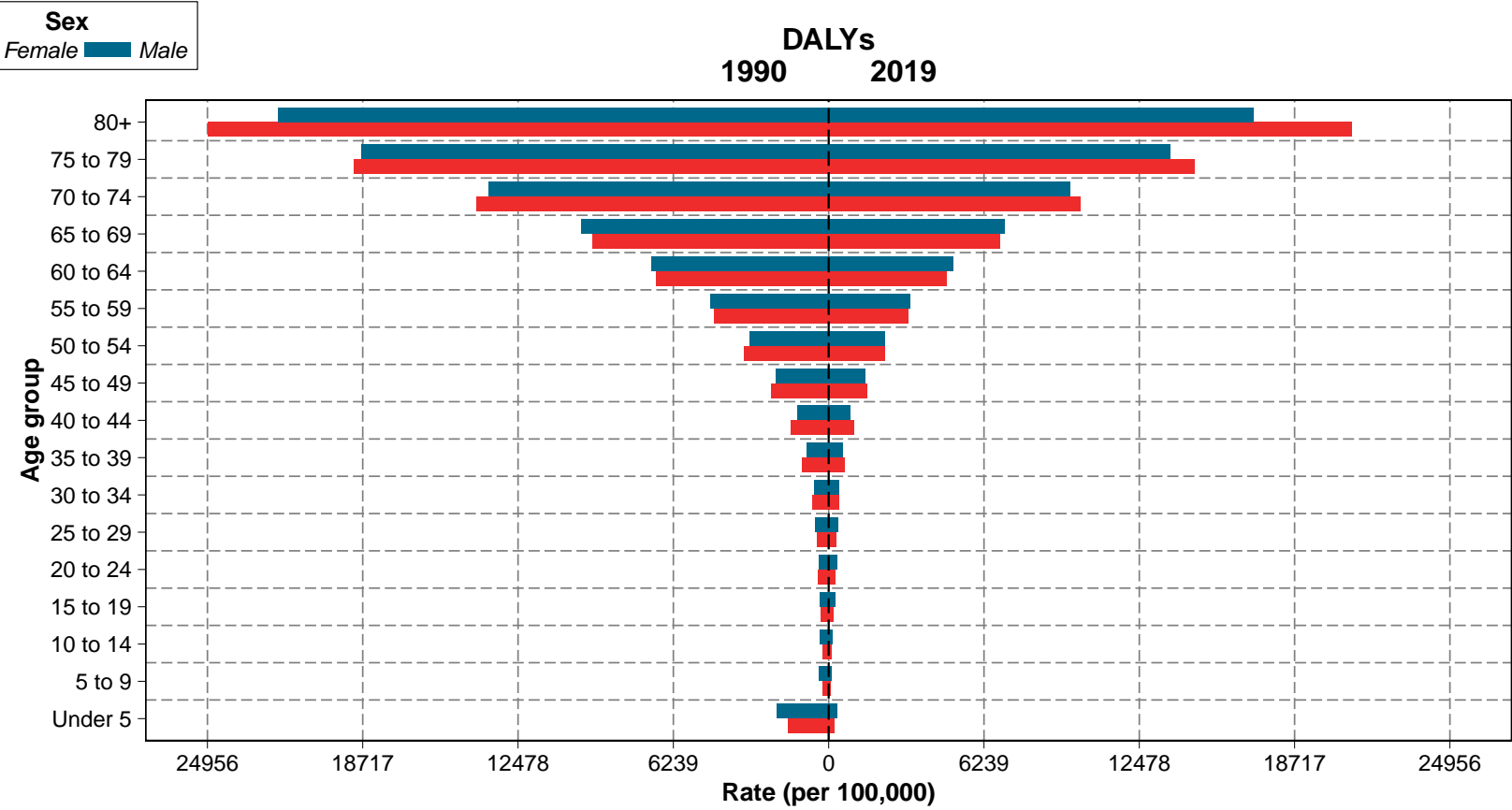

Sex  
Female Male

Supplement: Supplementary file 1 — Additional file 1: Supplementary Fig. 1. Burden of stroke in the super-region for different age groups by sex, 1990 compared to 2019 [file 12883_2022_2793_MOESM1_ESM.pdf]
