# Supplementary material for: Burden of stroke in North Africa and Middle East, 1990 to 2019: a systematic analysis for the global burden of disease study 2019
Source: BMC Neurol. 2022 Jul 27;22:279. doi: 10.1186/s12883-022-02793-0 (PMC9327376; doi:10.1186/s12883-022-02793-0)
Supplement: Supplementary file 2 — Additional file 2: Supplementary Table 1. All ages number and age-standardized attributable burden to stroke risk factors at the super-region [file 12883_2022_2793_MOESM2_ESM.pdf]

Measure	Metric	Year						% Change (1990 to 2019)		
		1990			2019					
		Both	Female	Male	Both	Female	Male	Both	Female	Male
Deaths	All ages number	139,646 (119,721 to 158,492)	70,687 (59,470 to 80,741)	68,960 (59,643 to 85,159)	268,925 (237,694 to 305,151)	135,278 (118,602 to 153,364)	133,647 (116,656 to 154,453)	92.6 (69 to 127.1)	91.4 (68.1 to 130.3)	93.8 (63.6 to 129)
	Age-standardized rate (per 100,000)	98.9 (83.4 to 112.4)	100 (82.8 to 115.1)	97.3 (83.7 to 119.8)	74.3 (65.5 to 84.4)	76.6 (66.5 to 87)	72 (63.2 to 83.1)	-24.9 (-33.5 to -11.6)	-23.4 (-32.5 to -7.3)	-26.1 (-37.2 to -13.2)
DALYs	All ages number	3,720,191 (3,296,673 to 4,210,080)	1,864,468 (1,630,099 to 2,100,616)	1,855,723 (1,625,798 to 2,239,319)	6,825,095 (5,997,712 to 7,672,914)	3,381,659 (2,964,458 to 3,806,477)	3,443,435 (3,000,704 to 3,949,761)	83.5 (62 to 112)	81.4 (59.1 to 112.7)	85.6 (59.2 to 118.2)
	Age-standardized rate (per 100,000)	2112.9 (1846.3 to 2382.6)	2148.6 (1852.9 to 2418.8)	2072.4 (1804.7 to 2486)	1566.9 (1393.2 to 1757.1)	1602.7 (1419.3 to 1798.2)	1530.7 (1345 to 1746.4)	-25.8 (-34.1 to -14)	-25.4 (-33.9 to -12.3)	-26.1 (-36.8 to -13.5)
YLLs	All ages number	3,343,813 (2,915,076 to 3,820,011)	1,642,895 (1,421,029 to 1,873,556)	1,700,919 (1,477,336 to 2,091,745)	5,856,993 (5,118,863 to 6,681,203)	2,826,616 (2,430,203 to 3,230,624)	3,030,377 (2,614,448 to 3,546,434)	75.2 (52.3 to 105.9)	72.1 (48.9 to 106.7)	78.2 (50.9 to 113.3)
	Age-standardized rate (per 100,000)	1909.3 (1647.4 to 2174.2)	1905.8 (1625.1 to 2166.3)	1907.7 (1648.8 to 2343.1)	1359.2 (1198.9 to 1548.9)	1358 (1182.6 to 1543.5)	1358.4 (1179.9 to 1577.9)	-28.8 (-37.9 to -15.8)	-28.7 (-37.8 to -14.4)	-28.8 (-39.7 to -15.5)
YLDs	All ages number	376,377 (275,042 to 477,102)	221,573 (162,779 to 280,478)	154,804 (112,886 to 197,367)	968,101 (708,487 to 1,217,543)	555,043 (406,276 to 703,151)	413,058 (300,546 to 520,677)	157.2 (151.4 to 163.6)	150.5 (143.9 to 157.8)	166.8 (159.1 to 174.8)
	Age-standardized rate (per 100,000)	203.6 (148.4 to 259)	242.9 (177.3 to 307.8)	164.7 (119.8 to 211.3)	207.7 (152.1 to 263.7)	244.7 (179.4 to 309.5)	172.2 (125.3 to 217.8)	2 (-0.3 to 4.4)	0.8 (-2 to 3.8)	4.5 (1.6 to 7.6)

Data in parentheses are 95% Uncertainty Intervals (95% UIs)

DALYs=Disability-Adjusted Life Years; YLLs=Years of Life Lost; YLDs=Years Lived with Disability

The attributed burden to all risk factors quantifies the burden caused by the stroke which can be attributed to known risk factors.
