# Supplementary material for: Burden of stroke in North Africa and Middle East, 1990 to 2019: a systematic analysis for the global burden of disease study 2019
Source: BMC Neurol. 2022 Jul 27;22:279. doi: 10.1186/s12883-022-02793-0 (PMC9327376; doi:10.1186/s12883-022-02793-0)
Supplement: Supplementary file 3 — Additional file 3: Supplementary Table 2. Age-standardized stroke burden in 1990 and 2019 at each country of the super-region [file 12883_2022_2793_MOESM3_ESM.pdf]

Country	Measure	Age-standardized rate (per 100,000)						% Change (1990 to 2019)		
		1990			2019					
		Both	Female	Male	Both	Female	Male	Both	Female	Male
**Afghanistan**	**Incidence**	235.9 (218.7 to 254.4)	253.7 (234.7 to 274.5)	215.5 (196.7 to 235.3)	217.7 (200.8 to 238.5)	229.5 (211.3 to 251.2)	207.3 (187.2 to 229.5)	-7.7 (-11.8 to -3.2)	-9.6 (-15 to -3.7)	-3.8 (-9.1 to 2.5)
	**Prevalence**	1597.6 (1485.6 to 1723.9)	1827.8 (1688 to 1974.4)	1363.9 (1256.8 to 1483)	1657.6 (1533.4 to 1779.5)	1875.8 (1738.2 to 2022.4)	1434.7 (1306.5 to 1561.9)	3.8 (-1.2 to 8)	2.6 (-3.2 to 8.1)	5.2 (-1.6 to 11.1)
	**Deaths**	198.6 (135.7 to 257.5)	219.5 (136.8 to 288.7)	177.9 (122 to 236.7)	161.5 (110.3 to 208.5)	184.9 (118 to 245.3)	137.3 (98.4 to 184.1)	-18.7 (-36.1 to -1.6)	-15.7 (-35.7 to 6.5)	-22.8 (-40.4 to -4.1)
	**DALYs**	4471.6 (3212.1 to 5710.6)	5125.9 (3559 to 6647.3)	3780.2 (2694.7 to 5062.8)	3498.2 (2508.8 to 4500.4)	4097.7 (2827.9 to 5410.5)	2878.5 (2109 to 3743.7)	-21.8 (-39.4 to -3.6)	-20.1 (-39.3 to 2.6)	-23.9 (-40.9 to -2.9)
	**YLLs**	4231.2 (2971.5 to 5515.6)	4835 (3252.5 to 6357.9)	3589.8 (2507.4 to 4861.4)	3249.7 (2264.5 to 4235.5)	3800.7 (2532.4 to 5132)	2680.7 (1917.4 to 3551.8)	-23.2 (-41.6 to -4)	-21.4 (-41.4 to 2.6)	-25.3 (-43.2 to -3.5)
	**YLDs**	240.4 (175.9 to 301.7)	290.8 (211.4 to 366.3)	190.4 (137.2 to 241)	248.5 (183.7 to 312)	297 (219.2 to 373.6)	197.8 (142.4 to 250.4)	3.4 (-2.1 to 8.1)	2.1 (-4 to 8.7)	3.9 (-3.4 to 11.1)
**Algeria**	**Incidence**	218.7 (200 to 239.8)	233.8 (212 to 257.3)	204.1 (185.3 to 226.1)	181.7 (164.3 to 202.2)	195.6 (176.7 to 217)	169.4 (150.9 to 190.9)	-16.9 (-21.1 to -12.3)	-16.3 (-22.1 to -10.5)	-17 (-22.8 to -9.8)
	**Prevalence**	1724.6 (1603.2 to 1858.9)	1921.5 (1776.1 to 2069.8)	1532.1 (1410.2 to 1671.4)	1540.3 (1417.2 to 1668)	1720 (1573 to 1873.1)	1369.2 (1244.9 to 1493.4)	-10.7 (-14.5 to -7)	-10.5 (-15.9 to -5.3)	-10.6 (-15.6 to -5.2)
	**Deaths**	179.4 (149.3 to 211.2)	198.1 (163.1 to 233.8)	167.9 (132.7 to 206.8)	101.5 (82.6 to 121.6)	120.7 (93 to 144.9)	89.5 (70.1 to 112.3)	-43.4 (-54.9 to -30.2)	-39.1 (-51.7 to -24.3)	-46.7 (-58.8 to -31.1)
	**DALYs**	3167.5 (2642 to 3755.9)	3421.3 (2800.4 to 4066.2)	2977 (2353.8 to 3676.1)	1755.4 (1459.6 to 2081.9)	1979.9 (1585.6 to 2345.6)	1586.9 (1262.8 to 1965.2)	-44.6 (-55 to -31.9)	-42.1 (-53.2 to -29.7)	-46.7 (-58.6 to -31.8)
	**YLLs**	2895.9 (2372.6 to 3490)	3099.4 (2467.5 to 3737.7)	2754.6 (2140.4 to 3446.9)	1512.6 (1223 to 1842.1)	1691.6 (1306.1 to 2042)	1387.4 (1058.8 to 1764.7)	-47.8 (-58.8 to -34)	-45.4 (-57 to -32)	-49.6 (-61.7 to -34)
	**YLDs**	271.7 (197.5 to 343.9)	322 (235.6 to 405)	222.4 (162 to 283.9)	242.8 (178.6 to 307.3)	288.3 (211.9 to 368.1)	199.5 (145.5 to 252.4)	-10.6 (-14.9 to -6)	-10.5 (-16.5 to -4.5)	-10.3 (-15.9 to -3.7)

Country	Measure	Age-standardized rate (per 100,000)						% Change (1990 to 2019)		
		1990			2019					
		Both	Female	Male	Both	Female	Male	Both	Female	Male
**Bahrain**	**Incidence**	168.7 (153.4 to 186.1)	192.8 (173.7 to 214.4)	147.6 (133.5 to 163.3)	113.4 (102.3 to 126.6)	129.3 (115.6 to 145.1)	102.3 (91.8 to 115)	-32.8 (-36.8 to -28.7)	-32.9 (-38.2 to -27.6)	-30.7 (-35.7 to -25.3)
	**Prevalence**	1499.7 (1387.3 to 1636.7)	1776.4 (1629.9 to 1946.5)	1261.5 (1160.6 to 1372.1)	1136.4 (1049 to 1234.8)	1343.4 (1231 to 1478.1)	993.7 (916.1 to 1082.9)	-24.2 (-27.5 to -20.8)	-24.4 (-28.9 to -19.8)	-21.2 (-25.7 to -16.5)
	**Deaths**	102.5 (90.2 to 118.9)	108.1 (94.2 to 123.6)	96.7 (82.7 to 123.3)	52.8 (43.5 to 66.9)	58.3 (47.3 to 70.6)	47.7 (38.7 to 68.2)	-48.5 (-58.4 to -36.5)	-46.1 (-57.8 to -31.2)	-50.6 (-61.4 to -37.7)
	**DALYs**	1981.3 (1768.7 to 2241.6)	2180.5 (1927.8 to 2463.4)	1811.4 (1579.4 to 2135.9)	940.9 (796.8 to 1146.2)	1069.9 (905.6 to 1266.2)	842.5 (698.9 to 1104.8)	-52.5 (-61.2 to -42.5)	-50.9 (-60 to -39.8)	-53.5 (-62.8 to -42.5)
	**YLLs**	1746.7 (1538.4 to 2013.1)	1884.5 (1645.3 to 2159.1)	1630.3 (1398.1 to 1971.2)	767 (633.5 to 977.6)	849.9 (694.6 to 1033.5)	701.2 (565 to 965.8)	-56.1 (-65 to -45)	-54.9 (-64.8 to -42.3)	-57 (-66.4 to -44.7)
	**YLDs**	234.6 (172 to 295.1)	295.9 (217.6 to 373.6)	181.1 (131.3 to 228.7)	173.8 (126 to 219.2)	220 (158.3 to 278.9)	141.3 (102.6 to 178.3)	-25.9 (-30 to -21.8)	-25.6 (-31 to -20.2)	-22 (-27.5 to -16)
**Egypt**	**Incidence**	208.4 (190.8 to 229.2)	225.8 (205.1 to 248.5)	190.9 (174.1 to 211.6)	228.4 (205 to 255.3)	263.1 (232.7 to 295.2)	202.8 (180.4 to 229.1)	9.6 (3.5 to 16.5)	16.5 (7.7 to 25.9)	6.2 (-1.1 to 14.4)
	**Prevalence**	1523.8 (1408.7 to 1660.1)	1713.4 (1573.1 to 1881.9)	1333.5 (1225.8 to 1457.5)	1806.1 (1658.1 to 1974.5)	2109.9 (1929.1 to 2306.9)	1561.1 (1415.7 to 1723.1)	18.5 (12.9 to 23.9)	23.1 (15.2 to 30.7)	17.1 (9.8 to 24.2)
	**Deaths**	110.8 (93.9 to 129)	113.5 (89.4 to 131.5)	107.9 (92 to 133.7)	85.7 (63.1 to 118.9)	106 (74.7 to 146.9)	76 (53.2 to 111.2)	-22.6 (-42.6 to 1.6)	-6.7 (-31.3 to 23.7)	-29.6 (-48.5 to -4.9)
	**DALYs**	3316 (2889.1 to 3785.7)	3159.1 (2603.7 to 3729.5)	3456.7 (2952.4 to 4055.5)	2138 (1636.8 to 2809.8)	2331.5 (1745.9 to 3073.2)	2050.6 (1517.6 to 2794.5)	-35.5 (-50 to -17)	-26.2 (-43.6 to -4.9)	-40.7 (-56.8 to -20.4)
	**YLLs**	3076.6 (2652.9 to 3538)	2873.7 (2308.1 to 3436.4)	3263.5 (2769.8 to 3852.6)	1855.4 (1367.5 to 2533.1)	1977.9 (1421.1 to 2695)	1825.1 (1299.2 to 2565.1)	-39.7 (-55.1 to -19.7)	-31.2 (-50.2 to -8)	-44.1 (-61.1 to -22.6)
	**YLDs**	239.4 (173.2 to 304.9)	285.4 (205.8 to 363.6)	193.1 (138.5 to 247.7)	282.6 (206.4 to 356.5)	353.6 (259.8 to 445.5)	225.6 (162.3 to 287.3)	18 (11.8 to 24.2)	23.9 (15.1 to 32.4)	16.8 (8.7 to 25)

Country	Measure	Age-standardized rate (per 100,000)						% Change (1990 to 2019)		
		1990			2019					
		Both	Female	Male	Both	Female	Male	Both	Female	Male
Iran (Islamic Republic of)	Incidence	166.6 (146.6 to 190.9)	166 (146.4 to 189.8)	167.8 (146.9 to 192.1)	138.8 (121.8 to 159.6)	143.7 (125.3 to 165.6)	134 (117.3 to 154.1)	-16.7 (-18.1 to -15.1)	-13.4 (-15.7 to -10.7)	-20.1 (-21.9 to -18.3)
	Prevalence	1446.9 (1279.8 to 1649.5)	1590.6 (1412.9 to 1796.1)	1306.1 (1144.4 to 1502.8)	1253.8 (1113.5 to 1418.4)	1349.7 (1199.5 to 1520.2)	1159.3 (1022.9 to 1319.6)	-13.3 (-15.9 to -10.7)	-15.1 (-17.9 to -12.3)	-11.2 (-14.9 to -7.3)
	Deaths	120.7 (102.8 to 133.5)	119.3 (97.9 to 132.8)	120.5 (99.8 to 136.2)	66.2 (58.7 to 71.3)	68.2 (59 to 74.4)	64.8 (57.3 to 70.3)	-45.1 (-50.6 to -35.4)	-42.8 (-49 to -28.2)	-46.3 (-53.9 to -37.9)
	DALYs	2324.3 (2051.8 to 2547)	2273.2 (1968 to 2521.3)	2353.7 (2039.8 to 2680)	1262.2 (1153.5 to 1346.3)	1252.8 (1126.4 to 1360.2)	1274.9 (1173.4 to 1371.5)	-45.7 (-51 to -38.3)	-44.9 (-50.5 to -34.8)	-45.8 (-52.2 to -38.4)
	YLLs	2099.4 (1838.1 to 2315.2)	2010.4 (1714.9 to 2235)	2165.6 (1872.3 to 2471.9)	1065.8 (976.7 to 1134.4)	1027.6 (914.9 to 1114.7)	1107.2 (1008.5 to 1192.1)	-49.2 (-54.5 to -41.4)	-48.9 (-54.7 to -38)	-48.9 (-55.5 to -41.1)
	YLDs	225 (161.2 to 290.2)	262.8 (188.6 to 336.4)	188.1 (135 to 244.4)	196.4 (140.3 to 251.9)	225.2 (162.4 to 288.2)	167.7 (119.6 to 216.7)	-12.7 (-15.5 to -10)	-14.3 (-17.2 to -11.4)	-10.9 (-14.6 to -6.8)
Iraq	Incidence	273.7 (249.1 to 300.1)	283 (256.6 to 313.4)	263.6 (239.4 to 291.4)	241.6 (216.9 to 267)	251.8 (223.5 to 279)	229 (205.5 to 255.4)	-11.7 (-16.4 to -6.7)	-11 (-17.8 to -3.8)	-13.1 (-18.8 to -6.7)
	Prevalence	2177.9 (2027.8 to 2349.2)	2361 (2194.4 to 2551.7)	1988.1 (1830.6 to 2150.2)	1968.8 (1823.4 to 2122.1)	2125.9 (1956.5 to 2301.3)	1798.1 (1653.6 to 1955.1)	-9.6 (-13.3 to -5.4)	-10 (-15.6 to -4.3)	-9.6 (-15 to -3.7)
	Deaths	166.1 (140.6 to 193.9)	159.8 (131.4 to 190.3)	171.8 (138.4 to 212.7)	143.3 (119.2 to 166.1)	130.4 (108.1 to 152.5)	156.6 (129.2 to 180.7)	-13.7 (-30.2 to 4.5)	-18.4 (-36.2 to 2.6)	-8.8 (-29.2 to 16.3)
	DALYs	3625.9 (3103.7 to 4212.9)	3440.4 (2892.9 to 4034.8)	3806.5 (3068.5 to 4676.9)	2922.9 (2399 to 3459.1)	2633.5 (2172.9 to 3105.6)	3215.2 (2605.8 to 3837.4)	-19.4 (-35.5 to -0.8)	-23.5 (-40.2 to -3.7)	-15.5 (-35.2 to 9.5)
	YLLs	3290.4 (2783.1 to 3856.6)	3051.6 (2496.5 to 3667.3)	3526.5 (2786 to 4394.2)	2616.5 (2102.7 to 3132.9)	2279.1 (1838 to 2745.4)	2960.2 (2363.2 to 3565.5)	-20.5 (-38.2 to 0)	-25.3 (-44.1 to -2.8)	-16.1 (-36.8 to 11.4)
	YLDs	335.5 (247.9 to 421)	388.7 (286.6 to 486.3)	280 (203.4 to 354.4)	306.4 (223.5 to 385.2)	354.5 (259.3 to 446.4)	255 (185.7 to 319.6)	-8.7 (-13.3 to -3.7)	-8.8 (-15 to -2)	-9 (-15.8 to -1.7)

Country	Measure	Age-standardized rate (per 100,000)						% Change (1990 to 2019)		
		1990			2019					
		Both	Female	Male	Both	Female	Male	Both	Female	Male
Jordan	Incidence	306 (274.1 to 345.1)	330.4 (295.3 to 371.9)	281.8 (252.1 to 319)	225.9 (200.5 to 253.4)	238.1 (211.1 to 268.8)	214.8 (188.9 to 242.4)	-26.2 (-30.9 to -20.8)	-27.9 (-34.1 to -20.7)	-23.8 (-30.3 to -16.5)
	Prevalence	2342.5 (2150.9 to 2519.7)	2636.2 (2425.5 to 2829.1)	2054.6 (1854.3 to 2240.7)	1793.9 (1621.2 to 1952.9)	2009 (1844.9 to 2184)	1600.9 (1402.7 to 1795.5)	-23.4 (-27.7 to -19.5)	-23.8 (-28.4 to -18.7)	-22.1 (-28.9 to -15.1)
	Deaths	150.9 (123.6 to 175.4)	179 (143 to 212.1)	122.5 (90.6 to 147.9)	75.7 (61.3 to 89)	83.4 (66.9 to 100.8)	69.3 (49.6 to 86.9)	-49.8 (-59.4 to -39.2)	-53.4 (-63.9 to -39.7)	-43.5 (-57.7 to -26)
	DALYs	2910 (2466.9 to 3357.8)	3397.8 (2813.2 to 3942)	2420 (1823.8 to 2868.7)	1448 (1220.4 to 1679.5)	1567.2 (1301 to 1843.4)	1341.5 (1037.4 to 1637.5)	-50.2 (-58.7 to -41.1)	-53.9 (-63 to -42.5)	-44.6 (-57.6 to -29.6)
	YLLs	2537.3 (2107.4 to 2961.2)	2951.2 (2380.7 to 3505.7)	2120 (1569.5 to 2582.4)	1163.5 (954.9 to 1374.6)	1225.7 (976.2 to 1498.5)	1108.5 (804.4 to 1401.8)	-54.1 (-63.2 to -43.8)	-58.5 (-68 to -45.7)	-47.7 (-62 to -30.7)
	YLDs	372.8 (275.9 to 467.3)	446.6 (326.1 to 560.9)	300 (218.2 to 380)	284.5 (207.6 to 358.1)	341.5 (250.3 to 429.2)	233.1 (167.4 to 299.5)	-23.7 (-28.5 to -19.2)	-23.5 (-28.6 to -18.2)	-22.3 (-30.3 to -14.4)
Kuwait	Incidence	129.3 (117.8 to 143.2)	143.2 (128.1 to 159.1)	118.8 (107.6 to 132.7)	125.8 (113.7 to 140.4)	114.2 (101.9 to 127.3)	133.2 (119.3 to 150.4)	-2.7 (-8.2 to 3.3)	-20.3 (-25.9 to -13.8)	12.1 (3.5 to 21.2)
	Prevalence	1307.7 (1215 to 1402.4)	1494.9 (1380.5 to 1620.6)	1182.3 (1088.2 to 1273)	1230.6 (1134.6 to 1332.5)	1258.3 (1158.6 to 1368.3)	1203.7 (1101.4 to 1315.5)	-5.9 (-10.4 to -1.4)	-15.8 (-20.9 to -10.7)	1.8 (-4.8 to 8.4)
	Deaths	51.5 (44.4 to 57.6)	57 (48.1 to 65.9)	45.9 (39.8 to 51.1)	46.5 (38 to 55.2)	35.1 (27.5 to 42.5)	54.1 (43.1 to 66)	-9.6 (-24.2 to 7.7)	-38.5 (-49.2 to -25.6)	17.8 (-3.8 to 44)
	DALYs	1062.6 (953.7 to 1169.4)	1196.2 (1049.2 to 1358.7)	962.9 (865.1 to 1060)	938.4 (795 to 1093.3)	733.2 (616.2 to 859.1)	1078 (885.2 to 1296.6)	-11.7 (-24 to 3.3)	-38.7 (-47.5 to -28.4)	12 (-7.3 to 34.4)
	YLLs	859.4 (762.7 to 948.7)	946.3 (815 to 1091.9)	791.2 (700.7 to 872.7)	749 (616.5 to 896.1)	523.9 (417.7 to 632.2)	903.6 (721.1 to 1118.7)	-12.8 (-27.8 to 5.7)	-44.6 (-54.8 to -31.9)	14.2 (-9.2 to 42)
	YLDs	203.3 (149.1 to 254.2)	249.9 (183.9 to 314.9)	171.6 (124.6 to 215.7)	189.4 (138.2 to 238)	209.2 (152.9 to 265.5)	174.3 (125.5 to 218.8)	-6.8 (-12.1 to -1.3)	-16.3 (-22.2 to -10.1)	1.6 (-6.2 to 10)

Country	Measure	Age-standardized rate (per 100,000)						% Change (1990 to 2019)		
		1990			2019					
		Both	Female	Male	Both	Female	Male	Both	Female	Male
Lebanon	Incidence	158.9 (144.4 to 175.1)	191.1 (172.2 to 213)	125.8 (113.8 to 139.9)	150.5 (135.7 to 167.8)	171.4 (152.6 to 193.8)	125.8 (113.2 to 140)	-5.3 (-9.9 to 0.3)	-10.3 (-17 to -3.4)	0 (-7 to 8)
	Prevalence	1395 (1289.8 to 1503.7)	1681.8 (1553.6 to 1818.8)	1095.5 (1005 to 1193.1)	1425.1 (1320.3 to 1538.1)	1637.2 (1503.3 to 1770.9)	1173.9 (1075 to 1276)	2.2 (-1.8 to 6.6)	-2.7 (-7.9 to 3.1)	7.1 (0.7 to 13)
	Deaths	58.1 (48.4 to 69.3)	62.9 (50.8 to 77.4)	51.9 (42 to 63)	35.2 (24.5 to 45.6)	34.6 (22.6 to 45.5)	35.6 (24.7 to 48.4)	-39.5 (-55.5 to -19.2)	-45 (-60.1 to -23.2)	-31.4 (-54.4 to -5)
	DALYs	1135.7 (980.3 to 1327.2)	1258.1 (1068.7 to 1486.1)	998.4 (832.2 to 1199.4)	752.9 (593.3 to 935.9)	776.9 (600.7 to 968.8)	722.7 (539.9 to 931.9)	-33.7 (-47.6 to -16)	-38.2 (-52.3 to -21)	-27.6 (-46.4 to -5.2)
	YLLs	915.1 (774.3 to 1101.2)	977.9 (802.1 to 1201.3)	840.3 (690.3 to 1035)	528.3 (372.5 to 692.1)	504.8 (337.8 to 675.3)	554.4 (377.8 to 765.6)	-42.3 (-59.3 to -21.3)	-48.4 (-63.8 to -26.6)	-34 (-57.7 to -7.6)
	YLDs	220.5 (159.4 to 277.9)	280.2 (203.4 to 354.5)	158.1 (113.7 to 200.8)	224.6 (165.6 to 281.1)	272.1 (199.6 to 341.7)	168.3 (124 to 213.4)	1.8 (-2.8 to 7.2)	-2.9 (-8.9 to 3.7)	6.5 (-0.5 to 14.6)
Libya	Incidence	158.4 (144.6 to 173.8)	189.7 (172.3 to 209.8)	131.3 (118.5 to 146.9)	170.9 (153.6 to 190.3)	205.9 (184.1 to 231.3)	137.3 (122.2 to 154.6)	7.9 (2.1 to 14.6)	8.6 (0.6 to 17.3)	4.6 (-3.1 to 13.6)
	Prevalence	1393.6 (1294.4 to 1505.9)	1722.8 (1593.9 to 1863.9)	1107.3 (1017.3 to 1206)	1588.4 (1461.7 to 1712.9)	1962.6 (1805.7 to 2124.5)	1230.9 (1123.6 to 1346.7)	14 (8.9 to 18.7)	13.9 (7.9 to 20.4)	11.2 (4.7 to 18.2)
	Deaths	84.6 (66.2 to 107)	90.1 (68.5 to 116.3)	79.7 (61.3 to 104)	69.4 (53 to 90.1)	73.3 (53.5 to 94.3)	65.4 (47.3 to 94.3)	-17.9 (-36.4 to 7.9)	-18.6 (-38.6 to 7.5)	-18 (-39.2 to 13.5)
	DALYs	1996.7 (1628.5 to 2421.5)	2181.7 (1758.3 to 2699.4)	1845.5 (1452.1 to 2305.6)	1570.5 (1237.3 to 2001.2)	1728.1 (1324.9 to 2172.1)	1418.4 (1048.8 to 1982)	-21.3 (-37.5 to 1.4)	-20.8 (-37.9 to 1.1)	-23.1 (-43.4 to 5.8)
	YLLs	1776.7 (1419.8 to 2228.9)	1894.6 (1468.8 to 2408.6)	1684.7 (1291.8 to 2149.2)	1322.4 (1000.5 to 1742.3)	1404.2 (1014.8 to 1834.1)	1242.9 (885.9 to 1792.2)	-25.6 (-43.4 to 0.2)	-25.9 (-44.4 to -1.3)	-26.2 (-47.4 to 5.6)
	YLDs	220 (160.5 to 278.1)	287.1 (210 to 361.9)	160.9 (117.4 to 204.8)	248.1 (179.9 to 311)	323.9 (237.3 to 405.4)	175.6 (126.9 to 221.7)	12.8 (6.9 to 18.8)	12.8 (5.7 to 20.4)	9.1 (2.1 to 17.3)

Country	Measure	Age-standardized rate (per 100,000)						% Change (1990 to 2019)		
		1990			2019					
		Both	Female	Male	Both	Female	Male	Both	Female	Male
Morocco	Incidence	208.9 (191.2 to 230.2)	222 (201.3 to 245.1)	195.8 (178.4 to 216.7)	199.8 (179.8 to 222)	218 (194.3 to 242.4)	181.4 (161.4 to 202.4)	-4.4 (-9.8 to 1.5)	-1.8 (-9 to 6.4)	-7.4 (-14.4 to 0)
	Prevalence	1638.9 (1517 to 1774.3)	1826.2 (1688 to 1978.3)	1442.7 (1326.7 to 1568.7)	1695.1 (1563.4 to 1833.2)	1933.9 (1777.3 to 2096.9)	1451.1 (1326 to 1578.6)	3.4 (-0.7 to 8.2)	5.9 (-0.1 to 12.4)	0.6 (-5.3 to 6.9)
	Deaths	132.2 (102.8 to 158.4)	132.2 (98.9 to 164)	132.7 (102 to 169.1)	116.4 (94.2 to 139.3)	115.8 (90.7 to 142.7)	117.1 (90.3 to 148.3)	-11.9 (-29.1 to 8)	-12.4 (-31.4 to 12)	-11.7 (-32.6 to 11.5)
	DALYs	2727.6 (2237.6 to 3235.4)	2805.4 (2199.3 to 3452)	2650.6 (2098.7 to 3299.8)	2257.8 (1838.8 to 2702.5)	2326.5 (1862.9 to 2840.5)	2189.2 (1716.8 to 2775.9)	-17.2 (-33.9 to 0.9)	-17.1 (-34.6 to 4.5)	-17.4 (-36.5 to 4)
	YLLs	2471.3 (1965.4 to 2986.8)	2502.3 (1924.2 to 3113.9)	2443.1 (1879 to 3075.6)	1992.7 (1584.1 to 2446.6)	2006.4 (1542 to 2524.3)	1980.4 (1507.2 to 2565.6)	-19.4 (-37.5 to 0.5)	-19.8 (-38.9 to 3.6)	-18.9 (-39.1 to 4.2)
	YLDs	256.4 (188.2 to 324.8)	303.2 (221.2 to 383.9)	207.6 (151.9 to 265.5)	265.1 (194.2 to 333)	320.1 (232.8 to 400.3)	208.8 (152.3 to 263.9)	3.4 (-1.4 to 9.1)	5.6 (-1.1 to 12.9)	0.6 (-6.5 to 7.6)
Oman	Incidence	218.7 (199.5 to 241)	218.7 (201.1 to 239.9)	220.3 (197.6 to 248.1)	196.2 (174.8 to 222.1)	196.7 (174.6 to 220.9)	197.2 (173.3 to 226.1)	-10.3 (-15.9 to -4.6)	-10.1 (-16.8 to -2.7)	-10.5 (-17.5 to -2.3)
	Prevalence	1666.8 (1535.5 to 1837.5)	1708.3 (1573.9 to 1868.3)	1654 (1503.8 to 1846)	1525.6 (1399.9 to 1686.6)	1620.9 (1485.5 to 1782.5)	1470.3 (1336.8 to 1639.5)	-8.5 (-12.4 to -4.5)	-5.1 (-10.6 to 0.5)	-11.1 (-16.7 to -5.2)
	Deaths	145.7 (112.8 to 183)	132.9 (99.7 to 172.9)	164.6 (123.6 to 211.7)	103.7 (90.7 to 118.7)	96.2 (81.3 to 112.7)	113.6 (94.9 to 135.1)	-28.8 (-45 to -3.7)	-27.6 (-47.4 to 2.6)	-31 (-48.8 to -3.5)
	DALYs	3071.8 (2428.5 to 3824)	2880.2 (2254.5 to 3648.2)	3309.8 (2481.3 to 4230.7)	1884.3 (1667.3 to 2127.1)	1850.6 (1600.6 to 2133.9)	1954.3 (1645.1 to 2297.2)	-38.7 (-51.9 to -20.7)	-35.7 (-51.4 to -13.8)	-41 (-55.7 to -19.3)
	YLLs	2813.8 (2171.3 to 3569.2)	2597.3 (1982 to 3336.9)	3070.4 (2265.1 to 3987.7)	1647.1 (1439.9 to 1874.5)	1579.7 (1341.4 to 1850.4)	1740.8 (1444.4 to 2086.9)	-41.5 (-55 to -21.9)	-39.2 (-55.6 to -15.2)	-43.3 (-58.5 to -20.2)
	YLDs	258 (187.8 to 325.9)	283 (204.6 to 359.2)	239.4 (173.3 to 305.9)	237.2 (174.1 to 301.6)	270.9 (199.5 to 347)	213.5 (154 to 271)	-8.1 (-12.8 to -3)	-4.3 (-10.5 to 2.8)	-10.8 (-17.6 to -4.7)

Country	Measure	Age-standardized rate (per 100,000)						% Change (1990 to 2019)		
		1990			2019					
		Both	Female	Male	Both	Female	Male	Both	Female	Male
Palestine	Incidence	205.3 (186.9 to 227.2)	219.9 (198.4 to 245.9)	189.1 (170.9 to 208.4)	189.7 (170.1 to 212.2)	204.9 (181.5 to 232.3)	174.1 (155.7 to 195.4)	-7.6 (-13.7 to -1.7)	-6.8 (-15.3 to 1.3)	-7.9 (-15.2 to -0.6)
	Prevalence	1569.1 (1439.1 to 1706)	1757.9 (1608.1 to 1909.3)	1346.3 (1216.9 to 1487)	1510.8 (1381.5 to 1666.5)	1709.5 (1551.3 to 1891.4)	1288.3 (1164.9 to 1422)	-3.7 (-8.5 to 1.4)	-2.8 (-9.1 to 4)	-4.3 (-11.3 to 2.9)
	Deaths	172.5 (141 to 208.3)	167.4 (134.4 to 203.8)	180.5 (143.7 to 218.9)	122.4 (105.6 to 138.2)	120.3 (103.4 to 137.5)	128.2 (106.9 to 146.6)	-29.1 (-43.5 to -10.1)	-28.1 (-43.6 to -8.9)	-29 (-44.2 to -8.9)
	DALYs	3154.1 (2576 to 3790.5)	3092.5 (2534.4 to 3756.6)	3242 (2606.5 to 3942.7)	2128.3 (1878.2 to 2399.7)	2125.8 (1871.5 to 2402.7)	2143.7 (1826.8 to 2453.1)	-32.5 (-46.4 to -15)	-31.3 (-45.2 to -14)	-33.9 (-48.2 to -14.8)
	YLLs	2906.5 (2327.1 to 3569.6)	2799.7 (2247.9 to 3452.3)	3047.8 (2405.3 to 3732.9)	1890.3 (1632.7 to 2140.5)	1840.8 (1585.3 to 2106.6)	1958.7 (1635.9 to 2263.7)	-35 (-49.6 to -16.4)	-34.3 (-49 to -15.3)	-35.7 (-50.5 to -15.8)
	YLDs	247.6 (182.7 to 312.7)	292.8 (214.3 to 366.6)	194.2 (141.2 to 247.4)	238 (173.6 to 302.4)	285.1 (206.6 to 364.4)	185 (134.4 to 235.8)	-3.9 (-9.4 to 1.6)	-2.6 (-9.7 to 5.6)	-4.7 (-13.1 to 3.7)
Qatar	Incidence	172 (156.7 to 190)	194 (175.9 to 213.7)	155.9 (140.2 to 174.9)	127.1 (114.7 to 141.6)	153.6 (138.5 to 170.8)	118.5 (106.3 to 133)	-26.1 (-30.3 to -21.7)	-20.8 (-26.3 to -14.7)	-24 (-29.5 to -18.1)
	Prevalence	1575.7 (1457.5 to 1705.2)	1842 (1703.7 to 1986.7)	1421.7 (1304.3 to 1545.9)	1226.3 (1127.4 to 1327.2)	1494.2 (1374.6 to 1608.3)	1138.8 (1041.5 to 1238.7)	-22.2 (-25.6 to -18.9)	-18.9 (-23.4 to -14.5)	-19.9 (-24.2 to -15.3)
	Deaths	83.5 (63.4 to 108.7)	87.7 (62.1 to 113.5)	78.4 (60.2 to 104.9)	52.9 (42.5 to 69.2)	85.3 (67.3 to 107.4)	44.5 (33.8 to 60.1)	-36.7 (-54 to -15.7)	-2.7 (-30.9 to 40.9)	-43.2 (-60.2 to -20)
	DALYs	1635.2 (1336.8 to 2028.2)	1810.5 (1411.2 to 2251)	1535.9 (1234 to 1994.8)	904.5 (747.6 to 1130.3)	1337.1 (1100.6 to 1637.6)	777.6 (615.8 to 1012.7)	-44.7 (-57.5 to -29.5)	-26.1 (-44.6 to -1.3)	-49.4 (-62.2 to -32.8)
	YLLs	1394.8 (1089.9 to 1796)	1507.8 (1102.5 to 1942.9)	1333.4 (1023 to 1773.9)	722.3 (572.2 to 941)	1093.5 (869.8 to 1382.2)	615.6 (462.5 to 844.9)	-48.2 (-62 to -31)	-27.5 (-48.5 to 4.6)	-53.8 (-67.9 to -35.2)
	YLDs	240.4 (176 to 302)	302.7 (221.4 to 378.9)	202.6 (146.9 to 256.8)	182.2 (132.5 to 228.2)	243.6 (177.9 to 307.5)	162 (117.2 to 203.8)	-24.2 (-28.2 to -19.9)	-19.5 (-24.8 to -14.4)	-20 (-25.4 to -14)

Country	Measure	Age-standardized rate (per 100,000)						% Change (1990 to 2019)		
		1990			2019					
		Both	Female	Male	Both	Female	Male	Both	Female	Male
Saudi Arabia	Incidence	173.2 (157.5 to 188.9)	184.1 (166.2 to 202.2)	164.7 (149.7 to 181.5)	193.9 (176.5 to 213.7)	208.5 (189.1 to 230.9)	183 (165.3 to 203.9)	11.9 (6.7 to 18)	13.3 (5.8 to 22.4)	11.1 (3.3 to 19.2)
	Prevalence	1823.4 (1631.5 to 2105.7)	2116.9 (1855.1 to 2480.5)	1606.9 (1452.2 to 1828.2)	1967.7 (1818.1 to 2143.5)	2303.7 (2114.7 to 2542.3)	1737.6 (1598.1 to 1892.9)	7.9 (-0.1 to 15.2)	8.8 (-1.8 to 18.8)	8.1 (0.3 to 15.7)
	Deaths	152.2 (119.3 to 186.2)	155.6 (119.3 to 192.9)	149.4 (109.4 to 184)	102.7 (80.4 to 120.5)	103.9 (80.9 to 128.2)	101 (71.5 to 118.8)	-32.5 (-49.1 to -10)	-33.3 (-52.2 to -8)	-32.4 (-48.6 to -7.4)
	DALYs	2982.3 (2362.6 to 3635)	3156.7 (2497.6 to 3896.3)	2859.9 (2110.2 to 3582.5)	2114.9 (1685.1 to 2485.5)	2193.9 (1787.9 to 2684.8)	2054.3 (1550.6 to 2417.9)	-29.1 (-46.4 to -5.6)	-30.5 (-48.8 to -6.9)	-28.2 (-46.2 to -1.7)
	YLLs	2703.1 (2094.2 to 3361.1)	2809.1 (2141.4 to 3546)	2631.5 (1898.1 to 3338.9)	1815.5 (1408.2 to 2165.5)	1814.6 (1398.7 to 2271.4)	1809.4 (1315.1 to 2152.9)	-32.8 (-51 to -7.4)	-35.4 (-54.6 to -9.4)	-31.2 (-49.5 to -2.6)
	YLDs	279.2 (202.4 to 366.3)	347.6 (245.7 to 463.1)	228.4 (162.9 to 295.8)	299.4 (218.9 to 378.4)	379.2 (277.3 to 480.2)	244.9 (178 to 311.8)	7.2 (-1.5 to 15.6)	9.1 (-2 to 19.9)	7.2 (-1.4 to 16.4)
Sudan	Incidence	238.5 (219.3 to 261.5)	258.3 (235.7 to 284.5)	219.6 (200.5 to 242)	227.1 (205 to 253.2)	251.4 (224.3 to 281.3)	205.6 (184.8 to 230.6)	-4.8 (-9.7 to 0.9)	-2.7 (-9.1 to 4.2)	-6.4 (-12.2 to 0.4)
	Prevalence	1650.5 (1527.1 to 1787.5)	1865.9 (1720.9 to 2030.5)	1447 (1330.1 to 1580)	1785.8 (1645.2 to 1936.9)	2055.9 (1879.7 to 2248.7)	1551.6 (1411.7 to 1686.8)	8.2 (3.7 to 12.8)	10.2 (3.8 to 16.3)	7.2 (1.5 to 13.4)
	Deaths	177.4 (127.6 to 228.6)	183.3 (126.2 to 232.8)	171.7 (121.1 to 235.2)	125.2 (92.6 to 174.9)	133.2 (95.2 to 177.3)	118.3 (82.4 to 182.8)	-29.4 (-42.2 to -12)	-27.3 (-40 to -9.2)	-31.1 (-46 to -11.2)
	DALYs	3858.9 (2972.6 to 4948.3)	4109.5 (3028.9 to 5205.6)	3619 (2618.1 to 4954.6)	2585.5 (1970.5 to 3552.9)	2783.9 (2061.8 to 3639.3)	2415.2 (1713.6 to 3693.4)	-33 (-45.3 to -16.9)	-32.3 (-44.4 to -16.2)	-33.3 (-48.2 to -13.2)
	YLLs	3603.3 (2717.3 to 4677.2)	3801.2 (2704.2 to 4909.9)	3412.7 (2448.8 to 4749.2)	2309 (1690.7 to 3286.2)	2443.2 (1714.4 to 3253.6)	2194 (1481.8 to 3466.6)	-35.9 (-49.3 to -18.7)	-35.7 (-48.5 to -18.9)	-35.7 (-51.6 to -14.8)
	YLDs	255.7 (185 to 323)	308.4 (224.3 to 391.1)	206.3 (147.6 to 262.4)	276.5 (201.3 to 351.5)	340.7 (248.5 to 435.3)	221.2 (159.2 to 279.8)	8.2 (2.6 to 13.3)	10.5 (3.1 to 17.4)	7.2 (-0.1 to 14.6)

Country	Measure	Age-standardized rate (per 100,000)						% Change (1990 to 2019)		
		1990			2019					
		Both	Female	Male	Both	Female	Male	Both	Female	Male
Syrian Arab Republic	Incidence	220.3 (204.4 to 238.9)	227.4 (209.1 to 248.1)	214.1 (197.1 to 233.9)	179.3 (164 to 196.4)	187.8 (170.4 to 208.4)	174 (158.4 to 192.6)	-18.6 (-22.5 to -14.7)	-17.4 (-23.5 to -11.2)	-18.7 (-23.7 to -13.3)
	Prevalence	1763.3 (1639.3 to 1893.7)	1873.4 (1735.8 to 2024.9)	1664.2 (1542.6 to 1805.3)	1518.7 (1403.8 to 1637.5)	1621.3 (1490.9 to 1762.6)	1425.9 (1313 to 1542.2)	-13.9 (-17.3 to -10.5)	-13.5 (-18.3 to -8.5)	-14.3 (-18.8 to -9.2)
	Deaths	146 (120.2 to 176.6)	154.6 (124.4 to 188.1)	138.6 (110.2 to 173.1)	99 (78.3 to 124.9)	112.4 (90.1 to 137.7)	94.4 (73.2 to 123.1)	-32.2 (-50.2 to -8.6)	-27.3 (-46.3 to -1.3)	-31.9 (-51.3 to -4.3)
	DALYs	3375.3 (2823.7 to 3954.9)	3343.4 (2777.6 to 3977.7)	3412.6 (2788.6 to 4131)	2018.1 (1589.6 to 2547.1)	2050 (1659.7 to 2522.7)	2062.1 (1600.6 to 2662.8)	-40.2 (-55.3 to -19.9)	-38.7 (-53.2 to -18.5)	-39.6 (-55.6 to -16.7)
	YLLs	3104 (2569.3 to 3700.7)	3033.6 (2450.6 to 3676.8)	3176.2 (2542.4 to 3889.2)	1784 (1385.4 to 2304.3)	1781.8 (1391.9 to 2254.3)	1860 (1415 to 2463)	-42.5 (-58.2 to -20.6)	-41.3 (-57 to -19.3)	-41.4 (-58.4 to -16.7)
	YLDs	271.3 (197.6 to 341.4)	309.7 (227.4 to 391.6)	236.3 (172.9 to 296.7)	234.1 (172.3 to 295.5)	268.2 (198.3 to 338)	202.1 (147.5 to 255.6)	-13.7 (-17.7 to -9.6)	-13.4 (-18.8 to -7.3)	-14.5 (-19.9 to -8)
Tunisia	Incidence	161.6 (146.8 to 177.5)	170.9 (153.7 to 189.4)	152.8 (138.2 to 168.8)	159.6 (144 to 178.9)	169.1 (150.9 to 190.1)	149.3 (133.9 to 169.2)	-1.2 (-6.6 to 4.3)	-1.1 (-8.1 to 6)	-2.3 (-9.2 to 6.2)
	Prevalence	1032.7 (941.1 to 1123.3)	1121 (1021.5 to 1220.6)	946.9 (850.7 to 1041.4)	1260.8 (1162.6 to 1366)	1368.1 (1250.5 to 1484.4)	1145.6 (1040.7 to 1258.2)	22.1 (16.3 to 28.2)	22 (14.2 to 29.8)	21 (13.5 to 29.2)
	Deaths	106.6 (88.9 to 126.5)	104.6 (84.8 to 127.5)	109.2 (88 to 132.1)	80 (60.6 to 101.4)	75.3 (56.3 to 95.4)	85.2 (62.2 to 111.9)	-25 (-43.8 to -0.6)	-27.9 (-46.3 to -3.8)	-21.9 (-45.5 to 5.9)
	DALYs	1961.4 (1663.1 to 2298.3)	1961.8 (1651.8 to 2354.9)	1964.8 (1570.2 to 2368.6)	1477.5 (1138.5 to 1875)	1418.7 (1102.8 to 1764.5)	1541.2 (1150.5 to 2009.7)	-24.7 (-42.8 to -2)	-27.7 (-44.7 to -5.7)	-21.6 (-43.5 to 5.8)
	YLLs	1796.9 (1502.7 to 2136.4)	1771.3 (1457.6 to 2158.4)	1825.2 (1434.4 to 2218.2)	1278.1 (957.8 to 1669)	1188.1 (897.8 to 1535.8)	1375.1 (989.2 to 1848.3)	-28.9 (-48 to -4.6)	-32.9 (-50.9 to -9.1)	-24.7 (-47.4 to 5.4)
	YLDs	164.5 (121.8 to 205.4)	190.6 (140.2 to 239.2)	139.6 (100.7 to 176.5)	199.4 (146.5 to 250.1)	230.6 (169.1 to 291.7)	166.1 (120.6 to 208.6)	21.2 (14.7 to 27.8)	21 (12.1 to 30.1)	19 (9.9 to 29.1)

Country	Measure	Age-standardized rate (per 100,000)						% Change (1990 to 2019)		
		1990			2019					
		Both	Female	Male	Both	Female	Male	Both	Female	Male
Turkey	Incidence	149.7 (137.7 to 163.2)	167.5 (153.2 to 182.7)	132 (120.9 to 144.4)	145.6 (133.4 to 160.7)	149.5 (136.4 to 165.5)	141 (128 to 157)	-2.8 (-7.6 to 1.9)	-10.7 (-16.3 to -4.2)	6.8 (-0.4 to 14.8)
	Prevalence	1328.5 (1232.1 to 1434.1)	1560.9 (1444.2 to 1685.1)	1079 (992.8 to 1178.4)	1213.6 (1124.7 to 1309.4)	1324.4 (1225.4 to 1436.5)	1091.9 (1004.3 to 1184.7)	-8.7 (-12.4 to -4.6)	-15.2 (-19.9 to -10.1)	1.2 (-4.6 to 7.4)
	Deaths	72.6 (58.2 to 96.1)	71.9 (54.4 to 90.8)	72.9 (58.3 to 106.1)	60.6 (48.7 to 73.6)	60.1 (47.3 to 73.7)	60.6 (48.9 to 73.8)	-16.5 (-39.5 to 6.2)	-16.4 (-39.8 to 9.8)	-16.9 (-46.2 to 13.5)
	DALYs	1520.5 (1299.1 to 1911)	1513.1 (1248.9 to 1836)	1520.5 (1242.1 to 2299.8)	1162.6 (965 to 1380.4)	1131.7 (939.7 to 1340.6)	1186.5 (968.8 to 1425.8)	-23.5 (-42.5 to -5.2)	-25.2 (-42 to -7.1)	-22 (-47.6 to 4.7)
	YLLs	1311.2 (1103.9 to 1713.3)	1255.2 (993.5 to 1567.4)	1364.1 (1090.4 to 2137.3)	971.3 (778.9 to 1182.1)	910.9 (724.6 to 1122.1)	1028.1 (814.4 to 1264.9)	-25.9 (-46.7 to -4.8)	-27.4 (-47.6 to -5.3)	-24.6 (-51.6 to 5.5)
	YLDs	209.2 (152.9 to 261.7)	257.9 (188.7 to 322.7)	156.4 (114.2 to 200)	191.2 (139.3 to 239.6)	220.9 (160 to 277.3)	158.4 (117 to 199.8)	-8.6 (-13.2 to -3.6)	-14.4 (-20.1 to -8)	1.3 (-6.5 to 8.7)
United Arab Emirates	Incidence	307.6 (277.8 to 340.6)	332.4 (297.7 to 372.3)	292 (261.1 to 324.5)	262.2 (233.8 to 294.3)	287.5 (257.2 to 324.2)	250.5 (221 to 281.9)	-14.8 (-19.2 to -9.9)	-13.5 (-20.5 to -6.1)	-14.2 (-20.6 to -8)
	Prevalence	2405.6 (2215 to 2643.8)	2711.8 (2489.9 to 2988.1)	2243 (2047.8 to 2486.8)	2225.7 (2037.3 to 2436.3)	2563.5 (2362.4 to 2805)	2096.7 (1909 to 2309.9)	-7.5 (-11.6 to -3.6)	-5.5 (-10.6 to -0.1)	-6.5 (-12 to -1.1)
	Deaths	182.5 (145.5 to 246.2)	195.6 (152 to 260.3)	174.2 (128.4 to 243.6)	91.3 (70.2 to 118.7)	89.1 (68.8 to 118)	92.4 (69 to 119.9)	-50 (-62 to -34.9)	-54.5 (-66 to -37)	-46.9 (-61.1 to -26.4)
	DALYs	3565.6 (2877.6 to 4746.3)	3825.2 (3034.8 to 4985.8)	3446.6 (2583.8 to 4747.3)	1925.6 (1517 to 2457.9)	1955.3 (1575.1 to 2440.1)	1920.6 (1479 to 2455.9)	-46 (-58.5 to -30.5)	-48.9 (-61.1 to -31.7)	-44.3 (-59.1 to -23.5)
	YLLs	3191.1 (2499.9 to 4322.8)	3368.8 (2590.5 to 4527.3)	3119.5 (2249 to 4437.6)	1589.3 (1200 to 2106.9)	1530.2 (1168.6 to 2022.2)	1619.5 (1182.6 to 2163)	-50.2 (-63.3 to -33.3)	-54.6 (-66.7 to -35.8)	-48.1 (-63.1 to -25.1)
	YLDs	374.4 (275.4 to 475.6)	456.4 (334.5 to 580.6)	327.1 (238.7 to 414.4)	336.4 (245.1 to 425.6)	425.1 (312.2 to 537)	301.1 (217.3 to 383.9)	-10.2 (-14.8 to -5.7)	-6.9 (-13 to -0.6)	-7.9 (-14.2 to -1.6)

Country	Measure	Age-standardized rate (per 100,000)						% Change (1990 to 2019)		
		1990			2019					
		Both	Female	Male	Both	Female	Male	Both	Female	Male
Yemen	Incidence	229.7 (212 to 249.6)	236.3 (216.7 to 259)	223 (204.7 to 243.6)	207.9 (190.1 to 228.3)	219.1 (198.4 to 241.9)	196.4 (178.4 to 218.4)	-9.5 (-14 to -4.7)	-7.3 (-13.4 to -0.8)	-11.9 (-17.7 to -5.6)
	Prevalence	1596.2 (1482.1 to 1716.9)	1715.1 (1595.3 to 1852)	1455.7 (1340.2 to 1590.7)	1609.4 (1480.2 to 1745.5)	1783.3 (1632 to 1944.4)	1430.6 (1307.3 to 1558)	0.8 (-4.2 to 5.7)	4 (-2.3 to 11.1)	-1.7 (-7.8 to 4.3)
	Deaths	174.2 (121.5 to 227.9)	172.3 (115.6 to 227)	177.7 (123.9 to 232.4)	135.7 (102.4 to 176.6)	138.4 (103.4 to 179.8)	132.8 (97.8 to 176.6)	-22.1 (-38.2 to 0.7)	-19.7 (-36.3 to 7.5)	-25.3 (-41.3 to -2.4)
	DALYs	3663.8 (2666.7 to 4746.9)	3689.1 (2612.8 to 4814.4)	3651.9 (2664.7 to 4784.8)	2765.5 (2161.6 to 3520.5)	2860.3 (2209 to 3665.2)	2667.9 (2012.3 to 3583.2)	-24.5 (-41 to -1.9)	-22.5 (-38.9 to 3.5)	-26.9 (-44 to -2.4)
	YLLs	3417.1 (2433.1 to 4490.4)	3408.6 (2334.9 to 4528.7)	3444.7 (2472.5 to 4563.6)	2515.8 (1917 to 3278.3)	2566.9 (1911.7 to 3379)	2463.3 (1816.6 to 3352.6)	-26.4 (-43.7 to -2.2)	-24.7 (-42 to 3.4)	-28.5 (-46.2 to -2.5)
	YLDs	246.7 (179.6 to 310.9)	280.4 (206 to 355.2)	207.3 (150.2 to 263)	249.6 (183.1 to 315.4)	293.4 (213.4 to 371.2)	204.6 (149.7 to 257)	1.2 (-4.2 to 7)	4.6 (-2.1 to 12.4)	-1.3 (-8.2 to 6)

Data in parentheses are 95% Uncertainty Intervals (95% UIs)

DALYs=Disability-Adjusted Life Years; YLLs=Years of Life Lost; YLDs=Years Lived with Disability
