# Supplementary material for: Burden of stroke in North Africa and Middle East, 1990 to 2019: a systematic analysis for the global burden of disease study 2019
Source: BMC Neurol. 2022 Jul 27;22:279. doi: 10.1186/s12883-022-02793-0 (PMC9327376; doi:10.1186/s12883-022-02793-0)
Supplement: Supplementary file 4 — Additional file 4: Supplementary Table 3. Age-standardized attributed burden to stroke risk factors in 1990 and 2019 at the super-region and its 21 countries [file 12883_2022_2793_MOESM4_ESM.pdf]

Location	Risk factor	Measure	1990			2019			% Change (1990 to 2019)		
			Both	Female	Male	Both	Female	Male	Both	Female	Male
North Africa and Middle East	All risk factors	Deaths	98.9 (83.4 to 112.4)	100 (82.8 to 115.1)	97.3 (83.7 to 119.8)	74.3 (65.5 to 84.4)	76.6 (66.5 to 87)	72 (63.2 to 83.1)	-24.9 (-33.5 to -11.6)	-23.4 (-32.5 to -7.3)	-26.1 (-37.2 to -13.2)
		DALYs	2112.9 (1846.3 to 2382.6)	2148.6 (1852.9 to 2418.8)	2072.4 (1804.7 to 2486)	1566.9 (1393.2 to 1757.1)	1602.7 (1419.3 to 1798.2)	1530.7 (1345 to 1746.4)	-25.8 (-34.1 to -14)	-25.4 (-33.9 to -12.3)	-26.1 (-36.8 to -13.5)
		YLLs	1909.3 (1647.4 to 2174.2)	1905.8 (1625.1 to 2166.3)	1907.7 (1648.8 to 2343.1)	1359.2 (1198.9 to 1548.9)	1358 (1182.6 to 1543.5)	1358.4 (1179.9 to 1577.9)	-28.8 (-37.9 to -15.8)	-28.7 (-37.8 to -14.4)	-28.8 (-39.7 to -15.5)
		YLDs	203.6 (148.4 to 259)	242.9 (177.3 to 307.8)	164.7 (119.8 to 211.3)	207.7 (152.1 to 263.7)	244.7 (179.4 to 309.5)	172.2 (125.3 to 217.8)	2 (-0.3 to 4.4)	0.8 (-2 to 3.8)	4.5 (1.6 to 7.6)
	Alcohol use	Deaths	0.5 (0.2 to 0.8)	0 (-0.1 to 0.2)	1 (0.5 to 1.4)	0.2 (0.1 to 0.4)	0 (-0.2 to 0.1)	0.5 (0.2 to 0.8)	-54.7 (-78.2 to -35.6)	-187 (-931.6 to 1078)	-50.3 (-67.4 to -33.4)
		DALYs	16.3 (9.1 to 23.4)	2.4 (-1.8 to 7)	29.7 (17 to 43.3)	7.7 (3.2 to 12.3)	-0.1 (-2.8 to 3.1)	14.9 (7.3 to 23.4)	-52.9 (-69.5 to -37.4)	-102.8 (-796.6 to 518.7)	-49.7 (-64.4 to -34.2)
		YLLs	15.4 (8.8 to 22.2)	2.5 (-1.2 to 6.6)	27.9 (16 to 40.8)	7.1 (3.2 to 11.4)	0.2 (-2 to 2.8)	13.5 (6.8 to 21.2)	-54.3 (-69.4 to -39.1)	-91.7 (-510 to 313.3)	-51.6 (-65.5 to -36.3)
		YLDs	0.8 (0.2 to 1.4)	-0.2 (-0.7 to 0.5)	1.8 (0.8 to 2.9)	0.6 (0 to 1.2)	-0.3 (-0.8 to 0.3)	1.4 (0.4 to 2.5)	-27.5 (-90.9 to 3.1)	70.3 (-621 to 646.5)	-20 (-50.1 to -1.7)
	Ambient particulate matter pollution	Deaths	18.8 (14.6 to 24)	17.8 (13.7 to 22.7)	19.7 (15.3 to 25.2)	19.3 (16.1 to 22.8)	19.1 (15.9 to 22.7)	19.4 (16 to 23.1)	2.4 (-15.9 to 26.2)	7 (-13.2 to 35)	-1.8 (-21 to 21.3)
		DALYs	453.5 (356.5 to 572)	431.8 (333.8 to 545.6)	474 (366.6 to 605.5)	466.4 (391.1 to 553.9)	461.6 (382.5 to 549.4)	470.5 (390.7 to 563.6)	2.8 (-14.7 to 26.1)	6.9 (-13 to 33.7)	-0.7 (-19 to 22.9)
		YLLs	404.6 (315.6 to 511)	375.8 (288.1 to 481.5)	432 (334.6 to 556.2)	399.1 (331.1 to 477.7)	383.4 (315.9 to 458.2)	413.6 (339.1 to 502.7)	-1.3 (-19.7 to 22.8)	2 (-18 to 29.2)	-4.3 (-23.2 to 20.2)
		YLDs	48.9 (33.6 to 66.1)	56 (38.3 to 75.9)	41.9 (28.6 to 57.2)	67.3 (47.1 to 86.3)	78.2 (54.8 to 100.9)	56.9 (39.9 to 72.9)	37.6 (22.3 to 61)	39.5 (23.4 to 66.5)	35.8 (20.6 to 58.3)
	Diet high in red meat	Deaths	3.5 (1.4 to 5.5)	3.5 (1.4 to 5.6)	3.5 (1.4 to 5.4)	2.4 (0.9 to 3.8)	2.5 (0.9 to 3.9)	2.4 (0.9 to 3.8)	-31.4 (-49.9 to -11.3)	-30.7 (-51.7 to -8)	-32 (-48.9 to -13)
		DALYs	95.6 (37.1 to 145.9)	97.8 (37.7 to 149.6)	93.4 (35.9 to 144.1)	66 (25.9 to 102.1)	67.2 (26.5 to 104.8)	64.9 (25 to 100.7)	-30.9 (-48 to -10)	-31.3 (-50.6 to -9)	-30.5 (-46.6 to -10.9)
		YLLs	85.5 (32.2 to 131.1)	85.7 (32.5 to 131.8)	85.2 (32.2 to 132.4)	56 (22 to 88.1)	55.4 (22.3 to 88.4)	56.5 (22.3 to 88.3)	-34.5 (-50.7 to -13.1)	-35.4 (-53.9 to -11.5)	-33.7 (-48.9 to -13.4)
		YLDs	10.1 (3.5 to 16.6)	12.1 (4.1 to 19.9)	8.2 (2.9 to 13.4)	10 (3.1 to 16.6)	11.8 (3.7 to 19.6)	8.4 (2.7 to 13.8)	-1 (-13.8 to 6.6)	-2.4 (-16.8 to 6)	1.6 (-10.8 to 9.2)
	Diet high in sodium	Deaths	1.9 (0.4 to 8)	1.4 (0.4 to 5.6)	2.5 (0.3 to 10.3)	1.4 (0.3 to 5.4)	1 (0.3 to 3.8)	1.8 (0.2 to 7.1)	-29.4 (-42 to -7.5)	-30.1 (-46.5 to -4.6)	-29.7 (-43.1 to 2.8)
		DALYs	47.2 (7.8 to 192.7)	33.3 (9.3 to 134.4)	60.8 (5.4 to 240.1)	33.6 (5.8 to 133.3)	23.2 (6.9 to 94.8)	43.6 (4.2 to 169.4)	-28.8 (-40.4 to -8.7)	-30.5 (-45.7 to -6.8)	-28.4 (-40.3 to 1.2)
		YLLs	42.9 (6.9 to 177.3)	29.6 (8.1 to 120.6)	55.9 (5 to 223)	29.2 (5 to 116.1)	19.5 (5.8 to 79.9)	38.5 (3.7 to 149)	-31.8 (-44 to -11.2)	-34 (-49.4 to -10.1)	-31.1 (-43.3 to -1.4)
		YLDs	4.3 (0.7 to 17.3)	3.8 (1 to 15.1)	4.9 (0.4 to 19.2)	4.4 (0.7 to 17.1)	3.6 (1 to 14.7)	5.1 (0.4 to 19.5)	0.8 (-14.6 to 21.9)	-3.2 (-24 to 23.5)	3.5 (-9.7 to 39.4)
	Diet low in fiber	Deaths	2.4 (0.6 to 4.5)	2.4 (0.6 to 4.5)	2.4 (0.6 to 4.5)	1.5 (0.4 to 2.7)	1.5 (0.4 to 2.8)	1.4 (0.4 to 2.6)	-38.8 (-46.4 to -27.2)	-36.7 (-45.7 to -22)	-40.8 (-48.5 to -29)
		DALYs	56.7 (13 to 107.3)	58.4 (13.3 to 111)	55.1 (12.9 to 105.1)	36.2 (9.2 to 66.7)	38.1 (9.8 to 70)	34.5 (8.7 to 64.3)	-36.1 (-44 to -23.8)	-34.6 (-44.2 to -19.2)	-37.4 (-45.5 to -24.7)
		YLLs	51.4 (11.9 to 98.5)	52.2 (12 to 99.2)	50.7 (11.8 to 97.2)	31.7 (8 to 58.5)	32.7 (8.3 to 60.1)	30.7 (7.7 to 56.9)	-38.4 (-46.7 to -25.3)	-37.3 (-47.3 to -20.8)	-39.5 (-47.8 to -26.3)
		YLDs	5.3 (1.2 to 10.4)	6.2 (1.4 to 12.1)	4.4 (1 to 8.6)	4.6 (1.1 to 8.9)	5.4 (1.3 to 10.6)	3.8 (0.9 to 7.4)	-13.1 (-16.8 to -5.6)	-12.2 (-17.4 to -2.8)	-13.4 (-18.8 to -4.9)
	Diet low in fruits	Deaths	5.1 (2.7 to 8.3)	5.3 (2.8 to 8.6)	4.9 (2.6 to 8)	2.6 (1.3 to 4.2)	2.7 (1.3 to 4.4)	2.5 (1.2 to 4)	-49.5 (-60.6 to -37.3)	-48.9 (-60.7 to -34)	-49.9 (-60.7 to -37.6)
		DALYs	132.9 (70.6 to 216)	141.1 (74.8 to 227.7)	124.9 (65.8 to 203.9)	70.5 (35.4 to 112.5)	74.7 (37.3 to 119)	66.5 (33.8 to 109.2)	-47 (-58.1 to -34.3)	-47.1 (-59.2 to -32.8)	-46.7 (-57.6 to -34.7)
		YLLs	121.1 (65.8 to 197.3)	126.7 (68.4 to 204.9)	115.7 (61.3 to 189.5)	61.5 (31.8 to 98.6)	63.8 (32.3 to 101.3)	59.4 (30.6 to 97.1)	-49.2 (-59.9 to -36.1)	-49.7 (-61.2 to -34.8)	-48.7 (-59.3 to -35.9)
		YLDs	11.8 (4.8 to 20.7)	14.4 (5.9 to 25.2)	9.2 (3.8 to 16.4)	9 (3.6 to 15.6)	10.9 (4.3 to 19.1)	7.2 (2.9 to 12.6)	-23.8 (-30.8 to -17.7)	-24.1 (-32.3 to -17.1)	-22.6 (-29.5 to -16)
	Diet low in vegetables	Deaths	2.7 (1.1 to 4.4)	2.7 (1.1 to 4.4)	2.7 (1.1 to 4.5)	1.2 (0.5 to 2)	1.3 (0.6 to 2.1)	1.2 (0.5 to 2)	-53.2 (-63.5 to -38.7)	-51.8 (-62.8 to -35)	-54.6 (-64.5 to -40.8)
		DALYs	64.2 (23.9 to 107.8)	66.5 (25.8 to 112.3)	62.1 (24.1 to 106.6)	31.1 (13.4 to 50.9)	32.8 (14.6 to 54.4)	29.5 (12.7 to 48.9)	-51.6 (-61.6 to -36.8)	-50.6 (-61.2 to -33.9)	-52.4 (-62.2 to -38.1)
		YLLs	59.3 (21.7 to 100.9)	60.7 (23.4 to 103.6)	57.9 (21.6 to 100.8)	27.6 (11.9 to 44.9)	28.8 (12.8 to 47.2)	26.6 (11.2 to 44.9)	-53.4 (-63.1 to -38.3)	-52.5 (-63 to -35.3)	-54.1 (-63.7 to -39.8)
		YLDs	5 (1.9 to 8.7)	5.8 (2.2 to 10)	4.2 (1.5 to 7.3)	3.5 (1.3 to 6.1)	4 (1.5 to 7)	3 (1.1 to 5.2)	-30.2 (-37.3 to -21)	-30.8 (-38.2 to -20.8)	-28.8 (-36.3 to -19.6)
	Diet low in whole grains	Deaths	5.1 (2.7 to 6.9)	5.2 (2.9 to 7)	5 (2.6 to 6.9)	4.6 (2.5 to 6.1)	4.9 (2.6 to 6.4)	4.4 (2.3 to 6)	-9.2 (-21.3 to 3.9)	-6.3 (-17.4 to 12.8)	-11.4 (-28.9 to 2)
		DALYs	105 (52.1 to 141.3)	107.6 (55.4 to 145.6)	102 (49.4 to 141)	96.7 (46.5 to 131.4)	101.6 (49.5 to 136.3)	91.9 (43.3 to 126.3)	-8 (-19.2 to 3.7)	-5.6 (-16.3 to 8.6)	-9.9 (-26.9 to 3.1)
		YLLs	88.2 (44 to 120)	87.4 (45.3 to 118.5)	88.6 (42.8 to 122.7)	79.3 (38.8 to 107.1)	80.8 (40.9 to 109.5)	77.8 (37.3 to 107.8)	-10.1 (-23.1 to 3.5)	-7.5 (-20 to 10.3)	-12.2 (-30.5 to 2.6)
		YLDs	16.8 (7.8 to 25.1)	20.3 (9.5 to 30.1)	13.4 (6.1 to 20.2)	17.3 (7.7 to 25.9)	20.8 (9.4 to 30.8)	14.1 (6.2 to 21)	3.1 (-0.3 to 5.8)	2.4 (-1.3 to 5.7)	5 (0.9 to 8.3)
	High body-mass index	Deaths	21 (12.8 to 30.1)	24.6 (15.9 to 34.4)	17.5 (9.7 to 26.5)	20 (13.3 to 28.2)	21.5 (14.7 to 30.1)	18.5 (11.8 to 26.6)	-5.1 (-21.8 to 19.4)	-12.7 (-27.7 to 7.9)	5.6 (-17 to 42.8)
		DALYs	658.8 (430.2 to 907)	777.1 (537.9 to 1035.1)	544.5 (320.9 to 790.2)	618 (440.1 to 822.7)	664.6 (484.5 to 874.4)	573.2 (389.4 to 777.6)	-6.2 (-21.8 to 16.6)	-14.5 (-28.3 to 2.6)	5.3 (-17 to 41.3)
		YLLs	588.2 (380 to 811.4)	681.4 (468 to 916.3)	498.1 (292.7 to 728.7)	525.8 (369.6 to 701.4)	549.3 (394 to 725.5)	502.7 (339.3 to 689.8)	-10.6 (-26.8 to 12.4)	-19.4 (-33.5 to -1)	0.9 (-20.9 to 37.6)
		YLDs	70.5 (42.5 to 105.3)	95.7 (60.3 to 138.7)	46.4 (25.5 to 73.6)	92.2 (60.2 to 130)	115.3 (76.6 to 160.1)	70.5 (44.3 to 102.1)	30.8 (19.5 to 49.8)	20.5 (11.5 to 34.7)	52 (32.1 to 90.9)
	High fasting plasma glucose	Deaths	19.6 (11.9 to 34.6)	20.4 (12 to 37.9)	18.6 (11.5 to 30.4)	25.5 (14.8 to 45.2)	26.5 (15.2 to 48.1)	24.5 (14.1 to 42.7)	30.3 (2.9 to 59.5)	30.1 (0.9 to 64)	31.6 (2.7 to 62.7)
		DALYs	374.5 (248.6 to 576.4)	389.2 (249.9 to 613.2)	358.1 (241.8 to 542.7)	504.9 (318.8 to 793.9)	524 (331.6 to 820.2)	485.7 (302 to 760.7)	34.8 (7.9 to 64.8)	34.6 (6.6 to 72.3)	35.6 (7.5 to 67.2)
		YLLs	339.2 (224.1 to 519.5)	346.7 (223.1 to 552.9)	330.2 (222.7 to 500.1)	440 (281 to 703.7)	447.3 (283.1 to 707.6)	432.4 (271.4 to 687.1)	29.7 (2.1 to 59.3)	29 (1 to 67.3)	31 (3.2 to 62.9)
		YLDs	35.3 (20.4 to 59.1)	42.5 (24.1 to 71.9)	28 (16.3 to 45.9)	64.9 (35.8 to 106.8)	76.7 (42.8 to 125.9)	53.4 (29.2 to 86.1)	83.9 (61.9 to 112.3)	80.5 (56.2 to 110.8)	90.9 (65 to 120.5)
	High LDL cholesterol	Deaths	12.8 (4.7 to 27.2)	13.4 (4.8 to 29.1)	12 (4.7 to 24.5)	12.2 (4.7 to 25.1)	13.2 (4.9 to 27.2)	11.2 (4.5 to 22.3)	-4.7 (-18.3 to 10.5)	-1.7 (-14.6 to 18.8)	-6.8 (-25 to 9.6)
		DALYs	277.1 (159.3 to 469.3)	293.4 (166.4 to 505.5)	259.6 (150.5 to 425.8)	274.7 (160.6 to 448.1)	297.1 (172.7 to 492.4)	253 (150.5 to 410.9)	-0.9 (-14 to 13.7)	1.2 (-10.7 to 18.8)	-2.5 (-20.5 to 13.8)
		YLLs	229 (124.2 to 398.7)	233.7 (124.1 to 423.1)	222.8 (125 to 374.1)	222 (126.5 to 376.2)	232.3 (127.5 to 404.6)	211.6 (122.5 to 349.4)	-3.1 (-18.6 to 14.5)	-0.6 (-15.8 to 21.9)	-5 (-24.8 to 14.2)
		YLDs	48.1 (28.9 to 78.1)	59.7 (36.4 to 96.9)	36.8 (21.7 to 59.6)	52.7 (32.1 to 84.9)	64.7 (39.5 to 105.4)	41.3 (25.1 to 66.5)	9.5 (6.7 to 12.8)	8.5 (5.4 to 12.1)	12.4 (8.3 to 16.9)
	High systolic [illegible]	Deaths	58.7 (46.7 to 71.7)	62 (48.5 to 77.1)	54.9 (43.4 to 69)	44 (35.1 to 53.5)	46.7 (36.5 to 57.4)	41.2 (32.6 to 51)	-25.2 (-35.3 to -10.7)	-24.7 (-35.4 to -7.7)	-25 (-36.4 to -10.7)

Location	Risk factor	Measure	1990			2019			% Change (1990 to 2019)		
			Both	Female	Male	Both	Female	Male	Both	Female	Male
	blood pressure	DALYs	1285.3 (1054.8 to 1530.5)	1357.3 (1111.3 to 1621.8)	1209.5 (979 to 1488.9)	965 (800.6 to 1143.2)	1009 (821.8 to 1196.7)	920.4 (751 to 1103.3)	-24.9 (-34.3 to -12)	-25.7 (-35.4 to -11.1)	-23.9 (-35.1 to -9.4)
		YLLs	1161.4 (939.6 to 1395.2)	1205.1 (969.5 to 1453.9)	1113.7 (894.6 to 1388.8)	836.8 (686.5 to 998.2)	855.8 (692.9 to 1021.3)	816.5 (662.3 to 988.7)	-27.9 (-37.6 to -13.9)	-29 (-39.3 to -13.5)	-26.7 (-38.3 to -11.6)
		YLDs	123.9 (87.8 to 162.5)	152.1 (108.4 to 199.3)	95.9 (67.7 to 127)	128.2 (90.9 to 167.2)	153.3 (108.6 to 200.3)	103.9 (73 to 136)	3.5 (-0.6 to 7.9)	0.7 (-4.4 to 6.5)	8.4 (3.5 to 13.8)
	High temperature	Deaths	1.8 (0.2 to 4)	1.8 (0.2 to 4.1)	1.8 (0.2 to 4)	1.5 (0.5 to 2.9)	1.5 (0.5 to 2.9)	1.5 (0.5 to 3)	-16.8 (-52.5 to 47.8)	-17.6 (-53.6 to 50.9)	-15.9 (-53.5 to 44.8)
		YLLs	37.3 (3.8 to 86.3)	36.6 (3.7 to 84.7)	37.8 (3.9 to 87.2)	29.4 (10.2 to 59.3)	28 (9.3 to 56.5)	30.7 (10.6 to 61.6)	-21.2 (-59.9 to 33.6)	-23.7 (-58.8 to 36)	-18.9 (-57.7 to 38.5)
	Household air pollution from solid fuels	Deaths	15 (9.9 to 21.5)	16.5 (10.8 to 23.3)	13.4 (8.5 to 19.8)	2.6 (1.7 to 3.7)	3.1 (2.1 to 4.4)	2.1 (1.2 to 3.2)	-82.7 (-86 to -78.6)	-80.9 (-84.6 to -76.1)	-84.6 (-88 to -80.5)
		DALYs	350.7 (238.1 to 487.3)	394.6 (271.3 to 542.6)	307.4 (196.5 to 440.6)	66.8 (44.8 to 93.1)	83.4 (57.4 to 115.7)	51.3 (31.2 to 77.4)	-80.9 (-84.8 to -76.1)	-78.9 (-83.2 to -73.6)	-83.3 (-87 to -78.7)
		YLLs	321.5 (216.2 to 446.5)	357.2 (240.8 to 491.1)	286.1 (182.2 to 411.8)	60.6 (40.3 to 85.3)	75.1 (50.8 to 106.2)	47 (28.4 to 71.6)	-81.1 (-85.1 to -76.1)	-79 (-83.5 to -73.4)	-83.6 (-87.3 to -78.9)
		YLDs	29.3 (18.4 to 42.6)	37.4 (23.6 to 54.5)	21.3 (12.9 to 31.9)	6.2 (4 to 8.8)	8.3 (5.5 to 11.7)	4.3 (2.5 to 6.5)	-78.7 (-81.8 to -75.1)	-77.8 (-80.8 to -74.3)	-79.9 (-83.1 to -76.2)
	Kidney dysfunction	Deaths	9 (6.6 to 11.8)	9.8 (6.9 to 13.1)	8.2 (6.1 to 10.5)	9.3 (6.2 to 12.2)	9.8 (6.4 to 13.3)	8.7 (5.9 to 11.5)	3 (-15.8 to 24.8)	0.3 (-20.1 to 26)	7 (-14.4 to 27.3)
		DALYs	194.6 (155 to 238.3)	215.4 (169.5 to 268)	173.5 (137.8 to 214.2)	200.8 (155.6 to 247.9)	216.2 (164.6 to 268.5)	185.9 (143 to 230.8)	3.2 (-12.2 to 21.1)	0.4 (-15.8 to 22.1)	7.1 (-10.9 to 26.3)
		YLLs	173.5 (138.2 to 215.9)	188.4 (147.4 to 239.1)	158.4 (124.9 to 198.8)	172.1 (131.3 to 214.2)	180.7 (135.1 to 227.9)	163.6 (124.1 to 205.5)	-0.9 (-17.1 to 19)	-4.1 (-21.2 to 20.2)	3.3 (-15 to 23.8)
		YLDs	21 (14.3 to 28.1)	27 (18.4 to 36.1)	15.1 (10.2 to 20.2)	28.8 (19.6 to 38.8)	35.5 (24.3 to 47.9)	22.3 (15 to 30)	36.9 (29.3 to 43.8)	31.5 (24.5 to 38)	47.2 (37.2 to 56.3)
	Lead exposure	Deaths	6.9 (4.4 to 9.8)	5.8 (3.4 to 8.4)	8 (5.3 to 11.4)	4.6 (2.9 to 6.7)	4 (2.2 to 6)	5.3 (3.4 to 7.4)	-33 (-44 to -20.6)	-31.7 (-43.3 to -16.4)	-34.1 (-44.8 to -22.7)
		DALYs	161.3 (103.9 to 224.5)	135.1 (79 to 195.5)	186.8 (126.2 to 261.8)	95.5 (57.8 to 137.7)	81.3 (45.3 to 122.9)	109.3 (69.3 to 153.2)	-40.8 (-50.5 to -30.4)	-39.8 (-50.4 to -27.9)	-41.5 (-50.9 to -30.7)
		YLLs	147.5 (94.7 to 207.2)	121.5 (72 to 177.2)	173 (115.7 to 243.7)	84.7 (51.7 to 121.2)	70.7 (39.6 to 106.7)	98.2 (62.3 to 138.7)	-42.6 (-52.6 to -31.5)	-41.8 (-52.4 to -28.5)	-43.3 (-52.7 to -31.9)
		YLDs	13.7 (7.8 to 21)	13.6 (7.2 to 21.8)	13.8 (8.4 to 20.5)	10.9 (5.9 to 17.1)	10.6 (5.2 to 17.5)	11.1 (6.3 to 17)	-20.8 (-29 to -15)	-22.2 (-30.8 to -16)	-19.5 (-27.6 to -13.6)
	Low physical activity	Deaths	5.8 (1.3 to 12.2)	6 (1.5 to 12.2)	5.6 (1.1 to 12)	5.6 (1.3 to 11.6)	6 (1.5 to 12.1)	5.2 (1 to 10.9)	-3.2 (-15.8 to 12.7)	0 (-13 to 20.1)	-5.9 (-22.5 to 9.5)
		DALYs	90.6 (19.1 to 204.6)	95.2 (22.1 to 208)	85.6 (16.2 to 198)	91.7 (18.8 to 203.4)	98.9 (22.9 to 211.8)	84.7 (15.5 to 189.5)	1.2 (-11.8 to 16)	3.9 (-10.1 to 22.1)	-1.1 (-18.1 to 15.4)
		YLLs	78.6 (16.8 to 175.9)	80.6 (18.7 to 174.2)	76.3 (14.6 to 175.7)	77.7 (15.8 to 170.3)	81.9 (19.5 to 174.9)	73.6 (13.6 to 164.5)	-1.1 (-15.8 to 15.5)	1.6 (-13.4 to 22.5)	-3.5 (-22.2 to 14.3)
		YLDs	12 (2.2 to 29.3)	14.6 (3 to 35.4)	9.3 (1.6 to 23.8)	14 (2.7 to 33.6)	17 (3.5 to 39.8)	11 (1.9 to 27.6)	16.6 (7.3 to 29.1)	16.6 (7.3 to 30)	18.3 (7.6 to 33)
	Low temperature	Deaths	8 (5.1 to 11.1)	8.1 (5.1 to 11.3)	7.9 (5.1 to 11)	5.9 (3.8 to 8)	6.2 (4.1 to 8.4)	5.6 (3.6 to 7.7)	-26.5 (-36.4 to -12.8)	-23.5 (-34 to -6.6)	-29.2 (-41.8 to -16.7)
		YLLs	153.7 (97.3 to 216.4)	152.4 (94.8 to 216.7)	154.4 (95.8 to 218.7)	100.9 (62 to 140.7)	102.5 (64.2 to 142.5)	99.1 (59.7 to 141.9)	-34.4 (-43.8 to -23.6)	-32.8 (-42.2 to -20.5)	-35.8 (-46.8 to -24.2)
	Secondhand smoke	Deaths	4.6 (3.3 to 5.9)	5.3 (3.9 to 6.9)	3.8 (2.8 to 5)	3 (2.2 to 3.8)	3.5 (2.6 to 4.4)	2.5 (1.8 to 3.2)	-35.7 (-43.9 to -24.5)	-35.4 (-43.9 to -22.1)	-35.5 (-45.6 to -24.3)
		DALYs	111.9 (82.2 to 142.9)	137.2 (100.8 to 175.5)	87.4 (62.2 to 113.3)	71.9 (53.5 to 91.9)	88 (64.9 to 113)	57 (41.2 to 74.5)	-35.7 (-43.7 to -24.8)	-35.9 (-44.9 to -23.8)	-34.8 (-44.5 to -23.2)
		YLLs	104.5 (76.1 to 134.5)	126.5 (92.8 to 162.5)	83.1 (58.8 to 108.3)	65.3 (48.3 to 83.3)	78.5 (57.2 to 101.4)	52.9 (38.1 to 69.1)	-37.5 (-46 to -26.2)	-37.9 (-47.2 to -25.1)	-36.3 (-46.2 to -24.3)
		YLDs	7.4 (4.8 to 10.3)	10.7 (6.9 to 14.8)	4.3 (2.8 to 6)	6.7 (4.3 to 9.2)	9.5 (6.1 to 13)	4 (2.6 to 5.6)	-10.4 (-13.1 to -7.7)	-11.2 (-14.3 to -8.1)	-6.3 (-10.7 to -1.6)
	Smoking	Deaths	14 (12.2 to 16.9)	5.2 (4.2 to 6.3)	23 (19.8 to 28.6)	9 (7.8 to 10.3)	3.1 (2.6 to 3.7)	14.7 (12.6 to 17)	-35.8 (-45.8 to -23.9)	-39.5 (-50.4 to -26.5)	-36.1 (-46.6 to -23.7)
		DALYs	377.9 (329.6 to 455)	151.6 (125.2 to 181.7)	599.2 (514.8 to 729.6)	246 (214.2 to 280.1)	91.3 (76.5 to 107.4)	393.2 (336.6 to 452.6)	-34.9 (-44.3 to -23.8)	-39.8 (-49.1 to -29.1)	-34.4 (-44.8 to -22.4)
		YLLs	340.2 (293.7 to 414)	129.6 (106.2 to 157.2)	546.4 (464.7 to 673.5)	212.6 (182.7 to 246.2)	74.1 (61.9 to 88)	344.6 (291.7 to 402.6)	-37.5 (-47.3 to -25.3)	-42.9 (-52.9 to -30.9)	-36.9 (-47.6 to -23.9)
		YLDs	37.6 (27.4 to 47.7)	22 (15.4 to 28.4)	52.8 (38.5 to 67.9)	33.4 (24.1 to 42.3)	17.2 (12.3 to 22.5)	48.6 (34.9 to 62.2)	-11.3 (-15.1 to -7.5)	-21.6 (-29.7 to -13)	-7.9 (-11.4 to -4.2)
Afghanistan	All risk factors	Deaths	171.7 (117.7 to 222.3)	193.1 (122.4 to 254.5)	150.8 (103.6 to 201.9)	141.8 (97.3 to 183.1)	163.1 (104.7 to 217.4)	119.7 (84.3 to 158.7)	-17.4 (-35.5 to 0.5)	-15.5 (-36.1 to 7.2)	-20.6 (-38.5 to -0.2)
		DALYs	3918.2 (2798.8 to 5039.1)	4546.8 (3139.6 to 5924.1)	3258.5 (2322.1 to 4396.6)	3136.6 (2253.2 to 4043.7)	3687.1 (2565.8 to 4876)	2567.2 (1870.1 to 3339.9)	-19.9 (-38.2 to -0.9)	-18.9 (-39 to 4.4)	-21.2 (-39.2 to 1)
		YLLs	3711.5 (2596.8 to 4842.1)	4293.9 (2877.2 to 5644.1)	3097.7 (2156.2 to 4225.7)	2919.3 (2036.9 to 3811.3)	3426.5 (2283 to 4642)	2395.1 (1694.6 to 3170.7)	-21.3 (-40.8 to -1.1)	-20.2 (-40.8 to 4.6)	-22.7 (-41.8 to 0.6)
		YLDs	206.7 (151.6 to 261.7)	252.9 (184.3 to 318.3)	160.9 (117.3 to 206.3)	217.3 (159.6 to 274.4)	260.6 (191.5 to 330.3)	172 (123.9 to 218.2)	5.2 (-0.7 to 11.2)	3 (-3.9 to 10.8)	6.9 (-1.6 to 15.2)
	Alcohol use	Deaths	0 (0 to 0)	0 (0 to 0)	0 (0 to 0)	0 (-0.1 to 0.1)	-0.1 (-0.1 to 0)	0.1 (-0.1 to 0.3)	-	-	-
		DALYs	0 (-0.1 to 0.1)	0 (-0.1 to 0)	0 (-0.2 to 0.2)	0.9 (-2.1 to 4.5)	-1 (-2.7 to 0.7)	2.8 (-2.4 to 10)	-	-	-
		YLLs	0 (-0.1 to 0.1)	0 (-0.1 to 0)	0 (-0.1 to 0.2)	0.9 (-1.8 to 4.3)	-0.8 (-2.4 to 0.8)	2.8 (-2.1 to 9.6)	-	-	-
		YLDs	0 (0 to 0)	0 (0 to 0)	0 (0 to 0)	-0.1 (-0.3 to 0.2)	-0.1 (-0.3 to 0)	0 (-0.4 to 0.5)	-	-	-
	Ambient particulate matter pollution	Deaths	6.8 (1.6 to 18.4)	6.4 (1.4 to 17.7)	7.2 (1.7 to 19.3)	14.4 (6.3 to 28)	14.6 (6 to 29.1)	14.2 (6 to 27.2)	110.7 (13.9 to 416.7)	128.2 (23.7 to 471.1)	98.7 (8.6 to 382.1)
		DALYs	164.7 (37.7 to 440.3)	161 (36.8 to 439.7)	165.4 (38.8 to 449.9)	350.1 (150.6 to 677.2)	363.8 (151.4 to 718.3)	337.9 (140.9 to 642.9)	112.6 (15.3 to 436.2)	126 (20.2 to 471.6)	104.2 (11.7 to 402.7)
		YLLs	155.9 (35.4 to 418.7)	151.9 (34.1 to 412)	157 (37.2 to 433.3)	325.1 (137.9 to 631.8)	337.3 (138.7 to 667.2)	314.4 (129.2 to 606.4)	108.5 (12.2 to 425.3)	122.1 (17.7 to 466.3)	100.2 (8.3 to 396.8)
		YLDs	8.8 (2.1 to 23.9)	9.1 (2.1 to 25.3)	8.4 (2 to 22.7)	25 (10.4 to 46.2)	26.5 (11 to 49.8)	23.5 (9.6 to 41.9)	184.8 (61 to 574.2)	191.7 (62.6 to 590.2)	179.8 (57.5 to 576.2)
	Diet high in red meat	Deaths	10.9 (4.5 to 17.4)	12.4 (4.9 to 20.1)	9.3 (3.8 to 15.3)	5.8 (2.1 to 9.8)	6.8 (2.5 to 11.6)	4.8 (1.7 to 8)	-47 (-66.6 to -23.8)	-45.5 (-66.5 to -19.2)	-48.7 (-67.3 to -25.7)
		DALYs	317.5 (129.6 to 500.2)	375.4 (147.9 to 606.1)	251.8 (104.2 to 416.1)	163.1 (59.8 to 276.1)	196.5 (70.9 to 337.2)	129.5 (46.4 to 222)	-48.6 (-68.2 to -24.9)	-47.6 (-68.2 to -20.7)	-48.6 (-67.5 to -25.2)
		YLLs	299.9 (120 to 478.8)	354 (135.4 to 582.7)	238.4 (96.2 to 399.7)	150.9 (54.2 to 255.8)	181.7 (65.9 to 319.9)	120.1 (42.6 to 207.8)	-49.7 (-68.8 to -25)	-48.7 (-69.4 to -21)	-49.6 (-68.1 to -25.3)
		YLDs	17.6 (7.7 to 27)	21.4 (9.5 to 33)	13.4 (5.8 to 20.6)	12.2 (4.2 to 20)	14.9 (5 to 24.4)	9.5 (3.2 to 15.6)	-30.6 (-52.6 to -16.2)	-30.5 (-53.8 to -14.6)	-29.3 (-53.4 to -14.1)
	Diet high in sodium	Deaths	3.5 (0.5 to 14.8)	2.8 (0.5 to 11.9)	4 (0.3 to 17.6)	2.6 (0.4 to 10.8)	2.2 (0.4 to 9.4)	3.1 (0.3 to 12.6)	-24.4 (-54 to 53.5)	-21.5 (-60.1 to 80.4)	-24.1 (-56.7 to 60.1)
		DALYs	87 (10.3 to 372)	73.3 (12.2 to 320.6)	99.3 (6.9 to 428.1)	65.6 (8 to 274.6)	56.3 (9.5 to 240.3)	75.9 (5.6 to 311.7)	-24.6 (-55.7 to 55)	-23.2 (-62.5 to 88.1)	-23.5 (-55.4 to 56.7)

Location	Risk factor	Measure	1990			2019			% Change (1990 to 2019)		
			Both	Female	Male	Both	Female	Male	Both	Female	Male
		YLLs	82.5 (9.6 to 353.2)	69.3 (11.4 to 305.2)	94.4 (6.5 to 411)	61 (7.3 to 256.3)	52.3 (8.7 to 223.5)	70.7 (5.2 to 293.3)	-26 (-56.8 to 53.8)	-24.6 (-63.1 to 84.8)	-25.1 (-56.4 to 54.8)
		YLDs	4.5 (0.5 to 18.4)	4 (0.7 to 17.4)	4.9 (0.3 to 20)	4.6 (0.6 to 18)	4 (0.7 to 17.2)	5.2 (0.4 to 20.3)	2.2 (-38.4 to 94.1)	0.2 (-53.4 to 135.1)	5.3 (-32.4 to 100.6)
	Diet low in fiber	Deaths	7.7 (1.5 to 15.2)	8.7 (1.7 to 17.6)	6.7 (1.4 to 13.6)	9 (2.2 to 16.5)	10.5 (2.4 to 19.5)	7.5 (1.9 to 13.8)	16.7 (-11.9 to 68.4)	20.9 (-12.8 to 82.1)	11.8 (-17 to 60.5)
		DALYs	200.9 (38.6 to 396.3)	236.6 (45.3 to 474.1)	160.1 (32.2 to 331.4)	236.5 (59 to 429)	284.5 (67.9 to 507.4)	188.4 (46.5 to 348)	17.7 (-13.6 to 76)	20.3 (-15.8 to 85.3)	17.7 (-14.8 to 74.8)
		YLLs	189.6 (35.9 to 378.8)	222.8 (42.1 to 450.8)	151.5 (30.5 to 320.5)	218.7 (53.4 to 403.7)	262.7 (61.6 to 474.4)	174.6 (42.9 to 325.8)	15.3 (-16.6 to 75.3)	17.9 (-19.1 to 83.9)	15.2 (-17.8 to 74.1)
		YLDs	11.3 (2.2 to 21.5)	13.7 (2.5 to 26)	8.7 (1.7 to 16.5)	17.9 (4.5 to 31.4)	21.8 (5.3 to 38.2)	13.9 (3.6 to 24.4)	57.9 (31.2 to 122.1)	58.8 (31 to 129.6)	60 (31.9 to 124.6)
	Diet low in fruits	Deaths	16.1 (8.5 to 27.1)	18.4 (9.8 to 31.5)	13.7 (7.1 to 23.3)	11.3 (5.6 to 19)	13.3 (6.5 to 22.5)	9.3 (4.4 to 16.1)	-29.8 (-49.7 to -3.6)	-27.8 (-49.1 to 0.6)	-32.3 (-51.3 to -7.5)
		DALYs	442.2 (240.2 to 734)	525.6 (277.3 to 879)	348.7 (183.7 to 586.8)	306.1 (156.6 to 499.8)	370.3 (189.6 to 609.3)	241.4 (118.2 to 408.4)	-30.8 (-49.8 to -4.2)	-29.5 (-50.2 to 0.7)	-30.8 (-50.2 to -3.5)
		YLLs	420.8 (223.5 to 700.1)	499.3 (258.6 to 848.9)	332.6 (169.8 to 567.7)	285.4 (146.4 to 470)	344.9 (173.6 to 574.5)	225.5 (111.7 to 389.1)	-32.2 (-51.8 to -4.8)	-30.9 (-51.7 to 0.3)	-32.2 (-51.6 to -4.3)
		YLDs	21.4 (10.4 to 35.6)	26.3 (12.9 to 43.4)	16.1 (7.6 to 27.2)	20.7 (9.1 to 34.9)	25.4 (11.2 to 42.6)	15.9 (7.1 to 27.3)	-3.3 (-19.5 to 9.3)	-3.5 (-20.1 to 10)	-1.1 (-17.2 to 12.2)
	Diet low in vegetables	Deaths	13.7 (5.8 to 22.9)	15.4 (6.2 to 26.7)	11.8 (5.1 to 20)	9.6 (4.2 to 15.5)	11.1 (4.9 to 18.1)	8 (3.5 to 13.1)	-29.9 (-48.8 to -4)	-27.7 (-48 to 1.5)	-32.5 (-50.7 to -9.1)
		DALYs	369.8 (153.1 to 621.9)	434.8 (180 to 748.7)	296.9 (125.7 to 500.6)	257.1 (113.1 to 415.5)	308.1 (134.9 to 505.6)	205.6 (91.1 to 337.9)	-30.5 (-50 to -3.2)	-29.1 (-49.8 to 1.6)	-30.7 (-50.1 to -4.5)
		YLLs	352.8 (139.7 to 603.3)	414 (166.1 to 717.6)	283.9 (117.9 to 480.1)	240.5 (104.8 to 393.9)	287.9 (127.9 to 474.1)	192.7 (85.3 to 319)	-31.8 (-51.7 to -3.9)	-30.5 (-51.5 to 0.9)	-32.1 (-51.7 to -5)
		YLDs	17 (7.5 to 27.4)	20.8 (9.2 to 33.4)	13 (5.6 to 20.8)	16.6 (7 to 27.5)	20.2 (8.5 to 33.8)	12.9 (5.5 to 21.2)	-2.4 (-18.1 to 11.2)	-2.9 (-19.1 to 12)	-0.1 (-14.6 to 13.7)
	Diet low in whole grains	Deaths	7 (4 to 10.9)	7.7 (4.3 to 11.8)	6.4 (3.4 to 10.5)	8.7 (4.7 to 12.7)	9.9 (5.2 to 14.8)	7.5 (4.1 to 11.4)	24.5 (-3.7 to 56.8)	29.1 (-1.9 to 64.3)	17.9 (-10.9 to 53.4)
		DALYs	143.7 (78.3 to 225.6)	162 (84.9 to 260)	125.1 (65.2 to 208.1)	186.8 (96.5 to 282.6)	217.6 (110 to 325.6)	155.3 (80.2 to 245.9)	30 (-0.4 to 63.2)	34.3 (1.8 to 71.6)	24.1 (-6.5 to 63.5)
		YLLs	126.8 (68.9 to 204)	141.6 (74.1 to 232.9)	111.5 (55.7 to 190.3)	166.8 (86.3 to 252.5)	193.6 (95.3 to 297.8)	139.3 (71.2 to 225.7)	31.5 (-2.8 to 70.4)	36.7 (0.2 to 80.4)	24.9 (-8.8 to 71.1)
		YLDs	17 (8.9 to 24.6)	20.3 (10.7 to 29.6)	13.6 (7.1 to 20)	20.1 (10.2 to 29.4)	24 (12.1 to 35.5)	16 (8.1 to 23.3)	18.3 (10.2 to 25.7)	17.8 (7.4 to 26.8)	17.5 (6.6 to 29.2)
	High body-mass index	Deaths	29.5 (13.7 to 49.4)	41 (20 to 66)	17.9 (6.2 to 34.7)	33.3 (18.7 to 51.1)	43.2 (24.4 to 66.7)	22.7 (11.9 to 37.4)	12.9 (-19.6 to 69.6)	5.4 (-27.3 to 55)	26.4 (-13.3 to 133.7)
		DALYs	963.6 (461.5 to 1583.3)	1357.2 (698.8 to 2135.6)	547.1 (193.7 to 1030.4)	1033 (612.1 to 1572.6)	1373.2 (825.9 to 2071)	681.5 (359.5 to 1095.6)	7.2 (-22.8 to 59.5)	1.2 (-29 to 50.6)	24.6 (-15.7 to 130)
		YLLs	914.8 (432.1 to 1520.3)	1286.9 (649.1 to 2055.1)	520.6 (181.8 to 997.3)	961 (560.2 to 1480.6)	1276.2 (753.5 to 1964)	635.3 (330.6 to 1037)	5 (-26 to 57.7)	-0.8 (-31.3 to 50.5)	22 (-18.9 to 129.2)
		YLDs	48.8 (24.3 to 80.1)	70.3 (37.6 to 110.1)	26.4 (9.4 to 48.7)	72 (43.3 to 107.2)	97 (60.8 to 141.5)	46.2 (24.5 to 72.4)	47.7 (24.4 to 98.4)	37.9 (15.7 to 83.9)	74.8 (34.8 to 192.8)
	High fasting plasma glucose	Deaths	38 (21 to 63.8)	49.1 (24.8 to 84.5)	28.1 (16.3 to 47.1)	53.8 (30.1 to 91.9)	61.6 (33.4 to 107)	45.6 (25.8 to 77.9)	41.7 (0.9 to 100.2)	25.5 (-15.5 to 89.8)	62.2 (7.5 to 133)
		DALYs	782.6 (468.4 to 1224.3)	1025.2 (548.6 to 1672)	562.2 (350.7 to 901.6)	1146.7 (695.3 to 1821.3)	1362.5 (812.6 to 2203.4)	922.3 (557.2 to 1457.8)	46.5 (1.9 to 107)	32.9 (-11.7 to 108.4)	64.1 (11.9 to 130.2)
		YLLs	740.3 (435 to 1166.1)	966.5 (503.3 to 1590.4)	534.6 (325.2 to 869.4)	1067 (635.2 to 1721.2)	1265.4 (737.8 to 2059.6)	861 (514.4 to 1345.7)	44.1 (-1.4 to 107.8)	30.9 (-14.8 to 107.3)	61 (8.5 to 127.6)
		YLDs	42.3 (23.2 to 70.5)	58.7 (30.8 to 103.9)	27.6 (16 to 45.4)	79.7 (44.4 to 131)	97.1 (54.7 to 158.1)	61.3 (33.8 to 100.1)	88.4 (51.6 to 143.5)	65.5 (26 to 134.6)	122.4 (66.2 to 192.5)
	High LDL cholesterol	Deaths	14.7 (5.3 to 31.9)	16.9 (6.1 to 36.8)	12.6 (4.3 to 27.4)	19.8 (7.8 to 40.4)	23.6 (9.4 to 48.9)	15.9 (6.1 to 33.8)	34.3 (4.5 to 75.6)	39.5 (4.9 to 88.9)	25.9 (-6.1 to 71.2)
		DALYs	326.5 (175.2 to 568.9)	385.5 (208.9 to 673.1)	265.7 (131.5 to 488.7)	461.2 (265.1 to 763.5)	563.9 (320 to 946.8)	357.2 (191.1 to 624.4)	41.3 (8.1 to 83.8)	46.3 (9.7 to 94.8)	34.4 (-0.5 to 80)
		YLLs	285 (145.9 to 512.2)	334.2 (168.2 to 601)	234.1 (110.3 to 447)	408.9 (222.6 to 702.3)	498.8 (276.3 to 855.1)	317.8 (163.4 to 576.2)	43.5 (6 to 94.8)	49.3 (7.7 to 108.7)	35.8 (-3.3 to 90.1)
		YLDs	41.5 (25.3 to 67)	51.3 (31.4 to 82.7)	31.6 (18.6 to 51)	52.4 (32.6 to 83.5)	65.1 (40.8 to 102.2)	39.3 (24 to 62.7)	26 (17 to 36.3)	26.9 (15.6 to 40.3)	24.3 (11.2 to 41.3)
	High systolic blood pressure	Deaths	94.3 (60.8 to 128.4)	110.1 (66.4 to 154.1)	79.2 (50.4 to 112.4)	76.2 (49.3 to 106.5)	90.2 (53.7 to 131.1)	61.5 (39.7 to 88.3)	-19.2 (-40.8 to 5.3)	-18 (-42.1 to 14.5)	-22.4 (-43 to 4.6)
		DALYs	2233.2 (1518.3 to 3020.1)	2655.2 (1697.2 to 3638.7)	1805.3 (1180.2 to 2508.3)	1759.3 (1176.5 to 2416.6)	2100.8 (1340 to 2985)	1399.8 (941.2 to 1974.6)	-21.2 (-42.2 to 3.7)	-20.9 (-44.6 to 11)	-22.5 (-42.6 to 6)
		YLLs	2118.2 (1410.3 to 2902.6)	2511.5 (1576.1 to 3485.4)	1718 (1097.6 to 2426.8)	1638.8 (1060.4 to 2277.9)	1955.1 (1182.9 to 2842.4)	1306.4 (844 to 1849.3)	-22.6 (-44 to 3.4)	-22.2 (-45.9 to 10.8)	-24 (-44.6 to 4.6)
		YLDs	115.1 (80.2 to 152.3)	143.7 (96.9 to 191.5)	87.3 (59.6 to 117.1)	120.4 (83.1 to 159.3)	145.7 (99.3 to 195.1)	93.4 (62.2 to 124.8)	4.7 (-10.5 to 23.4)	1.4 (-18.8 to 31.1)	7 (-11.2 to 28.3)
	High temperature	Deaths	1.3 (-0.5 to 3.8)	1.4 (-0.5 to 4.2)	1.2 (-0.4 to 3.4)	1 (-0.2 to 2.8)	1.2 (-0.2 to 3.1)	0.9 (-0.1 to 2.4)	-20.9 (-197.5 to 113)	-18 (-200.4 to 124.4)	-25 (-188.3 to 101.1)
		YLLs	27.5 (-10 to 79.3)	31.4 (-10.9 to 90.9)	23.4 (-8.3 to 68.9)	20.5 (-3.1 to 54.7)	24 (-3.6 to 63.5)	17 (-2.7 to 45.5)	-25.3 (-193.2 to 101.4)	-23.5 (-194.2 to 108.8)	-27.4 (-185.7 to 91.4)
	Household air pollution from solid fuels	Deaths	82.7 (51.8 to 121.8)	95.8 (58.9 to 141.2)	69.8 (43.3 to 105.6)	45.9 (29.8 to 65.2)	55.8 (34.5 to 80.5)	35.6 (22.4 to 52.6)	-44.5 (-58.1 to -28.4)	-41.8 (-56.3 to -23.9)	-49 (-61.6 to -32.8)
		DALYs	2004.6 (1330.6 to 2810.7)	2390.6 (1584.9 to 3426)	1596.8 (1009.4 to 2326.5)	1120.1 (755.8 to 1574.4)	1388.7 (912.8 to 1987.5)	841.8 (537.8 to 1220.7)	-44.1 (-57.9 to -26.8)	-41.9 (-57.4 to -22.5)	-47.3 (-60.9 to -29.5)
		YLLs	1896.9 (1240.2 to 2685.5)	2256.2 (1459.7 to 3258.3)	1516 (941.8 to 2228.1)	1039.6 (689.2 to 1480.4)	1287.3 (821.6 to 1876.4)	783 (494.5 to 1160.9)	-45.2 (-59.5 to -27.3)	-42.9 (-58.8 to -22.4)	-48.4 (-62.3 to -29.6)
		YLDs	107.7 (72.3 to 147.2)	134.4 (90.6 to 183.3)	80.8 (53.7 to 113)	80.5 (53.1 to 110)	101.3 (68.1 to 138.2)	58.8 (37.2 to 83)	-25.2 (-35.2 to -16.9)	-24.6 (-34.1 to -16.1)	-27.3 (-40.4 to -16.7)
	Kidney dysfunction	Deaths	13.8 (8.9 to 19.6)	16.7 (9.8 to 24.4)	11.1 (7.4 to 16.4)	15.5 (9.7 to 22.1)	18.4 (11.2 to 27.3)	12.4 (8.1 to 17.5)	12 (-15.3 to 42.2)	10 (-17 to 45.1)	11.6 (-16.7 to 44.3)
		DALYs	321.4 (221.5 to 440.4)	400.1 (260.3 to 565.9)	242.5 (170.3 to 345.1)	345.8 (241.5 to 470.4)	423.1 (281.3 to 592)	263.9 (186 to 357.5)	7.6 (-17.3 to 35.5)	5.7 (-20.4 to 37.2)	8.8 (-17 to 40.6)
		YLLs	301.9 (203.6 to 422.4)	374.4 (237.7 to 535.4)	228.9 (158.1 to 330.2)	319.3 (217.2 to 441.6)	389.9 (249.7 to 552.9)	244.6 (168.9 to 334.7)	5.8 (-20.4 to 35)	4.1 (-23 to 38.3)	6.8 (-20.2 to 40.2)
		YLDs	19.6 (13.5 to 25.7)	25.7 (17.7 to 34)	13.6 (9.3 to 18.2)	26.5 (18.3 to 35.2)	33.2 (22.8 to 44.2)	19.3 (13.1 to 25.8)	35.5 (25.3 to 45.5)	29.1 (18.5 to 40.2)	42.3 (28 to 57.2)
	Lead exposure	Deaths	21.1 (13.2 to 30.2)	20 (11.3 to 29.7)	22 (13.6 to 32)	19.3 (12.1 to 28)	19.2 (11.3 to 28.3)	19.5 (12.5 to 28.1)	-8.5 (-30.2 to 15.9)	-3.8 (-28.4 to 25.8)	-11.4 (-32.4 to 13.8)
		DALYs	523.3 (330.4 to 753.1)	514.5 (308.7 to 753)	524.5 (329.2 to 762.8)	434.9 (284.8 to 613.6)	441.8 (269.8 to 643.5)	429.8 (285.4 to 615.7)	-16.9 (-37.4 to 7)	-14.1 (-36.6 to 13.8)	-18.1 (-37.7 to 7.2)
		YLLs	496.7 (307.9 to 722.7)	486.8 (283.4 to 721.4)	499.1 (307.8 to 735.8)	405.6 (258.5 to 580.3)	411.5 (244.1 to 612.2)	401.6 (259.8 to 583)	-18.3 (-39.5 to 6.7)	-15.5 (-38.8 to 14.1)	-19.5 (-39.6 to 7)
		YLDs	26.6 (17.2 to 37.3)	27.7 (17.2 to 39.2)	25.4 (16.7 to 35.3)	29.2 (18.8 to 41.1)	30.3 (19.2 to 43.2)	28.2 (18.4 to 39)	9.8 (0.9 to 19.6)	9.4 (-1.8 to 23.3)	10.9 (-0.9 to 22.7)
	Low physical [illegible]	Deaths	6.4 (1.3 to 14.2)	7 (1.6 to 15.9)	5.9 (1.1 to 13.1)	8.1 (1.7 to 18)	9.3 (2.1 to 20.6)	6.9 (1.3 to 15.4)	27.2 (-0.5 to 60.4)	33.5 (0.7 to 75.7)	18.5 (-11.2 to 59.7)

Location	Risk factor	Measure	1990			2019			% Change (1990 to 2019)		
			Both	Female	Male	Both	Female	Male	Both	Female	Male
	activity	DALYs	98.6 (19.2 to 231.3)	110.3 (22.2 to 259.4)	87.5 (15.6 to 210.3)	129.9 (25.1 to 306.9)	151.6 (31.4 to 365.9)	107.5 (19.2 to 260.3)	31.8 (2.6 to 64.1)	37.4 (2.7 to 76.3)	22.9 (-8 to 59.7)
		YLLs	89 (17.6 to 208.8)	98.7 (20.2 to 236.7)	79.8 (14.5 to 191.6)	118.3 (23 to 281.7)	137.6 (27.6 to 328.8)	98.4 (18.1 to 238.6)	32.8 (1 to 69.1)	39.3 (1.7 to 82.1)	23.2 (-9.5 to 64.9)
		YLDs	9.5 (1.6 to 24.4)	11.6 (2.2 to 29.2)	7.6 (1.2 to 20)	11.6 (2 to 30)	14 (2.6 to 36.1)	9.1 (1.4 to 24.2)	21.8 (9.2 to 34.6)	21.2 (4.7 to 38.9)	19.1 (1.6 to 35.8)
	Low temperature	Deaths	18.8 (11.2 to 27.6)	20.8 (11.5 to 31)	16.8 (10 to 25)	15.4 (9.1 to 22.6)	17.6 (9.9 to 26.2)	13.1 (7.9 to 19.9)	-18 (-36.1 to 1.3)	-15.1 (-35.1 to 9)	-22.2 (-40.4 to -2.1)
		YLLs	400.5 (241.2 to 573.3)	457.8 (262.7 to 682.1)	339.6 (203.7 to 508)	310 (185.3 to 451)	362.6 (209.9 to 532.1)	255.7 (153.8 to 388.8)	-22.6 (-41.2 to -1.5)	-20.8 (-41.3 to 3.5)	-24.7 (-42.9 to -2.7)
	Secondhand smoke	Deaths	7 (4.4 to 9.9)	8.5 (5.1 to 12.2)	5.4 (3.2 to 7.9)	6 (3.9 to 8.5)	7.5 (4.5 to 10.7)	4.4 (2.8 to 6.5)	-14.5 (-34.5 to 7.6)	-12.5 (-34.7 to 14.4)	-17.4 (-38 to 10.6)
		DALYs	189.3 (119.8 to 269.4)	244.7 (152.5 to 349.2)	128 (77.7 to 193.2)	154.5 (101 to 218.8)	204.4 (130.4 to 296.2)	104.2 (65.8 to 154.9)	-18.4 (-39.4 to 6.2)	-16.4 (-39.1 to 13.9)	-18.6 (-39.4 to 11)
		YLLs	183.3 (115.8 to 260.8)	236.5 (145.7 to 339.9)	124.2 (74.6 to 189.6)	147.8 (94.9 to 212.7)	195.4 (122 to 288.1)	100 (62.7 to 149.1)	-19.4 (-40.5 to 6)	-17.4 (-41 to 13.7)	-19.5 (-40.9 to 10.6)
		YLDs	6 (3.8 to 8.5)	8.1 (5.1 to 11.5)	3.8 (2.4 to 5.4)	6.7 (4.3 to 9.4)	9.1 (5.8 to 12.7)	4.3 (2.7 to 6)	10.5 (0.2 to 21.5)	11.4 (-0.8 to 24.9)	12.1 (-4.2 to 31.2)
	Smoking	Deaths	7.9 (5.3 to 10.8)	3.6 (2.2 to 5.4)	11.8 (7.8 to 16.5)	9.3 (6.4 to 12.9)	4.7 (2.8 to 7.1)	14.4 (9.8 to 20.1)	17.7 (-13.7 to 59.4)	28 (-17.5 to 105.1)	22 (-9.1 to 62.9)
		DALYs	213.2 (146.3 to 285.7)	109.8 (68.2 to 163.6)	310.7 (209.4 to 436.8)	269.9 (190.2 to 369.4)	145.8 (90.5 to 217.3)	403.2 (277.5 to 559.6)	26.6 (-8.6 to 76.4)	32.8 (-15.8 to 113.5)	29.8 (-5.9 to 78.4)
		YLLs	202 (135.8 to 273.7)	103.5 (62.6 to 156)	294.7 (195.6 to 414.9)	250.1 (170.4 to 345.1)	134.7 (80.9 to 204)	374.1 (251.9 to 528.9)	23.8 (-11.7 to 74.4)	30.1 (-18.9 to 112.2)	27 (-9.4 to 76.6)
		YLDs	11.2 (7.8 to 14.7)	6.3 (4.1 to 8.8)	16 (11.2 to 21.2)	19.7 (14.1 to 26.1)	11 (7.1 to 16.2)	29.1 (20.5 to 37.4)	76 (48.6 to 108.8)	76.1 (26.6 to 149.6)	81.9 (55.6 to 111.2)
Algeria	All risk factors	Deaths	146.2 (120.5 to 176)	161.1 (127.8 to 194.7)	137.2 (108.7 to 170.1)	84.8 (68.6 to 104.3)	99.9 (77.4 to 121.8)	75.4 (57.9 to 96)	-42 (-54 to -28)	-38 (-51.9 to -22.4)	-45 (-58.2 to -28.5)
		DALYs	2625.7 (2165.1 to 3168.6)	2832 (2274.6 to 3421.8)	2470.6 (1956.2 to 3102.5)	1503.9 (1242.7 to 1802.3)	1681.6 (1342.6 to 2013.7)	1370.4 (1076.5 to 1712.2)	-42.7 (-54.3 to -29.1)	-40.6 (-52.8 to -27.1)	-44.5 (-57 to -28.6)
		YLLs	2395.6 (1951 to 2939.2)	2560.1 (2019.5 to 3144)	2281.6 (1759.4 to 2908.8)	1295.2 (1031 to 1598)	1435.2 (1099.6 to 1758.4)	1197.7 (912.6 to 1536.8)	-45.9 (-58.4 to -31.6)	-43.9 (-57 to -29.1)	-47.5 (-60.7 to -30.3)
		YLDs	230.2 (168.1 to 292.8)	271.9 (196.7 to 344.9)	189 (137.1 to 242)	208.7 (151.6 to 265.2)	246.4 (179.8 to 315.5)	172.7 (124.9 to 219.6)	-9.3 (-14.6 to -3.7)	-9.4 (-16 to -2.5)	-8.6 (-14.9 to -1.3)
	Alcohol use	Deaths	0.2 (-0.2 to 0.5)	-0.2 (-0.4 to 0)	0.5 (-0.1 to 1.2)	0.2 (-0.1 to 0.4)	-0.2 (-0.5 to 0)	0.5 (0.1 to 0.9)	-5.8 (-398.5 to 832.9)	11.9 (-153.8 to 229.4)	-4.2 (-294.5 to 442.5)
		DALYs	7.1 (-0.4 to 16.3)	-2.3 (-6.6 to 2.5)	16.5 (2.3 to 33.6)	6.7 (0.4 to 13.2)	-2.6 (-6.6 to 1.1)	15.6 (4.9 to 27.9)	-5.7 (-163.2 to 363.6)	17.4 (-502.8 to 443.5)	-5.6 (-54.8 to 190.5)
		YLLs	7 (-0.1 to 15.7)	-1.7 (-5.7 to 2.7)	15.7 (2.7 to 32.2)	6.1 (0.5 to 11.9)	-2 (-5.5 to 1.1)	13.8 (4.4 to 24.8)	-12.4 (-100.6 to 263.3)	18.9 (-480.3 to 669.9)	-12 (-57.5 to 154.1)
		YLDs	0.1 (-0.5 to 0.8)	-0.5 (-1.1 to -0.1)	0.8 (-0.4 to 2.1)	0.6 (-0.2 to 1.5)	-0.6 (-1.3 to 0)	1.7 (0.3 to 3.4)	411.6 (-2121.2 to 1439.2)	12.8 (-71.3 to 77.2)	121.1 (-1355.3 to 1437.6)
	Ambient particulate matter pollution	Deaths	27.7 (17.1 to 39.5)	28.8 (17.2 to 42.5)	27.3 (16.7 to 39.5)	19.5 (12.8 to 26.7)	22.3 (14.8 to 30.7)	17.5 (11.3 to 24.8)	-29.8 (-46.9 to -4.9)	-22.5 (-43.4 to 9)	-35.7 (-53.4 to -11.2)
		DALYs	578.5 (362.6 to 834.6)	597.2 (360.8 to 876.7)	566 (356.4 to 822.8)	409 (278 to 550.1)	451.9 (306 to 607.5)	373.5 (249.9 to 522.2)	-29.3 (-46 to -3.6)	-24.3 (-44.2 to 6)	-34 (-50.7 to -9.9)
		YLLs	523.4 (326.1 to 751.4)	534.2 (320.7 to 775.1)	518.7 (327.5 to 762.4)	346.3 (230 to 474.4)	377.6 (250.8 to 518.9)	322.1 (209 to 460.5)	-33.8 (-50.5 to -9.2)	-29.3 (-49.4 to 1.4)	-37.9 (-54.9 to -13.2)
		YLDs	55.1 (32.6 to 82.1)	63 (37.1 to 94.7)	47.3 (28.3 to 69.5)	62.7 (41 to 86.4)	74.3 (49.5 to 103.1)	51.4 (33.6 to 70.4)	13.7 (-1.3 to 44.3)	18.1 (-0.6 to 55.5)	8.7 (-5.2 to 36.8)
	Diet high in red meat	Deaths	4 (1.4 to 6.7)	4.3 (1.6 to 7.2)	3.8 (1.2 to 6.4)	2.5 (0.8 to 4.2)	2.9 (0.9 to 4.9)	2.3 (0.8 to 3.8)	-36.8 (-59.1 to -6.5)	-32.9 (-57.1 to 5.8)	-39.7 (-61.8 to -8.7)
		DALYs	93.1 (31.8 to 155.6)	100.9 (36.1 to 166.9)	86.3 (29.4 to 146.4)	59 (20.7 to 94.5)	65.5 (23.4 to 108.1)	53.5 (18.2 to 87.4)	-36.7 (-58.3 to -4.1)	-35.2 (-57.9 to 3)	-38 (-59.8 to -8.3)
		YLLs	83.8 (27.7 to 141.3)	89.8 (30.4 to 148.8)	78.7 (26.2 to 135.8)	49.4 (17.6 to 81.2)	54.1 (19.2 to 91.4)	45.7 (15.9 to 75.6)	-41.1 (-61.3 to -7.3)	-39.8 (-61.2 to 1.2)	-42 (-62.4 to -11.2)
		YLDs	9.4 (2.8 to 16)	11.1 (3.3 to 18.9)	7.6 (2.3 to 12.9)	9.6 (2.6 to 16.2)	11.4 (3.1 to 19.3)	7.8 (2.1 to 13.3)	2.3 (-17.5 to 22.5)	2.6 (-18.8 to 26.9)	2.7 (-17.4 to 23)
	Diet high in sodium	Deaths	2.7 (0.5 to 11.3)	2 (0.5 to 8)	3.3 (0.3 to 13.9)	1.4 (0.3 to 5.5)	1.1 (0.3 to 4.3)	1.7 (0.2 to 6.8)	-46.7 (-66.9 to 2.5)	-44.6 (-70.2 to 16.1)	-47.9 (-72.7 to 19)
		DALYs	56.6 (8 to 234.2)	42.4 (8.7 to 171.4)	70.9 (5.8 to 295.3)	30.4 (4.5 to 118.2)	22.9 (5.2 to 93.3)	37.7 (3.2 to 150.6)	-46.2 (-66.4 to -5.1)	-46 (-70.9 to 16.4)	-46.8 (-69 to 6.8)
		YLLs	51.5 (7.3 to 213.4)	38 (8 to 156.8)	65 (5.3 to 271.3)	26 (3.8 to 100.8)	19.2 (4.4 to 79.9)	32.6 (2.7 to 130.7)	-49.5 (-69.3 to -8.7)	-49.5 (-72.9 to 10.1)	-49.9 (-71.4 to 3)
		YLDs	5.1 (0.6 to 21.1)	4.4 (0.8 to 18.5)	5.8 (0.4 to 23.2)	4.5 (0.6 to 17.6)	3.7 (0.7 to 15.3)	5.2 (0.4 to 20)	-13.1 (-46.3 to 60)	-15.9 (-58.6 to 108.1)	-11.6 (-42.6 to 77.3)
	Diet low in fiber	Deaths	6 (1.3 to 11.2)	6.6 (1.4 to 12.7)	5.6 (1.2 to 10.7)	1.4 (0.4 to 3)	1.6 (0.4 to 3.6)	1.3 (0.3 to 2.7)	-76.7 (-84.4 to -65.8)	-75.3 (-85.9 to -60.6)	-77.5 (-86.3 to -64.5)
		DALYs	123.3 (24.7 to 234.1)	134.9 (27.2 to 258)	113.6 (22.7 to 218.8)	26 (6 to 57)	28.7 (6.6 to 63.2)	23.9 (5.3 to 52.3)	-78.9 (-85.5 to -69.6)	-78.7 (-86.6 to -68)	-78.9 (-86.4 to -69.1)
		YLLs	111.4 (22.3 to 212.6)	120.8 (24.1 to 233.1)	103.9 (20.7 to 203.8)	22.1 (5.2 to 48.4)	24.3 (5.6 to 54.1)	20.7 (4.7 to 45.3)	-80.1 (-86.5 to -70.7)	-79.9 (-87.6 to -69.6)	-80.1 (-87.5 to -70)
		YLDs	11.9 (2.3 to 23.3)	14.2 (2.7 to 27.8)	9.7 (1.9 to 18.8)	3.8 (0.8 to 8.5)	4.5 (1 to 10.4)	3.3 (0.7 to 7.2)	-67.7 (-77 to -57)	-68.5 (-79.2 to -57.1)	-66.4 (-76.4 to -54.3)
	Diet low in fruits	Deaths	9.4 (4.5 to 15.6)	10.3 (5 to 17.2)	8.7 (4.1 to 15)	3.2 (1.3 to 5.7)	3.9 (1.5 to 7)	2.8 (1.1 to 5)	-65.4 (-79.4 to -49.4)	-62 (-78.3 to -42.6)	-68 (-81.3 to -51.2)
		DALYs	208.8 (105.6 to 346.3)	229.1 (114.7 to 382.6)	191 (94.2 to 322.9)	68.4 (29.3 to 117.9)	78.9 (31.5 to 137.1)	59.6 (25.1 to 106.2)	-67.3 (-80.6 to -50.8)	-65.6 (-80.2 to -47.9)	-68.8 (-81.8 to -52.3)
		YLLs	190.1 (96.4 to 323.7)	206.7 (104 to 352.3)	175.9 (87.5 to 302.7)	58.3 (24.9 to 99.9)	66.6 (28.2 to 115.5)	51.7 (22 to 92.1)	-69.3 (-81.8 to -52.7)	-67.8 (-81.4 to -49.7)	-70.6 (-83.2 to -54.1)
		YLDs	18.7 (7.7 to 33.6)	22.4 (9.3 to 40.1)	15.1 (6.1 to 27.1)	10.1 (3.3 to 18.8)	12.3 (4 to 22.9)	7.9 (2.6 to 14.9)	-46.2 (-68.5 to -29.7)	-45.1 (-67.6 to -27.5)	-47.4 (-68.9 to -28.4)
	Diet low in vegetables	Deaths	5.8 (1.9 to 9.8)	6.3 (2 to 10.6)	5.6 (1.9 to 9.8)	1.8 (0.6 to 3.4)	2.1 (0.7 to 3.9)	1.7 (0.5 to 3.2)	-68.4 (-84.5 to -48)	-66.5 (-84.3 to -43.2)	-69.7 (-85.3 to -49.5)
		DALYs	122.9 (36 to 209.5)	131.3 (41.1 to 226.7)	115.9 (36.6 to 204.4)	36.1 (10.5 to 67.8)	39.1 (10.9 to 73.8)	33.9 (9.9 to 66.2)	-70.6 (-86.5 to -47.4)	-70.2 (-86.7 to -44.2)	-70.8 (-86.4 to -48.2)
		YLLs	112.4 (31.5 to 193.5)	119 (36.7 to 209.1)	107.3 (32.8 to 192.1)	30.8 (9.2 to 59.4)	33 (9.4 to 63.1)	29.4 (8.7 to 58.9)	-72.6 (-87.1 to -49.9)	-72.3 (-87.3 to -47.1)	-72.6 (-87.2 to -50.8)
		YLDs	10.5 (3.5 to 18.3)	12.3 (4.1 to 21.6)	8.7 (3 to 15.1)	5.3 (1.1 to 10.2)	6.1 (1.3 to 11.9)	4.5 (1 to 8.5)	-49.5 (-75.9 to -22.2)	-50.3 (-77.3 to -22.3)	-48 (-74.7 to -20.2)
	Diet low in whole grains	Deaths	7.9 (4.4 to 10.8)	9 (5 to 12.5)	7.2 (3.8 to 10.2)	5.6 (2.8 to 7.8)	6.7 (3.3 to 9.4)	4.9 (2.4 to 7.1)	-29.3 (-45.5 to -11)	-26.3 (-44.5 to -5.7)	-31.8 (-49.8 to -7.4)
		DALYs	134.4 (69.4 to 184.6)	148.9 (77.1 to 209.9)	123.9 (63 to 177.2)	101.6 (49 to 145.7)	115.3 (54.9 to 162.8)	91.1 (42.7 to 133)	-24.4 (-40.6 to -6.4)	-22.6 (-40.3 to -3.4)	-26.5 (-45.1 to -2.8)
		YLLs	115 (60 to 160.7)	125.6 (66.4 to 178.8)	108.4 (54.8 to 157.7)	83.3 (40.4 to 119.4)	93.5 (44.8 to 135.6)	76.3 (35.3 to 113.3)	-27.5 (-44.9 to -6.6)	-25.6 (-45.3 to -3.5)	-29.6 (-49.6 to -2.5)
		YLDs	19.4 (9.1 to 29.1)	23.3 (11.3 to 35)	15.5 (7 to 23.4)	18.3 (8.2 to 27.5)	21.8 (10 to 32.8)	14.8 (6.7 to 22.6)	-5.9 (-13.2 to 1.4)	-6.4 (-16.3 to 4.2)	-4.5 (-13.7 to 5.5)

Location	Risk factor	Measure	1990 Both	1990 Female	1990 Male	2019 Both	2019 Female	2019 Male	% Change (1990 to 2019) Both	% Change (1990 to 2019) Female	% Change (1990 to 2019) Male
	High body-mass index	Deaths	24.9 (14.3 to 38.4)	31.3 (18.5 to 47.8)	18.9 (8.7 to 32.7)	18.5 (11 to 28)	22.1 (13.2 to 33.4)	15.6 (8.8 to 24.8)	-25.7 (-45.1 to 2.9)	-29.5 (-48.5 to -3.3)	-17.3 (-42.2 to 31.2)
		DALYs	758.4 (465.7 to 1113.7)	964.7 (632.6 to 1362.5)	550.8 (274.8 to 908)	541.6 (362.1 to 750.9)	633.7 (431.4 to 862.4)	455.5 (281.3 to 682)	-28.6 (-45.9 to -4.3)	-34.3 (-49.4 to -12.6)	-17.3 (-41.4 to 27.4)
		YLLs	679.6 (411.9 to 1012.8)	856.5 (540.9 to 1228.3)	502.4 (249.5 to 839.9)	449.9 (289.4 to 640.4)	517.1 (337.8 to 725)	388 (231.3 to 590.6)	-33.8 (-51.4 to -8.4)	-39.6 (-55.9 to -16.9)	-22.8 (-47.2 to 21.4)
		YLDs	78.8 (46.9 to 117.3)	108.2 (67.9 to 155.9)	48.4 (23.4 to 80.4)	91.8 (59.1 to 130.2)	116.7 (77.7 to 163.4)	67.5 (40.4 to 99.8)	16.5 (2.8 to 38.8)	7.8 (-3.9 to 25.6)	39.4 (11.9 to 102.1)
	High fasting plasma glucose	Deaths	30.5 (16.9 to 57.9)	34.9 (18 to 71.5)	27.6 (15.6 to 50.1)	31.5 (16.3 to 62.5)	36 (18 to 72.7)	28.7 (14.8 to 58.9)	3.2 (-29.5 to 43.2)	3.2 (-32.4 to 53.6)	4.1 (-34.5 to 54)
		DALYs	506.9 (318.1 to 852.8)	561.5 (337.7 to 994.9)	465.9 (288.4 to 772)	536.7 (315.2 to 918.5)	586.5 (334.2 to 987)	501.3 (284.2 to 892)	5.9 (-25.1 to 46.9)	4.4 (-28.4 to 50.3)	7.6 (-28.2 to 57.7)
		YLLs	463.4 (286 to 779.7)	509.1 (299.6 to 913.7)	431.1 (264.2 to 713.5)	463.4 (262.6 to 805.2)	501.1 (278.4 to 853.3)	439.5 (245.2 to 790.8)	0 (-30.3 to 40.7)	-1.6 (-33.9 to 44.9)	1.9 (-34.6 to 53.4)
		YLDs	43.5 (24.7 to 72.5)	52.4 (29.6 to 89.6)	34.8 (19.4 to 58.8)	73.3 (40.4 to 121.1)	85.4 (46.4 to 142.6)	61.9 (33.6 to 104.2)	68.6 (33 to 116.4)	63 (20.2 to 117.2)	77.6 (35 to 132.8)
	High LDL cholesterol	Deaths	18.6 (5.5 to 43.7)	22.2 (6.2 to 54)	16.2 (5 to 37.5)	14.4 (4.5 to 33.7)	18.2 (5.1 to 41.8)	12 (3.8 to 27.4)	-22.2 (-37.6 to -0.2)	-18.1 (-36.4 to 6)	-26 (-44.2 to 1)
		DALYs	327.1 (160.9 to 620)	377.5 (181.5 to 737.2)	287.9 (141 to 540.7)	272 (137.7 to 493.8)	323.3 (160.5 to 594.1)	231.1 (116.9 to 421.7)	-16.8 (-32.6 to 5.3)	-14.4 (-32.2 to 10.1)	-19.7 (-38.4 to 7.6)
		YLLs	277.2 (125.8 to 543.1)	315.8 (137.5 to 664.1)	249.7 (114.7 to 485.3)	221 (105.2 to 416.5)	260.1 (118.2 to 499.9)	191.9 (89.4 to 368.7)	-20.3 (-38.1 to 6.2)	-17.7 (-38 to 12.1)	-23.1 (-44.1 to 9.1)
		YLDs	49.9 (29.3 to 81.8)	61.7 (36.3 to 100.6)	38.2 (22.1 to 63.2)	51 (29.7 to 83.6)	63.2 (37.3 to 103.7)	39.2 (22.9 to 65)	2.2 (-4.8 to 9.8)	2.4 (-6.4 to 13.7)	2.6 (-7.2 to 13.7)
	High systolic blood pressure	Deaths	90 (66.9 to 118.7)	103.1 (73.7 to 136.8)	81.1 (57.3 to 111.2)	48.1 (34.3 to 65)	58.4 (38.8 to 80)	41.4 (28.6 to 57.4)	-46.5 (-58.9 to -30.8)	-43.4 (-59.3 to -23.2)	-49 (-63.7 to -31.2)
		DALYs	1667.8 (1296.2 to 2105.6)	1864.4 (1392.7 to 2370.2)	1505.9 (1115.3 to 1997.4)	872 (662.5 to 1114.9)	995.9 (716.9 to 1288.7)	775.7 (563.6 to 1026.9)	-47.7 (-59.3 to -34.5)	-46.6 (-59.3 to -30.7)	-48.5 (-61.8 to -31.6)
		YLLs	1520.5 (1160.1 to 1940.1)	1684.6 (1246.3 to 2173.3)	1390.9 (1004 to 1866.5)	750.7 (558.1 to 975.3)	850.6 (601.1 to 1114.8)	677.3 (484.7 to 918.8)	-50.6 (-62.6 to -36.3)	-49.5 (-62.5 to -32.7)	-51.3 (-65.1 to -33.8)
		YLDs	147.4 (103.6 to 194.8)	179.8 (124.4 to 239.4)	115 (82.2 to 153.4)	121.3 (84 to 161.9)	145.3 (100.2 to 196.8)	98.4 (67.2 to 132.5)	-17.7 (-28.5 to -6.1)	-19.2 (-33 to -2.8)	-14.4 (-27.3 to 0.3)
	High temperature	Deaths	5.5 (1.2 to 10.8)	6.1 (1.3 to 11.6)	5.2 (1.1 to 10.1)	3.3 (1.1 to 5.9)	3.9 (1.4 to 7.1)	2.9 (1 to 5.3)	-40.9 (-57.8 to -7)	-36.3 (-56.7 to 0.5)	-44.3 (-61.8 to -17.6)
		YLLs	89.3 (19.3 to 176.6)	95.6 (20.3 to 185.2)	85 (18.8 to 168.1)	48.8 (17.6 to 89)	54.6 (19.8 to 99.6)	44.7 (16.1 to 81.8)	-45.4 (-62.6 to -13.9)	-42.9 (-60.7 to -8.3)	-47.3 (-64.8 to -20.8)
	Household air pollution from solid fuels	Deaths	7.7 (3.4 to 14.5)	9.7 (4.3 to 18)	6.1 (2.4 to 12.3)	0.1 (0 to 0.1)	0.1 (0 to 0.2)	0 (0 to 0.1)	-99.2 (-99.7 to -98.1)	-99.2 (-99.6 to -97.9)	-99.3 (-99.7 to -98.3)
		DALYs	162.5 (70.9 to 299.1)	201.8 (90.7 to 364.8)	126.5 (51 to 256.1)	1.3 (0.5 to 2.7)	1.7 (0.7 to 3.4)	0.9 (0.3 to 2.1)	-99.2 (-99.7 to -98.1)	-99.2 (-99.7 to -98)	-99.3 (-99.7 to -98.2)
		YLLs	146.6 (63.4 to 270.8)	180.6 (79.9 to 327.3)	116 (46.3 to 234.6)	1.1 (0.4 to 2.2)	1.4 (0.5 to 2.9)	0.8 (0.3 to 1.8)	-99.3 (-99.7 to -98.2)	-99.2 (-99.7 to -98.1)	-99.3 (-99.7 to -98.3)
		YLDs	15.9 (7.2 to 28.9)	21.2 (9.6 to 37.3)	10.5 (4.4 to 21.1)	0.2 (0.1 to 0.4)	0.3 (0.1 to 0.6)	0.1 (0 to 0.3)	-98.7 (-99.5 to -97.1)	-98.7 (-99.5 to -97)	-98.8 (-99.5 to -97.2)
	Kidney dysfunction	Deaths	12.6 (7.7 to 17.4)	14.9 (8.6 to 21.4)	10.9 (7 to 15.1)	9.5 (4.8 to 14.4)	11.1 (4.8 to 17.2)	8.3 (4.5 to 12.8)	-24.6 (-47.3 to -0.8)	-25.8 (-52.4 to -3.1)	-23.2 (-47 to 5)
		DALYs	237.3 (169.9 to 309.6)	277 (190 to 371.7)	201.6 (146.4 to 269.5)	188.2 (124.7 to 256.3)	216.5 (136.6 to 296.8)	163 (107.3 to 226.6)	-20.7 (-38.1 to 1.3)	-21.8 (-39.7 to -0.8)	-19.2 (-39.4 to 7.1)
		YLLs	213.2 (150 to 282.6)	245.9 (162.7 to 336.6)	184.5 (128.9 to 252.8)	158.6 (100.5 to 220.3)	179.6 (108.4 to 252.4)	140.6 (89.7 to 201.7)	-25.6 (-44.1 to -1.9)	-27 (-46.2 to -4)	-23.8 (-45 to 4.3)
		YLDs	24.2 (16.5 to 32.8)	31.1 (21.2 to 42.3)	17.1 (11.6 to 23.3)	29.6 (20.1 to 40.5)	37 (25 to 50.5)	22.4 (15.1 to 31.1)	22.4 (12.5 to 32.4)	18.9 (8 to 30.9)	31 (17.8 to 45.4)
	Lead exposure	Deaths	6.4 (3.2 to 10.7)	5.9 (2.5 to 10.2)	6.9 (3.7 to 11.3)	3.4 (1.6 to 5.6)	3.4 (1.4 to 5.9)	3.5 (1.8 to 5.8)	-46.9 (-59.6 to -32.6)	-42.7 (-57.5 to -24.8)	-49.4 (-62.3 to -32.9)
		DALYs	122.4 (60.7 to 200.5)	110.2 (43.1 to 187.9)	135.6 (73.5 to 216.7)	55.5 (24.6 to 93.2)	51.8 (18.2 to 93.6)	60 (29.1 to 96.1)	-54.7 (-65.8 to -42.4)	-53 (-66.2 to -40.4)	-55.7 (-67.8 to -42.7)
		YLLs	111.8 (55.2 to 183.6)	99.5 (38.5 to 173)	125.1 (66.7 to 203.2)	48.5 (21.2 to 82.3)	44.8 (15.7 to 81.5)	52.9 (25.3 to 85.3)	-56.6 (-67.9 to -43.7)	-54.9 (-68.1 to -40.9)	-57.7 (-69.9 to -43.9)
		YLDs	10.6 (4.8 to 17.9)	10.7 (4.1 to 19)	10.6 (5.2 to 16.9)	7.1 (2.6 to 12.8)	7 (2 to 13.2)	7.2 (3.2 to 12.3)	-33.7 (-47.4 to -24.8)	-35.3 (-53.6 to -24.1)	-32.1 (-45.1 to -22.4)
	Low physical activity	Deaths	10.7 (2.3 to 22)	12.4 (3.2 to 25.6)	9.6 (1.8 to 20.3)	8.2 (1.9 to 16.2)	10.3 (2.6 to 20.2)	7 (1.4 to 14.3)	-22.9 (-38.5 to -0.6)	-16.8 (-36.4 to 12.8)	-27.6 (-45.6 to 1.3)
		DALYs	138.2 (28.3 to 301.9)	156.8 (37.9 to 340)	125.4 (22 to 286.4)	110.3 (23.5 to 231.6)	131.7 (31.8 to 274.7)	95.2 (18.1 to 207.4)	-20.2 (-35.4 to 0.4)	-16.1 (-33.5 to 10.8)	-24.1 (-42.4 to 3.8)
		YLLs	124.5 (25.9 to 266.7)	140.2 (34.2 to 302.4)	114.4 (20.1 to 262.2)	95.5 (20.9 to 198.1)	113.7 (27.7 to 232.2)	83.4 (16.1 to 178.9)	-23.3 (-39.4 to -1.3)	-18.9 (-37.6 to 9.6)	-27.1 (-46.2 to 1.9)
		YLDs	13.8 (2.5 to 34.1)	16.7 (3.3 to 40.9)	11 (1.7 to 27.9)	14.8 (2.8 to 35.5)	18 (3.6 to 43)	11.8 (2 to 29)	7.4 (-3.1 to 25.8)	7.8 (-5.7 to 31.2)	6.9 (-6.7 to 30.1)
	Low temperature	Deaths	8.1 (1.6 to 14.7)	9 (1.8 to 16)	7.6 (1.5 to 14)	4.8 (0.8 to 8.8)	5.7 (1 to 10.5)	4.2 (0.8 to 7.7)	-41 (-56.9 to -24.4)	-36.5 (-54.4 to -17.1)	-44.5 (-59.8 to -26.1)
		YLLs	130.8 (25.6 to 239.7)	140 (27.7 to 250.4)	124.5 (24.2 to 230.1)	71.2 (12.6 to 130.3)	79.7 (14 to 148.5)	65.3 (11.5 to 119.3)	-45.5 (-60.5 to -28.8)	-43.1 (-59.3 to -26.1)	-47.5 (-62.6 to -29.3)
	Secondhand smoke	Deaths	7 (5 to 9.3)	9 (6.3 to 12)	5.3 (3.6 to 7.3)	3.9 (2.8 to 5.2)	5.2 (3.7 to 7)	2.9 (1.9 to 4)	-44 (-56.7 to -28.8)	-42.2 (-55.8 to -25.5)	-45.6 (-60.2 to -26.9)
		DALYs	152.8 (109.9 to 202.7)	199.5 (144 to 264.6)	108 (73.6 to 147.5)	82.3 (58.4 to 109.2)	108.5 (77.8 to 142.9)	58.2 (39 to 81.7)	-46.1 (-58.5 to -31.3)	-45.6 (-58.3 to -29.6)	-46.1 (-60.7 to -27.5)
		YLLs	143.4 (102.1 to 191.7)	186.1 (132.3 to 250.4)	102.6 (68.4 to 141.7)	74 (51.5 to 100.4)	96.8 (66.4 to 129.3)	53.4 (35.2 to 76.7)	-48.4 (-61 to -32.9)	-48 (-61.2 to -31.3)	-48 (-62.5 to -28.6)
		YLDs	9.4 (6.1 to 12.9)	13.4 (8.7 to 18.4)	5.4 (3.5 to 7.5)	8.3 (5.4 to 11.5)	11.8 (7.7 to 16.5)	4.9 (3.1 to 6.8)	-12.2 (-19.1 to -4.3)	-12.2 (-20 to -3.2)	-9.3 (-21.2 to 4.3)
	Smoking	Deaths	19.3 (15.3 to 24.2)	5.2 (3.5 to 7.3)	32.3 (24.9 to 41)	8.7 (6.6 to 10.9)	2.1 (1.5 to 2.9)	14.6 (10.9 to 18.8)	-55.2 (-65.8 to -41.3)	-59.3 (-72.6 to -37.1)	-54.8 (-65.9 to -39.7)
		DALYs	419.7 (330.2 to 523.2)	119.6 (82.8 to 166.5)	716.9 (550.6 to 911.1)	197.1 (156 to 245.9)	48.4 (34.7 to 64.9)	339.1 (263.4 to 429.8)	-53 (-63.5 to -39.8)	-59.5 (-72 to -38.8)	-52.7 (-63.6 to -37.5)
		YLLs	382.5 (295 to 487.2)	106.2 (73.6 to 149.4)	655.3 (495.7 to 855.2)	168.5 (127.5 to 216)	40 (27.9 to 54.2)	291 (216.4 to 379.7)	-55.9 (-66.8 to -41.5)	-62.3 (-74.6 to -41.2)	-55.6 (-67 to -39.9)
		YLDs	37.2 (27 to 47.9)	13.4 (8.8 to 19.1)	61.6 (44.9 to 79.3)	28.5 (20.4 to 36.3)	8.4 (5.5 to 12.3)	48.1 (34.2 to 61.7)	-23.3 (-31 to -14.8)	-37.2 (-54.2 to -11.7)	-21.9 (-29.9 to -13)
Bahrain	All risk factors	Deaths	88 (76.6 to 103.5)	93.2 (80.4 to 108.1)	82.6 (70 to 104.7)	44.8 (36.4 to 56.8)	49.6 (39.1 to 61.8)	40.5 (32.3 to 57)	-49.1 (-59.1 to -37.3)	-46.8 (-59 to -32.2)	-51 (-61.9 to -37.5)
		DALYs	1711.9 (1516.3 to 1952.6)	1890 (1659.5 to 2170.1)	1561.2 (1349.3 to 1877.8)	818.1 (689.8 to 989.9)	931.6 (777.1 to 1115.8)	733.3 (605.5 to 952.1)	-52.2 (-61 to -41.9)	-50.7 (-60.1 to -38.9)	-53 (-62.3 to -41.9)
		YLLs	1506.4 (1313.9 to 1759.1)	1629.7 (1406.6 to 1904.3)	1402.5 (1194.1 to 1720.9)	666.6 (544.8 to 843.8)	739.4 (597.4 to 910.9)	609.6 (488.6 to 839.3)	-55.7 (-65 to -44.4)	-54.6 (-65 to -41.3)	-56.5 (-66.4 to -44.6)
		YLDs	205.6 (148.9 to 260.9)	260.2 (186.6 to 329.6)	158.7 (114.6 to 203.1)	151.4 (109.5 to 192.5)	192.1 (138 to 243.8)	123.7 (90.7 to 156.9)	-26.3 (-30.5 to -22)	-26.2 (-31.8 to -20.5)	-22 (-28 to -15.5)
	Alcohol use	Deaths	1.8 (1.2 to 2.5)	0.5 (0 to 1.1)	3.1 (2 to 4.3)	0.2 (0 to 0.4)	-0.1 (-0.2 to 0.1)	0.4 (0.1 to 0.7)	-90.6 (-100.3 to -80.6)	-114.5 (-257.4 to -89.5)	-87.9 (-96.2 to -78.6)

Location	Risk factor	Measure	1990 Both	1990 Female	1990 Male	2019 Both	2019 Female	2019 Male	% Change (1990 to 2019) Both	% Change (1990 to 2019) Female	% Change (1990 to 2019) Male
		DALYs	50.2 (34.5 to 66.5)	16 (4.5 to 30.1)	77.9 (52.2 to 106.8)	6.7 (2.2 to 11.9)	-0.8 (-3.4 to 2.5)	11.5 (4.9 to 19.6)	-86.7 (-95 to -77.4)	-104.8 (-155.4 to -89)	-85.3 (-93.3 to -76.3)
		YLLs	45.8 (31.4 to 61.1)	15 (4.6 to 27.8)	71.2 (47.8 to 98)	5.6 (2 to 9.9)	-0.4 (-2.5 to 2.3)	9.5 (4 to 16.2)	-87.8 (-95.2 to -79.1)	-102.4 (-140.3 to -89.1)	-86.6 (-93.9 to -78.1)
		YLDs	4.3 (2.4 to 6.3)	1 (-0.3 to 2.7)	6.8 (3.8 to 10.1)	1.1 (0.2 to 2.2)	-0.4 (-1.1 to 0.2)	2 (0.6 to 3.6)	-75.3 (-95.3 to -59.3)	-140.6 (-674.6 to 475.4)	-71.1 (-87.6 to -57.2)
	Ambient particulate matter pollution	Deaths	28.5 (22.3 to 35.8)	29.8 (23.2 to 37)	27.3 (21.3 to 36)	14.6 (11.4 to 19.3)	16.1 (12.4 to 20.9)	13.2 (10.2 to 18.9)	-48.8 (-60.1 to -33.9)	-45.9 (-59.2 to -28.3)	-51.5 (-63.1 to -37.6)
		DALYs	618.1 (493.5 to 755)	673.8 (529.5 to 831.2)	574.6 (457.6 to 725)	304.4 (241.6 to 382)	347.9 (278.6 to 436.5)	274 (216.8 to 360.3)	-50.8 (-60.9 to -37.8)	-48.4 (-59.6 to -33.4)	-52.3 (-62.9 to -39.3)
		YLLs	539.4 (426 to 664.5)	575.4 (447.4 to 716.1)	511.9 (401.8 to 653.9)	242.3 (188.2 to 317.7)	268.4 (206.9 to 348.9)	223 (172.1 to 307.7)	-55.1 (-65.7 to -41.1)	-53.3 (-65.4 to -37.8)	-56.4 (-67.3 to -42.6)
		YLDs	78.7 (54.3 to 104.1)	98.4 (67.3 to 130.7)	62.7 (43.2 to 83.3)	62.2 (44.8 to 81.4)	79.5 (56.9 to 104.1)	51.1 (36.8 to 66.2)	-21 (-29.2 to -9.5)	-19.2 (-29.3 to -5.7)	-18.6 (-27.7 to -6.4)
	Diet high in red meat	Deaths	3.8 (1.6 to 5.8)	4 (1.7 to 6.3)	3.6 (1.5 to 5.6)	1.8 (0.7 to 2.8)	1.9 (0.8 to 3.1)	1.6 (0.6 to 2.6)	-53.6 (-66.4 to -36.3)	-51.8 (-67.5 to -29.6)	-55.1 (-69.1 to -33.6)
		DALYs	91.5 (39.7 to 137.6)	101.4 (42.9 to 154.9)	84.1 (36.9 to 128.2)	42 (17.3 to 65.3)	47.6 (19.7 to 74.8)	38.4 (15.7 to 60.6)	-54.1 (-65.9 to -38.7)	-53.1 (-66.7 to -33.6)	-54.4 (-67.5 to -35.1)
		YLLs	78.9 (34.1 to 120.1)	85.4 (35.4 to 132.6)	74 (31.9 to 114.1)	32.2 (13.4 to 51.4)	35 (14.2 to 56.6)	30.2 (12.4 to 48.4)	-59.1 (-70.4 to -43.5)	-59 (-72 to -38.9)	-59.1 (-71.5 to -40.3)
		YLDs	12.6 (4.7 to 20.4)	16 (6.1 to 25.9)	10.1 (3.6 to 16.6)	9.8 (3.7 to 15.6)	12.6 (4.6 to 20.6)	8.1 (3 to 12.9)	-22.4 (-36 to -6.8)	-21.4 (-40.4 to 0.2)	-19.4 (-37.9 to 5.5)
	Diet high in sodium	Deaths	1.6 (0.3 to 6.5)	1.2 (0.3 to 5.1)	2 (0.2 to 8.2)	0.8 (0.1 to 3.1)	0.6 (0.2 to 2.5)	0.9 (0.1 to 3.7)	-52.6 (-71 to -16)	-51.5 (-75.3 to -0.3)	-54.3 (-75.7 to 8.5)
		DALYs	36.6 (4.8 to 147.4)	28.3 (5.3 to 119)	43.7 (3.6 to 175.3)	17.1 (2.3 to 68.4)	13.2 (2.7 to 56.7)	19.9 (1.7 to 76.7)	-53.3 (-72.3 to -19.7)	-53.5 (-77.7 to 1)	-54.6 (-72.8 to -5.2)
		YLLs	32.2 (4.2 to 130)	24.3 (4.6 to 102.6)	39 (3.3 to 158)	13.7 (1.9 to 56)	10.3 (2.2 to 44.7)	16.2 (1.4 to 63.3)	-57.5 (-75.3 to -24.8)	-57.8 (-79.9 to -6.7)	-58.6 (-75.5 to -10.7)
		YLDs	4.4 (0.5 to 17.9)	4 (0.7 to 16.7)	4.7 (0.3 to 18.6)	3.4 (0.4 to 13.5)	2.9 (0.5 to 12.5)	3.7 (0.3 to 14.1)	-22.8 (-56.1 to 28.9)	-27.9 (-64.7 to 58.5)	-20.8 (-47 to 49)
	Diet low in fiber	Deaths	0.9 (0.3 to 2.1)	0.9 (0.3 to 2.3)	0.9 (0.3 to 2.1)	0.4 (0.1 to 0.8)	0.4 (0.1 to 0.9)	0.3 (0.1 to 0.8)	-61.5 (-76.1 to -38.6)	-59.7 (-78.7 to -22)	-63.3 (-80.4 to -29.7)
		DALYs	18.9 (5.3 to 42.7)	20 (5.7 to 47.2)	18 (4.8 to 41.4)	6.9 (2.3 to 15.3)	7.5 (2.5 to 18.1)	6.4 (2.1 to 14.6)	-63.5 (-76.4 to -43.7)	-62.6 (-78.3 to -34.6)	-64.3 (-79.5 to -37.1)
		YLLs	16.2 (4.6 to 37)	16.8 (4.9 to 40.6)	15.9 (4.3 to 36.4)	5.4 (1.9 to 12.2)	5.6 (1.9 to 13.4)	5.2 (1.7 to 12)	-66.8 (-79.1 to -47.8)	-66.5 (-81.3 to -39)	-67.3 (-81.8 to -41.4)
		YLDs	2.6 (0.7 to 6)	3.2 (0.8 to 7.7)	2.2 (0.5 to 5.1)	1.5 (0.4 to 3.4)	1.9 (0.5 to 4.6)	1.2 (0.4 to 2.9)	-43.4 (-63.2 to -15.2)	-42.6 (-68.5 to -0.6)	-42.1 (-66.9 to -0.2)
	Diet low in fruits	Deaths	2.3 (1.1 to 3.8)	2.5 (1.1 to 4.4)	2.1 (0.9 to 3.7)	0.9 (0.4 to 1.6)	1.1 (0.5 to 1.9)	0.8 (0.3 to 1.6)	-60.3 (-75.6 to -36.1)	-57.8 (-78.1 to -25.1)	-62.5 (-80 to -27.9)
		DALYs	54.4 (27.8 to 89.1)	62.6 (29.5 to 108.4)	48.3 (22.6 to 82.8)	21.5 (10 to 36.1)	25.8 (11.7 to 45.2)	18.6 (8.4 to 33.1)	-60.5 (-73.4 to -42)	-58.7 (-75.4 to -32)	-61.5 (-77 to -35.1)
		YLLs	47.4 (24.3 to 77.1)	53.3 (25.5 to 91.7)	42.9 (19.6 to 74.4)	16.7 (8 to 28.5)	19.3 (8.9 to 33.9)	14.9 (6.9 to 26.4)	-64.7 (-76.9 to -46.6)	-63.8 (-79.3 to -37.2)	-65.3 (-79.7 to -40.3)
		YLDs	7 (2.6 to 12.6)	9.3 (3.3 to 17.5)	5.4 (1.9 to 10.3)	4.7 (1.7 to 8.8)	6.5 (2.3 to 12.6)	3.7 (1.3 to 7.2)	-32.6 (-51.2 to -10.8)	-30 (-55.6 to 7.5)	-31.8 (-58.1 to 4.9)
	Diet low in vegetables	Deaths	1.1 (0.3 to 2.3)	1.1 (0.3 to 2.3)	1.1 (0.3 to 2.4)	0.4 (0.1 to 0.7)	0.4 (0.1 to 0.8)	0.4 (0.1 to 0.8)	-67.1 (-80.2 to -48.3)	-66.3 (-82.4 to -41.1)	-67.9 (-82.5 to -43.7)
		DALYs	22.2 (6.2 to 48.1)	23 (6.1 to 50.3)	21.7 (5.6 to 48.3)	6.6 (2.1 to 14.4)	6.8 (2.4 to 15.2)	6.4 (2 to 14.6)	-70.2 (-81.3 to -53.2)	-70.2 (-83.4 to -47.5)	-70.4 (-83 to -48.9)
		YLLs	19.4 (5.3 to 43.4)	19.6 (5.3 to 44.4)	19.4 (5 to 44.5)	5.3 (1.8 to 11.8)	5.4 (1.9 to 12)	5.3 (1.7 to 12.4)	-72.5 (-83 to -55)	-72.7 (-85.3 to -50.6)	-72.6 (-84.6 to -51.9)
		YLDs	2.8 (0.7 to 5.9)	3.4 (0.8 to 7.3)	2.3 (0.5 to 4.9)	1.3 (0.4 to 2.8)	1.5 (0.4 to 3.4)	1.1 (0.3 to 2.6)	-54.3 (-69.9 to -32.3)	-55.8 (-75.2 to -28)	-51.5 (-72.2 to -19.7)
	Diet low in whole grains	Deaths	3.9 (1.7 to 5.4)	4.2 (1.9 to 5.9)	3.5 (1.5 to 4.9)	2 (0.8 to 2.9)	2.1 (0.8 to 3.2)	1.8 (0.7 to 2.8)	-48.6 (-60.4 to -35.7)	-48.9 (-64.1 to -33.8)	-47.3 (-60.9 to -31.2)
		DALYs	74.3 (32.2 to 104.6)	84 (36.6 to 118.7)	65.2 (27.5 to 92.7)	37.7 (14 to 55.6)	42.2 (14.7 to 61.9)	34.2 (12.9 to 51.3)	-49.3 (-60.7 to -39)	-49.7 (-62.6 to -38.4)	-47.6 (-59.7 to -34.9)
		YLLs	59.4 (25.5 to 84)	65.1 (28.8 to 92.5)	53.7 (22.6 to 76.5)	27.2 (10.2 to 41)	29 (10.4 to 43.7)	25.5 (10.1 to 39.2)	-54.2 (-65.6 to -42.1)	-55.5 (-68.3 to -41.8)	-52.5 (-65.1 to -37.5)
		YLDs	14.9 (6 to 22.6)	19 (7.5 to 29.3)	11.5 (4.7 to 17.8)	10.5 (3.5 to 16.3)	13.3 (4.3 to 20.7)	8.7 (3.1 to 13.9)	-29.6 (-40.7 to -23.7)	-30 (-43.1 to -22.4)	-24.5 (-36 to -15.6)
	High body-mass index	Deaths	22 (13.7 to 31.1)	26.7 (17.4 to 36.3)	17.9 (10.3 to 26.7)	11 (6.6 to 16)	13.2 (8.3 to 19.4)	9.2 (5.2 to 13.7)	-50.2 (-62.1 to -35.4)	-50.4 (-62.8 to -34.3)	-48.6 (-61.9 to -30)
		DALYs	630.8 (422 to 845.7)	787.8 (556 to 1028.9)	508 (319.6 to 708.4)	317.5 (218.2 to 428.4)	389.4 (277.1 to 525.2)	270.6 (179.6 to 376.2)	-49.7 (-59.4 to -36.6)	-50.6 (-61.1 to -36.7)	-46.7 (-58.1 to -29.1)
		YLLs	546.8 (362.9 to 732.3)	671.3 (468 to 881)	448.3 (276.2 to 629.4)	244 (160.7 to 341.1)	289.9 (194.1 to 400.5)	212.9 (135 to 302.9)	-55.4 (-65.5 to -40.9)	-56.8 (-67.5 to -42.3)	-52.5 (-64 to -35)
		YLDs	83.9 (52.3 to 120.6)	116.5 (75.3 to 162.4)	59.7 (35.5 to 88.9)	73.5 (46.7 to 101.7)	99.6 (65.9 to 135.3)	57.8 (36.5 to 82.1)	-12.4 (-20 to -2.3)	-14.5 (-22.9 to -4.7)	-3.2 (-14.9 to 14.1)
	High fasting plasma glucose	Deaths	33 (19.5 to 58.1)	34.4 (19.5 to 58.6)	31.7 (18.6 to 57.4)	16 (8.9 to 30.9)	17.6 (9.7 to 33.7)	14.5 (7.8 to 28.6)	-51.7 (-64.8 to -37.4)	-48.8 (-64.3 to -30.3)	-54.3 (-68.6 to -38)
		DALYs	619 (394.5 to 974.1)	663.2 (407.3 to 1024.6)	580 (370 to 931.6)	289.5 (179.9 to 474.6)	328 (200.5 to 533.9)	261 (156.5 to 444.4)	-53.2 (-64.3 to -40.1)	-50.5 (-63.5 to -33.9)	-55 (-67.6 to -39.8)
		YLLs	548.7 (349.3 to 861.8)	576.6 (353.9 to 889.7)	525 (334.5 to 838.4)	237.2 (145.7 to 399.8)	263.5 (157 to 445)	217.4 (126.7 to 387.4)	-56.8 (-68 to -43.2)	-54.3 (-67.7 to -36.3)	-58.6 (-71.4 to -42.8)
		YLDs	70.2 (38 to 117.5)	86.6 (46.1 to 145.3)	55 (30.4 to 92.3)	52.3 (30.5 to 82)	64.6 (36.4 to 104.3)	43.6 (25.1 to 69.3)	-25.5 (-37.6 to -9.5)	-25.5 (-40.8 to -3.2)	-20.6 (-36.9 to -1.8)
	High LDL cholesterol	Deaths	13.9 (4.4 to 29.7)	15.6 (5.1 to 33.5)	11.8 (3.7 to 25.6)	7.8 (2 to 18.6)	8.9 (2.2 to 20.7)	6.7 (1.7 to 16.2)	-43.9 (-59.2 to -30.7)	-43 (-60.8 to -26)	-43.6 (-59 to -27.9)
		DALYs	265.7 (134.5 to 466.2)	311.2 (159.6 to 541.5)	222.7 (111.7 to 392.3)	145.6 (68.1 to 271.2)	171.5 (80.8 to 315.9)	125.2 (58.9 to 236.7)	-45.2 (-55.6 to -34)	-44.9 (-56.3 to -32.9)	-43.8 (-55.3 to -31)
		YLLs	210.3 (96.9 to 388.2)	238.1 (112.2 to 444.8)	181.5 (82.1 to 332.9)	103.7 (39.1 to 214.2)	115.9 (43.5 to 240.2)	91.9 (35.4 to 191.3)	-50.7 (-64.4 to -37.4)	-51.3 (-66.8 to -35.1)	-49.4 (-63.9 to -34.2)
		YLDs	55.5 (32.4 to 90.5)	73.1 (42.3 to 120.2)	41.3 (24.2 to 65.4)	41.9 (25.3 to 66.8)	55.6 (33.8 to 87.3)	33.3 (20.1 to 52.9)	-24.5 (-30.7 to -17.2)	-23.9 (-31.4 to -15)	-19.3 (-27.8 to -8.5)
	High systolic blood pressure	Deaths	51.1 (40.1 to 65.3)	56.3 (42.6 to 71.7)	45.5 (34.3 to 62.7)	25.6 (18.5 to 35.4)	29.3 (20.8 to 40.4)	22.3 (15.8 to 32.3)	-49.8 (-61.7 to -34.9)	-48 (-62.1 to -27.8)	-50.9 (-63.1 to -35.7)
		DALYs	1033.5 (835.8 to 1274.3)	1175.2 (916.1 to 1456.9)	907.4 (712.3 to 1175.2)	490.3 (383.5 to 626.8)	570.3 (435.5 to 728.6)	429.4 (326.5 to 570.9)	-52.6 (-62.5 to -40.1)	-51.5 (-63.4 to -34.2)	-52.7 (-63.4 to -39.4)
		YLLs	910.1 (731.1 to 1139.8)	1016.3 (785.7 to 1274.4)	814.9 (624.2 to 1073.2)	398.4 (303.2 to 528.5)	453.1 (337 to 600.4)	354.7 (264.2 to 489.1)	-56.2 (-66.7 to -42.8)	-55.4 (-67.5 to -37.1)	-56.5 (-67.5 to -42.4)
		YLDs	123.4 (85.8 to 163.6)	159 (109 to 212)	92.5 (63.4 to 123.5)	91.9 (65.5 to 120.8)	117.2 (80.6 to 155.8)	74.6 (51.7 to 98.2)	-25.5 (-33.8 to -14.6)	-26.3 (-38.4 to -10.6)	-19.3 (-29.9 to -5.3)
	High temperature	Deaths	3.9 (0.1 to 7.7)	4.1 (0.1 to 8.2)	3.7 (0.1 to 7.5)	2.3 (0.4 to 4.5)	2.5 (0.4 to 4.9)	2.1 (0.3 to 3.9)	-41.4 (-67.7 to -21.7)	-38.7 (-66.2 to -15.8)	-43.9 (-67.6 to -22.2)
		YLLs	66.9 (2.3 to 130.9)	72.2 (2.5 to 143.6)	62.5 (2.1 to 123.5)	33.4 (5.3 to 64)	37 (5.8 to 70.9)	30.5 (4.9 to 59.1)	-50.1 (-72.5 to -33.5)	-48.7 (-71.6 to -28.6)	-51.1 (-72.6 to -32.5)

Location	Risk factor	Measure	1990			2019			% Change (1990 to 2019)		
			Both	Female	Male	Both	Female	Male	Both	Female	Male
	Household air pollution from solid fuels	Deaths	2.7 (1.1 to 5.1)	3.3 (1.4 to 6.3)	2 (0.8 to 4.2)	0 (0 to 0.1)	0.1 (0 to 0.1)	0 (0 to 0.1)	-98.4 (-99.4 to -96.4)	-98.3 (-99.4 to -96.1)	-98.5 (-99.4 to -96.4)
		DALYs	57.7 (24.3 to 111)	75.8 (31.7 to 141.2)	42.5 (15.9 to 88.5)	0.9 (0.3 to 2.1)	1.2 (0.4 to 3.1)	0.6 (0.2 to 1.6)	-98.5 (-99.4 to -96.4)	-98.4 (-99.4 to -96.3)	-98.6 (-99.4 to -96.6)
		YLLs	50.3 (20.8 to 97)	64.7 (26.9 to 123.4)	37.9 (14.3 to 79.7)	0.7 (0.2 to 1.7)	0.9 (0.3 to 2.4)	0.5 (0.1 to 1.3)	-98.6 (-99.5 to -96.8)	-98.6 (-99.5 to -96.6)	-98.7 (-99.5 to -96.8)
		YLDs	7.5 (3 to 14)	11 (4.4 to 20.5)	4.6 (1.7 to 9.7)	0.2 (0.1 to 0.4)	0.3 (0.1 to 0.7)	0.1 (0 to 0.3)	-97.7 (-99.1 to -94.6)	-97.5 (-99 to -94.3)	-97.5 (-99 to -94.3)
	Kidney dysfunction	Deaths	8.6 (5.9 to 11.6)	9.9 (6.5 to 13.2)	7.4 (5.1 to 10.2)	5.8 (3.3 to 8.6)	6.5 (3.7 to 9.5)	5.2 (3 to 7.8)	-33 (-50.7 to -14.4)	-34.3 (-53.9 to -13.7)	-29.9 (-50.6 to -6.2)
		DALYs	173.3 (133.1 to 218)	210.2 (160.2 to 265.1)	141.6 (107.7 to 182.7)	111.6 (78.4 to 152.8)	132.8 (93.7 to 177.4)	96.7 (66.9 to 134.9)	-35.6 (-49.2 to -20.8)	-36.8 (-50.8 to -19.9)	-31.7 (-48 to -12.4)
		YLLs	150.9 (114.2 to 191)	179.2 (133.4 to 227.4)	126.4 (96.1 to 165.9)	90.2 (61 to 125.6)	104.4 (69.5 to 143.1)	79.9 (52.7 to 117.1)	-40.2 (-54.7 to -23.4)	-41.7 (-57.3 to -21.8)	-36.8 (-54.1 to -16)
		YLDs	22.4 (15.2 to 30.4)	31 (20.9 to 42.3)	15.1 (10.2 to 20.4)	21.4 (14.4 to 29)	28.4 (18.8 to 38.6)	16.8 (11.2 to 22.5)	-4.3 (-11.6 to 3.7)	-8.4 (-16.9 to 0.4)	10.8 (-0.6 to 22.7)
	Lead exposure	Deaths	2.6 (0.9 to 4.4)	2.3 (0.6 to 4.3)	2.9 (1.2 to 4.9)	1.2 (0.4 to 2.3)	1.1 (0.3 to 2.2)	1.3 (0.5 to 2.4)	-52.3 (-62.7 to -39.3)	-50.8 (-63.6 to -33.2)	-53.8 (-64.9 to -39.3)
		DALYs	52.9 (17.5 to 92.6)	47.4 (10.3 to 91.1)	57.8 (23.5 to 97.1)	20.3 (6.2 to 38.2)	18.5 (3.5 to 38.2)	21.8 (8 to 39.2)	-61.6 (-70.9 to -52.2)	-60.9 (-73.1 to -48.9)	-62.3 (-71.7 to -52.3)
		YLLs	46.9 (15.7 to 82.1)	41.2 (9 to 78.5)	52 (21.1 to 88.7)	17.1 (5.4 to 31.8)	15.3 (3.1 to 31.3)	18.6 (7 to 33)	-63.6 (-72.7 to -53.4)	-62.7 (-74.3 to -49)	-64.3 (-73.5 to -53.4)
		YLDs	6 (1.7 to 11.3)	6.2 (1.2 to 12.7)	5.7 (1.9 to 10.1)	3.2 (0.7 to 6.6)	3.2 (0.4 to 7.3)	3.2 (0.9 to 6.3)	-46.2 (-59.7 to -38.3)	-48.5 (-70.3 to -38.9)	-43.9 (-58.2 to -34.1)
	Low physical activity	Deaths	6.3 (1.5 to 13)	6.8 (1.7 to 13.7)	5.6 (1.2 to 11.7)	4 (1 to 8)	4.5 (1.1 to 8.9)	3.6 (0.7 to 7)	-36 (-49.2 to -17.9)	-34 (-49 to -10.1)	-36.9 (-52.3 to -14.2)
		DALYs	91.4 (20.7 to 194.3)	102.6 (23.6 to 219.6)	79.8 (16.2 to 173.6)	56.4 (12.5 to 117.5)	64.7 (15.1 to 130.7)	49.2 (9.4 to 103.2)	-38.3 (-49.3 to -22.7)	-37 (-49.3 to -16.9)	-38.4 (-51.9 to -19)
		YLLs	77.6 (17.4 to 165.1)	84.9 (19.9 to 179.1)	69.7 (14.3 to 151.6)	45.5 (10.1 to 91.9)	50.6 (12.2 to 101.9)	40.6 (7.9 to 83)	-41.4 (-53.8 to -23.2)	-40.4 (-54.3 to -16.9)	-41.8 (-56 to -20.5)
		YLDs	13.9 (2.7 to 32.7)	17.7 (3.8 to 41.3)	10.2 (1.8 to 24.8)	11 (2 to 25.9)	14.1 (2.8 to 33)	8.7 (1.4 to 20.7)	-20.9 (-29.2 to -11.6)	-20.6 (-30.7 to -8.2)	-15 (-25.3 to -1.7)
	Low temperature	Deaths	3 (-2.3 to 7.9)	3.2 (-2.4 to 8.4)	2.8 (-2.2 to 7.6)	1.5 (-1 to 4)	1.7 (-1.1 to 4.4)	1.4 (-0.9 to 3.6)	-	-	-
		YLLs	51.1 (-39.5 to 135.2)	55 (-42.5 to 145.7)	47.7 (-37.2 to 128.1)	22.3 (-14.1 to 56.6)	24.7 (-16.1 to 63.9)	20.4 (-12.9 to 54.1)	-	-	-
	Secondhand smoke	Deaths	3.1 (2.3 to 4.1)	3.7 (2.7 to 4.8)	2.6 (1.9 to 3.6)	1.3 (0.9 to 1.9)	1.5 (1.1 to 2.1)	1.2 (0.8 to 1.7)	-57.6 (-67.2 to -44.5)	-58.7 (-69.5 to -44.1)	-55.2 (-66.9 to -38.5)
		DALYs	68.4 (50.4 to 88.5)	85.5 (61.6 to 111.7)	55.1 (39.6 to 73)	27.4 (19.4 to 37.8)	33.5 (23.7 to 45.4)	23.6 (16.2 to 34)	-60 (-68.5 to -48.1)	-60.8 (-69.9 to -48.1)	-57.2 (-67.7 to -42.5)
		YLLs	62.9 (46 to 82)	77.7 (56 to 101.8)	51.4 (36.9 to 68.2)	23.6 (16.6 to 33.4)	28.2 (19.7 to 38.5)	20.8 (14 to 30.5)	-62.5 (-71.2 to -49.8)	-63.7 (-73.3 to -50.6)	-59.6 (-70.6 to -44.4)
		YLDs	5.5 (3.6 to 7.6)	7.9 (5.1 to 10.9)	3.7 (2.4 to 5.2)	3.8 (2.5 to 5.2)	5.4 (3.5 to 7.4)	2.8 (1.9 to 4)	-31.3 (-38.6 to -23.6)	-32 (-40.4 to -22)	-23.2 (-34.4 to -8.8)
	Smoking	Deaths	12.6 (10.3 to 15.9)	9 (6.3 to 12.2)	16.5 (13.4 to 21.2)	4.3 (3.3 to 5.9)	2.5 (1.7 to 3.6)	5.9 (4.6 to 8.3)	-66 (-74.9 to -55.1)	-72 (-82.1 to -56.4)	-64.1 (-73.5 to -52.2)
		DALYs	311.2 (255.6 to 381.9)	233.1 (163 to 313.9)	379.6 (312.9 to 461.9)	105 (82.9 to 135.7)	63 (44.2 to 90.1)	135.6 (106.9 to 177.2)	-66.2 (-74.3 to -55.7)	-73 (-82.3 to -59.2)	-64.3 (-72.5 to -53.6)
		YLLs	272.9 (221.4 to 340.7)	200.1 (137.7 to 273.6)	337.5 (272.6 to 417.6)	83 (64.1 to 111.4)	48.5 (33.1 to 69.5)	109 (83.2 to 150.3)	-69.6 (-77.4 to -59.3)	-75.8 (-84.5 to -61.3)	-67.7 (-76.2 to -56.3)
		YLDs	38.4 (26.7 to 50)	33 (21.4 to 47.9)	42.1 (29.7 to 54.7)	22 (15 to 28.9)	14.5 (9 to 21.1)	26.6 (18.6 to 34.6)	-42.6 (-52.2 to -30.6)	-56.1 (-69.7 to -37)	-36.9 (-45.6 to -26.5)
Egypt	All risk factors	Deaths	79.8 (65.7 to 97.7)	83.9 (64.3 to 100.8)	75.7 (62.9 to 105)	72.4 (51.3 to 101.5)	89.8 (62.3 to 124.6)	63.6 (43.7 to 94.4)	-9.3 (-33.7 to 21.7)	7 (-21.9 to 45.7)	-16 (-39.9 to 15.4)
		DALYs	1933.1 (1656.1 to 2236.4)	1968.9 (1569.5 to 2270.5)	1892.7 (1614.3 to 2361.3)	1755 (1315 to 2369.7)	1975.6 (1464.3 to 2627.9)	1625.3 (1172.4 to 2310.1)	-9.2 (-30.9 to 17.3)	0.3 (-24.7 to 32.3)	-14.1 (-36.3 to 15)
		YLLs	1735.2 (1460.9 to 2028)	1733.6 (1342.3 to 2019.4)	1732.5 (1456.9 to 2173.8)	1505.2 (1079.4 to 2119.2)	1663.2 (1160.5 to 2331.8)	1425.9 (975.8 to 2109.1)	-13.3 (-36.9 to 15.9)	-4.1 (-30.8 to 31.8)	-17.7 (-41.7 to 14.5)
		YLDs	197.9 (143.9 to 255.2)	235.4 (169 to 305.7)	160.2 (115 to 207.1)	249.9 (181.2 to 317.1)	312.3 (227.5 to 397.2)	199.3 (143.2 to 255.1)	26.2 (18.6 to 33.8)	32.7 (22.7 to 43.7)	24.4 (15.3 to 34.6)
	Alcohol use	Deaths	0.1 (-0.1 to 0.4)	-0.1 (-0.2 to 0)	0.3 (0 to 0.9)	0 (-0.1 to 0.2)	-0.1 (-0.3 to 0)	0.2 (-0.1 to 0.6)	-69.3 (-538.7 to 272.6)	109.2 (-926.4 to 915.5)	-47.5 (-212.7 to 145.1)
		DALYs	5.3 (-0.8 to 13.9)	-1.1 (-3.5 to 1.8)	11.7 (0 to 27.6)	2.4 (-2.6 to 8.9)	-2.8 (-5.6 to -0.1)	6.8 (-2 to 18.6)	-55.1 (-397.6 to 223.5)	142 (-1274.3 to 1502.4)	-41.5 (-161.5 to 148.3)
		YLLs	5.3 (-0.4 to 13.5)	-0.7 (-2.9 to 2)	11.3 (0.3 to 26.5)	2.5 (-2 to 8.4)	-2.1 (-4.6 to 0.1)	6.5 (-1.4 to 17.1)	-53.3 (-280.2 to 168.9)	188.3 (-1063.3 to 1824.5)	-42.6 (-133.5 to 155.2)
		YLDs	0 (-0.5 to 0.6)	-0.4 (-0.7 to -0.1)	0.4 (-0.5 to 1.5)	-0.1 (-0.7 to 0.6)	-0.6 (-1.1 to -0.1)	0.4 (-0.7 to 1.6)	-695.4 (-1700.9 to 1118.4)	54.6 (1.7 to 146)	-13.6 (-509.4 to 945.6)
	Ambient particulate matter pollution	Deaths	28.5 (21.3 to 36.6)	28.8 (20.2 to 37.1)	28.2 (21.2 to 38.9)	27.9 (19.2 to 39.9)	33.3 (22.1 to 47.8)	24.9 (16.5 to 37.2)	-2.1 (-28.5 to 35.2)	15.8 (-18.5 to 63.2)	-11.6 (-37.8 to 24.7)
		DALYs	738.7 (565.9 to 920.7)	729.9 (527.2 to 929.8)	746 (575.8 to 986.3)	733.5 (527.3 to 1009)	811.4 (576.7 to 1118.3)	684.3 (478.7 to 974.4)	-0.7 (-24.8 to 32.4)	11.2 (-18.6 to 51)	-8.3 (-33 to 26.3)
		YLLs	659.7 (498.8 to 833)	637.9 (450.5 to 821)	679.9 (516.6 to 912.8)	625.2 (431.4 to 897.6)	676.8 (456.2 to 972.7)	597.8 (400 to 889)	-5.2 (-32.5 to 31.2)	6.1 (-26.1 to 49.7)	-12.1 (-38.4 to 25.8)
		YLDs	79 (55.4 to 107.7)	92 (62.9 to 125.7)	66.1 (45.4 to 90.1)	108.3 (75 to 141.6)	134.6 (94 to 175.6)	86.5 (59.6 to 113.7)	37 (23.5 to 60.3)	46.3 (28 to 77.4)	30.9 (17.7 to 51.6)
	Diet high in red meat	Deaths	2.8 (1 to 4.5)	2.9 (1 to 4.7)	2.7 (0.9 to 4.5)	2.9 (1 to 5.1)	3.3 (1.1 to 5.9)	2.7 (0.9 to 4.8)	4.1 (-38.3 to 67)	17 (-35.5 to 98.3)	-1.3 (-40.7 to 62.3)
		DALYs	83 (27.6 to 132.8)	82.6 (28.5 to 133.9)	83.3 (27.8 to 133.4)	89.6 (32.9 to 150.7)	94.7 (33.9 to 160.4)	86.9 (30.5 to 150.2)	8 (-33.1 to 78.2)	14.7 (-33.3 to 94.5)	4.3 (-34.5 to 75.7)
		YLLs	74.1 (24.5 to 119.5)	72 (23.7 to 117.5)	76.1 (24.6 to 122.6)	76.2 (27.7 to 132.1)	78.2 (27.4 to 138.4)	76.2 (27.1 to 134.2)	2.8 (-39.5 to 79.2)	8.7 (-39.5 to 94)	0.1 (-38.8 to 75.6)
		YLDs	8.9 (2.7 to 14.9)	10.6 (3.2 to 17.9)	7.2 (2.2 to 12)	13.4 (4.1 to 21.9)	16.5 (5 to 27.6)	10.7 (3.3 to 17.5)	50.8 (19.6 to 89.7)	55.8 (22 to 98.9)	49 (18 to 91.2)
	Diet high in sodium	Deaths	1.7 (0.2 to 6.9)	1.2 (0.3 to 5)	2.2 (0.2 to 8.8)	1.5 (0.2 to 6.1)	1.2 (0.3 to 5)	1.7 (0.1 to 7.2)	-13.5 (-51.7 to 53.8)	-3.7 (-56.8 to 98.9)	-20 (-57.9 to 65)
		DALYs	45.1 (5.3 to 181.4)	31.6 (5.6 to 130.5)	58.5 (4.2 to 232)	39.6 (4.7 to 160.3)	29.7 (5.7 to 124.3)	48.7 (3.3 to 195.5)	-12.2 (-50.7 to 56.9)	-6.1 (-57.9 to 114.9)	-16.8 (-53.1 to 72)
		YLLs	40.7 (4.7 to 163.6)	27.8 (4.8 to 115.2)	53.4 (3.8 to 212.2)	34.2 (3.9 to 140.8)	24.9 (4.6 to 106.6)	42.6 (2.9 to 173.7)	-16 (-53.5 to 53.9)	-10.5 (-60.3 to 108.1)	-20.2 (-56.2 to 66.4)
		YLDs	4.4 (0.5 to 17.9)	3.8 (0.7 to 16)	5 (0.3 to 20.1)	5.5 (0.7 to 21.4)	4.8 (0.9 to 20.2)	6 (0.4 to 22.6)	23.3 (-29.5 to 110.9)	26.3 (-40.2 to 173.6)	19.9 (-20.1 to 144.2)
	Diet low in fiber	Deaths	0.7 (0.2 to 1.5)	0.7 (0.2 to 1.5)	0.7 (0.2 to 1.6)	0.3 (0.2 to 0.6)	0.4 (0.2 to 0.7)	0.3 (0.1 to 0.6)	-55 (-72.1 to -18.9)	-45.9 (-70.2 to 3.5)	-58.6 (-74.6 to -25.1)
		DALYs	16.9 (5.4 to 39.5)	15.6 (5.4 to 34.3)	18.2 (5.3 to 45.4)	7.5 (3.9 to 15.4)	7.7 (4.3 to 15.5)	7.6 (3.5 to 17.2)	-55.6 (-70.2 to -21.1)	-50.7 (-71.2 to -8.5)	-57.9 (-72.9 to -26.2)
		YLLs	15.2 (4.8 to 35.7)	13.6 (4.7 to 30.2)	16.7 (4.8 to 41.3)	6.4 (3.2 to 13.6)	6.4 (3.4 to 13.2)	6.7 (3 to 15.5)	-57.5 (-72.6 to -23.5)	-52.7 (-73.5 to -10)	-59.6 (-74.9 to -27.3)

Location	Risk factor	Measure	1990			2019			% Change (1990 to 2019)		
			Both	Female	Male	Both	Female	Male	Both	Female	Male
		YLDs	1.7 (0.6 to 3.9)	2 (0.7 to 4.5)	1.5 (0.5 to 3.4)	1.1 (0.6 to 2.1)	1.2 (0.7 to 2.5)	0.9 (0.4 to 2)	-38.5 (-53.7 to 5.5)	-36.3 (-56.9 to 12.1)	-38.9 (-56.2 to 3.8)
	Diet low in fruits	Deaths	4.7 (2.1 to 8.3)	4.9 (2.2 to 8.8)	4.5 (2 to 7.9)	1.8 (0.8 to 3.2)	2.2 (0.9 to 4.1)	1.5 (0.6 to 2.9)	-62.5 (-78.1 to -38.9)	-55.6 (-76.2 to -23.6)	-65.5 (-80.4 to -41.8)
		DALYs	134 (61.8 to 232.5)	136 (63.7 to 240.1)	131.9 (60.5 to 233.3)	54.3 (24 to 97)	59.8 (24.8 to 108.2)	51 (22.1 to 95.8)	-59.5 (-75.1 to -34.6)	-56 (-75.8 to -26.1)	-61.3 (-77.6 to -37.4)
		YLLs	121.1 (56.3 to 213.7)	120.3 (56.6 to 214.7)	121.6 (55.7 to 219.4)	46.7 (20.6 to 86.5)	50 (21 to 90.6)	45.4 (19.5 to 87.3)	-61.4 (-76.7 to -36.9)	-58.4 (-77.4 to -26.9)	-62.7 (-78.9 to -38.4)
		YLDs	13 (4.8 to 23.9)	15.7 (5.8 to 28.7)	10.3 (3.8 to 18.9)	7.6 (2.6 to 13.9)	9.8 (3.3 to 18.3)	5.6 (1.9 to 10.6)	-41.7 (-60.5 to -19)	-37.5 (-59.5 to -11)	-45.3 (-64.7 to -19.2)
	Diet low in vegetables	Deaths	0.5 (0.2 to 1)	0.4 (0.2 to 0.9)	0.5 (0.2 to 1.1)	0.2 (0.1 to 0.3)	0.2 (0.2 to 0.3)	0.2 (0.1 to 0.2)	-60.6 (-81 to -15.1)	-49 (-76.8 to 8.6)	-66.7 (-85.2 to -20.4)
		DALYs	9 (4.3 to 20.5)	8.2 (4.3 to 16.6)	9.8 (4.1 to 23.7)	4 (3 to 5.3)	4.6 (3.3 to 6.1)	3.6 (2.6 to 5.1)	-56.1 (-80.7 to -8.4)	-44.5 (-74.6 to 6.8)	-63.3 (-85 to -14.5)
		YLLs	8.1 (3.8 to 18.9)	7.3 (3.7 to 15.3)	9 (3.7 to 22.2)	3.4 (2.4 to 4.8)	3.8 (2.6 to 5.4)	3.1 (2.2 to 4.7)	-58.3 (-82.1 to -10.8)	-47 (-76.3 to 6.1)	-65 (-86.1 to -16.8)
		YLDs	0.9 (0.4 to 1.9)	1 (0.5 to 1.9)	0.8 (0.3 to 1.9)	0.6 (0.4 to 0.7)	0.7 (0.5 to 0.9)	0.5 (0.3 to 0.6)	-36.3 (-67.5 to 16.9)	-25.8 (-61.2 to 24.6)	-44.8 (-72.4 to 16)
	Diet low in whole grains	Deaths	2.3 (0.6 to 3.6)	2.3 (0.6 to 3.8)	2.2 (0.5 to 3.8)	2.7 (0.7 to 4.7)	3.3 (0.8 to 5.8)	2.4 (0.6 to 4.3)	19.7 (-15.3 to 58.6)	40 (-2.8 to 90)	11.9 (-24.7 to 54.6)
		DALYs	54.9 (13.5 to 85.6)	56.1 (13.9 to 90.3)	53.7 (12.8 to 89.4)	71.7 (17.5 to 120.5)	79.6 (18.5 to 136.7)	67 (15.9 to 115.6)	30.4 (-7.1 to 66.8)	41.9 (4.7 to 84.4)	24.7 (-15.7 to 66.5)
		YLLs	44 (10.8 to 71.4)	43.2 (10.7 to 71.3)	44.8 (10.3 to 79.5)	57.9 (14.1 to 102)	62.7 (14.7 to 114.8)	55.9 (12.9 to 100.7)	31.5 (-12.4 to 76.5)	45.2 (-0.6 to 103.6)	24.8 (-20.2 to 74.3)
		YLDs	10.9 (2.7 to 17.8)	12.9 (3.1 to 21.1)	8.9 (2.2 to 14.7)	13.7 (3.3 to 22.3)	16.9 (4 to 27.5)	11.1 (2.6 to 18.2)	25.9 (13.1 to 35.7)	30.9 (15.8 to 44.7)	24.3 (10.7 to 37.3)
	High body-mass index	Deaths	23.2 (14.1 to 33.5)	27.7 (17.2 to 38.4)	18.8 (10.1 to 29.5)	25 (14.9 to 38.6)	30.2 (18.4 to 47.1)	21.4 (12.1 to 35.4)	7.7 (-25.1 to 51.4)	9.1 (-25 to 56.2)	14.2 (-22.7 to 74.7)
		DALYs	775.3 (497 to 1071.6)	904 (597.3 to 1197.4)	647.4 (367.6 to 962.4)	829 (545.9 to 1208.4)	935.9 (619.5 to 1346.2)	748.6 (464.8 to 1145.5)	6.9 (-21.9 to 44.8)	3.5 (-24.6 to 41.1)	15.6 (-19.7 to 75.3)
		YLLs	696.5 (442.5 to 972.7)	796.8 (509.1 to 1061)	596.6 (338.1 to 892.1)	709.3 (449.5 to 1066.8)	777.4 (491.4 to 1169)	662.5 (401.1 to 1050.3)	1.8 (-28.9 to 43.3)	-2.4 (-32.6 to 39.3)	11 (-25.5 to 73.6)
		YLDs	78.8 (47.7 to 117)	107.2 (67.5 to 152)	50.8 (26.9 to 81)	119.7 (77.4 to 166.8)	158.5 (103.8 to 217.1)	86.2 (53 to 124.2)	51.9 (35.8 to 78.3)	47.8 (32.4 to 72.2)	69.5 (41 to 131.8)
	High fasting plasma glucose	Deaths	11.5 (6.5 to 21.2)	14.3 (7.8 to 28)	8.4 (5.1 to 14.5)	23.8 (12.2 to 44.2)	31.9 (15 to 61.8)	19 (9 to 37.6)	107.1 (31.9 to 205.1)	122.7 (35.8 to 245.5)	125.3 (36.7 to 245.5)
		DALYs	226.6 (144.1 to 365.4)	270.6 (165.7 to 462.3)	179.6 (114.3 to 285.5)	526.7 (295.2 to 920.9)	658.5 (352.8 to 1142)	434.9 (216.8 to 799.6)	132.5 (50.4 to 233.2)	143.4 (50.8 to 262.6)	142.2 (47.5 to 268.2)
		YLLs	202 (126.8 to 329)	238 (144 to 410.2)	163.2 (103.3 to 260.3)	449.6 (244.1 to 799.4)	556.1 (284.4 to 987.1)	377.7 (185.1 to 715.4)	122.6 (40.8 to 227.6)	133.7 (38.7 to 256.4)	131.4 (35.2 to 262.4)
		YLDs	24.6 (13.7 to 41.3)	32.6 (17.5 to 57.7)	16.3 (9.2 to 27.3)	77.1 (40.7 to 131.3)	102.4 (53.2 to 179.3)	57.1 (28.3 to 99)	213.1 (133.5 to 312.2)	214.2 (125.5 to 319.5)	249.9 (137.2 to 383.1)
	High LDL cholesterol	Deaths	9.3 (3.7 to 19.2)	10.3 (3.8 to 21.8)	8.2 (3.4 to 18)	12.2 (5.3 to 23.6)	16.1 (6 to 33.3)	10.3 (4.8 to 20.7)	32.1 (-4 to 85.1)	56.5 (10.7 to 117.9)	25.2 (-13.1 to 88.2)
		DALYs	222.5 (126.2 to 381.7)	241.6 (128.2 to 432.2)	202.9 (118.2 to 382.7)	325.4 (192.5 to 531.1)	384.5 (213.3 to 669.6)	287.1 (170.6 to 491.4)	46.2 (9.2 to 91)	59.1 (18.5 to 108.3)	41.5 (-0.7 to 98.5)
		YLLs	177.1 (96.4 to 318.3)	185 (87.1 to 344.7)	168.7 (94.3 to 319.3)	261.9 (147.8 to 441.4)	302.2 (155 to 544.4)	239.4 (137.1 to 423.6)	47.9 (2.6 to 105.5)	63.3 (12.4 to 132.7)	42 (-7.3 to 111.8)
		YLDs	45.4 (26.3 to 74.3)	56.5 (32.8 to 93.2)	34.2 (19.9 to 55)	63.4 (38.2 to 101.6)	82.2 (48.9 to 133.1)	47.7 (28.5 to 76.4)	39.8 (29.5 to 52.2)	45.4 (32 to 60.9)	39.5 (25.2 to 56.5)
	High systolic blood pressure	Deaths	42.9 (32.7 to 55.1)	47.1 (33.9 to 61.7)	38.7 (28.3 to 54.9)	42.2 (28.2 to 61.3)	54.5 (34.5 to 78.7)	35.3 (22.8 to 55)	-1.6 (-32.8 to 37.4)	15.7 (-24.8 to 72.9)	-8.7 (-38.5 to 32.5)
		DALYs	1035.5 (806.5 to 1280.5)	1093.2 (803.2 to 1389.7)	975.1 (744.9 to 1316.1)	1068.5 (757.4 to 1481.4)	1255.5 (872.9 to 1710.6)	943.6 (644.9 to 1405.9)	3.2 (-24.4 to 37.1)	14.9 (-18.8 to 62.8)	-3.2 (-31 to 36.3)
		YLLs	927.3 (715.6 to 1155.5)	961.3 (688.2 to 1228.5)	890.6 (674.5 to 1236.6)	913.1 (616.7 to 1328.7)	1054.4 (694.5 to 1526.7)	825.5 (535.1 to 1279.7)	-1.5 (-31.1 to 35.7)	9.7 (-25.5 to 60.7)	-7.3 (-36.8 to 34.1)
		YLDs	108.3 (73.4 to 145.4)	131.9 (86.1 to 180.9)	84.5 (56.3 to 115.3)	155.4 (108.5 to 205.4)	201.1 (140.5 to 267.1)	118.2 (80.8 to 158.8)	43.5 (25 to 69.1)	52.5 (25.5 to 89.6)	39.9 (19.1 to 67.2)
	High temperature	Deaths	1.7 (0 to 5.4)	1.7 (0 to 5.5)	1.6 (0 to 5.4)	1.8 (0.3 to 4.7)	2.3 (0.3 to 5.9)	1.6 (0.2 to 4.2)	10.9 (-74.5 to 142.7)	33.6 (-70.5 to 202.2)	1 (-77.7 to 126.1)
		YLLs	45.8 (1 to 151.5)	42.8 (0.8 to 141.9)	48.6 (1.2 to 168.1)	39.7 (5.6 to 100.1)	42.2 (6.2 to 107.7)	39.1 (5.5 to 102.1)	-13.3 (-80.4 to 86.7)	-1.2 (-78.2 to 112.5)	-19.5 (-82.6 to 74.4)
	Household air pollution from solid fuels	Deaths	4.6 (2.1 to 8.7)	5.6 (2.6 to 10.6)	3.6 (1.5 to 7.1)	0 (0 to 0.1)	0 (0 to 0.1)	0 (0 to 0)	-99.5 (-99.8 to -98.8)	-99.4 (-99.8 to -98.5)	-99.5 (-99.8 to -98.9)
		DALYs	119.1 (54.1 to 221.1)	142.4 (67.5 to 262.9)	95.5 (41 to 186.5)	0.6 (0.2 to 1.4)	0.8 (0.3 to 1.9)	0.4 (0.1 to 1.1)	-99.5 (-99.8 to -98.7)	-99.4 (-99.8 to -98.6)	-99.5 (-99.8 to -98.8)
		YLLs	105.8 (48.2 to 200.7)	124.5 (58.4 to 235.7)	87 (37.3 to 169.9)	0.5 (0.2 to 1.2)	0.7 (0.2 to 1.6)	0.4 (0.1 to 0.9)	-99.5 (-99.8 to -98.8)	-99.5 (-99.8 to -98.6)	-99.6 (-99.8 to -98.9)
		YLDs	13.2 (5.8 to 25.3)	17.9 (8 to 33.8)	8.5 (3.4 to 17)	0.1 (0 to 0.2)	0.1 (0 to 0.3)	0.1 (0 to 0.1)	-99.3 (-99.7 to -98.3)	-99.2 (-99.7 to -98.2)	-99.3 (-99.7 to -98.4)
	Kidney dysfunction	Deaths	7.7 (5.6 to 10.3)	9.1 (6.3 to 12.3)	6.3 (4.5 to 9)	10 (6.3 to 15.1)	12.8 (7.5 to 20.2)	8.2 (4.9 to 13.1)	30.1 (-8 to 75.7)	40.5 (-6.1 to 100.2)	29.9 (-9.9 to 80.5)
		DALYs	176.1 (137 to 219.8)	206.6 (155 to 262.4)	145.1 (110.2 to 195.1)	237.5 (167.1 to 335.2)	293 (196.3 to 420.2)	195.3 (131.4 to 291.2)	34.9 (0.4 to 75.2)	41.8 (2.4 to 92.7)	34.6 (-1.9 to 80.9)
		YLLs	154.7 (117.5 to 196.5)	178.4 (130.4 to 228.8)	130.6 (97.9 to 178.8)	200.4 (133.4 to 295)	243.5 (156.2 to 365.8)	168.4 (109.1 to 265.1)	29.5 (-9.3 to 74.9)	36.5 (-6.3 to 95.7)	29 (-10.5 to 80.9)
		YLDs	21.4 (14.5 to 29.1)	28.2 (19.2 to 38.3)	14.5 (9.6 to 19.7)	37.1 (24.8 to 50.4)	49.5 (33 to 67.3)	26.9 (17.9 to 36.4)	73.5 (59.6 to 86.4)	75.4 (59.1 to 91.8)	85.1 (65 to 104.6)
	Lead exposure	Deaths	6.2 (3.9 to 8.9)	5.2 (3 to 7.8)	7.2 (4.7 to 10.5)	5.2 (2.9 to 8.3)	5.3 (2.7 to 8.5)	5.3 (2.9 to 8.9)	-16.7 (-40.8 to 13.3)	0.9 (-30.8 to 40.8)	-26.5 (-49.2 to 3.3)
		DALYs	159.4 (102.3 to 222)	131.7 (75.1 to 195.7)	186.7 (123.3 to 266.3)	118.1 (65.7 to 186.6)	112.1 (58.3 to 178.1)	125.3 (71.4 to 204.1)	-25.9 (-45.2 to -2.3)	-14.9 (-39.4 to 15)	-32.9 (-51.6 to -8.5)
		YLLs	143.3 (91.1 to 201.6)	115.7 (65 to 171.9)	170.6 (112.2 to 248)	101.5 (54.5 to 164)	94.8 (47.4 to 154.9)	109.3 (58.1 to 185)	-29.2 (-49.7 to -2.5)	-18.1 (-44.4 to 15.4)	-35.9 (-56.3 to -9.3)
		YLDs	16.1 (9.5 to 24.2)	16 (8.8 to 24.7)	16.2 (9.9 to 23.6)	16.6 (9.2 to 25.4)	17.3 (9.1 to 27)	16 (9.3 to 24)	2.9 (-9.6 to 13)	8.3 (-7.9 to 23)	-1.2 (-15.2 to 10.7)
	Low physical activity	Deaths	4.4 (0.9 to 9.6)	4.8 (1.1 to 10.5)	3.9 (0.7 to 8.7)	5.3 (1.1 to 12.2)	7.5 (1.7 to 16.1)	4.3 (0.7 to 10.5)	22.8 (-12.6 to 63.7)	54.8 (7.6 to 119)	11.2 (-25.1 to 53.9)
		DALYs	73.6 (13.9 to 175.6)	80.5 (17.5 to 190.5)	66.5 (10.9 to 162.5)	100 (17.4 to 238.4)	127.2 (26.6 to 287.7)	83.7 (12.5 to 217)	35.8 (-1.8 to 74.6)	58 (13.9 to 111.1)	25.9 (-12.8 to 68.5)
		YLLs	61.5 (12 to 148.8)	65.8 (14.5 to 154)	57.2 (9.4 to 142.7)	83.1 (14.8 to 201)	105.1 (22.1 to 235.8)	70.8 (11 to 189.6)	35 (-7.5 to 79.7)	59.9 (8.6 to 121.6)	23.9 (-17.9 to 72.3)
		YLDs	12.1 (2.2 to 30.4)	14.8 (2.8 to 36.5)	9.3 (1.5 to 24.2)	16.9 (3 to 41.8)	22.1 (4.4 to 52.7)	12.9 (2 to 33.4)	40 (23.9 to 62.1)	49.6 (30.3 to 80.4)	38.6 (20.2 to 66.1)
	Low temperature	Deaths	4.5 (-0.1 to 9.7)	4.6 (-0.1 to 9.9)	4.3 (-0.1 to 9.8)	3.5 (-0.1 to 8.1)	4.3 (-0.1 to 10)	3.1 (-0.1 to 7.3)	-21.5 (-61.8 to 17.9)	-5.3 (-57.4 to 44)	-28.7 (-63.5 to 6.1)
		YLLs	123.3 (-1.8 to 269.1)	115.2 (-1.6 to 252.7)	130.8 (-1.9 to 284.6)	75.6 (-1.8 to 172)	80.6 (-1.9 to 186.8)	74.3 (-1.8 to 166.9)	-38.7 (-68.7 to -7.9)	-30 (-68 to 4.6)	-43.2 (-69.7 to -14.1)

Location	Risk factor	Measure	1990			2019			% Change (1990 to 2019)		
			Both	Female	Male	Both	Female	Male	Both	Female	Male
	Secondhand smoke	Deaths	4.1 (3 to 5.4)	5.2 (3.6 to 6.7)	3.1 (2.2 to 4.4)	3 (1.9 to 4.4)	4.4 (2.8 to 6.5)	1.9 (1.2 to 3.1)	-28 (-48.9 to -3.7)	-15.7 (-40.6 to 15.9)	-36.5 (-55.9 to -10.3)
		DALYs	113 (81 to 143.6)	147 (101.8 to 190)	79.4 (57.3 to 107.5)	83.2 (55.2 to 120.8)	119.7 (77.7 to 172.3)	52.6 (32.4 to 80.4)	-26.4 (-46 to -3.5)	-18.6 (-41.1 to 10)	-33.8 (-53.1 to -7.2)
		YLLs	105.2 (75.5 to 134.4)	135.3 (92.1 to 176.2)	75.3 (54.2 to 102.7)	74.9 (48.5 to 110.3)	106.5 (67.7 to 157.1)	48.6 (29 to 75.9)	-28.8 (-49.5 to -4.2)	-21.3 (-45.1 to 9.3)	-35.4 (-55.5 to -8)
		YLDs	7.8 (5 to 10.9)	11.7 (7.5 to 16.3)	4.1 (2.6 to 5.6)	8.3 (5.4 to 11.5)	13.2 (8.6 to 18.4)	3.9 (2.5 to 5.5)	5.3 (-3.1 to 14.5)	12.9 (2.6 to 24.7)	-3.5 (-15.6 to 10)
	Smoking	Deaths	10.4 (8.5 to 14)	1.1 (0.8 to 1.6)	19.7 (16 to 26.7)	10.2 (6.9 to 15.2)	1.2 (0.7 to 1.8)	17.4 (11.6 to 26)	-1.7 (-30.9 to 35.3)	3.6 (-38.5 to 64.5)	-11.8 (-38.2 to 22.8)
		DALYs	289.2 (242.3 to 374.7)	29.4 (20 to 42.4)	547.6 (454.9 to 714.5)	282.2 (202.3 to 403.2)	30.1 (19.4 to 44.8)	497.4 (350.3 to 716.2)	-2.4 (-28.3 to 32.1)	2.4 (-36.7 to 59.6)	-9.2 (-33.8 to 23.5)
		YLLs	261.4 (216.1 to 348.8)	25.6 (17.1 to 36.9)	495.9 (407.4 to 663.5)	244.5 (165.8 to 364.5)	25 (15.4 to 38.4)	431.7 (288.4 to 650.1)	-6.5 (-34.4 to 31.6)	-2.2 (-42 to 59)	-12.9 (-39.7 to 23.1)
		YLDs	27.8 (19.8 to 35.9)	3.8 (2.4 to 5.6)	51.7 (36.6 to 67.5)	37.7 (26.8 to 48.1)	5 (3.3 to 7.4)	65.6 (46.4 to 84.7)	35.6 (22.6 to 50)	33.6 (-6 to 95.7)	27 (14.9 to 40.7)
Iran (Islamic Republic of)	All risk factors	Deaths	97 (80.7 to 111.5)	95.4 (76.2 to 111.6)	97.2 (82.1 to 112.5)	54.7 (47.4 to 61.1)	56 (47.8 to 63.5)	53.7 (46.6 to 60)	-43.6 (-49.3 to -33.2)	-41.3 (-48.2 to -26.3)	-44.8 (-53.1 to -35.5)
		DALYs	1911.6 (1660.7 to 2129.8)	1859.3 (1578.8 to 2094.4)	1944.9 (1683.9 to 2197.4)	1072.1 (972.6 to 1165.2)	1058.3 (935.1 to 1166)	1088.5 (992 to 1187.8)	-43.9 (-49.4 to -35.4)	-43.1 (-49.2 to -31.6)	-44 (-51.2 to -35.5)
		YLLs	1725.3 (1487.2 to 1941)	1641.7 (1371.4 to 1864.8)	1788.7 (1539 to 2046.3)	905.8 (819.4 to 984)	868.2 (766.8 to 959.1)	946 (855.1 to 1033.2)	-47.5 (-53.4 to -38.7)	-47.1 (-53.4 to -34.8)	-47.1 (-54.6 to -38.2)
		YLDs	186.3 (133.9 to 241.9)	217.6 (155.9 to 280.2)	156.2 (111.1 to 204.2)	166.3 (118.7 to 213.7)	190.1 (136.4 to 245.1)	142.6 (101 to 185.2)	-10.7 (-14.1 to -7.4)	-12.6 (-16.1 to -8.7)	-8.7 (-13 to -4.4)
	Alcohol use	Deaths	0.1 (0 to 0.2)	0 (0 to 0)	0.1 (0 to 0.3)	0.2 (0.1 to 0.3)	0.1 (0 to 0.1)	0.4 (0.2 to 0.6)	251.4 (-2312.6 to 2759.2)	-841.6 (-4114.3 to 3814.2)	201 (-788.8 to 2447.6)
		DALYs	2.2 (0 to 4.8)	0 (-0.6 to 0.9)	4.1 (0.4 to 8.6)	6.4 (3.9 to 9.3)	1.3 (0.1 to 2.7)	11.5 (7 to 16.9)	197.8 (20 to 2297.6)	3217.1 (-7312.1 to 4765.7)	180 (39.2 to 1514.9)
		YLLs	2 (0.1 to 4.4)	0.1 (-0.5 to 0.8)	3.8 (0.4 to 8)	5.6 (3.4 to 8.1)	1.1 (0.2 to 2.2)	10.1 (6.2 to 14.7)	177.5 (13.7 to 1887.5)	1833.3 (-5279.6 to 5155.7)	163.2 (31.6 to 1255.6)
		YLDs	0.1 (0 to 0.4)	0 (-0.1 to 0.1)	0.3 (0 to 0.7)	0.8 (0.4 to 1.4)	0.2 (0 to 0.5)	1.5 (0.7 to 2.4)	483.4 (-3407.7 to 4949.8)	-1293.2 (-7172.6 to 8407.7)	401.3 (-1968.7 to 4439.8)
	Ambient particulate matter pollution	Deaths	23.6 (18.7 to 29.2)	22.4 (17.1 to 28.2)	24.6 (18.8 to 30.6)	14.5 (12.3 to 16.8)	14.5 (12 to 16.8)	14.6 (12.3 to 17)	-38.6 (-47 to -24.6)	-35.3 (-45.4 to -16.7)	-40.7 (-50.8 to -26.2)
		DALYs	520.8 (418.5 to 632.7)	490.6 (383.1 to 604.4)	546.2 (434.9 to 673.5)	325 (280.7 to 372.7)	315.1 (267.7 to 362.8)	335.4 (288.4 to 384.9)	-37.6 (-46 to -24.3)	-35.8 (-45.4 to -20.5)	-38.6 (-48.6 to -24)
		YLLs	466.2 (374.2 to 571.1)	428 (333.6 to 535.3)	499.2 (393.4 to 619.1)	270.5 (233.7 to 309.8)	252.8 (214.9 to 290.3)	288.6 (247.2 to 331.9)	-42 (-50.1 to -28.9)	-40.9 (-50.3 to -25.1)	-42.2 (-52.6 to -28.1)
		YLDs	54.6 (37.5 to 72.8)	62.6 (43 to 83.6)	46.9 (32 to 62.6)	54.6 (38.3 to 71.8)	62.3 (43.8 to 81.9)	46.9 (32.8 to 61.9)	-0.1 (-8.8 to 14.4)	-0.6 (-10.3 to 16.1)	-0.2 (-8.6 to 13)
	Diet high in red meat	Deaths	3.6 (1.3 to 5.6)	3.5 (1.2 to 5.5)	3.7 (1.4 to 5.7)	1.6 (0.6 to 2.6)	1.6 (0.5 to 2.6)	1.7 (0.6 to 2.7)	-54.7 (-64 to -45.7)	-53.8 (-65.1 to -41.5)	-54.9 (-65.6 to -44.9)
		DALYs	91.4 (35.5 to 138.2)	88.4 (33.9 to 134.4)	93.6 (37.3 to 143.3)	40.9 (14.1 to 63.5)	38.9 (12.5 to 61)	42.9 (15.4 to 66.6)	-55.3 (-64.5 to -47.5)	-56 (-66.7 to -47.2)	-54.2 (-64.5 to -45.4)
		YLLs	80.9 (32.2 to 123.2)	76 (29.1 to 115.8)	85 (34.6 to 130)	33.4 (12 to 51.8)	30.4 (10.5 to 47.8)	36.5 (13.5 to 56.8)	-58.7 (-67.5 to -50.9)	-60.1 (-69.9 to -50.7)	-57.1 (-67.5 to -48.1)
		YLDs	10.5 (3.2 to 17.5)	12.4 (3.7 to 20.6)	8.6 (2.6 to 14.5)	7.5 (2.1 to 12.6)	8.5 (2.4 to 14.5)	6.4 (1.8 to 11.1)	-28.5 (-39.6 to -21.5)	-31.2 (-43.3 to -22.6)	-25.4 (-38.3 to -16.2)
	Diet high in sodium	Deaths	2 (0.4 to 7.5)	1.3 (0.4 to 5.2)	2.6 (0.3 to 10)	1 (0.2 to 4.1)	0.7 (0.2 to 2.8)	1.4 (0.2 to 5.2)	-47.1 (-60.5 to -27.2)	-46.2 (-61.8 to -23.6)	-47.3 (-63.1 to -14.7)
		DALYs	44.7 (7.2 to 170.3)	29.1 (7.9 to 116.2)	59.2 (5.6 to 224.9)	23.9 (4.1 to 93.4)	15.5 (4.4 to 64)	32.4 (3.4 to 122.4)	-46.5 (-59.5 to -25.2)	-46.7 (-61.5 to -25.9)	-45.2 (-61.5 to -10.5)
		YLLs	40.4 (6.5 to 153.6)	25.5 (7.1 to 103)	54.2 (5.2 to 205.8)	20.2 (3.5 to 78.3)	12.5 (3.6 to 51.4)	27.9 (3 to 105.4)	-50 (-62.4 to -29.3)	-50.9 (-65 to -30.2)	-48.5 (-64.4 to -15.9)
		YLDs	4.3 (0.6 to 17.1)	3.6 (0.8 to 14.6)	5 (0.4 to 19.5)	3.7 (0.6 to 14.5)	3 (0.7 to 12.3)	4.5 (0.4 to 16.8)	-13.5 (-30.8 to 14)	-16.8 (-38.1 to 9.5)	-9.9 (-33.4 to 46.9)
	Diet low in fiber	Deaths	2.8 (0.7 to 5.4)	2.7 (0.6 to 5.3)	2.9 (0.7 to 5.4)	0.9 (0.2 to 1.7)	0.9 (0.2 to 1.8)	0.9 (0.2 to 1.7)	-69.1 (-76.2 to -58.6)	-68.5 (-78.6 to -53.8)	-69.1 (-78.3 to -57.4)
		DALYs	62.4 (14 to 120.4)	60.5 (13.1 to 118.4)	63.7 (14.4 to 121.5)	18.1 (4.6 to 36.7)	17.1 (4.3 to 35.3)	19.1 (5 to 38.4)	-71 (-76.7 to -63.1)	-71.7 (-79.1 to -61.6)	-70.1 (-77.7 to -60.4)
		YLLs	55.6 (12.3 to 106.7)	52.5 (11.3 to 103)	58.1 (13.1 to 111.4)	15 (3.9 to 30.5)	13.7 (3.5 to 28.6)	16.4 (4.2 to 33.1)	-73 (-78.3 to -64.8)	-73.9 (-80.9 to -63.7)	-71.8 (-79.3 to -62.4)
		YLDs	6.8 (1.4 to 13.6)	8 (1.6 to 16.1)	5.6 (1.2 to 11.2)	3 (0.7 to 6.4)	3.4 (0.8 to 7.3)	2.7 (0.6 to 5.7)	-55.2 (-62.8 to -46.1)	-57.2 (-66.7 to -46.4)	-52.4 (-62.1 to -41)
	Diet low in fruits	Deaths	2.4 (1.1 to 4.2)	2.5 (1.1 to 4.3)	2.3 (1 to 4)	0.9 (0.4 to 1.5)	0.9 (0.4 to 1.6)	0.8 (0.4 to 1.5)	-64.2 (-73.8 to -52.7)	-63 (-75.8 to -47.2)	-64.7 (-75.5 to -50.7)
		DALYs	58.4 (26.8 to 96.9)	59.8 (27.2 to 98.4)	56.6 (26.3 to 93.4)	21.7 (9.8 to 35.7)	22.1 (9.9 to 36.9)	21.3 (9.8 to 35.8)	-62.9 (-71.1 to -54)	-63.1 (-73.8 to -51.7)	-62.5 (-72.6 to -51.2)
		YLLs	52.2 (24.2 to 85.1)	52.2 (24.4 to 85.4)	51.8 (24 to 84.5)	17.8 (8.3 to 29.1)	17.4 (8.1 to 28.6)	18.2 (8.5 to 30.7)	-65.9 (-73.6 to -57.2)	-66.7 (-76.9 to -55.7)	-64.8 (-74.3 to -53.7)
		YLDs	6.2 (2.1 to 11.5)	7.7 (2.6 to 14.4)	4.8 (1.7 to 8.9)	3.9 (1.4 to 7)	4.7 (1.6 to 8.9)	3 (1.1 to 5.6)	-37.9 (-47.6 to -26.4)	-38.8 (-51.7 to -25.2)	-36.8 (-51.2 to -22.2)
	Diet low in vegetables	Deaths	2.4 (0.8 to 4.1)	2.2 (0.8 to 4)	2.5 (0.8 to 4.3)	0.5 (0.2 to 0.8)	0.5 (0.2 to 0.8)	0.5 (0.2 to 0.9)	-80.8 (-87.6 to -69.1)	-79.9 (-88.2 to -67.4)	-81.3 (-88.3 to -69.7)
		DALYs	52.8 (16.6 to 92.9)	49.7 (15.3 to 87.2)	55.4 (17.7 to 98.4)	7.5 (2.9 to 14.1)	7.1 (2.8 to 13.2)	8 (3 to 15.3)	-85.7 (-90.8 to -75.5)	-85.8 (-91.2 to -74.2)	-85.5 (-91.1 to -75.4)
		YLLs	47.4 (15.2 to 84.2)	43.5 (14 to 76.5)	50.8 (16.4 to 90.4)	6.5 (2.5 to 12)	5.9 (2.4 to 11)	7.1 (2.6 to 13.5)	-86.3 (-91.3 to -76.5)	-86.4 (-91.7 to -75.5)	-86.1 (-91.4 to -76.4)
		YLDs	5.4 (1.3 to 10)	6.2 (1.4 to 11.5)	4.6 (1.1 to 8.7)	1.1 (0.4 to 2.1)	1.1 (0.5 to 2.3)	1 (0.4 to 2)	-80.2 (-87 to -64.6)	-81.4 (-88.4 to -64.6)	-78.7 (-86.9 to -61.9)
	Diet low in whole grains	Deaths	8.2 (4.8 to 10.8)	8.1 (4.8 to 10.8)	8.1 (4.6 to 10.7)	4.7 (2.9 to 5.9)	4.8 (2.9 to 6.2)	4.6 (2.8 to 5.8)	-42.8 (-49.8 to -32.4)	-40.5 (-47.7 to -25.1)	-43.6 (-52 to -34.9)
		DALYs	163.6 (88.9 to 214.7)	161.6 (88.7 to 213.7)	163.9 (88.7 to 217.4)	94.2 (53 to 120.6)	94 (53.3 to 121.3)	94.7 (52.8 to 122)	-42.4 (-47.9 to -34.4)	-41.8 (-48 to -30.3)	-42.3 (-50.1 to -34.2)
		YLLs	143.6 (78.4 to 189)	137.8 (77 to 184.2)	147.6 (80.1 to 196.3)	76.7 (44 to 98.2)	73.9 (42.6 to 95.2)	79.7 (45.1 to 102.2)	-46.6 (-52.9 to -38.1)	-46.4 (-53.3 to -33.1)	-46 (-54.4 to -37.5)
		YLDs	20 (10.3 to 29.3)	23.8 (12.3 to 34.7)	16.4 (8.4 to 24.3)	17.5 (9.1 to 25.6)	20.1 (10.5 to 29)	15 (7.8 to 22)	-12.4 (-15.4 to -9.2)	-15.6 (-18.8 to -12.1)	-8.4 (-12.7 to -3.6)
	High body-mass index	Deaths	15.5 (8.9 to 23.1)	17.8 (10.7 to 26)	13.3 (6.9 to 21)	11.8 (7.7 to 16.4)	12.1 (7.9 to 16.9)	11.4 (7.3 to 16)	-24.2 (-38.7 to -0.5)	-31.9 (-46.6 to -9.7)	-14 (-33.6 to 27.2)
		DALYs	457.9 (282.9 to 645.9)	531.6 (341.4 to 733.6)	389.3 (215.5 to 592.9)	353.2 (248.5 to 462.5)	355.7 (255.4 to 465)	350.3 (238 to 468.2)	-22.9 (-37.2 to 0.4)	-33.1 (-45.5 to -15.6)	-10 (-29.8 to 32.9)
		YLLs	404.2 (247.9 to 576.8)	457.8 (295.9 to 636.6)	354 (196.6 to 541.3)	287.2 (202.7 to 375.3)	276.1 (198.7 to 360.2)	297.9 (205.1 to 396.9)	-28.9 (-42.7 to -7.1)	-39.7 (-51.7 to -22.3)	-15.8 (-35.1 to 24.4)
		YLDs	53.7 (30.9 to 82.4)	73.8 (44.3 to 111.4)	35.3 (18.3 to 57.1)	66 (42.1 to 94.5)	79.6 (50.9 to 112.3)	52.3 (31.9 to 76.6)	22.7 (7 to 50.5)	7.8 (-5 to 29.8)	48.1 (22.3 to 113.7)
	High fasting [illegible]	Deaths	18.2 (10.2 to 34.4)	17.7 (9.7 to 34.8)	18.4 (10.7 to 33.4)	19.1 (10.3 to 38.3)	20.4 (10.5 to 41.6)	18 (9.8 to 35.2)	5.1 (-15.3 to 30.3)	14.8 (-10.3 to 48.9)	-2 (-21.5 to 23.7)

Location	Risk factor	Measure	1990			2019			% Change (1990 to 2019)		
			Both	Female	Male	Both	Female	Male	Both	Female	Male
	plasma glucose	DALYs	333.2 (209.5 to 554.1)	319.9 (197.5 to 548.8)	342.5 (212.1 to 543.1)	352.2 (207.7 to 596.8)	366.1 (208.6 to 639.3)	339.2 (207.9 to 558.6)	5.7 (-13.3 to 29.2)	14.5 (-10.4 to 47.7)	-1 (-20.5 to 23.1)
		YLLs	301.5 (187.5 to 502.5)	283.8 (175.1 to 492.5)	315.2 (195 to 505.2)	299.6 (177.2 to 510)	304.3 (174.6 to 547.1)	295.9 (181.4 to 495.2)	-0.6 (-19.4 to 23.4)	7.2 (-17 to 41)	-6.1 (-25.8 to 18.6)
		YLDs	31.7 (17.4 to 53.6)	36.2 (19.8 to 60.9)	27.3 (15.1 to 46.4)	52.6 (27.7 to 92)	61.9 (32.7 to 108.2)	43.2 (23.2 to 74.7)	66.2 (41.9 to 93.2)	71.1 (41.7 to 103.4)	58.7 (36.2 to 85.8)
	High LDL cholesterol	Deaths	20 (7.8 to 40.7)	20.5 (7.3 to 43.9)	19 (7.8 to 39.5)	11 (4 to 22.8)	11.7 (3.8 to 24.6)	10.4 (4.2 to 21.6)	-44.9 (-53.4 to -35.6)	-42.8 (-53.4 to -29.7)	-45.4 (-54.2 to -36.9)
		DALYs	421.7 (247.9 to 713.2)	433.1 (249.8 to 733.7)	406.1 (241.8 to 663.2)	234.3 (135.1 to 399.7)	239.7 (131.7 to 415.8)	229.8 (135.9 to 377)	-44.4 (-50.7 to -37.5)	-44.6 (-51.8 to -35)	-43.4 (-50.8 to -35.6)
		YLLs	366.2 (208.8 to 624.7)	364.5 (200 to 645.2)	363.1 (211.9 to 606.8)	187.5 (104.3 to 324.6)	184.5 (94.5 to 332.8)	191.2 (112.1 to 320.7)	-48.8 (-55.5 to -41.3)	-49.4 (-58.2 to -38.5)	-47.3 (-55.3 to -38.9)
		YLDs	55.5 (33.5 to 89.9)	68.6 (41.7 to 110.2)	43 (25.6 to 70.3)	46.9 (27.9 to 77.1)	55.2 (32.8 to 90.2)	38.6 (23 to 63.6)	-15.6 (-18.5 to -12.5)	-19.5 (-23.2 to -15.5)	-10.2 (-14.7 to -5.4)
	High systolic blood pressure	Deaths	52.5 (40.4 to 66.3)	53.1 (40.1 to 68)	50.9 (38.4 to 64.2)	29.6 (23.5 to 36.4)	30.5 (23.5 to 38.3)	28.9 (22.8 to 35.2)	-43.6 (-50.2 to -33)	-42.5 (-50.5 to -27.6)	-43.3 (-52.1 to -33.7)
		DALYs	1069 (854.1 to 1295.5)	1065 (842.5 to 1294.8)	1062.2 (833.7 to 1305.2)	612.9 (506.3 to 718.1)	606.4 (493.8 to 722.3)	620.4 (513 to 720.1)	-42.7 (-48.8 to -33.6)	-43.1 (-50.1 to -31.3)	-41.6 (-50.1 to -32.6)
		YLLs	964.5 (768.2 to 1171.1)	940.6 (741.6 to 1147.2)	976.5 (768.5 to 1200.7)	516.6 (426 to 603.6)	496.2 (403.6 to 592)	538.1 (443.3 to 632.2)	-46.4 (-52.4 to -37.1)	-47.2 (-54.3 to -34.5)	-44.9 (-53.6 to -35.4)
		YLDs	104.5 (72.1 to 140.3)	124.4 (86.7 to 166.8)	85.6 (58.4 to 116.7)	96.2 (67.4 to 128.1)	110.1 (77.6 to 147.4)	82.2 (57.3 to 110.4)	-7.9 (-12.1 to -3.3)	-11.4 (-16.9 to -5.4)	-4 (-9.2 to 2)
	High temperature	Deaths	0.6 (-0.3 to 1.5)	0.6 (-0.3 to 1.5)	0.6 (-0.3 to 1.5)	0.5 (0.1 to 0.8)	0.5 (0.1 to 0.8)	0.5 (0.1 to 0.8)	-25.3 (-349.5 to 245.7)	-23.5 (-321.9 to 246)	-25.6 (-372.2 to 257.4)
		YLLs	10.4 (-4.6 to 26)	10 (-4.3 to 24.7)	10.8 (-4.8 to 27.1)	7.7 (1.6 to 14)	7.3 (1.6 to 13.3)	8.1 (1.7 to 14.9)	-26.1 (-404.2 to 244)	-26.4 (-293.6 to 261.4)	-24.7 (-488.1 to 297.9)
	Household air pollution from solid fuels	Deaths	5 (2.4 to 8.7)	5.5 (2.6 to 9.4)	4.4 (2 to 8.1)	0 (0 to 0.1)	0 (0 to 0.1)	0 (0 to 0.1)	-99.3 (-99.7 to -98.4)	-99.3 (-99.7 to -98.4)	-99.4 (-99.8 to -98.5)
		DALYs	109.4 (52.8 to 186.3)	121 (58.6 to 203.2)	97.2 (45.2 to 176.4)	0.8 (0.3 to 1.7)	0.9 (0.3 to 2)	0.7 (0.2 to 1.5)	-99.3 (-99.7 to -98.3)	-99.3 (-99.7 to -98.3)	-99.3 (-99.7 to -98.4)
		YLLs	98.3 (47.6 to 172.2)	106.4 (51.6 to 180.9)	89.4 (41.3 to 163)	0.6 (0.2 to 1.4)	0.7 (0.3 to 1.6)	0.6 (0.2 to 1.3)	-99.3 (-99.7 to -98.4)	-99.3 (-99.7 to -98.4)	-99.4 (-99.7 to -98.4)
		YLDs	11.1 (5.4 to 19.5)	14.6 (6.9 to 25)	7.8 (3.5 to 14.4)	0.1 (0 to 0.3)	0.2 (0.1 to 0.4)	0.1 (0 to 0.2)	-98.9 (-99.5 to -97.4)	-98.9 (-99.5 to -97.3)	-99 (-99.6 to -97.5)
	Kidney dysfunction	Deaths	10.4 (6.9 to 14.2)	10.7 (6.6 to 15.3)	10 (6.7 to 13.1)	6.6 (4 to 9.1)	7 (3.8 to 10)	6.3 (4.1 to 8.5)	-36.4 (-48.9 to -22.9)	-34.8 (-49 to -15.2)	-37.1 (-51.5 to -26)
		DALYs	216.8 (163.6 to 275.8)	225.5 (163.3 to 294.1)	206.9 (155.4 to 259.5)	139.5 (105.2 to 173.9)	146.1 (103.9 to 186.2)	133 (102.1 to 163.5)	-35.6 (-44.1 to -25)	-35.2 (-44.3 to -20.3)	-35.7 (-45.9 to -26.1)
		YLLs	193.9 (144.6 to 248.1)	197 (140.7 to 261.4)	189.2 (139.7 to 239.1)	116.5 (87.2 to 146.5)	118.5 (82.9 to 153.9)	114.7 (87 to 141.3)	-39.9 (-49 to -28.4)	-39.9 (-49.8 to -23.5)	-39.4 (-49.8 to -29.2)
		YLDs	22.9 (14.9 to 31.1)	28.5 (18.7 to 38.5)	17.7 (11.5 to 24.2)	23 (15.2 to 31.4)	27.7 (18.3 to 37.9)	18.3 (11.9 to 24.8)	0.4 (-4.6 to 5.4)	-2.8 (-8.4 to 2.9)	3.5 (-2.7 to 9.9)
	Lead exposure	Deaths	8.5 (5.7 to 11.9)	7 (4.3 to 10.2)	9.9 (6.7 to 13.7)	4.6 (3 to 6.4)	3.8 (2.3 to 5.6)	5.4 (3.7 to 7.4)	-45.8 (-54 to -35.7)	-45.2 (-53.9 to -29.9)	-45.7 (-55 to -35)
		DALYs	187 (129.8 to 251.9)	147.9 (93.8 to 205.2)	223 (158 to 297.6)	87.5 (57.8 to 117.9)	68.6 (41.7 to 98.5)	106.5 (73.3 to 139.1)	-53.2 (-60.6 to -45.7)	-53.6 (-61.2 to -43.1)	-52.2 (-60.9 to -43.9)
		YLLs	169.6 (116.9 to 227.2)	130.8 (83.7 to 180.6)	205.3 (143.7 to 272.9)	75.4 (50.1 to 101.3)	57.4 (35.2 to 82)	93.6 (64.3 to 122.8)	-55.5 (-62.8 to -47.6)	-56.1 (-63.7 to -45.1)	-54.4 (-63 to -45.7)
		YLDs	17.5 (10.7 to 25.7)	17.2 (9.8 to 26)	17.7 (11.1 to 25.8)	12 (7 to 18.3)	11.2 (6.1 to 17.6)	12.9 (7.6 to 19.2)	-31 (-38.8 to -24.8)	-34.6 (-44.1 to -27)	-27.2 (-35.5 to -20.1)
	Low physical activity	Deaths	6.8 (1.4 to 15.7)	7.1 (1.5 to 15.8)	6.3 (1.1 to 15.1)	4.2 (0.9 to 9.4)	4.7 (1.1 to 10.3)	3.7 (0.6 to 8.8)	-38.9 (-47.4 to -25.6)	-34.4 (-43.8 to -14)	-41.7 (-51.1 to -31.4)
		DALYs	104.4 (19.7 to 255.9)	109.5 (23.3 to 255.2)	97.9 (16 to 256.1)	63.4 (12 to 154.7)	70.1 (15.1 to 163.5)	57.1 (9.2 to 146.1)	-39.3 (-47.1 to -27.3)	-36.1 (-44.7 to -18.5)	-41.6 (-49.7 to -31.9)
		YLLs	93.5 (17.9 to 230.4)	96.2 (20.9 to 224.2)	89.5 (14.7 to 234.1)	53.5 (10.5 to 129.9)	57.9 (12.6 to 134.4)	49.5 (8 to 126.2)	-42.8 (-51.6 to -29.7)	-39.8 (-49.2 to -21.1)	-44.7 (-52.9 to -34.5)
		YLDs	10.9 (1.8 to 28.9)	13.3 (2.3 to 34.5)	8.4 (1.2 to 23.3)	9.9 (1.6 to 25.9)	12.1 (2.2 to 30.2)	7.6 (1.1 to 20.7)	-9.1 (-14.2 to -0.6)	-9 (-16.4 to 2.4)	-9.1 (-14.9 to -1.2)
	Low temperature	Deaths	10.6 (7.7 to 13.9)	10.5 (7.5 to 13.9)	10.6 (7.6 to 13.8)	6 (4.5 to 7.6)	6.2 (4.6 to 7.9)	5.8 (4.4 to 7.4)	-43.7 (-50.7 to -32.4)	-41.3 (-48.8 to -25.5)	-45 (-53 to -34.8)
		YLLs	185.4 (136.3 to 239.6)	177.9 (127.7 to 234.4)	191 (141.3 to 245.9)	95.6 (71.6 to 120.4)	92.5 (69.3 to 117.7)	99.1 (75 to 124.9)	-48.4 (-54.9 to -39.3)	-48 (-54.9 to -36.4)	-48.1 (-55.9 to -38.6)
	Secondhand smoke	Deaths	3.6 (2.6 to 4.8)	3.8 (2.7 to 5.2)	3.4 (2.4 to 4.5)	1.9 (1.4 to 2.4)	2.1 (1.5 to 2.7)	1.7 (1.2 to 2.3)	-47.6 (-55.8 to -36.9)	-45.7 (-55.1 to -31.5)	-49.2 (-60 to -36.3)
		DALYs	83 (59.5 to 108.5)	92.4 (66.2 to 120.7)	73.8 (51.5 to 98.8)	43.2 (32.2 to 53.8)	48.4 (35.6 to 61.1)	38.1 (27.6 to 49.1)	-48 (-55 to -38.8)	-47.6 (-56 to -36.1)	-48.4 (-58.7 to -36.8)
		YLLs	77.3 (55.3 to 101.5)	84.6 (60.3 to 110.7)	70.1 (48.7 to 94.3)	38.2 (28.4 to 48)	41.7 (30.5 to 52.8)	34.8 (25.3 to 45)	-50.6 (-57.9 to -41.3)	-50.7 (-58.9 to -38.8)	-50.3 (-60.5 to -38.5)
		YLDs	5.7 (3.7 to 8)	7.7 (4.9 to 11)	3.7 (2.4 to 5.4)	5 (3.2 to 6.9)	6.7 (4.3 to 9.3)	3.2 (2 to 4.6)	-12.3 (-19.4 to -4.4)	-13.3 (-21.5 to -4.2)	-13 (-25.3 to 1.8)
	Smoking	Deaths	12.2 (10.1 to 14.4)	4.7 (3.3 to 6.5)	19.5 (16 to 23.1)	5.2 (4.6 to 5.9)	2 (1.5 to 2.5)	8.5 (7.4 to 9.7)	-56.8 (-64.3 to -47.5)	-58.6 (-72 to -38.3)	-56.3 (-64.1 to -46.8)
		DALYs	324.3 (276.6 to 378.9)	133.9 (95 to 185.4)	502.7 (427.3 to 592.9)	145.3 (129.3 to 161.8)	58.1 (45.2 to 73.5)	232.9 (206.4 to 260.1)	-55.2 (-62.8 to -46.8)	-56.6 (-69.5 to -39.1)	-53.7 (-61.2 to -44.8)
		YLLs	292.8 (247.7 to 345.6)	115.7 (81.7 to 158.1)	458.6 (387 to 545.8)	121.9 (109.2 to 135.8)	45.3 (35.4 to 58.4)	198.8 (176.2 to 223.3)	-58.4 (-65.4 to -49.8)	-60.8 (-72.9 to -44.4)	-56.7 (-64.4 to -47.7)
		YLDs	31.6 (21.9 to 41.6)	18.2 (11.4 to 26.6)	44.1 (30.9 to 58)	23.4 (16.6 to 30.5)	12.8 (8.6 to 17.6)	34.1 (23.7 to 44.6)	-25.8 (-33.6 to -17)	-29.7 (-48.3 to -4.6)	-22.6 (-29.5 to -15.1)
Iraq	All risk factors	Deaths	143.5 (121.1 to 168.9)	136.7 (111.1 to 165.7)	149.8 (118.7 to 187.4)	127 (104.4 to 149.1)	114.3 (92.8 to 135.4)	140.3 (113.7 to 164.7)	-11.4 (-29.7 to 8.2)	-16.4 (-34.9 to 5.4)	-6.4 (-28.4 to 20.5)
		DALYs	3169.9 (2706.7 to 3706)	2992 (2486 to 3536.8)	3346.1 (2664.8 to 4174.9)	2640.3 (2134.7 to 3137.5)	2359.3 (1924.9 to 2808.6)	2925.9 (2324.1 to 3540.4)	-16.7 (-33.9 to 3.1)	-21.1 (-38.9 to -0.5)	-12.6 (-33.6 to 14.1)
		YLLs	2875.3 (2425.1 to 3419.1)	2652.8 (2132.8 to 3210.6)	3097.7 (2430 to 3915.7)	2366.2 (1878.2 to 2871.8)	2044.2 (1624.3 to 2480.1)	2695.6 (2129.4 to 3291.8)	-17.7 (-36.7 to 4.2)	-22.9 (-42.6 to 0.7)	-13 (-35.5 to 16.1)
		YLDs	294.6 (217.8 to 373.9)	339.2 (251.9 to 429.2)	248.4 (181.3 to 317.3)	274.1 (200.1 to 345)	315.1 (229 to 398.6)	230.3 (166.1 to 290.8)	-7 (-11.9 to -1.5)	-7.1 (-14 to 0.4)	-7.3 (-14.3 to 0.3)
	Alcohol use	Deaths	1.5 (0.7 to 2.5)	0 (-0.4 to 0.5)	3 (1.5 to 4.9)	0.3 (-0.1 to 0.8)	-0.2 (-0.4 to 0)	0.8 (0 to 1.7)	-81.7 (-113.6 to -48.9)	-812 (-1450.4 to 1243.1)	-75.1 (-101.8 to -44.4)
		DALYs	49.6 (26.1 to 81)	4 (-6 to 15.7)	95.6 (51.8 to 153.5)	12 (-0.7 to 26.6)	-3.2 (-7.6 to 1.8)	27.1 (3.8 to 55.7)	-75.8 (-102.8 to -44.8)	-179.9 (-988.8 to 1007.9)	-71.6 (-94.9 to -41.4)
		YLLs	47.3 (25 to 76.5)	4.4 (-4.8 to 15.4)	90.4 (48.7 to 146)	11.7 (-0.1 to 25.3)	-2.4 (-6.3 to 1.9)	25.7 (3.9 to 52.3)	-75.3 (-100.4 to -44.5)	-154.6 (-1082.6 to 572.5)	-71.6 (-94.6 to -40.7)
		YLDs	2.4 (0.7 to 4.4)	-0.4 (-1.5 to 0.8)	5.2 (2.2 to 8.7)	0.3 (-0.6 to 1.5)	-0.8 (-1.5 to -0.1)	1.5 (-0.3 to 3.5)	-85.7 (-157.3 to -44.6)	91.5 (-1366.2 to 1088.6)	-71.9 (-108.8 to -38.1)
	Ambient particulate matter	Deaths	36.7 (24.1 to 49.4)	32.8 (20.7 to 45.5)	40.8 (27.6 to 56.7)	39.1 (29.6 to 49.4)	34.9 (26 to 44.2)	43.6 (32.8 to 54.8)	6.6 (-21.2 to 48)	6.3 (-23.7 to 56.5)	6.8 (-22 to 53.2)
		DALYs	899.1 (601.9 to 1215.4)	798 (507.2 to 1102.4)	1001.7 (683.1 to 1402.2)	915.6 (686.5 to 1176)	816.7 (613.4 to 1045)	1015.4 (749.2 to 1292.4)	1.8 (-25.7 to 44.6)	2.3 (-27.2 to 50.8)	1.4 (-28 to 47.4)

Location	Risk factor	Measure	1990			2019			% Change (1990 to 2019)		
			Both	Female	Male	Both	Female	Male	Both	Female	Male
	pollution	YLLs	813.9 (537.6 to 1106.4)	704.1 (446.4 to 986.4)	925.3 (619.8 to 1284.6)	815.7 (595.9 to 1055.5)	701.7 (512.8 to 917.4)	931.2 (674.8 to 1196)	0.2 (-28.4 to 43.7)	-0.3 (-31.4 to 49.7)	0.6 (-29.8 to 48.8)
		YLDs	85.3 (52.7 to 121.5)	93.9 (56.3 to 135.5)	76.4 (48.2 to 108.1)	99.9 (67.6 to 130.5)	115 (78.6 to 152.3)	84.1 (57 to 111.5)	17.2 (-2.9 to 57)	22.4 (-2.6 to 69.3)	10.2 (-7.8 to 45.3)
	Diet high in red meat	Deaths	3.2 (1 to 5.4)	3 (1 to 5.1)	3.4 (1.1 to 5.9)	1.9 (0.6 to 3.4)	1.7 (0.6 to 3)	2.1 (0.7 to 3.8)	-40.5 (-58.1 to -20.7)	-43.8 (-62.8 to -19.6)	-37.5 (-57.2 to -12.5)
		DALYs	87.8 (27.6 to 148.1)	82.4 (26.2 to 137.6)	93.1 (29.2 to 159.7)	48.3 (15 to 87.2)	42.9 (13.3 to 77.5)	53.7 (16.7 to 97.3)	-45 (-61.2 to -25.5)	-47.9 (-65.1 to -24.8)	-42.3 (-60.6 to -18.8)
		YLLs	79.1 (24.8 to 133.5)	72.3 (22 to 123.1)	85.9 (26.2 to 148.5)	42.8 (13.2 to 78.8)	36.6 (11.3 to 67)	49.1 (15.3 to 90)	-45.8 (-62.6 to -24.9)	-49.4 (-66.9 to -25)	-42.9 (-61.8 to -17.2)
		YLDs	8.6 (2.4 to 15.2)	10 (2.8 to 17.7)	7.3 (2.1 to 12.7)	5.5 (1.5 to 10.1)	6.3 (1.7 to 11.7)	4.6 (1.3 to 8.5)	-36.9 (-48.3 to -23.1)	-37.6 (-50.6 to -22.3)	-36 (-49.1 to -22.2)
	Diet high in sodium	Deaths	3 (0.4 to 12.4)	2 (0.4 to 8.2)	4.1 (0.3 to 17.5)	2.6 (0.4 to 10.1)	1.5 (0.3 to 6.4)	3.7 (0.3 to 14.9)	-14.7 (-47.8 to 64.8)	-22.8 (-61.4 to 90.1)	-10 (-48.3 to 89.9)
		DALYs	75.6 (8.6 to 312.5)	48.2 (8.3 to 201.8)	103.7 (7.1 to 432.3)	61.9 (7.6 to 244.6)	35.7 (6.8 to 148.5)	88.9 (6.6 to 346.7)	-18.1 (-50.6 to 63)	-26 (-65 to 80.6)	-14.3 (-51.2 to 79.4)
		YLLs	69 (7.8 to 287.6)	42.7 (7.3 to 179)	96.1 (6.5 to 403.3)	55.8 (6.7 to 220.8)	30.8 (5.9 to 129.5)	81.7 (6.1 to 320.4)	-19.1 (-51.8 to 59)	-28 (-65.9 to 78.1)	-14.9 (-52.5 to 78.6)
		YLDs	6.6 (0.8 to 26.9)	5.5 (0.9 to 22.9)	7.7 (0.5 to 30.6)	6 (0.7 to 24.3)	4.9 (0.8 to 20.7)	7.2 (0.5 to 27.7)	-7.8 (-43.7 to 82.4)	-10.7 (-58.3 to 104.5)	-6.1 (-42.7 to 95.7)
	Diet low in fiber	Deaths	2.3 (0.5 to 4.9)	2.1 (0.5 to 4.7)	2.4 (0.6 to 5.3)	4.1 (0.8 to 7.9)	3.6 (0.7 to 7.1)	4.5 (0.9 to 8.9)	78.7 (24 to 158)	70.1 (11.1 to 157.5)	87.5 (28 to 179.1)
		DALYs	52.5 (11.6 to 116.8)	48.2 (10.9 to 108.9)	56.6 (12.3 to 125.9)	97.8 (19.2 to 191.5)	87.3 (16.9 to 175.7)	108.4 (20.7 to 212.5)	86.4 (29.6 to 177.4)	81 (21.8 to 173.5)	91.4 (32.1 to 196.9)
		YLLs	47.2 (10.4 to 105.8)	42.2 (9.4 to 96.3)	52.1 (11.3 to 116.7)	86.4 (16.8 to 170.8)	74.1 (14.1 to 151.7)	98.7 (18.8 to 196.1)	83.1 (26 to 181.4)	75.6 (13.9 to 173.1)	89.6 (27.8 to 202.9)
		YLDs	5.3 (1.1 to 11.7)	6 (1.3 to 13.6)	4.6 (1 to 10)	11.4 (2.3 to 22.9)	13.2 (2.5 to 26.4)	9.7 (1.9 to 19.4)	116.2 (66 to 185.6)	118.9 (63.4 to 202.1)	112 (61.8 to 192.3)
	Diet low in fruits	Deaths	7.2 (3.3 to 12.2)	7 (3.1 to 12)	7.4 (3.3 to 12.9)	7 (3 to 12.1)	6.3 (2.7 to 10.9)	7.8 (3.4 to 13.4)	-2.2 (-30.3 to 33.7)	-9.8 (-38.6 to 27.4)	5.5 (-27 to 54.3)
		DALYs	190 (86.5 to 323.4)	182.8 (82.1 to 315.9)	196.9 (88.3 to 337.5)	180.1 (79.8 to 308.2)	160.7 (70.8 to 270.3)	199.3 (88.6 to 348.5)	-5.2 (-32.5 to 35.7)	-12.1 (-40.1 to 24.8)	1.2 (-30.2 to 52.6)
		YLLs	172.7 (79.4 to 296.9)	162.3 (75.6 to 286.1)	182.7 (83.1 to 318.9)	160.8 (71.5 to 274.1)	138.6 (60.7 to 236.9)	183 (81.1 to 322.5)	-6.9 (-35.6 to 35.2)	-14.6 (-42.9 to 25.6)	0.2 (-32.4 to 52.5)
		YLDs	17.3 (6 to 32.1)	20.4 (7 to 37.8)	14.2 (5.1 to 26.2)	19.3 (7.2 to 35.2)	22.2 (8.2 to 40.9)	16.3 (6.3 to 29.5)	11 (-6.5 to 54.1)	8.4 (-10.9 to 49.4)	14.4 (-4.9 to 62)
	Diet low in vegetables	Deaths	1.7 (0.4 to 3.8)	1.5 (0.4 to 3.5)	1.9 (0.5 to 4.2)	1.3 (0.4 to 2.7)	1.1 (0.3 to 2.4)	1.5 (0.4 to 3)	-24 (-47.2 to 10.4)	-27.5 (-53.7 to 16.6)	-20.4 (-47.4 to 25)
		DALYs	37.3 (8.5 to 90.4)	32.8 (7.7 to 82.2)	41.9 (9.3 to 103)	25.2 (6.8 to 57.1)	20.9 (5.9 to 48.7)	29.5 (7.5 to 67.6)	-32.6 (-52.6 to -0.8)	-36.2 (-58.8 to 0.1)	-29.6 (-52.9 to 9.4)
		YLLs	33.8 (7.7 to 83.9)	28.9 (6.8 to 74.3)	38.6 (8.5 to 96.5)	22.5 (6.1 to 51.9)	18.1 (5 to 42.2)	27.1 (6.9 to 63.4)	-33.3 (-53.8 to 1.3)	-37.4 (-61 to 1.9)	-29.9 (-53.9 to 11.8)
		YLDs	3.6 (0.7 to 8)	3.9 (0.8 to 8.7)	3.2 (0.6 to 7.2)	2.6 (0.7 to 5.8)	2.8 (0.7 to 6.6)	2.4 (0.6 to 5.4)	-26.2 (-40 to -5.9)	-27 (-47.9 to -0.1)	-25.8 (-44.9 to -0.5)
	Diet low in whole grains	Deaths	7 (3.3 to 9.9)	6.8 (3.3 to 9.8)	7.1 (3.2 to 10.3)	6.1 (2.5 to 8.6)	5.6 (2.3 to 8)	6.5 (2.8 to 9.5)	-13.1 (-32.6 to 7)	-17.9 (-39.5 to 5.7)	-7.6 (-31 to 20.6)
		DALYs	152.8 (67.6 to 216.5)	150.1 (68.7 to 213.4)	155.3 (67.2 to 230.1)	125.3 (49.3 to 183.9)	118.7 (45.3 to 174.5)	132.2 (52 to 196.2)	-18 (-36.3 to 2.2)	-20.9 (-41.2 to -0.6)	-14.8 (-36.5 to 13.1)
		YLLs	130.2 (58.1 to 187.1)	123.1 (56.4 to 180.5)	137.2 (60.1 to 206.7)	106.3 (42.2 to 158.3)	96 (36.7 to 143.5)	117 (46.3 to 175.5)	-18.4 (-39.3 to 4.8)	-22 (-44.1 to 2.9)	-14.7 (-39.1 to 17.4)
		YLDs	22.6 (9.6 to 34.1)	27.1 (11.9 to 40.9)	18.1 (7.4 to 27.8)	19.1 (7.1 to 29.2)	22.7 (8.3 to 35.2)	15.2 (5.7 to 23.6)	-15.8 (-28.2 to -9.1)	-16 (-30 to -7.3)	-15.8 (-26.1 to -6.4)
	High body-mass index	Deaths	42.9 (25.8 to 60.7)	41.9 (26.3 to 58.7)	43.7 (23.6 to 66)	35.9 (22.3 to 51)	31.7 (20.1 to 45.6)	40 (24.2 to 57.7)	-16.3 (-37 to 11.5)	-24.3 (-44.7 to 3.8)	-8.3 (-34.8 to 30.2)
		DALYs	1314.6 (836.1 to 1795.1)	1283 (859.3 to 1725.7)	1343.6 (774.9 to 1976.9)	1056.1 (692.1 to 1471.5)	942.4 (629.6 to 1297.7)	1167.9 (746.4 to 1657.2)	-19.7 (-39.5 to 7.5)	-26.5 (-45.5 to -0.2)	-13.1 (-37.1 to 23.1)
		YLLs	1191 (755.3 to 1637.6)	1134.8 (745.9 to 1556.8)	1245 (717.6 to 1857.3)	939.5 (602.3 to 1319.7)	807 (525 to 1129.3)	1070.3 (669.9 to 1539.3)	-21.1 (-42.6 to 8.5)	-28.9 (-49.7 to 0.1)	-14 (-39.3 to 24.6)
		YLDs	123.5 (73 to 180.5)	148.2 (89.9 to 211.4)	98.7 (55.2 to 147.4)	116.6 (73.1 to 165.9)	135.4 (85.9 to 191)	97.7 (59.8 to 142.2)	-5.6 (-13.7 to 5.1)	-8.7 (-16.4 to 1.5)	-1 (-13.5 to 15.7)
	High fasting plasma glucose	Deaths	34 (21 to 56.6)	33.6 (20 to 58.3)	34.1 (20.5 to 57.1)	44.2 (27.5 to 72.3)	39.4 (23.8 to 65.4)	49 (29 to 81.1)	30 (-9.4 to 81.5)	17.4 (-22.8 to 77.5)	43.7 (-4.3 to 112.5)
		DALYs	707.3 (469.3 to 1088.1)	693.1 (445.6 to 1086.5)	720.3 (453.9 to 1141.1)	884.4 (563.9 to 1343.1)	782.5 (498 to 1203.6)	988.7 (615.5 to 1511.8)	25 (-12.1 to 74.3)	12.9 (-24.9 to 66.5)	37.3 (-9.9 to 101.6)
		YLLs	641.4 (423.7 to 998.8)	615.5 (390.3 to 972.5)	666.6 (419.1 to 1055.7)	794.6 (499.2 to 1199)	680.2 (424.6 to 1046.4)	912.4 (565.7 to 1409.4)	23.9 (-14.9 to 75.9)	10.5 (-27.8 to 65.7)	36.9 (-11.2 to 105.2)
		YLDs	65.9 (38.9 to 109.8)	77.6 (44.7 to 128.9)	53.6 (30.9 to 91.2)	89.9 (52 to 140.8)	102.3 (58.7 to 165.7)	76.3 (43.2 to 122.1)	36.3 (6.9 to 74)	31.9 (-3.6 to 82.6)	42.2 (3.5 to 91.1)
	High LDL cholesterol	Deaths	19.2 (7.7 to 38.6)	18.8 (7 to 39.5)	19.4 (8.2 to 38.3)	19.5 (7.4 to 40.3)	18.5 (6.7 to 38.9)	20.4 (8 to 41)	1.6 (-22.3 to 25.6)	-1.7 (-27.9 to 25.9)	5.4 (-23 to 39)
		DALYs	436.6 (251.8 to 718.9)	430.9 (244 to 718)	441.4 (250.9 to 744.7)	415.3 (234.4 to 709.9)	403.5 (222.3 to 676.1)	427.2 (240 to 738.5)	-4.9 (-26.6 to 19.7)	-6.4 (-28.9 to 19.7)	-3.2 (-28.9 to 30.7)
		YLLs	369.3 (207.3 to 616)	349.7 (186.3 to 614)	388.1 (217.5 to 661.9)	349.3 (186.7 to 619.3)	323.5 (161 to 572.7)	375.5 (202.2 to 662)	-5.4 (-31.2 to 24.2)	-7.5 (-34.9 to 26.8)	-3.2 (-32 to 36)
		YLDs	67.3 (40.3 to 111.4)	81.2 (49 to 133.7)	53.3 (31.5 to 86.7)	66.1 (39.3 to 109.2)	80 (47.6 to 131.4)	51.8 (30.8 to 85.5)	-1.9 (-8.7 to 5.5)	-1.5 (-10.6 to 8.8)	-2.8 (-12.6 to 8.2)
	High systolic blood pressure	Deaths	91.1 (71.7 to 113.8)	88.6 (67.7 to 114.9)	93.1 (70.3 to 123.4)	83.6 (64.9 to 104.3)	75.2 (56.6 to 95.2)	92.2 (70.5 to 115.6)	-8.2 (-28.7 to 14.2)	-15.1 (-36.8 to 13.1)	-1 (-25.6 to 30.5)
		DALYs	2097 (1692.7 to 2588.2)	2017.6 (1584.6 to 2523.2)	2172.7 (1648 to 2884.8)	1822.7 (1421.5 to 2274.3)	1628.4 (1266.6 to 2040.3)	2018.5 (1530.1 to 2530)	-13.1 (-33.1 to 10)	-19.3 (-39.1 to 6.9)	-7.1 (-31 to 24.5)
		YLLs	1904 (1516.6 to 2362.5)	1791.8 (1379.5 to 2273.1)	2013.7 (1506 to 2694.2)	1633.8 (1244.7 to 2072.3)	1411.8 (1067.9 to 1800.6)	1859.1 (1385 to 2359.2)	-14.2 (-36 to 11.6)	-21.2 (-42.4 to 7.5)	-7.7 (-32.8 to 26)
		YLDs	193 (137.2 to 254.6)	225.9 (162 to 302.4)	159 (110.6 to 210.5)	188.9 (136.2 to 244.9)	216.6 (155.2 to 282.5)	159.4 (113.3 to 208.2)	-2.1 (-11.2 to 9)	-4.1 (-17.1 to 11.9)	0.3 (-10.1 to 13.2)
	High temperature	Deaths	4 (0.5 to 8.8)	3.9 (0.5 to 8.5)	4.2 (0.6 to 9.5)	4.2 (0.9 to 8.4)	3.8 (0.8 to 7.7)	4.6 (1 to 9.2)	4 (-35.8 to 79.3)	-1.6 (-39.6 to 66.2)	10 (-33.3 to 93.3)
		YLLs	79.7 (10.4 to 177.4)	73.9 (9.3 to 164.4)	85.4 (11.6 to 193.4)	76.5 (16.3 to 156.3)	66.6 (13.6 to 133.6)	86.5 (19 to 176.6)	-4 (-41.6 to 62.6)	-9.9 (-45.1 to 52.9)	1.3 (-37.1 to 79.7)
	Household air pollution from solid fuels	Deaths	15 (7.1 to 25.3)	16.3 (8.1 to 27.2)	13.4 (5.9 to 23.8)	0.1 (0 to 0.2)	0.1 (0 to 0.2)	0.1 (0 to 0.2)	-99.3 (-99.7 to -98.4)	-99.3 (-99.7 to -98.5)	-99.3 (-99.7 to -98.4)
		DALYs	363.6 (173.3 to 611.3)	396.7 (201.8 to 655.1)	328.4 (144.8 to 577.1)	2.3 (0.8 to 5.1)	2.5 (0.9 to 5.5)	2.1 (0.7 to 4.8)	-99.4 (-99.7 to -98.5)	-99.4 (-99.7 to -98.5)	-99.4 (-99.8 to -98.4)
		YLLs	327.7 (155.1 to 554.2)	350.1 (174 to 578.4)	303.4 (130.9 to 533.4)	2 (0.7 to 4.6)	2.2 (0.8 to 4.8)	1.9 (0.6 to 4.4)	-99.4 (-99.8 to -98.5)	-99.4 (-99.8 to -98.5)	-99.4 (-99.8 to -98.4)
		YLDs	35.9 (17.2 to 61.1)	46.5 (23 to 77.9)	25 (10.9 to 45.4)	0.3 (0.1 to 0.6)	0.4 (0.1 to 0.8)	0.2 (0.1 to 0.4)	-99.3 (-99.7 to -98.3)	-99.2 (-99.7 to -98.3)	-99.3 (-99.7 to -98.4)
	Kidney	Deaths	14.4 (10.5 to 18.7)	14.8 (10.4 to 19.6)	13.9 (10 to 18.3)	17.1 (11.6 to 22.6)	15.8 (10.4 to 21.4)	18.6 (12.9 to 24.1)	19.1 (-8.3 to 48.3)	6.3 (-21.3 to 38.7)	33.9 (-1.1 to 76.9)

Location	Risk factor	Measure	1990			2019			% Change (1990 to 2019)		
			Both	Female	Male	Both	Female	Male	Both	Female	Male
	dysfunction	DALYs	325.7 (254.1 to 403.7)	338.5 (256.9 to 427.2)	312.1 (234.9 to 401.6)	367.2 (272.9 to 460.4)	345.2 (255 to 440.3)	390.2 (288 to 490.5)	12.7 (-11.3 to 40.4)	2 (-22.3 to 30.7)	25 (-7.3 to 64.4)
		YLLs	292.1 (226.8 to 367.1)	296.5 (223.7 to 377)	287.2 (213.5 to 372.9)	326.5 (239.3 to 416.1)	296.2 (216 to 384.3)	358.3 (262.5 to 459.4)	11.8 (-14.9 to 43)	-0.1 (-27 to 32.6)	24.8 (-9.7 to 68.9)
		YLDs	33.6 (23.1 to 44.8)	42 (28.8 to 56.2)	24.9 (16.9 to 33.3)	40.7 (27.8 to 54.5)	49 (32.9 to 66.1)	31.9 (21.8 to 42.6)	21.1 (12.2 to 30.6)	16.7 (6 to 28.5)	28.3 (15.2 to 43)
	Lead exposure	Deaths	6.9 (3.6 to 10.7)	5.2 (2.3 to 8.6)	8.8 (4.8 to 13.6)	5.8 (3.1 to 9)	4.1 (1.8 to 6.8)	7.6 (4.2 to 11.4)	-16.5 (-34.6 to 4)	-20.4 (-40.5 to 4.8)	-13.1 (-33.9 to 13.9)
		DALYs	159.9 (79.3 to 255.9)	116.3 (47.2 to 194.5)	204.7 (109.9 to 321.5)	113.9 (55.8 to 181.6)	79 (31 to 135.7)	150.8 (80.1 to 232.6)	-28.8 (-45.5 to -9.4)	-32.1 (-49.8 to -12.3)	-26.3 (-45.3 to -1.9)
		YLLs	146.2 (72.3 to 235.9)	103.6 (41.4 to 172.8)	190 (101.1 to 300.5)	103.3 (49.8 to 166.4)	69.1 (27 to 118.1)	139.6 (73.3 to 217.5)	-29.3 (-47.4 to -8.5)	-33.3 (-51.9 to -11.7)	-26.5 (-46.4 to -0.1)
		YLDs	13.7 (6.1 to 22.5)	12.7 (4.8 to 22.2)	14.7 (7.7 to 23.3)	10.6 (4.5 to 18.2)	9.9 (3.4 to 18.1)	11.2 (5.4 to 18.4)	-23 (-33.9 to -15.2)	-22.5 (-36.1 to -10.5)	-23.7 (-35.5 to -13.4)
	Low physical activity	Deaths	9.6 (2.3 to 19.9)	9.5 (2.3 to 19.9)	9.6 (1.9 to 20)	10 (2.3 to 20.6)	9.6 (2.4 to 19.3)	10.5 (2.2 to 21.5)	4.5 (-17 to 30.5)	0.9 (-22.3 to 34.2)	9 (-15.4 to 47.9)
		DALYs	154.9 (32.9 to 349.8)	154.3 (37 to 345.9)	155 (28.1 to 358.3)	155.5 (34.2 to 351.1)	150.9 (33.9 to 329.1)	160.3 (30.5 to 363.8)	0.4 (-19.5 to 25.3)	-2.2 (-23.3 to 28.2)	3.4 (-19.9 to 39.7)
		YLLs	135.7 (29.1 to 301.6)	131.4 (31.5 to 296.3)	139.8 (24.3 to 323.1)	136.3 (29.5 to 305.4)	127.7 (28.9 to 280.4)	145.6 (28.1 to 324.5)	0.4 (-21.8 to 28.1)	-2.8 (-26.8 to 32)	4.1 (-21.2 to 44.5)
		YLDs	19.1 (3.6 to 45.4)	22.9 (4.6 to 54.3)	15.1 (2.6 to 37.4)	19.1 (3.7 to 44.2)	23.2 (4.8 to 54.2)	14.7 (2.5 to 35.1)	0 (-8.7 to 13.8)	1.6 (-10.2 to 19.8)	-3.2 (-14.9 to 14.4)
	Low temperature	Deaths	7.9 (2.2 to 14.1)	7.6 (2.1 to 13.1)	8.2 (2.1 to 15.1)	7.3 (1.8 to 13.1)	6.7 (1.7 to 11.9)	8 (2 to 14.3)	-7.1 (-29.4 to 18.9)	-12.1 (-34.7 to 15.8)	-1.9 (-28.1 to 31)
		YLLs	156.2 (42.2 to 273.9)	144.7 (41 to 251.4)	167.6 (43.5 to 310.5)	133.7 (33.9 to 240.9)	116.5 (28.8 to 210.4)	151.3 (38 to 272.7)	-14.4 (-36.6 to 13)	-19.5 (-41.6 to 8.9)	-9.7 (-35.2 to 24.3)
	Secondhand smoke	Deaths	7.2 (5.2 to 9.5)	7.7 (5.5 to 10.3)	6.6 (4.7 to 9)	5.8 (4 to 7.7)	5.7 (4 to 7.7)	5.8 (4 to 7.9)	-19.7 (-38 to 1.1)	-26.1 (-45.7 to -2)	-11.6 (-34.2 to 19.1)
		DALYs	178.1 (128.6 to 233.6)	198.7 (140.9 to 262.3)	158 (110.1 to 220.1)	134.8 (92.6 to 184.5)	140 (96.8 to 188.9)	130.2 (87.7 to 181.6)	-24.3 (-42.3 to -3)	-29.5 (-49.5 to -4.7)	-17.6 (-39.8 to 12.4)
		YLLs	167.3 (120 to 221)	184.1 (129.5 to 246.3)	150.9 (104.8 to 209.5)	125.4 (85.1 to 172.5)	127.4 (87 to 175.5)	123.9 (82.5 to 173.9)	-25.1 (-43.8 to -2.3)	-30.8 (-51.5 to -4.2)	-17.9 (-41 to 13.5)
		YLDs	10.8 (7.1 to 14.9)	14.6 (9.6 to 20.2)	7.1 (4.6 to 9.9)	9.4 (6.1 to 12.9)	12.6 (8.2 to 17.1)	6.3 (4.1 to 8.8)	-12.8 (-20 to -4.9)	-13.8 (-22.3 to -4.7)	-11 (-22.8 to 3)
	Smoking	Deaths	28.7 (23.1 to 35.5)	11.5 (8.1 to 16.1)	46.9 (36.8 to 59.4)	20.5 (16.1 to 25)	7.7 (5.7 to 10.2)	34.4 (27.2 to 41.4)	-28.7 (-46.3 to -6.7)	-33.3 (-55.5 to -0.8)	-26.7 (-45.3 to -2.2)
		DALYs	765.9 (616.4 to 938.5)	305.5 (219.4 to 420)	1235.3 (971.1 to 1553.1)	523.7 (409.6 to 649.5)	196.5 (143.9 to 261.8)	860.4 (674 to 1060.4)	-31.6 (-48.8 to -9.9)	-35.7 (-56.1 to -6)	-30.3 (-48.2 to -5.6)
		YLLs	701.4 (555.4 to 876.7)	270 (190.6 to 373.9)	1141.6 (879.8 to 1460.6)	474.2 (361.2 to 597.9)	169.2 (122.1 to 228.8)	788.7 (606 to 992.5)	-32.4 (-50.4 to -9)	-37.3 (-58.6 to -5.8)	-30.9 (-49.8 to -4.3)
		YLDs	64.5 (46 to 81.9)	35.5 (22.9 to 49.9)	93.8 (67 to 121)	49.5 (35.2 to 63.4)	27.3 (18.1 to 38.1)	71.7 (50.9 to 91.4)	-23.3 (-32 to -13.6)	-23.1 (-42.7 to 3)	-23.5 (-31.1 to -15.7)
Jordan	All risk factors	Deaths	127 (103.3 to 151.1)	150.8 (119.4 to 183.3)	102.7 (75.1 to 125.1)	64.5 (52.5 to 76.8)	70.7 (55.9 to 86.9)	59.2 (42.2 to 74.3)	-49.2 (-59 to -38.3)	-53.1 (-63.6 to -39.2)	-42.3 (-57.3 to -23.8)
		DALYs	2527.4 (2119.7 to 2954)	2950.5 (2455.2 to 3496.7)	2101.4 (1563.8 to 2514.3)	1271.7 (1063 to 1477.1)	1367.2 (1135.6 to 1630)	1186 (912.8 to 1449.4)	-49.7 (-58.3 to -40.2)	-53.7 (-63.2 to -41.9)	-43.6 (-57.2 to -27.6)
		YLLs	2205.7 (1828.2 to 2607)	2564.9 (2065.7 to 3109.3)	1842.8 (1318.3 to 2260.4)	1023.3 (847.5 to 1213.8)	1070.4 (845.1 to 1334.9)	981.2 (715.4 to 1234.3)	-53.6 (-62.9 to -43.1)	-58.3 (-67.9 to -45.2)	-46.8 (-61.5 to -29.2)
		YLDs	321.7 (235.1 to 407.7)	385.5 (281.6 to 489.7)	258.7 (187.5 to 329.9)	248.4 (181.1 to 316.2)	296.8 (216.6 to 378)	204.8 (147.3 to 263.9)	-22.8 (-27.8 to -17.8)	-23 (-28.9 to -17)	-20.8 (-28.9 to -11.8)
	Alcohol use	Deaths	0.1 (-0.2 to 0.4)	-0.2 (-0.4 to 0)	0.4 (-0.1 to 1)	0.1 (-0.1 to 0.3)	-0.1 (-0.3 to 0)	0.3 (-0.1 to 0.6)	-5.7 (-672.3 to 545.7)	-41.4 (-124.6 to 67.4)	-31.1 (-260.7 to 239.1)
		DALYs	4.4 (-3 to 13.7)	-3.5 (-7.9 to 1.6)	12 (-1.1 to 28)	3.5 (-1.6 to 9.2)	-2.2 (-4.7 to 0.6)	8.5 (-0.6 to 18)	-21.7 (-578.9 to 398.7)	-38.5 (-200.6 to 109.5)	-29.5 (-174.7 to 191.9)
		YLLs	4.3 (-2.2 to 12.5)	-2.7 (-6.7 to 1.8)	11 (-0.3 to 25.8)	3.1 (-1 to 7.8)	-1.5 (-3.5 to 0.6)	7.2 (0 to 15.2)	-27.8 (-476.8 to 459.5)	-45.1 (-178.3 to 153.6)	-34.6 (-125.7 to 201.5)
		YLDs	0.1 (-0.8 to 1.2)	-0.8 (-1.5 to -0.1)	1 (-0.7 to 3)	0.4 (-0.6 to 1.4)	-0.7 (-1.3 to 0)	1.3 (-0.4 to 3.1)	201.4 (-960.2 to 1322.7)	-15.4 (-86.4 to 55.8)	29 (-646.1 to 886.3)
	Ambient particulate matter pollution	Deaths	28 (21.1 to 35.6)	32.6 (24.2 to 41.4)	23.3 (16.3 to 29.9)	14.6 (11.2 to 18.4)	15.6 (11.7 to 20.1)	13.6 (9.5 to 17.9)	-48 (-58.8 to -35.8)	-52 (-63 to -36.6)	-41.6 (-57.5 to -21.9)
		DALYs	627.4 (487.2 to 773.8)	724.5 (558.5 to 905.9)	530.9 (391.1 to 674)	327.8 (259 to 404.2)	348.8 (268.1 to 437.3)	308.7 (229.7 to 398.6)	-47.7 (-57.5 to -36.8)	-51.9 (-61.9 to -38.9)	-41.8 (-56.4 to -24.2)
		YLLs	541.8 (411.3 to 677.9)	622.6 (464 to 793.9)	461.3 (332.4 to 592.3)	258.1 (197 to 326.5)	265.7 (197 to 345.6)	251 (175.9 to 333.3)	-52.4 (-62.7 to -40.2)	-57.3 (-67.4 to -42.9)	-45.6 (-61.3 to -26)
		YLDs	85.5 (59 to 113.5)	101.9 (70.4 to 135.7)	69.6 (47.9 to 92.6)	69.7 (48.6 to 91.9)	83 (58 to 109)	57.7 (39.8 to 77.2)	-18.5 (-25 to -11.7)	-18.5 (-24.9 to -11.4)	-17 (-26.2 to -7.6)
	Diet high in red meat	Deaths	4.1 (1.4 to 6.6)	4.8 (1.6 to 7.8)	3.4 (1.2 to 5.5)	2 (0.7 to 3.1)	2.1 (0.7 to 3.4)	1.9 (0.6 to 3.1)	-52.4 (-63.2 to -38.9)	-56.6 (-68.7 to -39.5)	-45.9 (-62 to -25.5)
		DALYs	102.5 (36.3 to 159.2)	119.1 (41.3 to 186.5)	86.5 (30.3 to 136.6)	48.8 (16.8 to 77.1)	51.5 (17.4 to 83.3)	46.5 (15.8 to 75)	-52.4 (-62.7 to -41)	-56.8 (-67.8 to -44.1)	-46.3 (-61.1 to -28.5)
		YLLs	87.4 (31.9 to 138.5)	100.9 (36 to 160.3)	74.2 (25.4 to 119.1)	37.3 (13.2 to 58.5)	37.8 (14.2 to 62.1)	36.9 (12.5 to 60.8)	-57.3 (-67.4 to -45.5)	-62.6 (-72.8 to -48)	-50.3 (-65.4 to -30.5)
		YLDs	15.1 (4.1 to 25)	18.1 (4.9 to 30.2)	12.3 (3.3 to 20.4)	11.5 (3.1 to 19.3)	13.7 (3.6 to 23.2)	9.6 (2.6 to 16.2)	-23.9 (-30.6 to -15.7)	-24.6 (-33.8 to -13.7)	-21.5 (-32.1 to -10.5)
	Diet high in sodium	Deaths	2.3 (0.4 to 9)	2 (0.5 to 7.9)	2.5 (0.3 to 10.3)	1.1 (0.2 to 4.4)	0.9 (0.2 to 3.5)	1.4 (0.1 to 5.6)	-49.9 (-74 to -13.1)	-56.9 (-78.6 to -5)	-45.4 (-70.1 to 30.1)
		DALYs	51.9 (7.6 to 208.2)	44.6 (9 to 185.4)	59.1 (4.9 to 237.7)	26.3 (3.8 to 103.3)	19.3 (4.1 to 79.2)	32.6 (2.8 to 127.6)	-49.4 (-72.5 to -11.6)	-56.8 (-80.8 to 0.3)	-44.9 (-68.3 to 16.9)
		YLLs	45.1 (6.6 to 180.3)	38.5 (7.8 to 159.1)	51.6 (4.3 to 205.9)	21 (3 to 83.1)	14.9 (3.2 to 60.5)	26.5 (2.3 to 104.3)	-53.4 (-75 to -18.1)	-61.3 (-82.6 to -7.7)	-48.5 (-72.2 to 17.1)
		YLDs	6.8 (0.9 to 28.4)	6 (1.1 to 25.2)	7.5 (0.6 to 30.6)	5.3 (0.7 to 20.8)	4.4 (0.9 to 18.2)	6 (0.4 to 23.7)	-22.8 (-56.9 to 34.2)	-27.6 (-68.2 to 57.6)	-20.1 (-48 to 52.1)
	Diet low in fiber	Deaths	5.5 (1.2 to 10.3)	6.6 (1.4 to 12.2)	4.5 (1 to 8.4)	2.1 (0.5 to 4.1)	2.2 (0.5 to 4.5)	1.9 (0.4 to 3.8)	-62.5 (-72.1 to -51.9)	-65.7 (-76.8 to -52.7)	-57.2 (-70.7 to -40.1)
		DALYs	122 (25.4 to 223.6)	142.7 (28.7 to 268.5)	101.6 (22.1 to 190.5)	46.7 (9.6 to 89.8)	49.8 (9.8 to 98.7)	43.9 (8.7 to 86.6)	-61.7 (-70.2 to -52.6)	-65.1 (-74.2 to -54.6)	-56.8 (-68.4 to -42.5)
		YLLs	104.2 (21.8 to 192.5)	121.3 (24.8 to 230.3)	87.2 (18.1 to 165)	35.8 (7.3 to 69.9)	36.9 (7.4 to 75)	34.9 (7 to 68.8)	-65.6 (-74.1 to -56.3)	-69.6 (-78.6 to -58)	-60 (-72.5 to -44.1)
		YLDs	17.8 (3.6 to 33.7)	21.4 (4.2 to 40.7)	14.4 (3 to 26.9)	10.9 (2.1 to 21.7)	13 (2.5 to 26)	9 (1.7 to 17.8)	-38.9 (-48.1 to -31.1)	-39.3 (-51.1 to -29.3)	-37 (-50.1 to -26.5)
	Diet low in fruits	Deaths	6.3 (2.6 to 10.9)	7.5 (3.1 to 13.2)	5.1 (2 to 9.1)	3.1 (1.2 to 5.5)	3.3 (1.3 to 5.9)	2.8 (1.1 to 5.1)	-51.7 (-62.5 to -38.9)	-56 (-67.4 to -40.3)	-44.6 (-60.6 to -24.8)
		DALYs	147.3 (60.9 to 254.5)	174.3 (72.8 to 303.2)	120.9 (48.3 to 212.8)	72.8 (30.1 to 125.6)	78.1 (31.7 to 137.7)	68 (26.7 to 120.5)	-50.6 (-60.8 to -39.6)	-55.2 (-66.1 to -41.7)	-43.8 (-58.4 to -25.5)
		YLLs	127.3 (54.3 to 220.1)	150.1 (64.8 to 257.7)	105.1 (43 to 186.1)	56.7 (23.9 to 100.1)	58.8 (24.3 to 103.1)	54.8 (21.9 to 98)	-55.5 (-65.4 to -43.5)	-60.8 (-71.2 to -46.6)	-47.8 (-62.9 to -28.2)
		YLDs	20 (6.5 to 37.9)	24.3 (7.8 to 46)	15.9 (5.1 to 30.2)	16.1 (5.5 to 30)	19.4 (6.5 to 36.3)	13.2 (4.5 to 25)	-19.5 (-26 to -5.4)	-20.2 (-27.8 to -4.9)	-16.9 (-26.1 to -0.2)

Location	Risk factor	Measure	1990			2019			% Change (1990 to 2019)		
			Both	Female	Male	Both	Female	Male	Both	Female	Male
	Diet low in vegetables	Deaths	3.1 (1 to 5.6)	3.6 (1.1 to 6.5)	2.7 (0.8 to 4.8)	1.3 (0.4 to 2.3)	1.3 (0.4 to 2.4)	1.2 (0.3 to 2.3)	-59.2 (-70.3 to -47.8)	-62.9 (-75.3 to -48.8)	-53.8 (-68.3 to -36.5)
		DALYs	67.1 (19.6 to 123.4)	75.9 (21.4 to 140.1)	58.4 (16.6 to 109.3)	27.7 (7.5 to 51.2)	28.4 (7.4 to 52.5)	27.1 (7 to 51.5)	-58.7 (-69.2 to -47.5)	-62.6 (-73.5 to -49.7)	-53.6 (-67.5 to -37.3)
		YLLs	58.1 (16.9 to 110)	65.4 (18.4 to 122.7)	50.9 (14.2 to 96.7)	21.8 (5.9 to 40.7)	21.5 (5.9 to 40)	22 (5.8 to 42.2)	-62.5 (-72.6 to -51.2)	-67.1 (-77.7 to -53.3)	-56.8 (-70.7 to -38.7)
		YLDs	9 (1.9 to 17)	10.5 (2.2 to 20)	7.5 (1.7 to 14.2)	5.9 (1.2 to 11.5)	6.8 (1.4 to 13.4)	5.1 (1.1 to 10)	-34 (-46 to -24.4)	-34.8 (-48.7 to -24)	-31.8 (-47.3 to -18.9)
	Diet low in whole grains	Deaths	9.6 (5.6 to 12.9)	11.4 (6.6 to 15.4)	7.7 (4.3 to 10.5)	5 (3 to 6.7)	5.6 (3.3 to 7.6)	4.6 (2.5 to 6.3)	-47.6 (-58.5 to -35.9)	-51.2 (-62.2 to -36.3)	-40.9 (-56 to -22.6)
		DALYs	190.2 (107.2 to 253.4)	222 (125.4 to 298)	158 (87.4 to 213.6)	101.4 (57.6 to 136.3)	110.4 (62 to 148.5)	93.2 (51.7 to 131)	-46.7 (-56.5 to -37)	-50.3 (-60.4 to -38.5)	-41 (-54.5 to -24.5)
		YLLs	157.7 (88.5 to 212.3)	182.9 (102.9 to 250.2)	131.9 (72.4 to 181.6)	76.5 (44 to 103.6)	80.6 (45.7 to 111.1)	72.9 (39.2 to 103.9)	-51.5 (-62 to -39.9)	-55.9 (-66.3 to -41.6)	-44.8 (-59.9 to -25.3)
		YLDs	32.5 (17.1 to 46.9)	39.2 (20.9 to 56.9)	26.1 (13.7 to 37.7)	24.8 (13.1 to 35.8)	29.8 (15.7 to 43.3)	20.4 (10.5 to 29.6)	-23.7 (-29.1 to -18.4)	-23.9 (-29.8 to -17.2)	-21.8 (-30.8 to -12.9)
	High body-mass index	Deaths	31.6 (19.3 to 44.6)	40.2 (25.3 to 56.4)	23 (11.9 to 34.9)	18.2 (11.1 to 25.6)	19.9 (12.1 to 28.8)	16.8 (9.7 to 24.7)	-42.3 (-55.5 to -25.4)	-50.5 (-63.5 to -32.9)	-27 (-49.1 to 7.6)
		DALYs	893.3 (584.2 to 1198.1)	1135.8 (764.9 to 1513)	658.3 (374.9 to 959.3)	522 (358.2 to 687.1)	569.1 (393 to 758.7)	478.9 (313.2 to 662.4)	-41.6 (-53.3 to -26)	-49.9 (-60.9 to -35.5)	-27.3 (-47 to 2.3)
		YLLs	765.9 (489.9 to 1041.1)	968.8 (637.1 to 1319.6)	568.4 (308 to 834.6)	401 (266 to 537.8)	420.9 (278 to 574.5)	382.1 (230.8 to 545.1)	-47.6 (-59.1 to -31.5)	-56.6 (-68 to -41.2)	-32.8 (-53.3 to -1.4)
		YLDs	127.5 (77.7 to 185.1)	167 (105.9 to 236)	89.9 (51.4 to 137.4)	120.9 (79.4 to 166.9)	148.2 (99.1 to 203.1)	96.8 (60.2 to 136.9)	-5.1 (-14.6 to 8.5)	-11.3 (-20 to 1)	7.7 (-8.6 to 35.8)
	High fasting plasma glucose	Deaths	38.4 (21.5 to 68.8)	48.3 (26.1 to 93)	28 (15.6 to 48.9)	22.5 (12.3 to 40.8)	24.4 (13 to 46.1)	20.8 (11.2 to 37.4)	-41.3 (-56.9 to -21.1)	-49.4 (-65.6 to -26.4)	-25.7 (-53.8 to 18.4)
		DALYs	726.3 (442.4 to 1178.8)	888.3 (520.2 to 1527)	554.4 (328.5 to 885.6)	432.5 (257.9 to 673.1)	458.3 (266.9 to 746.4)	407.5 (241.2 to 655.3)	-40.4 (-55.6 to -20.8)	-48.4 (-64.7 to -26.9)	-26.5 (-53.1 to 12.6)
		YLLs	639 (387 to 1044.8)	779.9 (450.7 to 1354)	489.3 (287.2 to 784.8)	353.5 (209.8 to 562.8)	365.9 (208.7 to 613.4)	340.9 (194.1 to 551.6)	-44.7 (-59.4 to -24.8)	-53.1 (-68.8 to -31.9)	-30.3 (-57.2 to 10.6)
		YLDs	87.3 (48.7 to 148.8)	108.4 (56.4 to 191.3)	65.1 (34.9 to 113.5)	79.1 (43.5 to 128.7)	92.4 (49.6 to 156.7)	66.5 (36 to 109.7)	-9.4 (-27.5 to 13.4)	-14.8 (-36.5 to 16)	2.1 (-26.9 to 42.6)
	High LDL cholesterol	Deaths	22.5 (7.8 to 46.5)	27.9 (9.3 to 58.7)	17 (6.3 to 35.4)	12.6 (4.1 to 26.6)	14.6 (4.4 to 32.2)	10.9 (3.7 to 23.6)	-44.1 (-56.9 to -32)	-47.7 (-61.9 to -32.9)	-36.3 (-54.5 to -16.2)
		DALYs	468.1 (255.6 to 794.7)	567.3 (299.9 to 985)	370.2 (196.8 to 645.7)	264.8 (137.4 to 468.4)	298.8 (147.9 to 540.1)	234.5 (123.5 to 409)	-43.4 (-53.7 to -32.7)	-47.3 (-58.1 to -35.2)	-36.6 (-51.3 to -18.4)
		YLLs	381.3 (191.4 to 679.1)	458.8 (218.7 to 847.8)	303.9 (151.9 to 556.3)	194.5 (89.5 to 364.2)	211.9 (91 to 414.1)	178.7 (85 to 333.5)	-49 (-60.8 to -36.8)	-53.8 (-66.7 to -39.7)	-41.2 (-58.5 to -19.8)
		YLDs	86.8 (51 to 140.9)	108.4 (64.2 to 175)	66.3 (39.3 to 109.1)	70.4 (40.4 to 114.6)	86.9 (49.8 to 141.2)	55.8 (32.5 to 90.7)	-19 (-25.1 to -12.5)	-19.9 (-27.6 to -11.9)	-15.8 (-25.9 to -5.1)
	High systolic blood pressure	Deaths	72 (53.9 to 92.3)	89.7 (64.8 to 120)	54 (36.3 to 72.4)	36.5 (27.4 to 47.9)	41.4 (28.9 to 55.8)	32.3 (21.8 to 44.3)	-49.3 (-61 to -35.2)	-53.8 (-67.4 to -36)	-40.2 (-58.3 to -17.4)
		DALYs	1485.8 (1168.1 to 1832.9)	1808.8 (1386 to 2308.3)	1159.9 (840.7 to 1489.5)	752.3 (585.1 to 924.9)	825.7 (615.4 to 1057.1)	685.2 (493.8 to 877.9)	-49.4 (-60.1 to -37.2)	-54.4 (-66.2 to -38.6)	-40.9 (-57.3 to -21)
		YLLs	1298.3 (1005 to 1627.2)	1575.8 (1180.7 to 2036.8)	1017.6 (714 to 1318.7)	605.6 (465.2 to 763.2)	649 (472.2 to 862.2)	565.9 (386.4 to 744.8)	-53.4 (-64.7 to -40.7)	-58.8 (-70.6 to -42.7)	-44.4 (-61.2 to -22.8)
		YLDs	187.5 (130.4 to 249.5)	233 (159.6 to 312.9)	142.3 (95.9 to 192.6)	146.7 (101.3 to 197)	176.7 (119.3 to 238.4)	119.3 (82.5 to 161.6)	-21.7 (-31.6 to -8.9)	-24.2 (-37.6 to -5.7)	-16.1 (-29.5 to 1.9)
	High temperature	Deaths	0.3 (-0.5 to 1.8)	0.4 (-0.6 to 2.2)	0.3 (-0.4 to 1.6)	0.4 (-0.3 to 1.3)	0.4 (-0.3 to 1.4)	0.4 (-0.2 to 1.2)	24 (-609.2 to 760.5)	15.1 (-580.7 to 601.1)	40.1 (-669.2 to 943.2)
		YLLs	5.5 (-9.2 to 31.2)	6.4 (-10.7 to 35.2)	4.6 (-7.6 to 27.5)	6.2 (-4 to 19.8)	6.5 (-4.2 to 20.8)	5.9 (-3.7 to 19.4)	13.1 (-559.6 to 703)	2.4 (-523.7 to 520.3)	29.2 (-622.2 to 835.1)
	Household air pollution from solid fuels	Deaths	1 (0.4 to 2.1)	1.4 (0.6 to 2.7)	0.6 (0.2 to 1.4)	0 (0 to 0)	0 (0 to 0)	0 (0 to 0)	-99.3 (-99.7 to -98.1)	-99.3 (-99.7 to -98.2)	-99.1 (-99.7 to -97.8)
		DALYs	22.3 (9.7 to 46.3)	30.2 (13.4 to 59.5)	14.5 (5.4 to 31.9)	0.2 (0.1 to 0.4)	0.2 (0.1 to 0.5)	0.1 (0 to 0.3)	-99.3 (-99.7 to -98.1)	-99.3 (-99.7 to -98.2)	-99.2 (-99.7 to -97.8)
		YLLs	19.3 (8.3 to 39.2)	26 (11.2 to 52.6)	12.6 (4.6 to 27.9)	0.1 (0 to 0.3)	0.2 (0.1 to 0.4)	0.1 (0 to 0.2)	-99.3 (-99.7 to -98.2)	-99.4 (-99.8 to -98.4)	-99.2 (-99.7 to -97.9)
		YLDs	3.1 (1.2 to 6.3)	4.3 (1.7 to 8.8)	1.9 (0.7 to 4.2)	0 (0 to 0.1)	0.1 (0 to 0.1)	0 (0 to 0.1)	-98.8 (-99.6 to -97)	-98.8 (-99.6 to -97)	-98.8 (-99.6 to -97)
	Kidney dysfunction	Deaths	13.4 (9 to 17.9)	17.3 (11.1 to 23.7)	9.3 (6.1 to 12.7)	8.8 (5.3 to 12.3)	9.9 (5.5 to 14.2)	7.9 (4.6 to 11.5)	-34.2 (-50.6 to -18.3)	-43.1 (-58.8 to -24.2)	-15.4 (-41.2 to 14.6)
		DALYs	283.1 (211 to 359.7)	365.2 (266.9 to 470.6)	199.1 (139.4 to 258.2)	187.6 (135.7 to 240)	211.2 (144.4 to 277.2)	165.8 (113.9 to 225.7)	-33.7 (-47.2 to -20.5)	-42.2 (-55.1 to -26.3)	-16.7 (-37.6 to 8.6)
		YLLs	245.7 (181.1 to 317.3)	315.5 (227.1 to 410.2)	174.1 (120 to 227.6)	150.6 (103.9 to 196.1)	164.7 (108.3 to 221.8)	137.6 (87.7 to 192.9)	-38.7 (-53.1 to -23.8)	-47.8 (-61.9 to -30)	-21 (-45.3 to 7.4)
		YLDs	37.4 (25.6 to 50.6)	49.8 (34.1 to 67.2)	25 (17 to 33.9)	36.9 (24.9 to 50.6)	46.5 (31.6 to 63.6)	28.2 (18.8 to 39.2)	-1.1 (-10.3 to 8)	-6.6 (-15.7 to 3)	13 (-3.1 to 29.1)
	Lead exposure	Deaths	5 (2.4 to 8.1)	5.1 (2 to 8.6)	5 (2.6 to 7.8)	2.3 (1 to 3.8)	2.1 (0.8 to 3.9)	2.5 (1.2 to 4.1)	-54.5 (-65.3 to -43.9)	-58.8 (-70.5 to -44.9)	-50.3 (-64.2 to -33)
		DALYs	105.6 (49.2 to 167.5)	104 (39.4 to 175.1)	106.9 (55.4 to 166.6)	41.4 (16.9 to 70)	37 (11.7 to 67.3)	45.6 (20.1 to 75.3)	-60.7 (-71 to -51.7)	-64.5 (-75.4 to -53.5)	-57.3 (-69.3 to -44.4)
		YLLs	92.5 (42.9 to 146.4)	90.7 (34.5 to 153.6)	94 (48.4 to 149.4)	34.4 (14.3 to 58.3)	29.9 (9.6 to 55.3)	38.6 (17.4 to 64.4)	-62.8 (-72.7 to -53)	-67.1 (-77.7 to -55.7)	-59 (-71.8 to -44.6)
		YLDs	13.1 (5.4 to 22.6)	13.3 (4.5 to 24)	12.9 (6.1 to 21.7)	7 (2.4 to 13.2)	7.1 (1.8 to 14)	7 (2.8 to 12.8)	-46.2 (-59.1 to -37.6)	-46.7 (-64 to -37.4)	-45.5 (-59.6 to -35)
	Low physical activity	Deaths	7.9 (1.7 to 18.4)	9.9 (2.3 to 22.6)	5.9 (1 to 14.4)	4.9 (1.1 to 10.6)	5.7 (1.3 to 12.6)	4.2 (0.8 to 9.4)	-38 (-52.5 to -17.1)	-42.1 (-57.3 to -18.3)	-28.6 (-49.5 to 5.7)
		DALYs	118.9 (23.8 to 298.4)	147 (32.1 to 356)	90.2 (13.8 to 236)	75 (15.3 to 171.4)	87.2 (19 to 201.8)	64.2 (11.1 to 153.8)	-36.9 (-51.2 to -17.5)	-40.7 (-54.8 to -20.1)	-28.8 (-48.1 to 2.2)
		YLLs	102.4 (21.1 to 255.1)	126.3 (28 to 300.2)	78.1 (12 to 204.1)	60.2 (12.3 to 138.4)	68.4 (15 to 156.6)	53.1 (9.4 to 128.2)	-41.2 (-56.1 to -20.9)	-45.9 (-60 to -23.4)	-32 (-52.8 to 0.8)
		YLDs	16.4 (2.8 to 43.3)	20.7 (3.7 to 53.5)	12.1 (1.7 to 32.7)	14.8 (2.7 to 37.5)	18.8 (3.8 to 45.9)	11.2 (1.8 to 29.8)	-10 (-22.4 to 13.1)	-9.3 (-23.6 to 15.1)	-7.9 (-24.9 to 23)
	Low temperature	Deaths	9.5 (2.3 to 18.2)	11.3 (2.8 to 21.9)	7.7 (1.9 to 14.6)	4.8 (1.1 to 9.1)	5.2 (1.3 to 9.8)	4.3 (1.1 to 8.4)	-50.1 (-61.2 to -37.7)	-53.6 (-64.7 to -38.5)	-43.7 (-58.9 to -25.5)
		YLLs	159.9 (39.5 to 305.4)	186 (45.5 to 357.3)	133.7 (33.2 to 255.4)	73 (17.3 to 141)	76.9 (18.9 to 144.5)	69.5 (17 to 132.3)	-54.4 (-64.8 to -42.3)	-58.6 (-68.9 to -44.8)	-48 (-62.8 to -29.5)
	Secondhand smoke	Deaths	6.3 (4.5 to 8.3)	8.5 (5.9 to 11.3)	4.2 (2.7 to 5.7)	2.4 (1.7 to 3.2)	3 (2.1 to 4.1)	1.9 (1.2 to 2.7)	-62 (-70.2 to -52.5)	-64.6 (-73.6 to -53)	-55.1 (-67.9 to -37.6)
		DALYs	143.7 (102.1 to 188)	196.1 (137 to 258.2)	93.1 (59.4 to 126.3)	55.1 (39 to 72.3)	69.9 (49.6 to 93.3)	42.1 (27.3 to 58.6)	-61.7 (-69 to -52.6)	-64.4 (-72.8 to -54)	-54.8 (-67.3 to -38.7)
		YLLs	131.2 (91.8 to 171.7)	178.2 (121.9 to 236)	85.9 (53.8 to 117.9)	46.9 (32.8 to 62.5)	57.9 (40.1 to 78.6)	37.2 (23.2 to 52.8)	-64.3 (-71.9 to -54.8)	-67.5 (-76 to -57)	-56.7 (-69.7 to -39.6)
		YLDs	12.5 (8 to 17)	18 (11.5 to 24.5)	7.3 (4.7 to 10.2)	8.2 (5.3 to 11.4)	12 (7.8 to 16.5)	4.9 (3.1 to 7.1)	-34.2 (-39.5 to -28.7)	-33.4 (-39.7 to -26.2)	-32 (-42.1 to -21.5)
	Smoking	Deaths	24.6 (19.3 to 29.8)	16.4 (11.6 to 21.8)	33 (23.2 to 40.6)	9.9 (7.5 to 12.2)	5.3 (3.9 to 7)	14.3 (9.9 to 18.2)	-59.5 (-69.5 to -47.7)	-67.8 (-78.3 to -52.6)	-56.8 (-69 to -41.9)

Location	Risk factor	Measure	1990			2019			% Change (1990 to 2019)		
			Both	Female	Male	Both	Female	Male	Both	Female	Male
		DALYs	617.4 (491.1 to 743)	424.1 (310.4 to 556.6)	808.9 (595 to 978.1)	255.7 (203.2 to 307.2)	138.1 (106.7 to 176.1)	362.1 (272 to 452.5)	-58.6 (-67.7 to -47.7)	-67.4 (-76.9 to -54.5)	-55.2 (-66.4 to -41.3)
		YLLs	533.4 (414.2 to 652.7)	362.7 (258.7 to 480.2)	702.9 (495.2 to 868.1)	202.8 (154.3 to 252)	103.4 (76.6 to 135.1)	293 (208.2 to 379.9)	-62 (-71.8 to -50)	-71.5 (-80.5 to -58.4)	-58.3 (-70.4 to -42.9)
		YLDs	84 (60.7 to 110.1)	61.4 (40.9 to 84.7)	106.1 (77 to 136.3)	52.9 (38.4 to 68.3)	34.7 (24.4 to 46.5)	69.2 (49.5 to 88.4)	-37 (-44.2 to -29.3)	-43.6 (-56 to -27.7)	-34.8 (-42.5 to -26.7)
Kuwait	All risk factors	Deaths	44.5 (38.1 to 49.9)	49.5 (40.9 to 56.9)	39.6 (33.7 to 44.2)	40.5 (32.7 to 48.4)	30.5 (24 to 36.8)	47.2 (37 to 58.2)	-8.9 (-24 to 9.3)	-38.3 (-49.3 to -25)	19.3 (-2.9 to 46.4)
		DALYs	928.7 (830.1 to 1023.3)	1049.8 (917.6 to 1189.5)	838.6 (747.6 to 923.2)	831 (700 to 979)	645.7 (536.8 to 758.9)	956.9 (782.2 to 1159.5)	-10.5 (-23.2 to 4.5)	-38.5 (-47.7 to -27.6)	14.1 (-5.4 to 37.2)
		YLLs	750 (661.6 to 827.1)	829.9 (709 to 951.1)	687.3 (607 to 762.2)	664.4 (544.1 to 802.6)	462.1 (367.6 to 559.7)	803 (633.7 to 998.5)	-11.4 (-26.8 to 7.8)	-44.3 (-55 to -31.1)	16.8 (-7.2 to 45.1)
		YLDs	178.6 (130.8 to 225)	219.9 (160.4 to 277.7)	151.3 (110.6 to 192.8)	166.6 (121.4 to 209.6)	183.5 (133.4 to 233.7)	153.9 (111.4 to 193.6)	-6.7 (-12.3 to -0.8)	-16.5 (-22.5 to -10)	1.7 (-6.5 to 10.7)
	Alcohol use	Deaths	0 (0 to 0)	0 (0 to 0)	0 (0 to 0)	0 (0 to 0)	0 (0 to 0)	0 (-0.1 to 0.1)	-	-	-
		DALYs	0 (-0.1 to 0)	0 (0 to 0)	0 (-0.1 to 0)	0 (-0.9 to 1.4)	-0.3 (-0.6 to 0)	0.2 (-1.3 to 2.6)	-	-	-
		YLLs	0 (-0.1 to 0)	0 (0 to 0)	0 (-0.1 to 0)	0.1 (-0.6 to 1.2)	-0.2 (-0.4 to 0)	0.3 (-1 to 2.3)	-	-	-
		YLDs	0 (0 to 0)	0 (0 to 0)	0 (0 to 0)	-0.1 (-0.3 to 0.1)	-0.1 (-0.2 to 0)	-0.1 (-0.4 to 0.3)	-	-	-
	Ambient particulate matter pollution	Deaths	14.9 (12.4 to 17.3)	16.5 (13.3 to 19.8)	13.4 (11.2 to 15.7)	13.7 (10.9 to 16.9)	10 (7.7 to 12.4)	16.2 (12.7 to 20.6)	-7.7 (-22.6 to 11.2)	-39.4 (-50.3 to -25.6)	20.7 (-2.3 to 49.9)
		DALYs	356.2 (304 to 413.8)	404 (339.8 to 475.3)	323.7 (275 to 374.7)	321.5 (267.4 to 391)	247.8 (202 to 300.3)	371.8 (298.6 to 464.1)	-9.7 (-22.2 to 6.4)	-38.6 (-48 to -27.5)	14.9 (-4.5 to 39.3)
		YLLs	283.2 (239.7 to 326.2)	313.7 (258.2 to 377.2)	261.6 (219.3 to 304.5)	252.1 (204.1 to 315.9)	171.1 (132.5 to 212.4)	308.2 (240.4 to 395.7)	-11 (-26.8 to 9)	-45.5 (-56.4 to -31.7)	17.8 (-6 to 48.6)
		YLDs	72.9 (52.4 to 94.7)	90.2 (65.1 to 117.3)	62.1 (44.5 to 80.9)	69.4 (50 to 89)	76.8 (54.9 to 99.9)	63.7 (45.6 to 81.5)	-4.9 (-11 to 2)	-14.9 (-21.4 to -7.3)	2.4 (-6.9 to 12.3)
	Diet high in red meat	Deaths	3.1 (1.6 to 4.2)	3.4 (1.7 to 4.8)	2.8 (1.5 to 3.9)	2.7 (1.4 to 3.9)	1.9 (0.9 to 2.8)	3.2 (1.7 to 4.7)	-12.2 (-29.4 to 9.5)	-43.4 (-56.5 to -26.5)	14.6 (-12.7 to 47.6)
		DALYs	87 (49.1 to 116.1)	99.1 (56 to 134.5)	79.5 (45.7 to 106.6)	73.1 (40.2 to 101.2)	56.7 (31 to 78.8)	84.5 (45.7 to 119.9)	-16 (-29.2 to -0.4)	-42.8 (-53.4 to -31.3)	6.3 (-14.6 to 31)
		YLLs	67.7 (38.4 to 90.8)	74.9 (42.5 to 102.3)	63 (35.5 to 84.4)	55.4 (29.9 to 78.4)	36.8 (19.2 to 53.2)	68.6 (37.3 to 98.9)	-18.1 (-33.8 to 1.3)	-50.8 (-63 to -37.3)	9 (-16.9 to 39.4)
		YLDs	19.3 (10 to 28.1)	24.2 (12.3 to 35.5)	16.5 (8.6 to 24)	17.6 (8.9 to 26)	19.8 (10.2 to 29.3)	15.9 (7.7 to 23.4)	-8.7 (-17.6 to 0.7)	-17.9 (-27.7 to -5.8)	-3.7 (-17.3 to 10.3)
	Diet high in sodium	Deaths	1.5 (0.1 to 5.5)	1.1 (0.1 to 4.8)	1.8 (0.1 to 5.9)	1.5 (0.1 to 5.4)	0.7 (0.1 to 2.8)	2.1 (0.1 to 7.1)	1.9 (-42.4 to 60.2)	-41.9 (-65.9 to 8.4)	18.1 (-32.4 to 88.1)
		DALYs	37.6 (2.4 to 127.8)	27.4 (2.3 to 115.1)	44 (2.1 to 137.9)	36.1 (2 to 120.8)	16.2 (1.4 to 67.2)	49.8 (2.4 to 157.9)	-3.9 (-41.8 to 45.7)	-41 (-60.4 to -3.3)	13.2 (-35.8 to 75.8)
		YLLs	30.1 (1.9 to 103.3)	21.5 (1.9 to 89.8)	35.7 (1.7 to 112.1)	29.2 (1.6 to 97.8)	11.3 (1 to 48.3)	41.5 (1.9 to 132.5)	-3.2 (-42.1 to 51.9)	-47.2 (-66.2 to -9)	16.1 (-34.6 to 83.5)
		YLDs	7.4 (0.4 to 25.6)	5.9 (0.4 to 24.9)	8.3 (0.4 to 26.4)	6.9 (0.4 to 23.5)	4.8 (0.4 to 19.6)	8.3 (0.4 to 26.3)	-7.1 (-39.9 to 34.7)	-18.6 (-42 to 26.9)	0.4 (-40.8 to 59.1)
	Diet low in fiber	Deaths	1.7 (0.4 to 3.1)	1.8 (0.4 to 3.5)	1.5 (0.3 to 2.8)	1 (0.2 to 2.1)	0.7 (0.2 to 1.6)	1.2 (0.3 to 2.4)	-39.1 (-54.8 to -21)	-59.4 (-72.4 to -44.7)	-20.5 (-45.3 to 9.9)
		DALYs	40.6 (8.3 to 74.9)	46.1 (9 to 87.5)	36.8 (7.7 to 67.5)	25 (5.3 to 49.1)	19.7 (3.9 to 39.8)	28.7 (6.2 to 56.9)	-38.4 (-51.7 to -24.1)	-57.2 (-68 to -46.8)	-22.2 (-42.3 to -0.7)
		YLLs	31.8 (6.7 to 59)	35.1 (7 to 67.6)	29.3 (6.2 to 54.3)	19 (4.1 to 37.9)	13 (2.6 to 26.4)	23.3 (5 to 47)	-40.2 (-54.6 to -23.3)	-63 (-74.3 to -51.4)	-20.7 (-42.7 to 4.9)
		YLDs	8.8 (1.7 to 17.1)	11 (2 to 21.3)	7.5 (1.5 to 14.6)	6 (1.2 to 12.2)	6.7 (1.2 to 14)	5.4 (1.1 to 11)	-32.1 (-44.1 to -22)	-38.7 (-52.2 to -26.5)	-28 (-43.5 to -14.6)
	Diet low in fruits	Deaths	1.8 (0.7 to 3.1)	2 (0.8 to 3.6)	1.6 (0.7 to 2.7)	1.7 (0.7 to 3)	1.2 (0.5 to 2.2)	2 (0.8 to 3.6)	-4.7 (-23.9 to 21.2)	-38.2 (-52.5 to -19.2)	26 (-5.8 to 66.5)
		DALYs	47.2 (19.8 to 79.5)	54.6 (22.4 to 93.6)	42.5 (18.3 to 72.2)	45.3 (19 to 76.4)	35.6 (14.7 to 59.7)	52.2 (22 to 87.9)	-4 (-20.2 to 17.1)	-34.8 (-47.1 to -19.6)	22.8 (-2.8 to 54.9)
		YLLs	37.4 (16.1 to 62.7)	42 (17.5 to 71.7)	34.3 (15.1 to 58.1)	35 (15.5 to 59.5)	23.7 (10.3 to 40)	43 (18.8 to 74.3)	-6.4 (-24.7 to 18.4)	-43.5 (-56.9 to -25.9)	25.4 (-4.1 to 62.1)
		YLDs	9.8 (3.2 to 18.3)	12.5 (4.2 to 23.2)	8.2 (2.7 to 15)	10.4 (3.7 to 19)	11.8 (4.2 to 22.1)	9.2 (3.2 to 17.2)	5.1 (-5.7 to 23.7)	-5.8 (-16.9 to 14.1)	11.7 (-3.9 to 35.5)
	Diet low in vegetables	Deaths	0.4 (0.1 to 0.9)	0.4 (0.1 to 0.9)	0.4 (0.1 to 0.9)	0.2 (0.1 to 0.4)	0.1 (0.1 to 0.3)	0.3 (0.1 to 0.6)	-51.3 (-69.3 to -16.4)	-67.9 (-82.2 to -42.1)	-38.4 (-64.7 to 11.5)
		DALYs	8.3 (2.3 to 18.1)	8.4 (2.5 to 18.3)	8.1 (2.1 to 18.1)	4 (1.9 to 8.5)	2.8 (1.4 to 5.8)	4.9 (2.1 to 11)	-51.5 (-67.1 to -16)	-66.9 (-79.7 to -40.8)	-39.9 (-61.2 to 10.6)
		YLLs	6.7 (1.9 to 14.8)	6.7 (2 to 14.5)	6.7 (1.7 to 15)	3.3 (1.5 to 6.9)	2 (1 to 4.2)	4.1 (1.8 to 9.1)	-51.6 (-67.5 to -15.8)	-69.9 (-82.4 to -44.7)	-38.3 (-61 to 13)
		YLDs	1.6 (0.4 to 3.7)	1.8 (0.5 to 4.2)	1.5 (0.3 to 3.5)	0.8 (0.3 to 1.7)	0.8 (0.4 to 1.7)	0.8 (0.3 to 1.8)	-51 (-65.2 to -11.4)	-55.8 (-71.8 to -20.1)	-46.8 (-65 to -2.1)
	Diet low in whole grains	Deaths	1.7 (0.4 to 2.6)	2 (0.5 to 3)	1.5 (0.4 to 2.2)	1.5 (0.4 to 2.3)	1.1 (0.3 to 1.8)	1.7 (0.4 to 2.7)	-14.2 (-28.9 to 2.9)	-42.9 (-53.2 to -30.4)	15.1 (-8.3 to 42.9)
		DALYs	40.5 (9.7 to 60.3)	47.5 (11.4 to 71.8)	35.7 (8.5 to 53.2)	34.2 (8.4 to 52.4)	28 (6.7 to 42.7)	38.4 (9.6 to 60.2)	-15.5 (-26.9 to -2.2)	-41.1 (-49.4 to -31.4)	7.8 (-10.5 to 30.3)
		YLLs	30 (7.3 to 44.6)	34.4 (8.2 to 51.9)	26.8 (6.5 to 39.5)	24.5 (6.2 to 38.3)	17.2 (4.2 to 27.1)	29.6 (7.4 to 47.2)	-18.3 (-33.4 to 0)	-49.9 (-59.6 to -37.8)	10.5 (-14.3 to 40.6)
		YLDs	10.5 (2.5 to 16.9)	13.1 (3.1 to 21.2)	8.9 (2.1 to 14.5)	9.7 (2.3 to 15.8)	10.7 (2.5 to 17.8)	8.9 (2.2 to 14.4)	-7.5 (-15 to -0.1)	-17.9 (-26.5 to -9.4)	-0.2 (-11.1 to 11.3)
	High body-mass index	Deaths	11 (7.1 to 15.4)	13.7 (9.2 to 19)	9.1 (5.5 to 12.9)	11.9 (7.3 to 16.8)	8.9 (5.5 to 12.7)	13.9 (8.5 to 20)	8.1 (-13.7 to 35.6)	-35.1 (-50.1 to -16.7)	52.9 (16.7 to 99.5)
		DALYs	361.4 (255.1 to 468.1)	454.3 (329.3 to 578.6)	306.2 (207.2 to 404.7)	368.9 (255.9 to 482.7)	301.7 (216.8 to 388)	416.6 (281.6 to 568.4)	2.1 (-14.4 to 21.5)	-33.6 (-44.9 to -19.7)	36 (8.7 to 68.7)
		YLLs	282.5 (196.4 to 366.6)	345.9 (249.8 to 446.4)	244.5 (163.7 to 325.1)	281.2 (188.3 to 376.9)	198.2 (134.4 to 262.2)	340.8 (219.1 to 469.3)	-0.5 (-20.3 to 23.6)	-42.7 (-56.1 to -26.7)	39.4 (7.6 to 78.8)
		YLDs	78.9 (49.8 to 110.3)	108.4 (71.4 to 148.1)	61.7 (36.9 to 89.7)	87.7 (58.2 to 119.5)	103.5 (70.2 to 138.8)	75.7 (48.8 to 105.8)	11.1 (1.2 to 23.5)	-4.5 (-13.2 to 5.7)	22.7 (7 to 43.9)
	High fasting plasma glucose	Deaths	15.5 (8.6 to 28.8)	17.2 (9 to 33.2)	13.9 (7.8 to 25.1)	14.1 (8.3 to 25.4)	10.7 (5.8 to 21)	16.3 (9.5 to 29.3)	-9.6 (-29 to 17.5)	-37.6 (-53.2 to -17.5)	16.7 (-15.7 to 61.5)
		DALYs	299.2 (184.1 to 488.8)	334.6 (191.5 to 556.9)	271.1 (168.9 to 432.3)	281.1 (178.1 to 446.7)	217.1 (131.9 to 359.4)	324.2 (204.3 to 507.5)	-6 (-26.1 to 22.2)	-35.1 (-50.6 to -15.9)	19.6 (-13.3 to 66.4)
		YLLs	244.1 (148.3 to 407.9)	268.5 (152.4 to 462.7)	223.8 (138.9 to 360.1)	227.1 (142.7 to 366.7)	158.7 (94.8 to 270.2)	273.1 (171.1 to 428.9)	-7 (-29.1 to 24.7)	-40.9 (-56.7 to -20.6)	22 (-13.7 to 73.2)
		YLDs	55.1 (30.7 to 90.5)	66.1 (35.7 to 111.1)	47.3 (25.7 to 78)	54 (31.7 to 85.7)	58.4 (32.3 to 93.7)	51 (30.4 to 82.1)	-1.9 (-18.8 to 21.8)	-11.6 (-31.3 to 12.4)	7.9 (-16.3 to 44.4)
	High LDL cholesterol	Deaths	8.1 (3 to 16.9)	9.5 (3.7 to 19.5)	6.6 (2.6 to 13.5)	7.3 (2.5 to 15.7)	6 (1.9 to 13.7)	8.2 (2.8 to 17.6)	-9.6 (-26.7 to 7.6)	-36.8 (-53 to -22.6)	23.3 (-4.2 to 53.2)
		DALYs	184.3 (108.1 to 303.3)	223.9 (132 to 376)	155.8 (92.5 to 256.9)	164 (90.3 to 282.1)	142.4 (77.8 to 245.3)	178.7 (97.4 to 309)	-11 (-24.9 to 3.1)	-36.4 (-47 to -25.2)	14.7 (-7.6 to 39.6)

Location	Risk factor	Measure	1990			2019			% Change (1990 to 2019)		
			Both	Female	Male	Both	Female	Male	Both	Female	Male
		YLLs	136.6 (76.8 to 234.6)	162.2 (91 to 276.4)	116.8 (66.2 to 199.3)	116.9 (57.3 to 213.9)	88.3 (40.2 to 171)	136.9 (67.4 to 252.1)	-14.4 (-32.9 to 4.4)	-45.5 (-60.1 to -31.5)	17.2 (-12 to 50.9)
		YLDs	47.7 (28.6 to 76.9)	61.7 (37.2 to 98.5)	38.9 (23.4 to 62.6)	47.1 (29.1 to 76)	54.1 (33.3 to 85.3)	41.8 (24.8 to 67.7)	-1.2 (-8.8 to 7.9)	-12.4 (-21.1 to -2.9)	7.3 (-5 to 20.9)
	High systolic blood pressure	Deaths	25.3 (19.6 to 31.7)	29.1 (21.5 to 37.3)	21.6 (16.9 to 27)	23 (16.8 to 29.9)	17.9 (12.6 to 24.4)	26.4 (18.7 to 35.3)	-9.3 (-27.3 to 11.9)	-38.4 (-53.3 to -20.6)	22.1 (-5.8 to 57.1)
		DALYs	533.9 (434.2 to 633.3)	615.7 (480.6 to 756.4)	471.1 (381.2 to 559.5)	478.8 (375.2 to 590.9)	373.9 (284.9 to 472.3)	551.9 (423 to 698)	-10.3 (-26.2 to 8.7)	-39.3 (-51.5 to -23.8)	17.2 (-6.4 to 47.1)
		YLLs	432 (352.3 to 512.8)	489.1 (375.5 to 608.2)	385.9 (308.9 to 462.5)	384.6 (295 to 488.4)	271.1 (197.7 to 354.5)	463.4 (342.7 to 601)	-11 (-29 to 11)	-44.6 (-58.1 to -27.4)	20.1 (-7.2 to 56.8)
		YLDs	101.9 (71.2 to 134.4)	126.6 (86 to 172.9)	85.2 (58.4 to 114.1)	94.1 (66.3 to 123.3)	102.7 (71.9 to 138.9)	88.5 (62.4 to 117.1)	-7.6 (-17.8 to 4.2)	-18.9 (-33.1 to -2.6)	3.8 (-10.6 to 19.4)
	High temperature	Deaths	2.3 (0.3 to 4.2)	2.6 (0.4 to 4.7)	2.1 (0.3 to 3.7)	2.3 (0.6 to 3.9)	1.7 (0.4 to 3)	2.7 (0.6 to 4.7)	-1.2 (-31 to 28.1)	-32.8 (-52.4 to -12.5)	28.7 (-10.4 to 69.4)
		YLLs	38.7 (5.6 to 69.8)	42.6 (6.1 to 77.8)	35.6 (5.1 to 64.3)	36.8 (8.6 to 62.9)	25.7 (6.7 to 44.5)	44.4 (10.4 to 78.4)	-4.8 (-31.4 to 22.4)	-39.5 (-57.6 to -19.4)	24.7 (-13.8 to 65.6)
	Household air pollution from solid fuels	Deaths	0.2 (0.1 to 0.5)	0.3 (0.1 to 0.7)	0.2 (0.1 to 0.4)	0 (0 to 0)	0 (0 to 0)	0 (0 to 0)	-97.8 (-99.2 to -94.8)	-98.4 (-99.5 to -96.2)	-96.9 (-98.9 to -92.6)
		DALYs	5.7 (1.8 to 12.7)	8 (2.7 to 17.5)	4.2 (1.3 to 9.1)	0.1 (0 to 0.4)	0.1 (0 to 0.4)	0.1 (0 to 0.4)	-97.8 (-99.2 to -94.9)	-98.4 (-99.4 to -96.2)	-97 (-98.9 to -93)
		YLLs	4.5 (1.5 to 10.2)	6.2 (2.1 to 14)	3.4 (1 to 7.5)	0.1 (0 to 0.3)	0.1 (0 to 0.2)	0.1 (0 to 0.3)	-97.9 (-99.2 to -94.9)	-98.6 (-99.5 to -96.6)	-97 (-98.9 to -92.8)
		YLDs	1.2 (0.4 to 2.7)	1.8 (0.6 to 3.9)	0.8 (0.2 to 1.9)	0 (0 to 0.1)	0 (0 to 0.1)	0 (0 to 0.1)	-97.6 (-99.1 to -94.3)	-97.8 (-99.2 to -94.9)	-97.4 (-99.1 to -94)
	Kidney dysfunction	Deaths	4.3 (2.7 to 5.9)	5.3 (3.3 to 7.4)	3.4 (2.3 to 4.5)	4.9 (2.9 to 6.8)	3.7 (2.1 to 5.5)	5.6 (3.5 to 7.9)	13.9 (-8.4 to 40.4)	-30.3 (-46.5 to -13.1)	64.2 (26.3 to 106.7)
		DALYs	94.6 (72 to 117.6)	123.4 (92.6 to 155.3)	75.4 (58.3 to 92.8)	104.4 (74.3 to 135)	86.3 (61.6 to 112.7)	116.1 (83.4 to 154.2)	10.3 (-7.1 to 31.3)	-30.1 (-42.1 to -15.8)	54 (23.1 to 88.1)
		YLLs	75.3 (56.4 to 95.4)	96.2 (70.6 to 125.4)	61.1 (47 to 75)	82.9 (57.6 to 110.3)	61.1 (40.3 to 83.1)	97.3 (67.5 to 132)	10.1 (-11.3 to 36.8)	-36.5 (-50.4 to -19.8)	59.4 (22.1 to 101.6)
		YLDs	19.3 (13 to 26)	27.2 (18.3 to 36.8)	14.3 (9.6 to 19.2)	21.4 (14.3 to 29.4)	25.2 (16.9 to 34.1)	18.8 (12.7 to 25.6)	11.1 (1.7 to 21.6)	-7.5 (-16.8 to 2)	31.3 (15 to 48.6)
	Lead exposure	Deaths	1.6 (0.7 to 2.5)	1.4 (0.5 to 2.5)	1.7 (0.9 to 2.6)	1.4 (0.6 to 2.4)	0.8 (0.2 to 1.5)	1.8 (0.8 to 3)	-10.9 (-27.3 to 8.9)	-45.2 (-58.6 to -31.6)	7.6 (-14.9 to 34.1)
		DALYs	35.7 (16.5 to 57.3)	31.3 (9.7 to 55.3)	38.3 (19.5 to 58.4)	25.8 (10.3 to 44.2)	14.2 (3.7 to 28)	33.5 (14.5 to 55.8)	-27.7 (-43.4 to -12.5)	-54.6 (-68.2 to -44.8)	-12.5 (-33.3 to 9.2)
		YLLs	28.8 (13.3 to 45.8)	24.8 (7.8 to 43.9)	31.3 (16 to 47.7)	21.4 (8.7 to 36.5)	10.7 (2.8 to 21.2)	28.6 (12.1 to 47.8)	-25.7 (-42.1 to -8.2)	-56.9 (-69.9 to -45.6)	-8.8 (-32.2 to 16.3)
		YLDs	6.8 (2.7 to 11.8)	6.5 (1.8 to 12.4)	7 (3 to 11.5)	4.4 (1.4 to 8.3)	3.5 (0.7 to 7.5)	4.9 (1.8 to 8.9)	-36.1 (-52.8 to -25.5)	-45.8 (-66.5 to -34.8)	-29.5 (-47.4 to -17.1)
	Low physical activity	Deaths	4.3 (1.1 to 8.2)	5.2 (1.4 to 9.6)	3.3 (0.7 to 6.6)	3.9 (1 to 7.5)	3.4 (0.9 to 6.2)	4.2 (1 to 8.3)	-9.3 (-25.2 to 10.1)	-33.6 (-46.3 to -15.4)	25.6 (-0.5 to 61)
		DALYs	69.7 (15.8 to 146)	87.1 (22.3 to 177.4)	55.3 (10.4 to 119.7)	64 (14.5 to 129.7)	58.7 (14.9 to 112.8)	67.5 (14.1 to 142.6)	-8.2 (-22.2 to 9.2)	-32.6 (-43 to -15.4)	22 (-1.2 to 57.3)
		YLLs	55.4 (13.1 to 111.8)	68.1 (17.4 to 136.1)	44.2 (8.5 to 94.2)	49.8 (11.8 to 100.1)	41.7 (11.3 to 78.1)	55.2 (11.9 to 114.2)	-10.1 (-26.7 to 11.4)	-38.8 (-51 to -19.2)	24.8 (-2.3 to 64)
		YLDs	14.4 (3 to 31.9)	19.1 (4.2 to 42.6)	11 (1.7 to 25.9)	14.2 (2.9 to 32.1)	17 (3.6 to 37)	12.2 (2.1 to 28.6)	-0.8 (-10.9 to 11.7)	-10.7 (-21 to 5.1)	11 (-2.3 to 34.4)
	Low temperature	Deaths	2 (-1.6 to 5.4)	2.3 (-1.8 to 6.1)	1.8 (-1.4 to 4.8)	2 (-1.6 to 5.4)	1.5 (-1.2 to 4)	2.3 (-1.9 to 6.3)	-2 (-47 to 62.2)	-33.4 (-63 to 9.3)	28 (-32.2 to 109.5)
		YLLs	34 (-26.5 to 89.2)	37.5 (-29.4 to 100.7)	31.2 (-24.9 to 82.6)	32.1 (-26 to 87.7)	22.4 (-18.1 to 60)	38.8 (-32.2 to 106.7)	-5.5 (-49 to 52.8)	-40.1 (-66.5 to -2.9)	24.1 (-34.6 to 96.8)
	Secondhand smoke	Deaths	1.8 (1.3 to 2.3)	2.4 (1.8 to 3.2)	1.3 (1 to 1.7)	1.5 (1 to 2)	1.4 (1 to 1.9)	1.5 (1.1 to 2.1)	-17.3 (-33.5 to 1)	-43.3 (-55.9 to -27.2)	17.6 (-10.1 to 50.6)
		DALYs	42.3 (31.8 to 54.3)	62.6 (46.7 to 80.4)	30 (22.1 to 38.9)	34.8 (25.3 to 45.4)	35.4 (25.6 to 46.1)	34 (23.3 to 46.1)	-17.8 (-31.8 to -1.1)	-43.4 (-54.6 to -30)	13.4 (-12.6 to 45.6)
		YLLs	36.5 (27.4 to 47)	53.1 (39.1 to 69.2)	26.4 (19.3 to 34.6)	29.2 (20.8 to 38.8)	27.2 (19.1 to 36.4)	30.4 (20.6 to 42.1)	-20.1 (-36.2 to -1.2)	-48.7 (-60.9 to -33.9)	15.1 (-13.2 to 49.6)
		YLDs	5.8 (3.8 to 7.9)	9.6 (6.3 to 13.1)	3.6 (2.3 to 5.1)	5.6 (3.7 to 7.7)	8.2 (5.4 to 11.3)	3.6 (2.3 to 5.1)	-2.9 (-12.1 to 7.4)	-14.1 (-23.2 to -3.8)	0.8 (-15.4 to 20.6)
	Smoking	Deaths	6.1 (5.2 to 7.1)	2.8 (1.9 to 3.9)	8.5 (7.3 to 9.8)	5.4 (4.3 to 6.8)	1.1 (0.7 to 1.5)	8.3 (6.5 to 10.5)	-11.2 (-30.5 to 12.7)	-61.5 (-74.6 to -38.5)	-2.4 (-24.4 to 24.2)
		DALYs	178.2 (154 to 204.4)	79.2 (55.9 to 108)	239 (208.1 to 272)	144.9 (117.3 to 176.4)	32.8 (23.8 to 44.5)	223.1 (177.8 to 274.3)	-18.7 (-34.1 to 0.3)	-58.5 (-71.3 to -37.2)	-6.7 (-24.1 to 14.6)
		YLLs	141.4 (121 to 161.7)	61 (42.2 to 84.7)	191.3 (164.3 to 218.8)	117 (91.1 to 147.3)	22.1 (15.6 to 30.7)	182.9 (139.4 to 231.9)	-17.2 (-36.3 to 6.4)	-63.8 (-75.8 to -42.6)	-4.4 (-25.9 to 22.6)
		YLDs	36.8 (26.1 to 47.7)	18.1 (11.4 to 26.4)	47.7 (34.4 to 62)	27.9 (19.9 to 35.8)	10.7 (7 to 15.2)	40.2 (28.5 to 51.4)	-24.2 (-33.8 to -13.7)	-40.8 (-58.4 to -16.2)	-15.7 (-26.2 to -4.4)
Lebanon	All risk factors	Deaths	46.7 (38.3 to 56.3)	50.2 (39.7 to 62.5)	42.2 (34.1 to 51.9)	29.8 (20.8 to 38.6)	29 (19.3 to 38.4)	30.6 (20.6 to 41.5)	-36.2 (-54 to -15)	-42.2 (-58.6 to -19.2)	-27.6 (-52.4 to 1.2)
		DALYs	942 (802 to 1109.7)	1036.6 (865.6 to 1242.9)	836.4 (695.2 to 1006.2)	660.8 (511.1 to 818.3)	677.3 (523.4 to 845.6)	639.8 (470.7 to 829.1)	-29.8 (-45.1 to -11.7)	-34.7 (-49.4 to -16.3)	-23.5 (-44.1 to 0.8)
		YLLs	758 (634.1 to 923.9)	803.8 (650.4 to 1005.9)	703.5 (573.4 to 875.9)	463.6 (324.1 to 607.7)	439.3 (294.7 to 589.9)	491.4 (331.8 to 677.5)	-38.8 (-57 to -16.7)	-45.3 (-62.1 to -22.6)	-30.1 (-55.4 to -1.7)
		YLDs	184 (133.3 to 233.5)	232.8 (169.2 to 297.8)	132.9 (95.3 to 167.8)	197.2 (145.7 to 249)	238 (173.4 to 302.2)	148.4 (108.9 to 188.7)	7.2 (1.3 to 13.6)	2.2 (-5.4 to 10.2)	11.7 (3.3 to 20.5)
	Alcohol use	Deaths	0.7 (0.4 to 1.1)	0.2 (-0.1 to 0.4)	1.4 (0.9 to 2)	0.2 (0 to 0.3)	-0.1 (-0.1 to 0)	0.4 (0.2 to 0.7)	-79 (-95 to -59.8)	-134.6 (-567.4 to 714.2)	-70.1 (-86.4 to -49.1)
		DALYs	19.7 (12.4 to 28.3)	4.1 (-0.5 to 10.3)	35.9 (23.5 to 52.2)	4.9 (1.5 to 9.5)	-1 (-3.1 to 1)	12.1 (5.4 to 20.9)	-75 (-91 to -56.5)	-125.2 (-628.7 to 289.9)	-66.4 (-82.2 to -47.1)
		YLLs	17.1 (10.9 to 24.7)	3.9 (0 to 9)	31 (19.9 to 45.9)	4 (1.4 to 7.7)	-0.5 (-1.9 to 0.9)	9.3 (4 to 17.2)	-76.8 (-91.1 to -58.5)	-113.2 (-347.3 to -51.8)	-69.8 (-84.8 to -49.6)
		YLDs	2.5 (1.2 to 4)	0.3 (-0.8 to 1.5)	4.9 (2.6 to 7.5)	0.9 (0.1 to 2)	-0.5 (-1.3 to 0.2)	2.7 (1.1 to 4.6)	-62.5 (-94.8 to -35.7)	-301.2 (-2389.7 to 1606.9)	-44.3 (-69.4 to -21.5)
	Ambient particulate matter pollution	Deaths	8.2 (5.8 to 11)	8.5 (5.7 to 11.7)	7.8 (5.4 to 10.5)	6.3 (4 to 8.5)	6.1 (4 to 8.6)	6.5 (3.8 to 9.4)	-23.7 (-46.8 to 7.8)	-29 (-52.4 to 8.2)	-16.8 (-44.5 to 20.3)
		DALYs	194.1 (137.7 to 257.2)	208.2 (145.9 to 281.2)	178.5 (126.1 to 241.7)	165.6 (115.7 to 215.5)	170.9 (117.1 to 227.7)	159.1 (103.9 to 219.9)	-14.7 (-35.1 to 15.8)	-17.9 (-39.7 to 16.6)	-10.9 (-36.3 to 22.6)
		YLLs	153.1 (107.1 to 205.8)	157.6 (107.8 to 219.6)	147.6 (101.9 to 202.1)	112.1 (70 to 155.3)	106 (67.7 to 152)	119 (71.3 to 175.2)	-26.8 (-50.6 to 4.7)	-32.7 (-56.4 to 3.8)	-19.4 (-48.8 to 18.5)
		YLDs	41 (26 to 57.4)	50.7 (31.5 to 72.7)	30.9 (19.8 to 43.1)	53.6 (36.6 to 71.6)	64.9 (43.9 to 87)	40.1 (27.1 to 53.8)	30.7 (13.2 to 62.1)	28.1 (8.3 to 64.1)	29.6 (12.2 to 59.9)
	Diet high in red meat	Deaths	2.1 (0.8 to 3.2)	2.2 (0.9 to 3.5)	1.9 (0.8 to 3)	1.4 (0.5 to 2.2)	1.3 (0.5 to 2.2)	1.4 (0.5 to 2.4)	-32.9 (-58 to -3)	-39.5 (-63.6 to -6.8)	-23.8 (-56.1 to 18.5)
		DALYs	54.8 (23.6 to 82.5)	61 (25.2 to 92.8)	48.1 (20.1 to 74.1)	43.3 (17.1 to 65.9)	45 (18.3 to 69.2)	41.3 (16.2 to 65.3)	-20.9 (-45.3 to 13.4)	-26.2 (-49.7 to 7.6)	-14 (-43.4 to 26)
		YLLs	42.4 (17.8 to 65.1)	45.2 (18.5 to 70)	39.1 (16.4 to 61.6)	28.2 (10.6 to 45.6)	26.7 (9.4 to 44.9)	30 (11.4 to 50.2)	-33.4 (-59.6 to 2.2)	-41 (-65.3 to -3)	-23.3 (-54.6 to 23.7)

Location	Risk factor	Measure	1990			2019			% Change (1990 to 2019)		
			Both	Female	Male	Both	Female	Male	Both	Female	Male
	Diet high in sodium	YLDs	12.4 (4.4 to 19.7)	15.7 (5.6 to 25)	9 (3.2 to 14.1)	15.1 (5.8 to 23.5)	18.3 (6.8 to 28.3)	11.3 (4.4 to 17.8)	21.5 (7.4 to 47.4)	16.4 (0.5 to 42.9)	26.3 (9.2 to 58)
		Deaths	0.8 (0.2 to 3.4)	0.6 (0.2 to 2.6)	1 (0.1 to 4.3)	0.5 (0.1 to 2.1)	0.3 (0.1 to 1.4)	0.7 (0.1 to 2.9)	-39.2 (-69.4 to 8.5)	-48.2 (-76.2 to 0.2)	-30.5 (-69 to 65.4)
		DALYs	19.6 (2.9 to 79.8)	15.5 (3.2 to 64.7)	23.8 (2 to 96.5)	13.2 (1.9 to 54.9)	9.2 (2 to 38.8)	17.9 (1.4 to 71.6)	-32.5 (-60.3 to 16.9)	-40.5 (-68.9 to 25.4)	-24.6 (-59.5 to 61.9)
		YLLs	15.7 (2.3 to 65)	11.8 (2.5 to 49.6)	19.8 (1.6 to 80.3)	9.3 (1.3 to 38.7)	5.8 (1.2 to 25.2)	13.4 (1 to 53.3)	-41 (-68 to 4.4)	-51 (-76.9 to 4.8)	-32.2 (-65.5 to 58.6)
	Diet low in fiber	YLDs	3.9 (0.5 to 15.8)	3.7 (0.7 to 15.8)	4.1 (0.3 to 16.1)	3.9 (0.5 to 15.8)	3.4 (0.6 to 14.3)	4.5 (0.3 to 17.6)	2.1 (-37.2 to 81.9)	-6.8 (-51.6 to 114.6)	12.1 (-30.3 to 130)
		Deaths	0.4 (0.1 to 0.9)	0.4 (0.1 to 1)	0.4 (0.1 to 0.9)	0.3 (0.1 to 0.7)	0.3 (0.1 to 0.7)	0.3 (0.1 to 0.8)	-25.2 (-55.6 to 15.9)	-31.5 (-65.2 to 20.8)	-16.2 (-56.2 to 47.8)
		DALYs	7.8 (2.7 to 16.6)	8.2 (2.9 to 17.4)	7.3 (2.4 to 16.3)	7.7 (2.1 to 17.2)	7.7 (2 to 17.9)	7.6 (2 to 18.5)	-1.3 (-33.5 to 36.2)	-5.6 (-42.4 to 39.4)	4.6 (-34.8 to 59.2)
		YLLs	6.2 (2.2 to 13.6)	6.4 (2.2 to 14)	6.1 (2 to 13.2)	5.2 (1.4 to 11.8)	4.8 (1.2 to 10.9)	5.7 (1.4 to 14.1)	-17 (-49.6 to 21.9)	-25.1 (-59.6 to 19.2)	-6.6 (-48.8 to 49.5)
	Diet low in fruits	YLDs	1.5 (0.5 to 3.5)	1.8 (0.6 to 4.2)	1.2 (0.4 to 2.8)	2.5 (0.6 to 5.9)	3 (0.7 to 7.4)	2 (0.5 to 4.6)	62.5 (15.1 to 102)	62.7 (6.7 to 122.6)	60 (13.1 to 111.9)
		Deaths	0.4 (0.2 to 0.7)	0.5 (0.2 to 1)	0.3 (0.2 to 0.6)	0.4 (0.2 to 0.8)	0.5 (0.2 to 0.9)	0.4 (0.2 to 0.8)	5.3 (-44.4 to 94.4)	-6.9 (-54.2 to 101.1)	26.9 (-47 to 208.8)
		DALYs	9.2 (5.3 to 14.4)	11.4 (6.4 to 18.3)	6.6 (3.8 to 11.1)	14.4 (6 to 25.8)	15.8 (6.4 to 29)	12.7 (5.2 to 23.6)	57 (-9.1 to 153.1)	38.7 (-21.2 to 136.5)	91.4 (-0.7 to 240.6)
		YLLs	7.3 (4.2 to 11.5)	8.8 (4.8 to 14.3)	5.5 (3.1 to 9.4)	9.4 (3.8 to 17.1)	9.4 (3.5 to 18.1)	9.2 (3.7 to 18.2)	28.9 (-30.1 to 122.7)	7.4 (-47.1 to 100.6)	67.2 (-19 to 217.5)
	Diet low in vegetables	YLDs	1.9 (0.9 to 3.3)	2.6 (1.2 to 4.6)	1.1 (0.6 to 2)	5 (1.7 to 9.2)	6.4 (2.2 to 12.2)	3.5 (1.2 to 6.7)	164 (73 to 273.4)	143.8 (55.5 to 274.4)	209.9 (86.3 to 383.6)
		Deaths	0.1 (0.1 to 0.2)	0.2 (0.1 to 0.3)	0.1 (0.1 to 0.2)	0.1 (0.1 to 0.2)	0.1 (0.1 to 0.2)	0.1 (0.1 to 0.2)	-36.3 (-62.4 to 0.8)	-42.6 (-71.3 to 4.8)	-27.4 (-66.8 to 30.3)
		DALYs	2.5 (2 to 3.4)	2.8 (2.2 to 3.9)	2.2 (1.7 to 3.3)	1.8 (1.3 to 2.5)	1.8 (1.2 to 2.6)	1.7 (1.2 to 2.8)	-30 (-51.8 to -2.5)	-35.9 (-57.1 to -7.6)	-21.9 (-52 to 18.1)
		YLLs	2.1 (1.6 to 2.9)	2.2 (1.6 to 3.2)	1.9 (1.4 to 2.9)	1.3 (0.8 to 1.9)	1.2 (0.8 to 1.9)	1.4 (0.8 to 2.3)	-38.1 (-60.2 to -8.8)	-45.4 (-68.8 to -13.3)	-28.2 (-61.5 to 12)
	Diet low in whole grains	YLDs	0.4 (0.3 to 0.6)	0.6 (0.4 to 0.7)	0.3 (0.2 to 0.4)	0.5 (0.3 to 0.6)	0.6 (0.4 to 0.8)	0.4 (0.3 to 0.5)	7.4 (-5 to 31.1)	0.7 (-13.5 to 26.7)	15.6 (-3.2 to 55.5)
		Deaths	3.2 (1.9 to 4.2)	3.4 (2.1 to 4.7)	2.9 (1.7 to 4)	2.4 (1.3 to 3.5)	2.4 (1.2 to 3.5)	2.5 (1.2 to 3.9)	-23.8 (-47.2 to 0.4)	-31.2 (-52.1 to -4.3)	-12.9 (-42.9 to 20.6)
		DALYs	64.2 (36.7 to 86.5)	71.3 (40.9 to 95.7)	56.3 (30.9 to 78.2)	56.5 (30.5 to 79.8)	58.4 (30.3 to 83.4)	54.2 (28.2 to 80.5)	-12 (-31.9 to 7.9)	-18.2 (-36.4 to 3.1)	-3.6 (-32.4 to 25.1)
		YLLs	46.4 (26.8 to 62.8)	48.8 (28.2 to 67.6)	43.4 (24.6 to 61.4)	37.2 (17.5 to 55.1)	35 (16 to 53.1)	39.8 (19.3 to 62)	-19.9 (-46.2 to 7)	-28.3 (-52 to 1.9)	-8.4 (-44.6 to 30.1)
	High body-mass index	YLDs	17.8 (9.2 to 26)	22.5 (11.5 to 32.8)	12.9 (6.6 to 19)	19.3 (9.9 to 28.1)	23.4 (11.8 to 34.2)	14.5 (7.5 to 21.1)	8.4 (2 to 15.3)	3.7 (-4.2 to 12.7)	12.4 (2.5 to 23.8)
		Deaths	9.1 (5.3 to 13.8)	10.9 (6.4 to 16.4)	7.2 (3.8 to 11.5)	6.7 (3.6 to 11.1)	6.8 (3.5 to 11.3)	6.5 (3.4 to 11)	-26.8 (-49.6 to 3.7)	-37.8 (-59 to -7.9)	-9 (-45.1 to 39.3)
		DALYs	302.2 (192 to 434.9)	369.8 (236.1 to 522)	231.4 (132.9 to 352)	246.6 (159.2 to 364.3)	268.1 (173.5 to 385.9)	220.8 (133.2 to 339.5)	-18.4 (-38 to 7)	-27.5 (-44.9 to -4.2)	-4.6 (-32.9 to 33)
		YLLs	232.8 (142.3 to 332.4)	274.5 (170 to 392.1)	188.4 (105.6 to 293.4)	158.7 (88.9 to 248.2)	157.4 (84 to 254.5)	159.5 (87.7 to 263.1)	-31.9 (-53.1 to -1.5)	-42.7 (-62.5 to -13)	-15.4 (-47.6 to 27.1)
	High fasting plasma glucose	YLDs	69.4 (41.4 to 103.5)	95.3 (57.5 to 140)	42.9 (23.5 to 67.2)	87.9 (55.6 to 125)	110.7 (70.9 to 153.8)	61.3 (37.1 to 89.1)	26.7 (12.7 to 48.2)	16.2 (3.2 to 34.9)	42.8 (22.8 to 79.5)
		Deaths	12.2 (6.4 to 25)	12.5 (6.4 to 26.9)	11.8 (6 to 23.4)	11.2 (5 to 22.6)	10.7 (4.6 to 21.6)	11.8 (4.9 to 25.4)	-8.2 (-40.3 to 34.3)	-14.2 (-46.5 to 36.6)	0.1 (-42.8 to 64.6)
		DALYs	218.2 (127.4 to 386.4)	225.9 (129.7 to 407)	208.7 (119.1 to 365.9)	234.3 (122.7 to 400.6)	232.6 (124.2 to 399.7)	235.8 (119.5 to 412.5)	7.4 (-25.7 to 51.7)	3 (-29.8 to 52.9)	13 (-26.9 to 77.6)
		YLLs	179.1 (102.9 to 326.4)	178.8 (99.9 to 328.7)	178 (100.8 to 319.7)	167.2 (81.2 to 293.4)	153.9 (74.4 to 269.2)	182.8 (82.8 to 337.9)	-6.7 (-39.9 to 37.2)	-13.9 (-46.7 to 37.9)	2.7 (-41.1 to 67.8)
	High LDL cholesterol	YLDs	39.1 (21.5 to 68.8)	47.1 (25.1 to 83.7)	30.6 (16.1 to 51.8)	67.1 (36 to 113.2)	78.7 (41.8 to 135.4)	53 (28.9 to 90)	71.7 (35.8 to 126.2)	67.2 (26.6 to 127.3)	73.1 (28.6 to 142.4)
		Deaths	8.1 (2.4 to 18.8)	9.1 (2.6 to 22)	6.8 (2.2 to 15.4)	6.6 (2 to 14)	6.6 (1.8 to 14.5)	6.5 (2 to 14.5)	-18.3 (-44.9 to 13.1)	-27.1 (-49.4 to 6.2)	-3.8 (-39.1 to 38.8)
		DALYs	170.4 (91.4 to 306.9)	196.6 (101.2 to 363.2)	141 (75.1 to 255)	162.5 (86.2 to 271.8)	172 (90.1 to 289.5)	150.8 (79.2 to 269.5)	-4.6 (-25.1 to 21)	-12.5 (-31.3 to 14.4)	6.9 (-24.2 to 42.4)
		YLLs	119.7 (56.6 to 237.2)	130.8 (56.4 to 275.6)	106 (51.2 to 204.9)	104.2 (45.2 to 190.4)	100.5 (42.1 to 192.5)	108.2 (47.1 to 209.3)	-12.9 (-42.1 to 24.4)	-23.2 (-49.8 to 16.3)	2.1 (-36.4 to 50.7)
	High systolic blood pressure	YLDs	50.7 (30.1 to 80.8)	65.7 (39 to 105.7)	35 (20.9 to 55.1)	58.3 (35 to 94)	71.5 (42.9 to 115.4)	42.6 (25.5 to 68.6)	15 (7.4 to 24.3)	8.8 (-0.7 to 19.9)	21.6 (8.7 to 37.3)
		Deaths	25.2 (18.4 to 33.9)	27 (18.1 to 37.7)	23 (16.9 to 30.9)	15.8 (10 to 22.3)	14.8 (8.7 to 21.6)	17 (10.3 to 24.8)	-37.5 (-57.3 to -12)	-45.2 (-65.6 to -15.6)	-26.3 (-55.5 to 8.4)
		DALYs	539.8 (424.3 to 682.1)	583.8 (430.1 to 757.9)	490.8 (377.1 to 632.1)	387.3 (281.4 to 503.4)	381 (266.2 to 509.2)	393.8 (278.7 to 523.5)	-28.3 (-45.9 to -6.1)	-34.7 (-53.2 to -9.7)	-19.8 (-45.4 to 9.1)
		YLLs	433.3 (330.3 to 568.4)	451.9 (323 to 608.4)	410.7 (308.2 to 545.2)	267.9 (174 to 366.9)	242.5 (149.4 to 342.2)	297.5 (187.6 to 423.9)	-38.2 (-58 to -13.8)	-46.3 (-65.4 to -18.6)	-27.6 (-57 to 5.8)
	High temperature	YLDs	106.4 (73.2 to 141.4)	132 (88.2 to 179.3)	80.1 (54.5 to 104.8)	119.4 (82.8 to 158.5)	138.5 (95.3 to 185.9)	96.3 (68.2 to 127.2)	12.2 (-1.4 to 29.7)	5 (-14.7 to 28.9)	20.2 (4.7 to 38.3)
		Deaths	0 (0 to 0)	0 (0 to 0)	0 (0 to 0)	0 (0 to 0)	0 (0 to 0)	0 (0 to 0)	-89.6 (-975 to 1070.3)	-89.1 (-893.3 to 892.6)	-90.6 (-1090.9 to 1367.8)
		YLLs	0.1 (-0.1 to 0.2)	0.1 (-0.1 to 0.2)	0.1 (-0.1 to 0.2)	0 (-0.2 to 0.3)	0 (-0.2 to 0.3)	0 (-0.2 to 0.3)	-89.2 (-944.4 to 995.2)	-88.8 (-868.9 to 851.8)	-90 (-1006.7 to 1328.5)
	Household air pollution from solid fuels	Deaths	2.2 (1.1 to 4)	2.7 (1.4 to 4.7)	1.6 (0.7 to 3.2)	0 (0 to 0)	0 (0 to 0.1)	0 (0 to 0)	-99.1 (-99.7 to -97.8)	-99.2 (-99.7 to -97.9)	-99.1 (-99.7 to -97.6)
		DALYs	52.6 (27 to 93.4)	66.5 (34 to 114.4)	37.5 (16.8 to 70.9)	0.5 (0.2 to 1.3)	0.6 (0.2 to 1.5)	0.4 (0.1 to 1)	-99 (-99.7 to -97.6)	-99.1 (-99.7 to -97.6)	-99 (-99.7 to -97.4)
		YLLs	41.2 (20.9 to 73.8)	50.4 (25.1 to 87.1)	31 (13.7 to 60.5)	0.3 (0.1 to 0.8)	0.4 (0.1 to 1)	0.3 (0.1 to 0.8)	-99.2 (-99.7 to -97.9)	-99.2 (-99.8 to -98)	-99.1 (-99.7 to -97.6)
		YLDs	11.4 (5.7 to 20.4)	16.1 (8.1 to 28.5)	6.5 (3 to 12.9)	0.2 (0.1 to 0.4)	0.2 (0.1 to 0.6)	0.1 (0 to 0.2)	-98.5 (-99.5 to -96.2)	-98.5 (-99.5 to -96.4)	-98.5 (-99.5 to -96.2)
	Kidney dysfunction	Deaths	4.3 (2.7 to 5.9)	5 (3 to 7)	3.5 (2.3 to 4.8)	3.7 (1.7 to 5.7)	3.6 (1.5 to 5.8)	3.8 (1.8 to 6)	-13.9 (-45.1 to 18)	-27.1 (-56.7 to 5.4)	7.8 (-38 to 54.8)
		DALYs	91.6 (68 to 117.5)	109.9 (79.6 to 144.7)	71.9 (52.7 to 93.8)	90.2 (60.1 to 122.1)	95.7 (62.9 to 132)	83.6 (53.4 to 116.4)	-1.5 (-25 to 25.7)	-12.9 (-34.3 to 13.2)	16.3 (-19 to 56.2)
		YLLs	71.8 (52.4 to 93.6)	82.6 (57.8 to 112.7)	59.8 (42.8 to 80.5)	61.9 (35.4 to 88.1)	60.2 (31.7 to 89.3)	63.8 (35 to 94.5)	-13.8 (-42.4 to 19.3)	-27.2 (-53 to 6.5)	6.8 (-31.2 to 53.1)
	Lead exposure	YLDs	19.8 (13.5 to 26.6)	27.3 (18.6 to 36.9)	12.1 (8.3 to 16.2)	28.4 (19.1 to 38.4)	35.5 (24.2 to 48.3)	19.7 (13.4 to 27.1)	43.2 (31.6 to 55.9)	30.3 (19.1 to 43.9)	63.4 (44.2 to 82.8)
		Deaths	2.2 (1.1 to 3.4)	2 (0.9 to 3.3)	2.4 (1.3 to 3.7)	1.1 (0.5 to 2)	0.9 (0.3 to 1.7)	1.4 (0.6 to 2.5)	-48.8 (-66.6 to -29.8)	-55.5 (-71.8 to -35.7)	-41 (-62.3 to -15.3)
		DALYs	44.6 (21.8 to 68.8)	40.5 (17 to 67.4)	48.8 (26.3 to 72.8)	20.6 (8.1 to 37)	16.4 (4.9 to 32.5)	25.6 (11.4 to 43.9)	-53.9 (-68.3 to -40.4)	-59.5 (-74.7 to -45.2)	-47.6 (-65.3 to -28.3)

Location	Risk factor	Measure	1990			2019			% Change (1990 to 2019)		
			Both	Female	Male	Both	Female	Male	Both	Female	Male
		YLLs	36.3 (17.7 to 56.9)	31.7 (13 to 52.4)	41.1 (22.3 to 62.6)	15.3 (6 to 28)	11.4 (3.4 to 22.9)	20.2 (8.8 to 35.5)	-57.8 (-72.9 to -42)	-64.1 (-78.6 to -47.6)	-50.9 (-69.8 to -28.7)
		YLDs	8.3 (3.6 to 13.9)	8.9 (3.3 to 15.7)	7.7 (3.8 to 12.1)	5.2 (1.7 to 9.8)	5.1 (1.3 to 10.4)	5.4 (2.3 to 9.3)	-37.1 (-54.8 to -25.2)	-43 (-65.5 to -30)	-29.7 (-45.5 to -16.5)
	Low physical activity	Deaths	3.6 (0.8 to 7.7)	3.7 (0.8 to 8.2)	3.5 (0.7 to 7.2)	2.9 (0.6 to 6.1)	2.7 (0.6 to 6.1)	3.1 (0.6 to 6.4)	-18.8 (-45.9 to 10.3)	-25.8 (-50.7 to 8.3)	-9.1 (-41.8 to 30.5)
		DALYs	54 (11.3 to 120.9)	56.5 (12.8 to 128.6)	51 (10.4 to 113.3)	49.6 (10.1 to 109.7)	48.4 (10 to 111.5)	51 (9.3 to 112.4)	-8.1 (-32.5 to 15.5)	-14.2 (-35.6 to 13.9)	0 (-31.1 to 35.1)
		YLLs	42.6 (9.1 to 94.7)	42.9 (9.6 to 99.9)	42.1 (8.6 to 90.9)	35.8 (7.4 to 78)	32.5 (6.7 to 74.2)	39.8 (7.2 to 87.4)	-16.1 (-45.3 to 13)	-24.3 (-50.2 to 9.2)	-5.3 (-38.9 to 36)
		YLDs	11.4 (2.1 to 28.4)	13.6 (2.6 to 34.3)	8.9 (1.5 to 22.2)	13.8 (2.5 to 33.6)	16 (3 to 39.1)	11.2 (1.8 to 26.8)	21.7 (7.9 to 41.6)	17.5 (1.1 to 42)	25.3 (7.7 to 48.3)
	Low temperature	Deaths	5.8 (3.9 to 8)	6.2 (4.2 to 8.7)	5.2 (3.5 to 7.3)	3.3 (1.9 to 4.7)	3.2 (1.9 to 4.8)	3.3 (1.8 to 5)	-42.9 (-58.6 to -23.7)	-48.2 (-62.4 to -27.5)	-35.3 (-57.7 to -10.9)
		YLLs	90.8 (62.1 to 125.9)	97 (65.2 to 137)	83.4 (55.7 to 117.4)	49.4 (27.2 to 72.1)	47.2 (27.2 to 71.1)	51.9 (27.4 to 78.8)	-45.6 (-61.8 to -26.3)	-51.3 (-66.1 to -30.7)	-37.8 (-59.7 to -12.8)
	Secondhand smoke	Deaths	1.9 (1.4 to 2.6)	2.1 (1.5 to 2.9)	1.7 (1.2 to 2.4)	1.3 (0.8 to 1.8)	1.2 (0.8 to 1.8)	1.3 (0.8 to 2)	-34.4 (-53.8 to -11.4)	-42 (-60.4 to -16.7)	-23.6 (-50.6 to 10.6)
		DALYs	45.4 (32.9 to 59.1)	52.9 (38.4 to 69.4)	37.4 (26.2 to 50.9)	31.7 (20.8 to 43.2)	33.4 (21.8 to 46.5)	29.7 (18 to 43.3)	-30.2 (-47.2 to -9.2)	-36.8 (-53 to -13)	-20.5 (-44.9 to 10.2)
		YLLs	39 (28 to 51.7)	44.1 (31.4 to 59.4)	33.4 (23.2 to 45.7)	24.5 (14.9 to 34.8)	24.1 (14.3 to 35.5)	24.9 (14.3 to 37.6)	-37.3 (-57.2 to -13.2)	-45.4 (-63.7 to -18.9)	-25.4 (-53.2 to 9.2)
		YLDs	6.4 (4.2 to 8.9)	8.8 (5.8 to 12.3)	4 (2.6 to 5.6)	7.2 (4.7 to 10)	9.3 (6.1 to 12.8)	4.9 (3.2 to 6.7)	12.6 (2.9 to 23.2)	6.2 (-4.7 to 19.3)	20.9 (2.9 to 40.7)
	Smoking	Deaths	8.7 (7.1 to 11)	8.1 (5.9 to 10.9)	9.4 (7.5 to 12)	6.5 (4.6 to 8.6)	5.9 (3.9 to 8.5)	7.1 (4.7 to 9.8)	-25.8 (-49 to 3.6)	-26.8 (-54.1 to 13.3)	-24.2 (-52 to 8.9)
		DALYs	235.9 (196.7 to 287.4)	230.2 (173.2 to 300.3)	242.8 (197.9 to 304.5)	194 (148.6 to 249)	192.5 (138.9 to 253)	195.6 (140.6 to 258.6)	-17.8 (-38.5 to 7.3)	-16.4 (-41 to 14.5)	-19.5 (-42.4 to 7.6)
		YLLs	184.3 (150.7 to 233.2)	170.6 (126.2 to 229.5)	198.9 (156 to 261)	129.5 (88.9 to 178)	117 (74.7 to 169.9)	144.3 (94.8 to 204.1)	-29.7 (-53.4 to -0.6)	-31.4 (-57 to 5.5)	-27.4 (-55.6 to 4.8)
		YLDs	51.6 (36.4 to 66.4)	59.6 (40.6 to 79.5)	43.9 (30.9 to 57)	64.6 (46.4 to 82.8)	75.5 (53.2 to 98)	51.2 (37 to 66.3)	25 (7.5 to 44.9)	26.8 (2.8 to 60.3)	16.7 (4.6 to 30.5)
Libya	All risk factors	Deaths	69.6 (54.2 to 89.9)	75.2 (57 to 98.4)	64.6 (48.6 to 87)	59.9 (45.4 to 80.6)	63.6 (46 to 82.8)	56.1 (40.1 to 82.1)	-14 (-33.2 to 14.3)	-15.5 (-36.8 to 13)	-13.1 (-36.2 to 19.5)
		DALYs	1515.1 (1221 to 1911.7)	1718.1 (1364.6 to 2172.6)	1343.7 (1037.8 to 1832.5)	1355.5 (1061.2 to 1716.9)	1502.2 (1138.1 to 1907.6)	1214 (894.2 to 1718.2)	-10.5 (-29.5 to 16.2)	-12.6 (-32 to 13.8)	-9.7 (-31.9 to 22.4)
		YLLs	1324.6 (1041.7 to 1706.6)	1469 (1122.1 to 1917.2)	1204.5 (904.6 to 1655.7)	1135.7 (847.2 to 1490.7)	1215.2 (868.5 to 1595.2)	1058.6 (741.2 to 1555.8)	-14.3 (-34.9 to 16.8)	-17.3 (-39.6 to 13.7)	-12.1 (-36.5 to 24.2)
		YLDs	190.5 (137.5 to 240.3)	249.2 (181.2 to 313.4)	139.2 (100.9 to 178.3)	219.8 (158.9 to 277.1)	287.1 (207.6 to 362.3)	155.4 (111.4 to 196.6)	15.4 (8.7 to 22.1)	15.2 (7.4 to 23.8)	11.6 (3.5 to 20.7)
	Alcohol use	Deaths	0 (0 to 0.1)	0 (0 to 0)	0 (0 to 0.1)	0.1 (0 to 0.2)	0 (0 to 0.1)	0.2 (0 to 0.4)	439.8 (-1589.2 to 3855.6)	1802.3 (-11045.4 to 11355)	406.5 (-345.1 to 2436.9)
		DALYs	1 (0.2 to 2.1)	0.2 (-0.2 to 0.6)	1.7 (0.3 to 3.5)	4.7 (0.1 to 7.9)	1.3 (-0.1 to 2.5)	8 (0.7 to 13.7)	373.1 (-130.7 to 1564.7)	657.3 (-4786.3 to 5376.2)	373.3 (-100 to 1552.4)
		YLLs	0.9 (0.2 to 2)	0.2 (-0.1 to 0.6)	1.6 (0.4 to 3.3)	4.2 (0.2 to 7.3)	1.1 (-0.1 to 2.1)	7.1 (0.6 to 12.6)	344 (-100.1 to 1463.3)	514.3 (-7424.7 to 8227.7)	350.6 (-100 to 1513)
		YLDs	0.1 (0 to 0.2)	0 (-0.1 to 0)	0.1 (0 to 0.3)	0.5 (0 to 0.9)	0.2 (-0.1 to 0.4)	0.9 (0.1 to 1.5)	855.8 (-7777.1 to 11175.9)	-1546.9 (-12851.7 to 7640.6)	688.2 (-2637.6 to 4872)
	Ambient particulate matter pollution	Deaths	14.5 (9.1 to 22)	14.9 (8.8 to 23)	14.3 (8.7 to 21.7)	16.5 (10.7 to 23.9)	17.5 (11.4 to 24.7)	15.4 (9.6 to 24.3)	13.4 (-17 to 66.3)	17.5 (-18 to 77.9)	8.2 (-23.5 to 64.1)
		DALYs	355.2 (223.4 to 517.9)	385.9 (232.4 to 570.5)	331.1 (207.2 to 483.9)	424.7 (287.5 to 593.5)	473.7 (317.2 to 643.4)	377.8 (242.4 to 577.7)	19.5 (-11.7 to 73.8)	22.8 (-11.6 to 84.3)	14.1 (-17.5 to 70.3)
		YLLs	308.8 (192.1 to 459)	327.9 (193.5 to 496.4)	294.6 (180.1 to 445.1)	352.7 (230.2 to 512.6)	379.4 (241.6 to 534.9)	327 (201.2 to 517.3)	14.2 (-19.3 to 68.6)	15.7 (-20.5 to 81.4)	11 (-23.1 to 72.1)
		YLDs	46.4 (27 to 68)	58 (32.6 to 87.3)	36.4 (21.7 to 53)	72 (48.3 to 99.2)	94.3 (63.5 to 129.5)	50.8 (33.2 to 70.3)	55.2 (29.1 to 109.8)	62.5 (31.8 to 125.1)	39.4 (18.6 to 86.5)
	Diet high in red meat	Deaths	3.7 (1.7 to 5.8)	4.1 (1.8 to 6.5)	3.4 (1.5 to 5.5)	2.1 (0.7 to 3.6)	2.2 (0.8 to 3.9)	1.9 (0.6 to 3.4)	-44.2 (-67.7 to -17.1)	-45.9 (-68.9 to -17.3)	-43.1 (-67.3 to -12.5)
		DALYs	104.7 (48.5 to 158.4)	122.5 (57.7 to 185.3)	90.5 (40.9 to 141.6)	61.3 (21.7 to 99.8)	68.8 (24.4 to 114.5)	54.2 (18.9 to 93.8)	-41.5 (-65.9 to -14.7)	-43.8 (-67.7 to -16)	-40.1 (-65 to -10.3)
		YLLs	90.4 (41.4 to 138.5)	103.5 (46.4 to 157.9)	80 (35.5 to 127.8)	50.5 (17.8 to 83.8)	54.6 (20.2 to 93.4)	46.5 (16.9 to 82.6)	-44.2 (-68 to -15.3)	-47.3 (-70.5 to -16)	-41.8 (-66.5 to -8)
		YLDs	14.4 (5.8 to 22.3)	19 (7.6 to 29.6)	10.5 (4.2 to 16.4)	10.9 (3.1 to 18.2)	14.2 (3.8 to 23.8)	7.7 (2.2 to 12.8)	-24.3 (-50.8 to -9.3)	-25.1 (-53.6 to -8.7)	-27.1 (-52.6 to -10)
	Diet high in sodium	Deaths	1.4 (0.2 to 5.6)	1.1 (0.2 to 4.4)	1.6 (0.1 to 7)	1.2 (0.2 to 4.9)	0.9 (0.2 to 3.7)	1.4 (0.1 to 6.4)	-15.2 (-48.9 to 57.1)	-19.4 (-61.6 to 80.8)	-12.1 (-52.5 to 88.7)
		DALYs	33.6 (4.2 to 136.3)	27.5 (4.8 to 110.7)	39.1 (2.9 to 163.2)	30.1 (3.7 to 121.9)	23 (4.2 to 96.7)	36.9 (2.5 to 152.9)	-10.4 (-48 to 71)	-16.2 (-60 to 89.4)	-5.7 (-44.4 to 91.5)
		YLLs	29.5 (3.6 to 121.5)	23.5 (4 to 96.6)	34.9 (2.6 to 147.5)	25.5 (3.1 to 104.4)	18.6 (3.3 to 80.1)	32 (2.2 to 136)	-13.8 (-51.1 to 65.5)	-20.8 (-62.7 to 82.3)	-8.4 (-46.6 to 91.9)
		YLDs	4.1 (0.5 to 16.2)	4 (0.7 to 16.4)	4.2 (0.3 to 16.3)	4.7 (0.6 to 18.7)	4.4 (0.8 to 18.7)	4.9 (0.3 to 18.3)	13.9 (-32.3 to 104.5)	11.1 (-47.9 to 144.4)	16.9 (-25.9 to 116.3)
	Diet low in fiber	Deaths	1.3 (0.3 to 2.7)	1.4 (0.3 to 3)	1.2 (0.3 to 2.7)	1.7 (0.4 to 3.6)	1.8 (0.4 to 3.8)	1.6 (0.3 to 3.6)	32.6 (-3.3 to 93.6)	31.7 (-9.9 to 99.3)	32 (-10.8 to 104)
		DALYs	31.2 (6.4 to 68.6)	35.1 (7.1 to 78.6)	28.2 (5.9 to 61.6)	44.8 (8.9 to 95.8)	49.3 (9.7 to 103.4)	40.6 (8.3 to 90.7)	43.6 (8 to 104.1)	40.3 (1.4 to 103.9)	44.2 (2.7 to 115.2)
		YLLs	27 (5.6 to 59.1)	29.6 (6 to 65.8)	24.9 (5.2 to 55.4)	37 (7.4 to 82.7)	39.2 (7.6 to 84.8)	35 (7 to 81)	37.4 (-0.4 to 101.8)	32.3 (-9 to 101.8)	40.4 (-4.6 to 119.2)
		YLDs	4.3 (0.8 to 9.4)	5.5 (1.1 to 12.3)	3.2 (0.6 to 7.1)	7.8 (1.5 to 15.9)	10.1 (1.9 to 21.1)	5.6 (1.1 to 11.3)	82.4 (54.1 to 129.5)	83.4 (47.6 to 144.8)	72.9 (40.3 to 128)
	Diet low in fruits	Deaths	2.7 (1.3 to 4.6)	3.1 (1.4 to 5.3)	2.4 (1.2 to 4.2)	2.5 (1 to 4.7)	2.7 (1 to 5.1)	2.3 (0.9 to 4.4)	-9.6 (-41.4 to 31.1)	-13.3 (-45.4 to 29.2)	-6.7 (-43.4 to 41.8)
		DALYs	74.8 (35.7 to 124.1)	89.6 (42.5 to 151.1)	62.8 (29.5 to 108.8)	70.4 (27.9 to 127)	80.2 (31.9 to 147.4)	61.2 (24.4 to 115.6)	-5.8 (-37.7 to 33.5)	-10.6 (-42.3 to 29.1)	-2.6 (-37.8 to 43.6)
		YLLs	65.4 (31.5 to 110.6)	77 (36.8 to 130.6)	56.2 (26.5 to 98)	58.6 (23.9 to 109.4)	64.4 (26.4 to 120.1)	53 (21.2 to 103.4)	-10.4 (-42 to 33.5)	-16.3 (-47.3 to 27.8)	-5.6 (-41.5 to 45.2)
		YLDs	9.4 (3.4 to 16.8)	12.7 (4.5 to 22.9)	6.6 (2.4 to 11.9)	11.8 (3.8 to 22.4)	15.7 (5 to 30)	8.1 (2.8 to 15.4)	26.4 (2 to 46.6)	24.3 (-1.7 to 47.7)	22.5 (-2.1 to 49)
	Diet low in vegetables	Deaths	1 (0.2 to 2.1)	1 (0.2 to 2.2)	0.9 (0.2 to 2.2)	1.2 (0.3 to 2.3)	1.2 (0.3 to 2.3)	1.2 (0.3 to 2.4)	24.3 (-25.6 to 112.5)	25.7 (-27.8 to 142.1)	22.1 (-29.3 to 114.2)
		DALYs	21.3 (4.4 to 50.5)	22.8 (4.6 to 54.6)	20.2 (4.3 to 48.7)	30 (7.5 to 59.1)	32.1 (7.2 to 63.6)	28 (7.1 to 58.1)	40.6 (-18 to 156.1)	40.9 (-19.8 to 177.8)	38.5 (-20 to 155.6)
		YLLs	18.6 (3.9 to 45.4)	19.3 (4 to 47.8)	18 (3.9 to 43.6)	25 (6.3 to 50.2)	25.7 (5.8 to 51.5)	24.2 (6.1 to 51.6)	34.5 (-23.5 to 158.6)	32.9 (-27.1 to 172.8)	34.7 (-24.7 to 156.7)
		YLDs	2.8 (0.5 to 6.1)	3.4 (0.6 to 7.8)	2.2 (0.4 to 4.9)	5 (1 to 9.9)	6.4 (1.2 to 12.6)	3.7 (0.8 to 7.3)	81.5 (27.7 to 198.3)	86 (24.5 to 224.1)	69.7 (20.5 to 174.1)
	Diet low in [illegible]	Deaths	3.8 (1.9 to 5.8)	4.1 (2.1 to 6.1)	3.5 (1.7 to 5.6)	4 (1.9 to 6)	4.3 (2 to 6.3)	3.7 (1.7 to 6.1)	6 (-19.3 to 41.8)	4.3 (-23.1 to 43)	7.3 (-23.4 to 50.9)

Location	Risk factor	Measure	1990			2019			% Change (1990 to 2019)		
			Both	Female	Male	Both	Female	Male	Both	Female	Male
	whole grains	DALYs	80.8 (40.5 to 118.9)	92.8 (45.4 to 134.8)	70.4 (34.4 to 110.3)	94.7 (41 to 139.3)	107 (46.5 to 156.8)	83 (36.1 to 131.5)	17.2 (-8.7 to 50.9)	15.3 (-11.4 to 50.8)	17.8 (-13.7 to 61.4)
		YLLs	64.9 (32.4 to 99)	71.6 (35.1 to 107.6)	59 (28.4 to 96.7)	75.6 (33.1 to 115.6)	81.5 (34.7 to 124.5)	69.9 (29.4 to 115.8)	16.5 (-14.4 to 59)	13.9 (-19.8 to 59.6)	18.3 (-17.9 to 72.1)
		YLDs	15.9 (7.6 to 23.9)	21.2 (10 to 31.9)	11.4 (5.3 to 17.3)	19.1 (8.5 to 28.9)	25.4 (11.5 to 38)	13.1 (5.8 to 19.9)	20.1 (9.3 to 28.9)	20 (7.5 to 31.9)	15.3 (2.1 to 29)
	High body-mass index	Deaths	18.4 (11.1 to 27.3)	23.3 (14.2 to 33.1)	14.1 (7.8 to 22.7)	18.7 (11.2 to 28.2)	21.5 (13.4 to 31.7)	15.9 (8.7 to 26.1)	1.4 (-25.3 to 40.9)	-7.7 (-33 to 28.3)	12.5 (-20.9 to 69.2)
		DALYs	585.3 (374.6 to 824)	762.5 (507.7 to 1045.6)	438.4 (257 to 661.9)	605.3 (392.5 to 858.3)	717.2 (473.8 to 1001.6)	498.7 (297.8 to 782.8)	3.4 (-20.6 to 38.1)	-5.9 (-28.4 to 27.6)	13.8 (-16.5 to 63.4)
		YLLs	508.4 (327 to 724.6)	651.6 (422.4 to 907.2)	389.8 (223.2 to 602.1)	499.9 (316.5 to 731.1)	573.3 (368.6 to 822.4)	429.7 (244.7 to 701.3)	-1.7 (-27.9 to 38.7)	-12 (-37.7 to 26.5)	10.2 (-22.9 to 66.2)
		YLDs	76.9 (47.3 to 110.1)	111 (70 to 156.8)	48.5 (28.5 to 72.5)	105.5 (68.7 to 147)	143.9 (95.2 to 198.6)	69 (42.6 to 97.5)	37.1 (24.3 to 55.1)	29.7 (17.6 to 46.6)	42.1 (23.7 to 72.3)
	High fasting plasma glucose	Deaths	21.7 (11.4 to 42.2)	22.1 (11.3 to 42.8)	21.3 (11.1 to 41.1)	23.1 (12.2 to 44.4)	24.6 (12.8 to 46)	21.6 (10.9 to 42.5)	6.7 (-20.7 to 49.6)	11.5 (-20.1 to 67.3)	1.5 (-28.4 to 48.6)
		DALYs	413.2 (241.1 to 701.7)	434.5 (241.4 to 749.9)	394.4 (221.9 to 689.6)	502.5 (284.9 to 866.2)	559 (322.6 to 933.7)	447.5 (248.1 to 797.9)	21.6 (-9.8 to 69.8)	28.7 (-8 to 91.3)	13.5 (-20 to 67.5)
		YLLs	363.1 (209.6 to 618.6)	372.8 (206.6 to 650.8)	355 (198.3 to 622.3)	421.1 (235.4 to 722.8)	452.6 (254.7 to 770.2)	390.4 (208.8 to 692.1)	16 (-16.9 to 67.8)	21.4 (-17.1 to 85.6)	10 (-25 to 68.6)
		YLDs	50.1 (26.2 to 87.3)	61.7 (31.8 to 109.4)	39.4 (20.1 to 70.6)	81.4 (44.6 to 135.6)	106.5 (57.7 to 178.6)	57.2 (30.7 to 95.8)	62.4 (33 to 109.3)	72.5 (33.4 to 137.6)	45.1 (19.8 to 91.4)
	High LDL cholesterol	Deaths	8.5 (3.1 to 19.4)	9.7 (3.6 to 22.1)	7.5 (2.6 to 16.9)	11 (4.5 to 22.2)	12 (5.1 to 24.3)	9.9 (3.8 to 20.2)	28.2 (-1.9 to 83.9)	24.1 (-8.7 to 79.3)	32.2 (-5.9 to 99.8)
		DALYs	192.7 (105.7 to 347.3)	232 (131.7 to 410.8)	158.9 (81.7 to 299.8)	277.1 (162.6 to 451.8)	321.1 (192.8 to 530.3)	235 (130.7 to 407)	43.8 (12.1 to 94.4)	38.4 (6.9 to 85.3)	47.9 (8.1 to 115.5)
		YLLs	151.8 (78.7 to 291)	175.6 (91.8 to 336.5)	131.1 (63.7 to 267.4)	218.4 (121.7 to 369.7)	241.6 (135.4 to 422.7)	196 (102.1 to 357.3)	43.9 (5.7 to 108.2)	37.6 (-3.1 to 103.3)	49.5 (2 to 132.9)
		YLDs	40.9 (24.1 to 67.5)	56.4 (33.1 to 91.6)	27.8 (16.4 to 46.3)	58.7 (36.4 to 94.2)	79.5 (48.9 to 126.8)	39 (23.6 to 62.9)	43.6 (31.7 to 58.1)	40.9 (27.4 to 57.2)	40.2 (24.4 to 61.2)
	High systolic blood pressure	Deaths	44.4 (32.4 to 59.7)	51.2 (37.1 to 69)	38.1 (26.6 to 54.4)	37.5 (26.4 to 51.9)	41.4 (28.4 to 56.2)	33.5 (22.2 to 50.6)	-15.6 (-36.4 to 13.6)	-19.2 (-41.3 to 11.2)	-12 (-37.6 to 23.2)
		DALYs	991.9 (765.2 to 1282.8)	1186.8 (909.5 to 1550.5)	822.9 (604.5 to 1146.9)	893.2 (668.4 to 1153.5)	1012.2 (733.6 to 1309.2)	777.9 (541.4 to 1104.3)	-9.9 (-30.1 to 17.9)	-14.7 (-36.6 to 13.6)	-5.5 (-30.8 to 29.6)
		YLLs	866.2 (653.3 to 1153.1)	1016.2 (750.6 to 1352.1)	736.8 (524.2 to 1057.2)	746 (537.3 to 1007.6)	817.9 (566.4 to 1102.5)	675.9 (454.7 to 1014.1)	-13.9 (-36 to 18.3)	-19.5 (-42.7 to 13.4)	-8.3 (-35.7 to 31.6)
		YLDs	125.6 (88.1 to 163.1)	170.6 (119.2 to 223.2)	86.2 (59.5 to 114.7)	147.1 (104.9 to 193.1)	194.3 (136.9 to 258.2)	102 (71.6 to 133.6)	17.1 (4.9 to 30.3)	13.9 (-2.6 to 32)	18.3 (3.7 to 35)
	High temperature	Deaths	1.3 (-0.6 to 5.1)	1.3 (-0.7 to 5.5)	1.2 (-0.6 to 4.8)	1.3 (-0.3 to 4)	1.4 (-0.3 to 4.3)	1.2 (-0.3 to 3.9)	5.1 (-187.6 to 261.2)	4.3 (-197.2 to 252.4)	5 (-179.9 to 261.9)
		YLLs	26.3 (-13 to 106.1)	28 (-14 to 116)	24.9 (-12.2 to 101.6)	25.1 (-6.1 to 78.1)	26.6 (-6.5 to 82.8)	23.6 (-5.4 to 72.8)	-4.7 (-177.2 to 223)	-5 (-184 to 225)	-5.6 (-168 to 219)
	Household air pollution from solid fuels	Deaths	6.7 (3.2 to 11.4)	8.3 (4 to 14.1)	5.3 (2.2 to 9.9)	0 (0 to 0.1)	0.1 (0 to 0.1)	0 (0 to 0.1)	-99.4 (-99.8 to -98.5)	-99.4 (-99.8 to -98.5)	-99.5 (-99.8 to -98.5)
		DALYs	165.1 (78.1 to 277.2)	215.5 (107.3 to 353.4)	122.3 (52.6 to 226.6)	1 (0.3 to 2.6)	1.4 (0.4 to 3.4)	0.7 (0.2 to 1.8)	-99.4 (-99.8 to -98.4)	-99.4 (-99.8 to -98.4)	-99.4 (-99.8 to -98.5)
		YLLs	142.9 (66.7 to 243.9)	183.1 (89.8 to 304.2)	108.9 (45.8 to 206.5)	0.8 (0.2 to 2.1)	1.1 (0.3 to 2.8)	0.6 (0.2 to 1.6)	-99.4 (-99.8 to -98.5)	-99.4 (-99.8 to -98.5)	-99.4 (-99.8 to -98.5)
		YLDs	22.2 (10.8 to 36.4)	32.4 (16.4 to 52.7)	13.4 (5.9 to 23.7)	0.2 (0.1 to 0.5)	0.3 (0.1 to 0.7)	0.1 (0 to 0.3)	-99.2 (-99.7 to -98)	-99.2 (-99.7 to -98)	-99.3 (-99.8 to -98.3)
	Kidney dysfunction	Deaths	6.5 (4.4 to 9.2)	7.7 (5.1 to 11.2)	5.5 (3.7 to 7.8)	7.7 (4.7 to 11)	8.6 (5.1 to 12.6)	6.8 (4.1 to 10.5)	18.2 (-12.7 to 59.8)	12.2 (-19.2 to 55.6)	25.1 (-14 to 76.8)
		DALYs	144.4 (107.5 to 191.8)	179.9 (132.3 to 244.6)	113.2 (82.1 to 156.8)	179.3 (125 to 238.2)	212.8 (148.5 to 287.9)	147.3 (99.3 to 212.1)	24.2 (-4.8 to 62.4)	18.3 (-10.5 to 57)	30 (-4.6 to 78.7)
		YLLs	124.3 (90.9 to 170.6)	151.4 (107.5 to 211.2)	100.6 (71.4 to 140.5)	148.6 (102 to 205)	170.2 (112.3 to 236.1)	127.8 (82.9 to 190.1)	19.5 (-13.3 to 64)	12.4 (-20.2 to 58.4)	27 (-11.3 to 80.7)
		YLDs	20.1 (13.6 to 26.9)	28.5 (19.3 to 38.3)	12.6 (8.5 to 16.9)	30.8 (21 to 41.3)	42.6 (29.2 to 57.3)	19.5 (13 to 26.4)	53.4 (42.5 to 65.1)	49.6 (37 to 63.7)	54.3 (39.5 to 70.7)
	Lead exposure	Deaths	3.1 (1.5 to 4.9)	2.7 (1.1 to 4.6)	3.4 (1.7 to 5.4)	1.9 (0.7 to 3.4)	1.6 (0.5 to 3.2)	2.1 (0.9 to 3.9)	-39.2 (-59.5 to -17.7)	-42 (-64.1 to -18.7)	-36.9 (-58.7 to -8.7)
		DALYs	68.3 (30.7 to 110.5)	63.4 (22.9 to 108.4)	72.8 (35.7 to 116.7)	35.3 (11.2 to 68.2)	31.1 (6.9 to 65.5)	39.3 (15.2 to 74.5)	-48.3 (-67.9 to -29.9)	-50.9 (-73.7 to -31)	-46 (-66 to -22.9)
		YLLs	60 (26.7 to 98.7)	54.4 (19.9 to 94)	65.3 (31.5 to 106.1)	30.2 (10 to 60)	25.6 (5.8 to 56.1)	34.6 (13.3 to 66.8)	-49.7 (-69.2 to -29.9)	-53 (-75.2 to -31.6)	-47 (-67.8 to -21.6)
		YLDs	8.2 (3.6 to 14.1)	9 (3.2 to 16.1)	7.6 (3.7 to 12.2)	5.1 (1.5 to 10.1)	5.6 (1.1 to 11.8)	4.7 (1.7 to 8.6)	-37.9 (-61.6 to -23.2)	-38.4 (-69.2 to -21.6)	-37.7 (-58 to -23.9)
	Low physical activity	Deaths	4.7 (1.1 to 9.8)	5 (1.2 to 10.6)	4.3 (0.8 to 9.2)	5.1 (1.2 to 10.9)	5.4 (1.3 to 11.4)	4.7 (1 to 10.4)	8.8 (-16.8 to 45)	7.9 (-20.8 to 48.1)	9.4 (-21.8 to 52.9)
		DALYs	74.6 (16.1 to 169.7)	84.3 (19.4 to 190.9)	65.8 (12.1 to 152.3)	89.1 (17.7 to 202.7)	100.1 (22.8 to 227.4)	78.4 (13.1 to 183.7)	19.4 (-7.3 to 53.5)	18.7 (-9.2 to 55.5)	19.2 (-12.4 to 63.1)
		YLLs	62.3 (13.6 to 138.7)	67.9 (15.9 to 153.4)	57.1 (10.9 to 130)	73.3 (15 to 165.8)	79 (18 to 179.8)	67.8 (12.1 to 158.7)	17.7 (-11.6 to 59.2)	16.3 (-16.6 to 61.6)	18.6 (-16.4 to 68)
		YLDs	12.4 (2.3 to 29.5)	16.4 (3.3 to 38.9)	8.7 (1.4 to 21.7)	15.8 (2.9 to 37.9)	21.1 (4.2 to 50.4)	10.7 (1.6 to 26.4)	27.8 (15.2 to 42.8)	28.5 (13.5 to 48.8)	22.9 (6.4 to 40.5)
	Low temperature	Deaths	3.4 (-0.3 to 7.7)	3.6 (-0.3 to 8.4)	3.2 (-0.3 to 7.5)	2.8 (-0.5 to 6.6)	3 (-0.6 to 7.2)	2.7 (-0.5 to 6.4)	-15.9 (-68.1 to 40.8)	-16.6 (-68 to 40.6)	-15.9 (-68 to 44.2)
		YLLs	70.9 (-6 to 159.9)	75.7 (-6.9 to 172.9)	67.2 (-5.8 to 155.3)	54.2 (-10 to 126.5)	57.5 (-11.3 to 135.9)	50.9 (-8.5 to 123.2)	-23.6 (-70.7 to 25.9)	-24 (-72.1 to 29.1)	-24.2 (-72.4 to 33.2)
	Secondhand smoke	Deaths	3.3 (2.3 to 4.5)	3.9 (2.7 to 5.4)	2.8 (1.9 to 4.1)	3 (2 to 4.3)	3.5 (2.3 to 4.9)	2.5 (1.5 to 3.8)	-9.7 (-31 to 23)	-10.9 (-35.4 to 23.9)	-11.5 (-37.2 to 25.8)
		DALYs	84.8 (58.2 to 114.8)	112.3 (78 to 153.2)	63.2 (41.8 to 91.8)	79.4 (53.5 to 110.4)	102.1 (69.2 to 141.6)	57.9 (36.9 to 88.6)	-6.4 (-28.5 to 25.3)	-9.1 (-32.8 to 24.8)	-8.4 (-34.2 to 29.6)
		YLLs	77.7 (53.1 to 106.2)	101.6 (69.4 to 140.7)	59.1 (38.7 to 87.1)	70.4 (46.3 to 99.9)	88.6 (58.6 to 127.6)	53.2 (32.8 to 83.5)	-9.4 (-32.7 to 24.9)	-12.8 (-38.3 to 25.3)	-9.9 (-37.3 to 30.5)
		YLDs	7.1 (4.6 to 9.7)	10.8 (7 to 14.8)	4.1 (2.7 to 5.9)	9 (5.9 to 12.4)	13.5 (8.8 to 18.6)	4.7 (3.1 to 6.6)	26.4 (15.6 to 38.8)	25.3 (12.5 to 40.6)	14 (-2.5 to 33.4)
	Smoking	Deaths	8.8 (6.6 to 11.9)	1.2 (0.8 to 1.8)	15.7 (11.7 to 21.5)	6.2 (4.4 to 8.9)	0.6 (0.4 to 0.9)	11.6 (8.1 to 16.7)	-29.2 (-50 to 0.4)	-49.6 (-70.3 to -18.1)	-26 (-48 to 5.7)
		DALYs	235.7 (181 to 321.9)	34.5 (23.3 to 50.7)	409.6 (312.6 to 561.7)	175.8 (124.8 to 246.9)	18.2 (11.7 to 26.7)	325.7 (229 to 463.3)	-25.4 (-45.4 to 3.6)	-47.1 (-68 to -16)	-20.5 (-41.6 to 10.4)
		YLLs	207.7 (154.3 to 290.2)	29.2 (19.3 to 43.6)	362.4 (266.3 to 513.1)	150.4 (102 to 219.7)	14.5 (9.1 to 22.2)	279.8 (188.4 to 413.1)	-27.6 (-49.9 to 5.5)	-50.3 (-71.5 to -17.4)	-22.8 (-46.5 to 13.8)
		YLDs	28 (19.9 to 36.2)	5.3 (3.4 to 7.6)	47.2 (33.8 to 61.3)	25.4 (18.1 to 32.4)	3.7 (2.3 to 5.5)	46 (32.9 to 58.6)	-9.3 (-18.5 to 0.1)	-29.2 (-53.4 to 6.3)	-2.6 (-12.5 to 7.7)
Morocc	All risk factors	Deaths	111.5 (86.3 to 133.6)	113.4 (83.5 to 141.7)	109.5 (83.5 to 141.1)	100.7 (79.6 to 122.9)	101.5 (78.7 to 127.1)	100 (76.7 to 129.4)	-9.6 (-27.7 to 11.8)	-10.5 (-30.2 to 15.3)	-8.7 (-30.4 to 17.4)
		DALYs	2322.5 (1881.9 to 2800.4)	2410.8 (1894.7 to 2964.1)	2230.9 (1748.2 to 2782.6)	1995.8 (1606.4 to 2406.6)	2075.6 (1647 to 2575.6)	1914.7 (1488.1 to 2439.4)	-14.1 (-32 to 6.4)	-13.9 (-32.6 to 9.5)	-14.2 (-35.3 to 8.5)

Location	Risk factor	Measure	1990 Both	1990 Female	1990 Male	2019 Both	2019 Female	2019 Male	% Change (1990 to 2019) Both	% Change (1990 to 2019) Female	% Change (1990 to 2019) Male
		YLLs	2102.1 (1663.2 to 2582.5)	2148.5 (1636 to 2695.4)	2054.2 (1567 to 2626.8)	1762.1 (1369.5 to 2153.1)	1791.8 (1370.7 to 2259.8)	1732.5 (1315.6 to 2253.8)	-16.2 (-35.4 to 6.2)	-16.6 (-37.4 to 9.5)	-15.7 (-38.4 to 9.6)
		YLDs	220.3 (161.1 to 280.3)	262.3 (191.2 to 335.4)	176.6 (131 to 226.7)	233.7 (170 to 294.8)	283.9 (206.8 to 356.9)	182.2 (133.2 to 233)	6.1 (0.4 to 12.4)	8.2 (0.6 to 16.7)	3.2 (-4.7 to 11.1)
	Alcohol use	Deaths	0.7 (0.3 to 1.2)	0 (0 to 0)	1.4 (0.6 to 2.4)	0.2 (-0.1 to 0.4)	0 (0 to 0)	0.3 (-0.2 to 0.8)	-78.2 (-119.6 to -49.5)	-219.9 (-1671.1 to 1370.2)	-78.3 (-119.4 to -50)
		DALYs	22.7 (11.4 to 36.8)	0.2 (-0.1 to 0.4)	45.7 (22.7 to 74.4)	6 (0 to 12.9)	-0.1 (-0.2 to 0.1)	12 (0 to 26.1)	-73.6 (-100.1 to -49.1)	-135.4 (-785.4 to 641.2)	-73.7 (-100.1 to -49.5)
		YLLs	21.3 (10.5 to 35)	0.2 (0 to 0.4)	42.9 (21.1 to 70.6)	5.5 (0.1 to 12)	0 (-0.1 to 0.1)	11 (0.1 to 23.9)	-74.2 (-99.6 to -49.8)	-124.7 (-662.2 to 228.3)	-74.4 (-99.5 to -50.2)
		YLDs	1.4 (0.6 to 2.4)	0 (0 to 0)	2.8 (1.1 to 4.8)	0.5 (-0.2 to 1.3)	0 (0 to 0)	1 (-0.3 to 2.5)	-64 (-124 to -31.6)	674.8 (-2228 to 2686.3)	-63.9 (-121.9 to -32)
	Ambient particulate matter pollution	Deaths	12.3 (6.3 to 20.1)	11 (5.4 to 18.7)	13.7 (7 to 22.4)	24.7 (18.4 to 32.2)	24.4 (17.7 to 32.4)	25 (17.5 to 33.6)	101.4 (30.5 to 271.6)	123 (38.6 to 324.9)	82.5 (15.3 to 233.4)
		DALYs	287.9 (143.3 to 476.6)	263.7 (126.4 to 447.8)	313.1 (158.9 to 517.4)	557.6 (417.5 to 724.5)	575.5 (421.6 to 751.5)	539.9 (381.1 to 727.1)	93.7 (24.3 to 264)	118.2 (34 to 324.8)	72.4 (7.7 to 215.8)
		YLLs	259.6 (129.7 to 431.3)	233.5 (111.8 to 397.1)	286.8 (142 to 479.6)	487.3 (353.7 to 647)	491 (345.6 to 659.4)	484 (334.3 to 670.1)	87.7 (17.2 to 257.7)	110.3 (27 to 318.3)	68.7 (5.2 to 211.6)
		YLDs	28.3 (14.4 to 47.7)	30.3 (14.7 to 52.4)	26.3 (13.2 to 43.3)	70.3 (49.1 to 93.8)	84.4 (57.9 to 113)	55.9 (39.4 to 73.9)	148.4 (69.8 to 328.7)	179 (83.1 to 401.2)	112.9 (48.1 to 275.2)
	Diet high in red meat	Deaths	3.5 (1.3 to 5.7)	3.5 (1.2 to 5.9)	3.5 (1.2 to 6)	3.6 (1.3 to 5.6)	3.6 (1.3 to 5.8)	3.5 (1.3 to 5.8)	2.4 (-33.2 to 59.9)	2.7 (-36.6 to 66.6)	1.8 (-35.3 to 66.8)
		DALYs	90.9 (31.3 to 148.3)	94.1 (32.3 to 155.9)	87.6 (30 to 149.7)	90.8 (33.1 to 143.8)	96.4 (35.1 to 155)	85.2 (32.7 to 138.9)	-0.1 (-35.1 to 59.6)	2.5 (-35.9 to 64.8)	-2.8 (-36.2 to 59.8)
		YLLs	81.3 (26.6 to 134.7)	82.7 (26.5 to 139.9)	79.9 (26.2 to 139.4)	78.2 (28.1 to 125)	81 (29.3 to 132.3)	75.3 (27 to 125)	-3.8 (-38.7 to 61.2)	-2 (-40.7 to 67.1)	-5.7 (-40 to 61.1)
		YLDs	9.6 (2.9 to 16.1)	11.4 (3.5 to 19.1)	7.7 (2.4 to 13)	12.6 (3.8 to 20.6)	15.4 (4.5 to 25.4)	9.9 (3.2 to 16.2)	31.7 (5.1 to 64.3)	35.1 (4.4 to 68.7)	27.3 (1.9 to 63.7)
	Diet high in sodium	Deaths	2.3 (0.3 to 9.4)	1.7 (0.3 to 7.2)	3 (0.3 to 12.4)	1.9 (0.3 to 7.7)	1.3 (0.3 to 5.5)	2.5 (0.2 to 10.2)	-16.8 (-53.9 to 51.4)	-20.1 (-63.7 to 73.6)	-15.3 (-54.6 to 92.7)
		DALYs	54.9 (6.6 to 225.9)	40.4 (6.8 to 173.1)	69.8 (5 to 278.9)	43.8 (5.7 to 173.3)	31.6 (5.8 to 134.2)	56.2 (4.5 to 226.8)	-20.4 (-55.3 to 47.4)	-21.7 (-63.6 to 89.5)	-19.5 (-53.4 to 72.4)
		YLLs	49.9 (6 to 206.2)	36 (6.1 to 154.4)	64.2 (4.6 to 255.6)	38.6 (4.9 to 153.3)	27.1 (4.9 to 115)	50.5 (4 to 206.2)	-22.5 (-57.5 to 44.6)	-24.6 (-65.3 to 80.8)	-21.3 (-55.3 to 69.2)
		YLDs	5.1 (0.6 to 20.2)	4.5 (0.7 to 18.6)	5.7 (0.4 to 22.4)	5.1 (0.6 to 20.3)	4.5 (0.8 to 18.9)	5.7 (0.4 to 22.3)	0.9 (-41 to 89.4)	1.3 (-54 to 130)	0.7 (-37.6 to 92)
	Diet low in fiber	Deaths	0.5 (0.3 to 0.9)	0.4 (0.2 to 0.8)	0.5 (0.3 to 1)	0.3 (0.2 to 0.6)	0.3 (0.2 to 0.6)	0.3 (0.2 to 0.6)	-29.3 (-54.1 to -0.4)	-27.6 (-56 to 11.2)	-31.9 (-59.8 to 6.8)
		DALYs	8.6 (5.2 to 14.7)	8.3 (5.1 to 14.5)	8.9 (5.2 to 16.5)	6.4 (4.3 to 10.6)	6.4 (4.3 to 11)	6.4 (4.1 to 10.8)	-25.7 (-46.2 to -0.6)	-22.7 (-46.5 to 7.5)	-28.9 (-52.7 to 2)
		YLLs	7.8 (4.7 to 13.4)	7.4 (4.4 to 12.9)	8.2 (4.6 to 15)	5.6 (3.7 to 9.4)	5.5 (3.6 to 9.3)	5.7 (3.6 to 9.8)	-28 (-48.9 to -1.2)	-25.5 (-49.6 to 6.6)	-30.6 (-55.6 to 1.7)
		YLDs	0.8 (0.5 to 1.5)	0.9 (0.5 to 1.7)	0.7 (0.4 to 1.3)	0.8 (0.5 to 1.4)	0.9 (0.5 to 1.7)	0.7 (0.4 to 1.2)	-3.9 (-21.4 to 14)	0.2 (-22.4 to 26.8)	-8.7 (-31.1 to 14.6)
	Diet low in fruits	Deaths	6.7 (3 to 11.6)	6.8 (3.1 to 11.6)	6.6 (2.9 to 12.1)	2.3 (1 to 4)	2.5 (1.1 to 4.6)	2.1 (0.9 to 3.9)	-65.6 (-80.6 to -44.4)	-63.3 (-80 to -39.8)	-68 (-83.8 to -44.7)
		DALYs	167 (77.1 to 285.7)	174.6 (80 to 299.6)	159.3 (69.6 to 288.2)	54 (24.6 to 94.5)	61.3 (27.4 to 109.7)	46.5 (20.6 to 86.1)	-67.7 (-81.5 to -46.2)	-64.9 (-81.1 to -40.9)	-70.8 (-84.4 to -48.4)
		YLLs	151.1 (69.9 to 262.8)	155.5 (70.8 to 269.4)	146.6 (64.2 to 270.9)	46.8 (21.8 to 82.4)	52.1 (23.4 to 92.7)	41.3 (18.4 to 76.7)	-69.1 (-82.6 to -46.9)	-66.5 (-82.5 to -41.5)	-71.8 (-85.3 to -49.1)
		YLDs	15.9 (6 to 29.1)	19 (7.1 to 34.6)	12.7 (4.7 to 23.2)	7.2 (2.5 to 13.2)	9.2 (3.1 to 17.8)	5.2 (1.8 to 9.9)	-54.6 (-70.5 to -33.4)	-51.7 (-69.9 to -29.3)	-58.9 (-75.1 to -36.1)
	Diet low in vegetables	Deaths	3.9 (1.3 to 7.1)	3.8 (1.1 to 7)	4 (1.3 to 7.6)	1.3 (0.3 to 2.6)	1.2 (0.3 to 2.4)	1.3 (0.3 to 2.7)	-68 (-86.5 to -33)	-69.1 (-87.8 to -32)	-67 (-86.6 to -25.3)
		DALYs	93.7 (27.7 to 173.1)	94.2 (25.7 to 173.7)	93.3 (28.5 to 178.7)	24.9 (6 to 52.8)	24.4 (5.6 to 53.3)	25.5 (6 to 53.5)	-73.4 (-90.2 to -38.1)	-74.1 (-91 to -36.5)	-72.6 (-89.9 to -34.8)
		YLLs	85 (23.9 to 159.1)	84.2 (21.9 to 158)	86.1 (25 to 167.5)	21.9 (5.3 to 46.5)	20.8 (5 to 45.4)	22.9 (5.5 to 48.6)	-74.3 (-90.5 to -39.7)	-75.2 (-91.6 to -37.8)	-73.4 (-90.4 to -34.5)
		YLDs	8.7 (2.7 to 15.5)	10.1 (3.1 to 17.9)	7.2 (2.2 to 12.8)	3.1 (0.6 to 6.7)	3.5 (0.7 to 8)	2.6 (0.5 to 5.8)	-64.5 (-85.5 to -29.9)	-65.1 (-86.3 to -28.9)	-63.7 (-86.2 to -28.6)
	Diet low in whole grains	Deaths	6.5 (3.9 to 9)	6.6 (3.8 to 9.5)	6.3 (3.6 to 9.4)	7.5 (4.3 to 10.4)	7.6 (4.3 to 10.5)	7.5 (4.1 to 10.7)	16.4 (-9.8 to 41.3)	15.2 (-11.7 to 48.1)	17.6 (-15.3 to 53.5)
		DALYs	128.5 (73.3 to 177.7)	134.7 (75 to 192.9)	122.3 (65.9 to 175.8)	150.7 (80.8 to 207.1)	158.8 (84.2 to 222)	142.6 (76.4 to 205.1)	17.3 (-9.1 to 42.6)	17.9 (-8.5 to 49.9)	16.6 (-14.7 to 51.4)
		YLLs	107.9 (62.3 to 151.9)	110.1 (60.7 to 162.1)	105.7 (57.5 to 156.3)	127.7 (67.8 to 181.8)	130.7 (69.3 to 185.6)	124.8 (65.7 to 180.6)	18.4 (-12 to 49.2)	18.7 (-13 to 58.9)	18.1 (-16.7 to 59.7)
		YLDs	20.7 (10.9 to 30)	24.6 (13 to 35.6)	16.6 (8.5 to 24.3)	23 (11.7 to 33.4)	28.1 (14.5 to 41.2)	17.8 (9 to 25.9)	11.4 (4.1 to 18.7)	14.3 (4.4 to 24.6)	7.5 (-2 to 17.4)
	High body-mass index	Deaths	21.4 (12.1 to 32.9)	24.7 (14.1 to 38.5)	18 (8.7 to 29.5)	23.8 (13.8 to 35.8)	26.6 (15.9 to 40.4)	21 (11 to 33.7)	11.4 (-18.6 to 58.4)	7.6 (-23.6 to 53.5)	16.5 (-21.5 to 82)
		DALYs	662.1 (393.9 to 988.5)	779.8 (470.2 to 1173.7)	542.1 (272.4 to 874.3)	695.1 (432.3 to 999.1)	803.6 (514.4 to 1164.8)	585.2 (333.4 to 907.4)	5 (-22.1 to 48.4)	3.1 (-25.2 to 45.4)	7.9 (-27.3 to 68.4)
		YLLs	595 (344.8 to 904.5)	691.6 (410.5 to 1056.2)	496.5 (246.2 to 818.8)	599.8 (360.3 to 884.7)	679.2 (417.2 to 1008.8)	519.2 (287.6 to 824.7)	0.8 (-27.8 to 46.7)	-1.8 (-31.3 to 43.3)	4.6 (-31.1 to 66)
		YLDs	67.1 (37.9 to 103.9)	88.3 (51.5 to 133.5)	45.6 (22.6 to 73.5)	95.3 (58.8 to 137.5)	124.4 (78.6 to 177.5)	66 (38.5 to 99.9)	42 (22.3 to 77.4)	41 (20.5 to 72.1)	44.8 (18.1 to 107)
	High fasting plasma glucose	Deaths	19.2 (11.1 to 35.3)	19.6 (10.4 to 39.6)	18.7 (11.1 to 32.2)	38 (20.3 to 69.3)	37.3 (19.7 to 69.7)	38.8 (20.8 to 69.7)	98.5 (36.6 to 176)	90.4 (22.1 to 188.7)	107.6 (37.5 to 198)
		DALYs	364.3 (233.2 to 605.9)	374.3 (222.5 to 668.9)	353.5 (227.8 to 574.9)	720.2 (429.3 to 1202.1)	728.1 (427.4 to 1237.1)	712.8 (418.2 to 1158.7)	97.7 (40.1 to 173.2)	94.5 (24.7 to 183.9)	101.6 (37.4 to 184.4)
		YLLs	331 (211.8 to 554.8)	335.2 (196 to 599.5)	326.4 (207.7 to 535)	640.9 (378.3 to 1048.2)	632.8 (370.2 to 1074.2)	649.8 (374.2 to 1067.4)	93.6 (34.9 to 170.3)	88.8 (19.8 to 181)	99.1 (33.3 to 184.9)
		YLDs	33.3 (18.5 to 57.8)	39.1 (20.8 to 69.8)	27.2 (15.7 to 46.3)	79.3 (42.9 to 136.4)	95.3 (51.2 to 165.4)	63 (33.2 to 106.8)	138.3 (86.9 to 201.6)	143.5 (74.6 to 224.5)	131.7 (75.5 to 194.2)
	High LDL cholesterol	Deaths	14.1 (4.7 to 31.7)	15.2 (5.2 to 35)	12.8 (4.6 to 28.5)	17.7 (6.6 to 38.5)	19.2 (7.1 to 41.7)	16.2 (5.7 to 35.2)	25.7 (-3.6 to 58)	26.5 (-3.4 to 70.7)	26 (-12.5 to 67.3)
		DALYs	297.6 (163.7 to 544.6)	330.1 (180.2 to 605)	263.3 (139.8 to 490)	378.2 (204.9 to 661.9)	428.3 (236.6 to 736.2)	326.8 (165.2 to 608.9)	27.1 (-1.6 to 60.2)	29.8 (-2 to 68.8)	24.1 (-10.6 to 63.7)
		YLLs	244.9 (125.9 to 466.4)	264.5 (130.6 to 516.2)	223.9 (112.1 to 438.7)	315.1 (158.8 to 568.4)	347.7 (178.9 to 625.5)	281.7 (136.8 to 541)	28.7 (-5.6 to 70.9)	31.4 (-5.7 to 82.9)	25.8 (-15.2 to 73.7)
		YLDs	52.8 (32.2 to 85.7)	65.6 (39.8 to 106.1)	39.4 (23.2 to 64.8)	63.1 (38.4 to 100.7)	80.7 (49.2 to 127.5)	45.1 (27 to 72.9)	19.4 (11.9 to 28.8)	23 (12.2 to 35.8)	14.5 (3.1 to 27.5)
	High systolic blood pressure	Deaths	78.4 (57.7 to 101)	85.4 (61 to 111.5)	70.7 (51.2 to 99.4)	65.8 (49 to 86.2)	70.6 (51.7 to 94.2)	60.8 (43 to 85.5)	-16.1 (-34.9 to 6.5)	-17.4 (-38.6 to 9)	-14 (-36.5 to 14.5)
		DALYs	1684.8 (1300.5 to 2108.9)	1860.1 (1385.4 to 2357.5)	1501.6 (1133 to 1989.8)	1360.9 (1030.4 to 1725.8)	1492.6 (1142.5 to 1945.3)	1224.7 (897.1 to 1630.3)	-19.2 (-36.9 to 1.7)	-19.8 (-40 to 5.4)	-18.4 (-39.6 to 6.9)
		YLLs	1524.8 (1146.9 to 1926.8)	1659.9 (1199.8 to 2145.7)	1383.2 (1017.3 to 1858.2)	1199.1 (895.1 to 1547.2)	1288.6 (951.3 to 1722.7)	1106.2 (783.7 to 1494.6)	-21.4 (-40.4 to 2.1)	-22.4 (-43.9 to 4.6)	-20 (-42.4 to 7.5)

Location	Risk factor	Measure	1990			2019			% Change (1990 to 2019)		
			Both	Female	Male	Both	Female	Male	Both	Female	Male
		YLDs	160 (115 to 207.8)	200.2 (141.1 to 259.6)	118.4 (82.7 to 155.3)	161.8 (115 to 211.2)	204 (141.3 to 266.6)	118.5 (83.7 to 155)	1.1 (-8.5 to 11.7)	1.9 (-11.6 to 17.6)	0.1 (-12 to 13.9)
	High temperature	Deaths	0.7 (-0.1 to 2.1)	0.7 (-0.1 to 2.1)	0.7 (-0.1 to 2.1)	0.6 (0 to 1.7)	0.6 (0 to 1.7)	0.6 (0 to 1.7)	-10.4 (-158.8 to 145.5)	-10.7 (-156.9 to 143)	-10.3 (-157.6 to 152.9)
		YLLs	13.4 (-2.3 to 38)	13.5 (-2.4 to 39.9)	13.2 (-2.4 to 37.5)	11 (0.2 to 28.9)	11 (0.2 to 28.2)	10.9 (0.2 to 28.3)	-18 (-153.2 to 123)	-18.4 (-151.5 to 113.3)	-17.6 (-155.3 to 124.7)
	Household air pollution from solid fuels	Deaths	18.5 (10.5 to 29)	20.2 (12 to 31.3)	16.7 (8.6 to 27.8)	1.6 (0.7 to 3.1)	1.9 (0.8 to 3.6)	1.3 (0.5 to 2.7)	-91.5 (-96 to -84.1)	-90.8 (-95.6 to -82.6)	-92.5 (-96.6 to -85.1)
		DALYs	433.6 (250.3 to 662.5)	485.7 (293.9 to 740.6)	379.7 (195.9 to 623.8)	35.6 (15.6 to 69.3)	44 (19.1 to 85)	27.1 (10.4 to 57.8)	-91.8 (-96.2 to -84.5)	-90.9 (-95.7 to -82.8)	-92.9 (-96.8 to -85.7)
		YLLs	389.6 (223.3 to 602)	430 (259.5 to 663.7)	347.9 (179.8 to 575.1)	31 (13.3 to 60.8)	37.5 (15.8 to 74)	24.3 (9.1 to 52.6)	-92 (-96.3 to -84.8)	-91.3 (-95.9 to -83.3)	-93 (-96.9 to -85.8)
		YLDs	43.9 (23.8 to 67.6)	55.7 (31.6 to 85.1)	31.8 (15.7 to 50.5)	4.6 (2 to 9)	6.4 (2.8 to 12.5)	2.8 (1.1 to 5.8)	-89.5 (-94.8 to -80.5)	-88.5 (-94.4 to -78.9)	-91.2 (-96 to -82.9)
	Kidney dysfunction	Deaths	8.9 (6.2 to 12.5)	9.7 (6.3 to 14)	8.1 (5.6 to 11.8)	12.5 (7.7 to 17)	12.7 (7.4 to 17.7)	12.4 (7.5 to 17.2)	40.9 (2.9 to 81.9)	31.1 (-6.2 to 75.9)	53.4 (7.7 to 104.2)
		DALYs	190.2 (144.9 to 249)	213.2 (152.7 to 287.1)	166.6 (124.2 to 232.5)	260.8 (185 to 334.2)	277.1 (194.4 to 359.9)	244.4 (169.3 to 322.8)	37.1 (3.5 to 72.3)	30 (-2.6 to 69.5)	46.8 (8.2 to 90.7)
		YLLs	170.1 (125.4 to 226.8)	187.5 (132.1 to 258)	152.2 (111.2 to 216.1)	228.9 (161 to 297.1)	237 (160.1 to 315.5)	220.9 (147.7 to 295.1)	34.5 (-2.2 to 74.4)	26.4 (-9.3 to 72.3)	45.1 (3.1 to 93.1)
		YLDs	20 (13.7 to 27)	25.6 (17.5 to 34.8)	14.3 (9.7 to 19.4)	31.9 (21.7 to 43.1)	40.1 (27.3 to 54.1)	23.5 (16.2 to 32.2)	59.3 (45.1 to 74.4)	56.5 (41.3 to 72.9)	64.3 (44.6 to 85.1)
	Lead exposure	Deaths	5.9 (3.1 to 9.3)	5 (2.4 to 8.4)	6.8 (3.7 to 10.7)	4.7 (2.4 to 7.4)	3.8 (1.6 to 6.5)	5.5 (3.1 to 8.8)	-20.7 (-39.9 to 1.3)	-24.1 (-44.2 to -1.2)	-18.6 (-40.2 to 7.4)
		DALYs	128.1 (66.3 to 199.5)	110.2 (49.7 to 181.2)	146.7 (80.7 to 228.1)	86 (41.7 to 138.9)	70.9 (27.6 to 126.3)	101.6 (54.4 to 161.3)	-32.9 (-49.7 to -14.3)	-35.7 (-54.6 to -17)	-30.8 (-49.8 to -8.9)
		YLLs	116.5 (60.1 to 183.8)	98.6 (44.1 to 164)	135.3 (73.2 to 213.8)	77.1 (37.5 to 125.4)	62 (24.5 to 111.1)	92.7 (48.6 to 148.5)	-33.9 (-51.4 to -13.3)	-37.1 (-56.6 to -16.3)	-31.5 (-51.8 to -7.6)
		YLDs	11.5 (5.7 to 18.6)	11.6 (4.9 to 19.5)	11.4 (6.1 to 18)	8.9 (3.9 to 15.3)	8.9 (3.2 to 16)	8.9 (4.2 to 14.6)	-23 (-37.5 to -12.7)	-23.8 (-43.1 to -10.9)	-22.2 (-35.6 to -10.1)
	Low physical activity	Deaths	6.4 (1.4 to 13.7)	6.5 (1.5 to 14)	6.3 (1.3 to 13.7)	8.2 (1.8 to 16.7)	8.4 (2 to 17)	8 (1.5 to 16.9)	28.1 (-0.4 to 61.5)	28.2 (-2.4 to 73.9)	27.8 (-6.9 to 69.2)
		DALYs	97.6 (20.2 to 218.6)	102.8 (21.1 to 233.5)	92.4 (17 to 215.3)	126.5 (25.2 to 277.9)	133.4 (29.2 to 298.2)	119.4 (21.1 to 266.9)	29.5 (2 to 60.6)	29.8 (-0.7 to 70.6)	29.2 (-5.9 to 69.6)
		YLLs	85 (17.6 to 189)	87.6 (18.5 to 196.6)	82.5 (14.7 to 190.5)	110.8 (22.6 to 241.2)	114.1 (25.8 to 250.8)	107.5 (18.7 to 242.7)	30.3 (-0.9 to 64.3)	30.2 (-4.1 to 77.5)	30.3 (-8.3 to 74.1)
		YLDs	12.6 (2.3 to 32)	15.1 (3 to 37.6)	9.9 (1.6 to 26.1)	15.6 (2.9 to 38.7)	19.3 (3.7 to 46.7)	11.9 (2 to 30)	24.1 (11.9 to 42)	27.4 (12 to 49.7)	20 (2.3 to 44.2)
	Low temperature	Deaths	9.7 (6 to 14.7)	9.7 (5.9 to 14.7)	9.7 (5.9 to 15)	8.5 (5.4 to 12.9)	8.4 (5.2 to 12.6)	8.5 (5.2 to 13.2)	-12.2 (-29.5 to 8.6)	-12.6 (-31.9 to 12.1)	-12 (-33 to 11.5)
		YLLs	180.7 (111.6 to 271.2)	183 (113.7 to 280.5)	178.5 (109.1 to 274.8)	145.2 (90.2 to 218.3)	146.3 (88.8 to 220)	144.2 (88.7 to 222.5)	-19.6 (-37.7 to 0.6)	-20 (-39.1 to 4)	-19.2 (-39.8 to 4.1)
	Secondhand smoke	Deaths	4.8 (3.3 to 6.5)	5.5 (3.7 to 7.5)	4.1 (2.7 to 5.9)	3.3 (2.2 to 4.4)	3.6 (2.4 to 5)	2.9 (1.9 to 4.3)	-31.6 (-47.8 to -13.8)	-33.9 (-50.5 to -14.2)	-28.2 (-47.9 to -6.4)
		DALYs	115.4 (80.1 to 153.7)	140.6 (96.6 to 190.5)	89.5 (60 to 127.5)	76.1 (52.3 to 103.5)	90.1 (59.4 to 125.2)	62 (40.1 to 87.9)	-34.1 (-50.5 to -14.7)	-35.9 (-53 to -14.2)	-30.8 (-50.5 to -8.6)
		YLLs	108.1 (74 to 145.2)	130.4 (88.3 to 178.5)	85.1 (56.3 to 122.3)	69.9 (47.4 to 95.5)	81.5 (52.2 to 115.6)	58.1 (36.9 to 83.4)	-35.4 (-52.7 to -14.7)	-37.5 (-55.3 to -14.6)	-31.7 (-52.1 to -8.7)
		YLDs	7.3 (4.7 to 10.2)	10.2 (6.6 to 14.1)	4.4 (2.9 to 6.2)	6.2 (4.1 to 8.7)	8.6 (5.5 to 12)	3.8 (2.5 to 5.4)	-15.2 (-22 to -7.9)	-15.8 (-24.3 to -7.1)	-12.6 (-23.6 to 0.3)
	Smoking	Deaths	12.1 (9.4 to 15.6)	2.4 (1.6 to 3.4)	22.3 (17 to 29.1)	7.3 (5.4 to 9.5)	1.3 (0.9 to 1.8)	13.6 (9.9 to 17.9)	-39.4 (-55.9 to -20.2)	-44.8 (-62.9 to -17.3)	-39 (-56 to -18.3)
		DALYs	326.5 (256.4 to 422.8)	68.7 (47.6 to 97.4)	590.6 (458.5 to 778.2)	192.6 (143.6 to 248.6)	36.9 (25.2 to 53)	351.2 (256.8 to 462)	-41 (-56.8 to -22.1)	-46.3 (-64.8 to -19.9)	-40.5 (-56.8 to -21)
		YLLs	296.8 (226.2 to 386.2)	60.5 (41 to 87.3)	539 (406.2 to 713.9)	170.4 (122.4 to 225.3)	31.3 (20.5 to 46.1)	312.3 (220.5 to 420.3)	-42.6 (-59.3 to -22)	-48.3 (-67.4 to -20.6)	-42.1 (-58.7 to -20.7)
		YLDs	29.7 (21.2 to 38.4)	8.2 (5.3 to 11.9)	51.6 (37 to 66.6)	22.2 (16 to 28.8)	5.7 (3.7 to 8)	39 (27.8 to 50.5)	-25.2 (-32.7 to -16.1)	-31.2 (-50.2 to -3.1)	-24.5 (-32.4 to -15)
Oman	All risk factors	Deaths	114 (86.4 to 146.6)	102.9 (75.4 to 136.3)	129.2 (95.8 to 168.2)	87.7 (74.8 to 102.8)	82.1 (67.6 to 97.8)	95.2 (78.2 to 116.2)	-23.1 (-41.6 to 4.8)	-20.2 (-42.1 to 14.6)	-26.4 (-46.4 to 3.8)
		DALYs	2408.2 (1845.8 to 3129.9)	2220 (1681.2 to 2854.9)	2622.7 (1948.6 to 3398.3)	1610.3 (1402.6 to 1845.1)	1591.6 (1358.3 to 1856.5)	1659.3 (1382.2 to 1975.8)	-33.1 (-48 to -12.1)	-28.3 (-46.3 to -1.3)	-36.7 (-53.2 to -12.7)
		YLLs	2199.7 (1631.6 to 2894.6)	1993.1 (1459 to 2631.8)	2427.3 (1747.5 to 3208.1)	1404.2 (1208 to 1625.8)	1355.6 (1126.4 to 1606.1)	1473.6 (1214.7 to 1784.8)	-36.2 (-51.7 to -13.3)	-32 (-51.5 to -2.5)	-39.3 (-56.1 to -13.8)
		YLDs	208.5 (150.3 to 266.4)	227 (164.6 to 288.6)	195.5 (141.3 to 252.7)	206.2 (150.9 to 262.4)	236 (172.8 to 303.6)	185.7 (133.4 to 238.6)	-1.1 (-6.7 to 5.1)	4 (-4.4 to 12.8)	-5 (-13 to 2.6)
	Alcohol use	Deaths	0 (-0.2 to 0.3)	-0.1 (-0.2 to 0.1)	0.1 (-0.4 to 0.6)	0 (-0.2 to 0.2)	-0.1 (-0.2 to 0)	0 (-0.3 to 0.4)	-280.6 (-584.6 to 980.3)	68.1 (-649.1 to 950)	-90.6 (-565.1 to 314.7)
		DALYs	2.2 (-4.4 to 9.6)	-1.2 (-3.9 to 2)	4.4 (-7.2 to 17.9)	0.4 (-4.2 to 5.3)	-2 (-4.3 to 0.4)	1.9 (-6 to 10.3)	-82.9 (-661.9 to 391.3)	72.6 (-1161.7 to 1021.4)	-56.2 (-503.7 to 421.2)
		YLLs	2.4 (-3.7 to 9.4)	-0.8 (-3.3 to 2.1)	4.5 (-6.2 to 16.9)	0.5 (-3.3 to 4.8)	-1.5 (-3.5 to 0.6)	1.8 (-5 to 9.2)	-78.4 (-637.7 to 332.6)	92 (-958.8 to 1141.8)	-59.2 (-423.8 to 254.9)
		YLDs	-0.2 (-0.8 to 0.5)	-0.4 (-0.8 to -0.1)	-0.1 (-1.1 to 1.1)	-0.1 (-0.9 to 0.6)	-0.5 (-1 to -0.1)	0.1 (-1 to 1.4)	-27.7 (-376.7 to 565.4)	35.8 (-3.4 to 97.2)	-316.1 (-597.4 to 412.8)
	Ambient particulate matter pollution	Deaths	22 (11.9 to 34.7)	18.1 (9.2 to 29.7)	26.7 (14.3 to 42.5)	25.4 (19 to 32.5)	23.9 (17.6 to 31.2)	27.3 (20 to 36.3)	15.4 (-22.3 to 99)	32.4 (-13.6 to 145.6)	2.2 (-33.5 to 76.5)
		DALYs	505 (271.7 to 797.7)	422.4 (213 to 689.2)	586.5 (316.7 to 925.2)	515.7 (388.7 to 651.2)	516.1 (394.1 to 655.5)	526.4 (389.9 to 687.1)	2.1 (-30.9 to 69.8)	22.2 (-20.4 to 123.9)	-10.3 (-41.5 to 53.4)
		YLLs	459.8 (240 to 735.5)	377.2 (188.8 to 632.7)	540.5 (288.7 to 867.9)	443.6 (332 to 561.1)	432.8 (318.7 to 555.4)	461.1 (338.3 to 603.8)	-3.5 (-35.6 to 65.2)	14.7 (-26.3 to 118.4)	-14.7 (-45.4 to 47.9)
		YLDs	45.2 (23.6 to 71.2)	45.2 (22.6 to 73.3)	46 (24.4 to 70.9)	72.1 (49.9 to 98.7)	83.3 (58.5 to 115.3)	65.2 (44.9 to 89.6)	59.5 (17.3 to 150.4)	84.4 (30.1 to 210.6)	41.7 (5.7 to 120.7)
	Diet high in red meat	Deaths	6.4 (2.6 to 10.1)	6 (2.3 to 9.6)	6.9 (2.9 to 11.1)	4.9 (2.3 to 7.1)	4.7 (2.4 to 7.1)	5.1 (2.2 to 7.5)	-23.4 (-46 to 12.7)	-20.3 (-48 to 33.2)	-25.9 (-49.4 to 13.8)
		DALYs	162.5 (68.7 to 256)	155.8 (62.2 to 248.4)	170 (71.3 to 270)	112.1 (56.9 to 157.9)	115.8 (60.1 to 167.8)	111.4 (51.5 to 160.3)	-31 (-50.1 to 1.6)	-25.7 (-49.1 to 19)	-34.4 (-54.2 to -0.2)
		YLLs	146.8 (61.2 to 236.2)	137.9 (51.6 to 225.8)	155.6 (65.4 to 250.2)	94.2 (47.2 to 135.6)	94.6 (47.9 to 139.4)	95.4 (44 to 139.6)	-35.9 (-54.6 to -3.1)	-31.4 (-54.2 to 16.3)	-38.7 (-58.9 to -3.8)
		YLDs	15.7 (6.1 to 24.4)	17.9 (7.4 to 27.8)	14.4 (5.3 to 22.8)	17.9 (8.2 to 27)	21.2 (10.1 to 32.4)	16.1 (7.1 to 24.4)	14.1 (0.2 to 45.5)	18.3 (0.7 to 54)	11.8 (-4.8 to 46.2)
	Diet high in sodium	Deaths	2.4 (0.4 to 10.2)	1.6 (0.3 to 6.8)	3.3 (0.3 to 14)	1.6 (0.3 to 6.6)	1.1 (0.3 to 4.5)	2.1 (0.2 to 8.6)	-34.2 (-60.5 to 34)	-30.9 (-66.2 to 68.8)	-37.1 (-64.7 to 50.5)
		DALYs	56.6 (6.9 to 241.4)	36.8 (6.8 to 166)	74.2 (5.9 to 324.3)	33.4 (4.6 to 136.8)	23.7 (4.6 to 99.5)	41.8 (4.1 to 173.1)	-40.9 (-63.6 to 20.5)	-35.6 (-69.8 to 59.1)	-43.7 (-66.7 to 26)
		YLLs	51.8 (6.2 to 220.1)	33.1 (6 to 149.4)	68.5 (5.4 to 301.6)	28.9 (4 to 117.9)	20.1 (3.9 to 84.2)	36.6 (3.6 to 149.7)	-44.2 (-66 to 17.4)	-39.3 (-71.7 to 54.7)	-46.6 (-69.2 to 22.3)
		YLDs	4.8 (0.6 to 19.9)	3.7 (0.6 to 15.3)	5.7 (0.4 to 23.3)	4.5 (0.5 to 18.3)	3.6 (0.6 to 14.4)	5.2 (0.4 to 20.9)	-5.4 (-39.8 to 68.9)	-2.9 (-52.5 to 107.2)	-8.7 (-39.3 to 86.1)

Location	Risk factor	Measure	1990			2019			% Change (1990 to 2019)		
			Both	Female	Male	Both	Female	Male	Both	Female	Male
	Diet low in fiber	Deaths	4.4 (0.9 to 8.9)	4 (0.8 to 8.1)	4.9 (1 to 9.5)	1.3 (0.3 to 2.7)	1.2 (0.3 to 2.7)	1.5 (0.4 to 3.2)	-69.6 (-79.4 to -53.1)	-70 (-82.2 to -49.6)	-69.5 (-81 to -50.3)
		DALYs	100.1 (18.7 to 201.7)	94.3 (17.9 to 188.7)	106.9 (20.2 to 213.7)	25.8 (6.2 to 54.7)	24.8 (6 to 55.5)	27.1 (6.8 to 58.7)	-74.3 (-82 to -61)	-73.7 (-83.1 to -58)	-74.6 (-83.7 to -60.9)
		YLLs	90.3 (16.7 to 184.6)	83.1 (15.5 to 168.6)	97.8 (18.4 to 199.7)	21.8 (5.4 to 46.4)	20.3 (5 to 46)	23.5 (5.9 to 51.5)	-75.8 (-83.3 to -62.7)	-75.5 (-84.9 to -59.4)	-75.9 (-84.8 to -62)
		YLDs	9.8 (1.9 to 19.2)	11.2 (2 to 22.3)	9 (1.7 to 17.7)	3.9 (0.9 to 8.6)	4.5 (0.9 to 10.2)	3.6 (0.8 to 8.1)	-60.1 (-69.7 to -48.6)	-59.6 (-71.7 to -46)	-60.1 (-71.3 to -47.8)
	Diet low in fruits	Deaths	4.1 (2 to 6.7)	4.2 (2.1 to 7)	4 (1.8 to 6.9)	1.6 (0.8 to 2.7)	1.7 (0.8 to 3)	1.6 (0.6 to 3)	-60.6 (-73.9 to -41.6)	-59.3 (-75.8 to -33.5)	-61.4 (-77.8 to -35.6)
		DALYs	101.6 (50.5 to 164.6)	107.8 (53.3 to 180.5)	97.6 (43.6 to 164.3)	35.2 (17.3 to 57.8)	40.4 (19.4 to 67.1)	32.2 (13.7 to 56.8)	-65.4 (-76.3 to -50.7)	-62.5 (-75.7 to -42.8)	-67.1 (-79.3 to -48.6)
		YLLs	92.5 (46.1 to 152.6)	96.5 (47.3 to 164.4)	89.8 (40.6 to 151.6)	29.7 (14.9 to 48.2)	33.3 (16.1 to 55.7)	27.6 (12 to 49)	-67.9 (-78.2 to -52.5)	-65.5 (-78.2 to -44.6)	-69.3 (-81.1 to -50.4)
		YLDs	9.1 (3.4 to 16.2)	11.3 (4.6 to 19.6)	7.8 (2.7 to 14.3)	5.5 (2 to 9.9)	7.1 (2.7 to 13.4)	4.6 (1.5 to 8.5)	-40.2 (-50.7 to -28.8)	-36.8 (-50.6 to -22.5)	-41.7 (-56.4 to -25.5)
	Diet low in vegetables	Deaths	2.9 (0.8 to 5.8)	2.6 (0.6 to 5.8)	3.2 (1 to 6)	1.4 (0.4 to 2.7)	1.3 (0.3 to 2.7)	1.6 (0.4 to 3)	-49.5 (-68.5 to -19.6)	-49.7 (-70.2 to -9)	-49.6 (-70 to -21.3)
		DALYs	62.3 (15.8 to 132.9)	57.2 (12.1 to 138.3)	67.2 (18.4 to 130.4)	28.6 (6.9 to 55.6)	27.4 (6.1 to 57.5)	30.1 (6.8 to 56.9)	-54 (-70.6 to -28.1)	-52.1 (-70.7 to -15.3)	-55.2 (-73.1 to -30.5)
		YLLs	56.7 (14.2 to 123.7)	51.2 (10.3 to 129.2)	62 (16.9 to 123.5)	24.6 (6.2 to 47.7)	22.9 (5.2 to 48.7)	26.3 (6.1 to 49.2)	-56.7 (-72.4 to -29.7)	-55.3 (-73 to -16.7)	-57.5 (-74.5 to -32)
		YLDs	5.5 (1.2 to 10.7)	6 (1.4 to 12.2)	5.2 (1.2 to 10.1)	4.1 (0.8 to 8.1)	4.5 (0.8 to 9.3)	3.8 (0.7 to 7.5)	-26.6 (-45.7 to -10.7)	-24.9 (-47.4 to -3.1)	-27.3 (-46.7 to -12.9)
	Diet low in whole grains	Deaths	4 (1.2 to 6.3)	2.6 (0.8 to 4.3)	5.6 (1.6 to 9)	3.1 (0.8 to 4.8)	2.4 (0.6 to 3.8)	3.9 (1 to 6.1)	-21.5 (-42.5 to 6.5)	-7.6 (-38.3 to 29.9)	-30.8 (-50.8 to -2.7)
		DALYs	88.4 (25.1 to 138.7)	57.7 (16.1 to 91.7)	116.8 (32.8 to 188)	61.5 (15.1 to 93)	50 (12.1 to 77)	71.9 (17.5 to 110.7)	-30.4 (-47.7 to -7.8)	-13.3 (-38.6 to 12.7)	-38.4 (-55.8 to -15.1)
		YLLs	75.3 (21 to 119.7)	44.4 (12.1 to 72.7)	103.8 (29.2 to 171.3)	50 (12.5 to 76.5)	37.3 (9 to 58.7)	61 (15 to 96)	-33.6 (-52.2 to -7.7)	-16.1 (-44.3 to 19)	-41.2 (-59.1 to -14.9)
		YLDs	13 (3.3 to 20.8)	13.3 (3.4 to 21.3)	12.9 (3.3 to 21)	11.5 (2.9 to 18.8)	12.8 (3.1 to 21)	10.8 (2.7 to 17.7)	-11.5 (-25.4 to -4.2)	-4.2 (-20.9 to 6.9)	-16.1 (-29.8 to -7.8)
	High body-mass index	Deaths	19.8 (10.3 to 32)	23.5 (13 to 37)	17 (7.3 to 29.6)	22.4 (14.1 to 31.8)	24.4 (15.7 to 34.3)	21 (12 to 30.9)	13.1 (-25.9 to 84.3)	4.1 (-33.8 to 65.9)	23.6 (-25.2 to 141.7)
		DALYs	603.9 (336.6 to 939)	737.4 (442.4 to 1122.6)	511.1 (241.6 to 854.6)	595.5 (401.5 to 804.2)	673.2 (468 to 893.9)	545.1 (343.1 to 786)	-1.4 (-31.5 to 55.7)	-8.7 (-38.1 to 37.4)	6.6 (-32.6 to 97.6)
		YLLs	547.2 (298.6 to 868.5)	657.3 (378.9 to 1019.4)	469.2 (217.9 to 794.3)	502.2 (331.6 to 686.7)	556.4 (376.9 to 750.1)	465.7 (289.3 to 678.7)	-8.2 (-38.1 to 47.8)	-15.3 (-44.6 to 32)	-0.7 (-39.3 to 90.2)
		YLDs	56.8 (32.4 to 87.4)	80.1 (47.8 to 121.1)	41.9 (20.4 to 69.9)	93.3 (61.2 to 130.1)	116.8 (78.1 to 160.9)	79.4 (50.1 to 112.6)	64.3 (28 to 130.6)	45.7 (17.4 to 87.3)	89.4 (41.6 to 230.6)
	High fasting plasma glucose	Deaths	25.9 (15.4 to 43.8)	24.4 (13.2 to 42.8)	28 (16.4 to 49.1)	33.2 (18.5 to 61.5)	30.2 (17.2 to 52.5)	37 (19 to 73.3)	28.5 (-16.6 to 95.7)	23.6 (-27.4 to 109.3)	32.1 (-16.2 to 104.9)
		DALYs	519.1 (324.9 to 832)	492.2 (280.9 to 834.6)	546.6 (336.4 to 888.8)	600.6 (370.3 to 978.1)	566.5 (345.4 to 900.7)	638.3 (362.4 to 1098.8)	15.7 (-20.5 to 73.4)	15.1 (-28.8 to 87.5)	16.8 (-23.1 to 81.9)
		YLLs	475.4 (296.2 to 768.8)	444.9 (248.5 to 742)	506.7 (309 to 835.7)	528.7 (327.2 to 867.5)	490 (299.7 to 771.7)	570.5 (321 to 1001.3)	11.2 (-24.8 to 71.3)	10.1 (-33.3 to 86.3)	12.6 (-27.7 to 81)
		YLDs	43.7 (24.6 to 71)	47.3 (25.9 to 80)	39.9 (22.8 to 67.5)	71.9 (39.1 to 118.3)	76.5 (41.8 to 127.2)	67.8 (34.9 to 114.7)	64.5 (30.2 to 107.8)	61.7 (17.3 to 122.6)	69.7 (29 to 117.3)
	High LDL cholesterol	Deaths	15.8 (5.9 to 33.8)	11 (3.5 to 25.2)	21.7 (7.9 to 44.9)	16.1 (5 to 36)	13.1 (3.7 to 29.8)	19.3 (5.9 to 43.8)	1.9 (-30.5 to 40)	19.4 (-20.5 to 71.3)	-11 (-41.9 to 26.5)
		DALYs	343.7 (182.9 to 619)	233.8 (115.5 to 443.7)	443.8 (228.1 to 782.4)	302.4 (146.3 to 570.7)	255.6 (118.8 to 472.2)	343.7 (163.1 to 649.1)	-12 (-36.5 to 17.2)	9.3 (-18.8 to 44.1)	-22.6 (-46.7 to 10.4)
		YLLs	291.1 (144.1 to 535.9)	178.2 (77.3 to 368.2)	393.4 (189.5 to 712.4)	245.5 (109.2 to 482)	190.4 (75.5 to 387.9)	292 (127.2 to 579.5)	-15.7 (-43.5 to 18.7)	6.9 (-30.3 to 55.2)	-25.8 (-52.4 to 11.5)
		YLDs	52.6 (30.4 to 86.9)	55.6 (32.5 to 90.4)	50.4 (29.1 to 83.6)	57 (33.5 to 94)	65.1 (38.3 to 105.7)	51.8 (29.9 to 86.4)	8.3 (-0.5 to 17.5)	17.1 (5 to 30.3)	2.7 (-8.5 to 13.2)
	High systolic blood pressure	Deaths	54.7 (37.1 to 76.5)	51.3 (32.7 to 76.8)	58.7 (38.5 to 86.4)	50.9 (38.8 to 64.5)	51.2 (37.6 to 65.3)	51.4 (38.2 to 68.4)	-6.9 (-33 to 32.5)	-0.1 (-33.2 to 59.3)	-12.5 (-40.2 to 31.2)
		DALYs	1205.7 (849.2 to 1659.2)	1137.5 (742.6 to 1633.4)	1271.2 (843.5 to 1787.1)	986.4 (779.1 to 1209.1)	1031.9 (806.3 to 1289.8)	959.8 (734.5 to 1215.7)	-18.2 (-38.9 to 14)	-9.3 (-36.5 to 37.8)	-24.5 (-47.6 to 9.8)
		YLLs	1102.8 (761.5 to 1564.3)	1025.7 (654.6 to 1510.5)	1175.6 (766.7 to 1685.2)	856.2 (671.1 to 1057.5)	879.5 (670.6 to 1112.4)	846 (637.6 to 1096.9)	-22.4 (-42.9 to 11.9)	-14.3 (-41.9 to 35.7)	-28 (-51.2 to 8.2)
		YLDs	102.9 (68.5 to 138.7)	111.8 (70.6 to 154)	95.6 (63.9 to 131.3)	130.2 (92 to 170.7)	152.4 (107.2 to 201.1)	113.8 (78.3 to 151.4)	26.6 (10 to 49.6)	36.3 (9.1 to 74.2)	19.1 (-1 to 46.3)
	High temperature	Deaths	8.3 (-4.5 to 22.4)	7.6 (-3.9 to 19.9)	9.4 (-5.3 to 27.6)	6.1 (1.9 to 13.3)	5.6 (1.7 to 12.6)	6.7 (2.1 to 14.8)	-26.9 (-77.1 to 2.9)	-25.7 (-77 to 17)	-29.2 (-78.2 to 12.7)
		YLLs	160.6 (-84.4 to 440.4)	148.1 (-74.2 to 385.6)	175.3 (-95.9 to 500.2)	96.5 (29.4 to 212.3)	92.4 (27.3 to 210.8)	102.1 (32.6 to 226.7)	-39.9 (-81.8 to -13.4)	-37.6 (-80.3 to 0.9)	-41.7 (-82.4 to -9.1)
	Household air pollution from solid fuels	Deaths	21.2 (11.4 to 33.4)	21.6 (12 to 33.8)	21 (10.5 to 36)	0.1 (0 to 0.3)	0.1 (0 to 0.3)	0.1 (0 to 0.2)	-99.5 (-99.9 to -98.6)	-99.5 (-99.8 to -98.5)	-99.6 (-99.9 to -98.8)
		DALYs	477.6 (258.5 to 748.3)	503.9 (285.1 to 773)	461.7 (229.2 to 775.7)	2 (0.5 to 5.3)	2.5 (0.7 to 6.3)	1.7 (0.4 to 4.6)	-99.6 (-99.9 to -98.8)	-99.5 (-99.9 to -98.6)	-99.6 (-99.9 to -99)
		YLLs	433.9 (230.6 to 692.4)	449.9 (249.9 to 705.7)	425.4 (207.4 to 717.1)	1.8 (0.4 to 4.5)	2.1 (0.6 to 5.4)	1.5 (0.4 to 4.1)	-99.6 (-99.9 to -98.8)	-99.5 (-99.9 to -98.7)	-99.7 (-99.9 to -99)
		YLDs	43.7 (23.6 to 68.1)	53.9 (30.3 to 81.9)	36.2 (17.7 to 59.5)	0.3 (0.1 to 0.8)	0.4 (0.1 to 1.1)	0.2 (0.1 to 0.6)	-99.3 (-99.8 to -98.3)	-99.2 (-99.8 to -98)	-99.4 (-99.8 to -98.4)
	Kidney dysfunction	Deaths	11.2 (7.8 to 15.2)	10.8 (7.4 to 15.2)	11.7 (7.7 to 16.5)	11.8 (7.6 to 16.2)	11.3 (7.3 to 15.7)	12.4 (7.3 to 17.4)	5.5 (-23.6 to 46.4)	5 (-28.5 to 57.4)	6 (-27.6 to 54)
		DALYs	233.4 (172 to 309.9)	230.7 (164.7 to 309.2)	238.6 (167.3 to 329.6)	224 (163.8 to 287.1)	225.5 (164.9 to 292.3)	225.9 (158.5 to 296.3)	-4 (-27.2 to 30.1)	-2.3 (-28.7 to 38.1)	-5.3 (-31.6 to 34.8)
		YLLs	212.1 (151.4 to 283.2)	205.9 (143 to 282.6)	220.1 (150.7 to 310)	195.1 (138.9 to 248.8)	191.6 (136.4 to 250.2)	200.5 (137.3 to 265.9)	-8 (-32.1 to 29.6)	-6.9 (-35.2 to 37.7)	-8.9 (-36.7 to 33.7)
		YLDs	21.3 (14.3 to 29)	24.8 (16.6 to 33.7)	18.5 (12.5 to 25.6)	28.9 (19.4 to 39.4)	33.8 (23.1 to 46.5)	25.4 (17.1 to 34.7)	35.8 (22.8 to 49.2)	36.5 (22.3 to 51.2)	37.3 (21.8 to 54.1)
	Lead exposure	Deaths	5.9 (2.9 to 9.4)	4.8 (2.1 to 8.1)	7.1 (3.7 to 11.2)	3.4 (1.6 to 5.4)	2.8 (1.2 to 4.7)	4.1 (2 to 6.7)	-42.1 (-58.8 to -20.6)	-41.9 (-61.9 to -13.3)	-42.5 (-60 to -17.7)
		DALYs	129.2 (64.4 to 206.9)	106.2 (45.3 to 180.2)	151 (77.9 to 240.9)	57.7 (25.6 to 94)	49 (18 to 85.2)	66.3 (31.3 to 108.1)	-55.4 (-69.1 to -39.8)	-53.8 (-69.9 to -32.7)	-56.1 (-70 to -37.8)
		YLLs	118.4 (59.1 to 192.2)	95.9 (39.5 to 165.8)	139.8 (70.6 to 225.9)	51.2 (23.1 to 83.6)	42.8 (15.9 to 74.6)	59.6 (28 to 97.3)	-56.8 (-70.7 to -40.2)	-55.4 (-71.7 to -32.8)	-57.3 (-71.7 to -37.3)
		YLDs	10.8 (5 to 17.7)	10.3 (4.1 to 17.9)	11.2 (5.5 to 17.9)	6.5 (2.5 to 11.8)	6.3 (1.9 to 12)	6.7 (2.8 to 11.8)	-39.5 (-54.3 to -29.4)	-39.2 (-58 to -27.7)	-40.1 (-54.7 to -29.9)
	Low physical activity	Deaths	6.7 (1.4 to 15)	4.9 (1.1 to 10.5)	9.6 (1.8 to 21.7)	7.7 (1.7 to 15.3)	6.2 (1.6 to 12.4)	9.6 (1.9 to 19.6)	15.1 (-14.7 to 71.7)	26.9 (-10.9 to 98.1)	-0.3 (-29.4 to 57.8)
		DALYs	108 (20.8 to 263.6)	77.5 (17 to 180.2)	145 (25.1 to 359.3)	113.3 (24.8 to 235.8)	94.4 (22.2 to 198.2)	133.2 (24 to 280.2)	4.9 (-21.8 to 54.9)	21.9 (-10.7 to 81.6)	-8.1 (-33 to 46.5)
		YLLs	95.1 (18.2 to 231.6)	64.1 (14 to 147.1)	132.4 (23 to 327.5)	97.9 (21.4 to 202.1)	77.3 (18.8 to 161.1)	119.2 (22.1 to 252.1)	3 (-25.4 to 58)	20.5 (-16.2 to 91.4)	-10 (-36 to 46)

Location	Risk factor	Measure	1990			2019			% Change (1990 to 2019)		
			Both	Female	Male	Both	Female	Male	Both	Female	Male
		YLDs	12.9 (2.3 to 32.3)	13.3 (2.6 to 32.6)	12.5 (2.1 to 32)	15.4 (2.9 to 36.2)	17.1 (3.5 to 39.2)	14 (2.3 to 33.8)	18.8 (0.2 to 49.9)	28.4 (9 to 65.5)	11.5 (-7.6 to 46)
	Low temperature	Deaths	4.7 (-7 to 15.8)	4.2 (-6.5 to 14.2)	5.3 (-7.6 to 17.9)	3.3 (-4.7 to 10.5)	3.1 (-4.5 to 9.9)	3.6 (-5.2 to 11.5)	-28.7 (-172.9 to 518.4)	-27.2 (-178.7 to 539.6)	-31.1 (-164.8 to 483.5)
		YLLs	89.9 (-132.9 to 300.7)	82.6 (-128.7 to 278.4)	98.5 (-141.7 to 330.7)	52.6 (-73.8 to 163.7)	50.5 (-72.9 to 157.7)	55.7 (-80.1 to 173.7)	-41.4 (-160.3 to 426.2)	-38.9 (-166.3 to 446.5)	-43.4 (-157.3 to 381.3)
	Secondhand smoke	Deaths	3.8 (2.5 to 5.4)	3.8 (2.4 to 5.4)	4 (2.6 to 5.9)	2.4 (1.7 to 3.3)	2.3 (1.6 to 3.1)	2.6 (1.8 to 3.7)	-36.4 (-53 to -10.9)	-38.3 (-57.3 to -8.9)	-35.5 (-56.4 to -2.9)
		DALYs	86.9 (56.8 to 125.3)	92.1 (58.6 to 132.4)	86.4 (56 to 127.8)	48.3 (34.6 to 64.6)	51.6 (36.6 to 69.1)	48 (33.1 to 67.3)	-44.4 (-58.6 to -23.2)	-44 (-60.2 to -19.8)	-44.5 (-62 to -18.3)
		YLLs	82.1 (53.2 to 119.8)	85.9 (54.2 to 125)	82.5 (52.9 to 122.9)	44 (31.1 to 59.1)	46 (32.2 to 61.8)	44.4 (30.2 to 62.7)	-46.4 (-61.1 to -24)	-46.5 (-62.6 to -20.7)	-46.1 (-63.6 to -19.2)
		YLDs	4.8 (3.1 to 6.7)	6.2 (3.9 to 8.6)	4 (2.6 to 5.7)	4.3 (2.7 to 6)	5.6 (3.6 to 7.8)	3.6 (2.3 to 5.2)	-11 (-20.4 to -0.2)	-9.5 (-20.6 to 3)	-10 (-25.6 to 9.1)
	Smoking	Deaths	13.3 (9.6 to 17.8)	3.8 (2.3 to 5.9)	22.4 (15.8 to 29.7)	5.4 (4.4 to 6.6)	1.7 (1.2 to 2.5)	8.7 (6.8 to 11)	-59.5 (-71.5 to -41.6)	-54.9 (-73.8 to -24.3)	-61.4 (-73.4 to -42.8)
		DALYs	355.5 (258.6 to 471.4)	95.5 (59.7 to 143)	568.4 (405.4 to 754.2)	129.7 (106.9 to 160.6)	41.6 (29.7 to 59.5)	198.2 (160 to 247.5)	-63.5 (-73.8 to -47.9)	-56.5 (-73.7 to -27.3)	-65.1 (-75.4 to -49.5)
		YLLs	325.1 (231.4 to 440.5)	85.8 (52.3 to 129.9)	522.2 (360.4 to 703.8)	110.9 (88.9 to 140)	34.9 (24.5 to 50.2)	171 (133.8 to 218.7)	-65.9 (-76.2 to -49.7)	-59.3 (-76.3 to -29.6)	-67.3 (-77.6 to -50.9)
		YLDs	30.5 (21.6 to 40.1)	9.8 (6.1 to 14.3)	46.2 (32.8 to 60.7)	18.9 (13.3 to 24.9)	6.7 (4.3 to 9.8)	27.2 (19 to 35.5)	-38 (-46.3 to -28.4)	-31.8 (-54.9 to 3.5)	-41.2 (-49.4 to -32.6)
Palestine	All risk factors	Deaths	141.7 (114.1 to 173)	136.6 (107.6 to 169.7)	148.8 (118.1 to 180.5)	101.7 (86.2 to 119.4)	98.7 (83.3 to 117)	107.8 (88.3 to 126.6)	-28.3 (-43.1 to -8.1)	-27.7 (-43.8 to -7.6)	-27.6 (-43.3 to -7.1)
		DALYs	2660 (2165 to 3234.6)	2579.6 (2090 to 3165.8)	2765.3 (2182.1 to 3370.1)	1825.8 (1582.8 to 2103.3)	1796.4 (1551.6 to 2071.5)	1868.2 (1566.8 to 2178.6)	-31.4 (-45.6 to -13.5)	-30.4 (-44.9 to -12.4)	-32.4 (-47.4 to -12.4)
		YLLs	2450.8 (1968.7 to 3025.9)	2334.4 (1850.1 to 2916.2)	2598.9 (2024.9 to 3207.3)	1623 (1387.1 to 1881.2)	1555.9 (1317.6 to 1820.3)	1707.5 (1414.3 to 2004.8)	-33.8 (-48.8 to -14.2)	-33.4 (-48.7 to -13.6)	-34.3 (-49.6 to -13.2)
		YLDs	209.2 (153.4 to 266.7)	245.1 (177.4 to 311.8)	166.4 (121.1 to 214.1)	202.8 (146.8 to 256.3)	240.5 (172.6 to 306.1)	160.7 (115.9 to 204.9)	-3.1 (-9.3 to 3.8)	-1.9 (-9.9 to 7.1)	-3.4 (-12.3 to 5.5)
	Alcohol use	Deaths	1 (0.4 to 1.7)	0.3 (0 to 0.7)	2 (0.8 to 3.2)	0.9 (0.5 to 1.3)	0.3 (0 to 0.6)	1.7 (1 to 2.5)	-12.9 (-51.1 to 112.3)	7.1 (-450.9 to 701.8)	-16.2 (-49.9 to 81.4)
		DALYs	26.3 (11.8 to 41.1)	6.8 (0 to 14.9)	49.9 (23.4 to 78.6)	22.8 (14.3 to 32.3)	6.8 (1.7 to 12.5)	39.9 (24.9 to 57.6)	-13.5 (-48.6 to 88.6)	-1.1 (-90.2 to 350.7)	-20 (-50.1 to 64.2)
		YLLs	25 (11.2 to 39)	6.5 (0.2 to 14.1)	47.5 (22 to 74.7)	20.8 (13.1 to 29.8)	6.1 (1.6 to 11.2)	36.6 (23 to 53.3)	-16.9 (-51.1 to 82.4)	-6.4 (-86.7 to 374.5)	-22.9 (-52.6 to 60.4)
		YLDs	1.3 (0.4 to 2.2)	0.3 (-0.2 to 1)	2.5 (1 to 4.1)	2 (1.1 to 3.1)	0.7 (0 to 1.5)	3.3 (1.8 to 5.2)	50.9 (-5.2 to 288.9)	98.3 (-1453.2 to 1469.8)	34.3 (-7.3 to 152.3)
	Ambient particulate matter pollution	Deaths	18.1 (8.8 to 29.8)	15.8 (7.1 to 26.7)	20.9 (10.3 to 33.8)	23.6 (17.2 to 30.1)	23.1 (16.8 to 29.8)	24.5 (17.4 to 31.3)	30.7 (-16.6 to 145.4)	46.2 (-7.8 to 184.8)	16.8 (-23.5 to 110)
		DALYs	374.2 (179.1 to 615.7)	330.2 (146 to 557.1)	428.2 (213.2 to 692.4)	477.9 (349 to 603.3)	473.4 (346.1 to 598.3)	482.4 (347.3 to 610.4)	27.7 (-18.1 to 142.3)	43.4 (-8.9 to 180.7)	12.6 (-26.6 to 108.7)
		YLLs	342.9 (164.5 to 570.1)	296 (131.1 to 503.6)	400.3 (198.4 to 650.8)	419.2 (305.6 to 532.1)	403.1 (293 to 512.8)	436.3 (311.5 to 558.5)	22.3 (-22.8 to 131.4)	36.1 (-15.8 to 167.8)	9 (-29.9 to 103.4)
		YLDs	31.3 (15 to 52.8)	34.2 (15.6 to 59.5)	27.9 (13.6 to 46.6)	58.7 (38.1 to 79.7)	70.4 (45.9 to 96.5)	46 (29.9 to 63.1)	87.3 (28 to 256.9)	105.8 (35.8 to 296.7)	64.8 (15.2 to 196.3)
	Diet high in red meat	Deaths	3.4 (1.2 to 5.8)	3.3 (1.1 to 5.6)	3.6 (1.3 to 6.2)	2 (0.7 to 3.4)	2 (0.6 to 3.3)	2.2 (0.7 to 3.7)	-40.5 (-58.4 to -19.9)	-40.2 (-59 to -17.1)	-40.1 (-59.2 to -15.3)
		DALYs	77.9 (25.9 to 131.7)	75.6 (25.2 to 126.9)	81 (27.5 to 137.1)	44.2 (14.1 to 74.3)	43.3 (13.7 to 73.1)	45.4 (14.5 to 76.3)	-43.2 (-60.5 to -24)	-42.8 (-60.1 to -23.1)	-43.9 (-61.5 to -21.2)
		YLLs	70.5 (23.1 to 119.4)	66.9 (21.7 to 114.4)	75.1 (24.6 to 128.5)	38.2 (12.4 to 64.1)	36 (11.9 to 61.1)	40.6 (13 to 68.7)	-45.8 (-62.9 to -25.2)	-46.1 (-63.1 to -25)	-45.9 (-63.6 to -21.8)
		YLDs	7.4 (2 to 12.9)	8.8 (2.4 to 15.3)	5.8 (1.6 to 10.2)	6 (1.6 to 10.7)	7.3 (2 to 13.1)	4.8 (1.2 to 8.5)	-18.4 (-29 to -9.7)	-17.1 (-30.2 to -5.7)	-18.1 (-31.4 to -6)
	Diet high in sodium	Deaths	2.7 (0.4 to 10.6)	1.9 (0.4 to 7.5)	3.6 (0.4 to 15)	1.8 (0.3 to 6.9)	1.3 (0.3 to 5)	2.4 (0.3 to 9.8)	-33.9 (-60.5 to 24.7)	-33.4 (-69.2 to 54.6)	-32.9 (-62.3 to 48.8)
		DALYs	56.7 (7.7 to 228.6)	39.8 (7.7 to 162.2)	77.1 (6.3 to 310.8)	37.1 (5.5 to 149.7)	25.9 (5.5 to 108.8)	49.7 (4.6 to 194.4)	-34.6 (-62 to 19.6)	-34.9 (-70.8 to 52.3)	-35.5 (-62.6 to 35.3)
		YLLs	52.3 (7 to 211)	35.9 (7 to 144.5)	72.2 (5.9 to 292.2)	32.9 (4.9 to 132.6)	22.2 (4.7 to 93.9)	45.1 (4.2 to 177.1)	-37.1 (-63.5 to 16.4)	-38 (-72.8 to 48.3)	-37.6 (-64.6 to 33.4)
		YLDs	4.3 (0.6 to 17.5)	3.9 (0.7 to 15.9)	4.9 (0.4 to 20)	4.1 (0.6 to 16.3)	3.6 (0.7 to 15.8)	4.7 (0.4 to 17.7)	-4.8 (-48.8 to 77.2)	-6.4 (-57.5 to 113.5)	-5 (-39 to 85.1)
	Diet low in fiber	Deaths	8.4 (2.3 to 14.7)	8.1 (2.2 to 14.2)	8.8 (2.5 to 15.4)	5.9 (1.7 to 9.8)	5.7 (1.5 to 9.7)	6.2 (1.8 to 10.5)	-29.4 (-45.4 to -7.6)	-28.8 (-47.8 to -0.7)	-29.2 (-47.7 to -2)
		DALYs	173.2 (47.2 to 303.8)	169.1 (44.6 to 297.2)	178.9 (48.4 to 317.1)	118.1 (32.6 to 199.2)	116.6 (30.5 to 199.8)	120.1 (35.6 to 202.1)	-31.8 (-47.5 to -9.6)	-31 (-48.3 to -4.4)	-32.9 (-50.4 to -6.9)
		YLLs	157.1 (41.9 to 279.7)	149.9 (39.9 to 270.4)	166.3 (44.4 to 296.7)	102.4 (28 to 173.6)	97.7 (24.8 to 167.5)	107.8 (31.6 to 183.5)	-34.8 (-51 to -11.7)	-34.8 (-53 to -7.5)	-35.2 (-53.3 to -8.4)
		YLDs	16.1 (4.3 to 28)	19.2 (5 to 33.7)	12.6 (3.4 to 22)	15.7 (4.4 to 27.3)	18.9 (5.1 to 33.2)	12.3 (3.5 to 21.4)	-2.9 (-15.5 to 14.4)	-1.4 (-18 to 21.8)	-3 (-20.2 to 19.9)
	Diet low in fruits	Deaths	8.5 (3.8 to 14.4)	8.3 (3.7 to 14)	8.9 (4 to 15.1)	4.9 (2 to 8.3)	4.8 (1.9 to 8.3)	5.1 (2 to 8.8)	-42.8 (-61.3 to -22.3)	-42.1 (-61.5 to -19.6)	-43 (-62.4 to -21.5)
		DALYs	183.6 (83.5 to 307.5)	180.3 (82.3 to 301.9)	188.3 (84.9 to 318.7)	101.8 (43.5 to 170.4)	101.6 (43.6 to 170.3)	102.3 (42.5 to 177.2)	-44.5 (-62 to -25.1)	-43.6 (-61.1 to -23)	-45.7 (-63.6 to -23.5)
		YLLs	167.9 (77.3 to 284.3)	161.6 (74.6 to 274)	176.2 (81 to 299.7)	88.7 (38.6 to 149.7)	85.6 (37.5 to 144.1)	92.2 (38.2 to 161.2)	-47.2 (-64.1 to -26.7)	-47 (-64.4 to -25)	-47.7 (-65.4 to -24.9)
		YLDs	15.7 (5.8 to 28.7)	18.8 (7.1 to 34.3)	12.1 (4.6 to 22.2)	13.1 (4.7 to 24.7)	16 (5.7 to 30.3)	10.1 (3.5 to 19.4)	-16.5 (-32.2 to -6.3)	-14.6 (-32.2 to -1.3)	-17.1 (-36.7 to -3.1)
	Diet low in vegetables	Deaths	8.2 (3.8 to 12.8)	7.8 (3.5 to 12.3)	8.7 (4 to 13.8)	2.3 (0.8 to 4)	2.2 (0.8 to 3.8)	2.6 (0.9 to 4.5)	-71.7 (-86.1 to -51.7)	-72.1 (-86.9 to -50.9)	-70.2 (-85.1 to -50.1)
		DALYs	175.6 (80 to 273.1)	169 (75.7 to 267.3)	184.2 (84.8 to 292.5)	38.3 (11.1 to 69.8)	36.3 (10.7 to 67.1)	41 (11.9 to 75.7)	-78.2 (-91.2 to -58.8)	-78.5 (-92 to -58.9)	-77.7 (-90.8 to -59.4)
		YLLs	161.7 (72.6 to 252.3)	152.7 (67.3 to 242.1)	173.2 (78.4 to 275.8)	33.9 (10.1 to 62)	31.2 (9.6 to 57.6)	37.4 (11.3 to 69.1)	-79 (-91.5 to -59.2)	-79.5 (-92.2 to -60.4)	-78.4 (-91 to -59.2)
		YLDs	13.9 (5.1 to 23.7)	16.3 (5.9 to 27.5)	11 (4.1 to 19)	4.4 (0.9 to 8.8)	5.1 (1 to 10.5)	3.6 (0.7 to 7)	-68.5 (-89.9 to -41.3)	-68.9 (-90.5 to -40.2)	-67.5 (-89.3 to -40)
	Diet low in whole grains	Deaths	9.4 (5.5 to 13)	9.5 (5.5 to 13)	9.5 (5.3 to 13.3)	7.4 (4.3 to 10.1)	7.5 (4.4 to 10)	7.6 (4.1 to 10.3)	-21.2 (-38.1 to 0.9)	-21.3 (-39.1 to 1.3)	-20.3 (-38.8 to 5)
		DALYs	174.3 (96.8 to 244.7)	177.6 (99.7 to 247.3)	171.1 (89.2 to 244.7)	134 (73.2 to 181.9)	137.2 (77.7 to 184.5)	130.6 (67.3 to 180.5)	-23.1 (-39.4 to -3.2)	-22.8 (-39.4 to -3.5)	-23.6 (-41.1 to -1.6)
		YLLs	153.6 (85.9 to 218)	152.6 (85.1 to 215.4)	155.4 (81.1 to 224.9)	114.9 (63.7 to 157.1)	113.9 (64.7 to 154.1)	116.1 (59.4 to 161.1)	-25.2 (-43 to -2.6)	-25.4 (-43.7 to -2.5)	-25.3 (-44.2 to -0.5)
		YLDs	20.7 (10.7 to 30.1)	25 (13 to 36)	15.7 (8 to 23.1)	19.1 (9.6 to 27.8)	23.3 (12 to 34.2)	14.5 (7.1 to 21.1)	-7.7 (-15.1 to -0.7)	-6.6 (-15.3 to 2.3)	-7.5 (-18 to 2.7)
	High body-mass index	Deaths	24.4 (12.6 to 40.2)	27.8 (15.2 to 44)	20.6 (9.1 to 36.6)	19.8 (11.9 to 29.8)	21.4 (13.3 to 31.1)	17.7 (9.8 to 27.9)	-18.9 (-40 to 14.7)	-22.7 (-43.3 to 10.1)	-13.9 (-38.3 to 33.2)
		DALYs	689.1 (388.5 to 1081.1)	785.4 (469.5 to 1194.2)	576.6 (281.5 to 975)	542.5 (354.3 to 758.9)	586.6 (397 to 804.1)	490.9 (301.3 to 719.9)	-21.3 (-40.5 to 7)	-25.3 (-43.7 to 0.3)	-14.9 (-38.9 to 27.9)

Location	Risk factor	Measure	1990			2019			% Change (1990 to 2019)		
			Both	Female	Male	Both	Female	Male	Both	Female	Male
		YLLs	621.2 (341.4 to 980.1)	695.1 (402.8 to 1074.1)	535.1 (258.8 to 917)	464 (299.4 to 657.4)	484.7 (321 to 678.1)	436.3 (263.4 to 645.7)	-25.3 (-45.1 to 4.3)	-30.3 (-49.3 to -2.5)	-18.5 (-42.8 to 24.8)
		YLDs	67.9 (38.5 to 104.9)	90.3 (53.9 to 133)	41.5 (20.3 to 69.6)	78.5 (50 to 113.4)	101.9 (64.6 to 144.7)	54.7 (32.8 to 82.3)	15.6 (3.5 to 36.4)	12.8 (1.1 to 32.2)	31.8 (9 to 80)
	High fasting plasma glucose	Deaths	42.3 (22.5 to 76.3)	38.1 (20.3 to 72.4)	47.6 (25.5 to 84.3)	41 (22.2 to 78.8)	40 (21 to 78.2)	43.4 (23.4 to 82.5)	-3.1 (-30.8 to 37.4)	4.8 (-29.1 to 62.7)	-8.9 (-37.1 to 33.6)
		DALYs	762.2 (429.5 to 1238)	683.7 (383.9 to 1159.3)	857.7 (479.4 to 1378.5)	733.5 (439.8 to 1237.8)	717.4 (411.7 to 1230.3)	753.3 (438.6 to 1263.8)	-3.8 (-30.7 to 35.6)	4.9 (-28.6 to 57.9)	-12.2 (-37.3 to 30.4)
		YLLs	709.3 (392 to 1143.8)	625.8 (347.2 to 1081.5)	810.6 (450.6 to 1296.3)	658.1 (388.5 to 1123.9)	629.9 (363.5 to 1110.5)	692.1 (405.8 to 1160.6)	-7.2 (-34 to 31.7)	0.6 (-33.9 to 54.4)	-14.6 (-40.3 to 28.2)
		YLDs	52.9 (27.9 to 93.1)	57.9 (30.2 to 106.8)	47.1 (23.3 to 84.1)	75.4 (39.7 to 127.1)	87.5 (46.4 to 148.6)	61.3 (32.5 to 104.6)	42.6 (16.7 to 85.1)	51.3 (16.6 to 112.2)	30.2 (3.5 to 78.6)
	High LDL cholesterol	Deaths	21.8 (7.6 to 48.3)	21.9 (7.7 to 48.4)	21.9 (7.6 to 47.4)	18.7 (6 to 39.9)	18.6 (5.8 to 40.7)	19.2 (6.1 to 40.8)	-14.2 (-34.4 to 9.4)	-15 (-36.1 to 9.6)	-12.5 (-34.4 to 13.8)
		DALYs	422.4 (213.5 to 770.2)	432.2 (221.7 to 792.8)	412.7 (203 to 756.7)	350.1 (174.7 to 627.8)	356 (177.7 to 642.8)	344.2 (171.5 to 609.5)	-17.1 (-34.8 to 4.3)	-17.6 (-36.2 to 3.7)	-16.6 (-36.9 to 8.3)
		YLLs	366.1 (172.4 to 692.5)	363.9 (174.2 to 689.8)	370.7 (172.2 to 691.9)	295.1 (138.7 to 554.1)	288.7 (132.8 to 550.1)	302.5 (142.9 to 556.7)	-19.4 (-39.5 to 5.6)	-20.7 (-42.3 to 4.4)	-18.4 (-40.4 to 9.6)
		YLDs	56.3 (33.6 to 91.7)	68.3 (40.4 to 111.1)	42 (24.7 to 68.9)	55 (32.5 to 90.6)	67.3 (39.4 to 112)	41.7 (24.9 to 68.3)	-2.4 (-10.4 to 5)	-1.4 (-11.2 to 8.5)	-0.8 (-12.5 to 10.7)
	High systolic blood pressure	Deaths	74.6 (54.4 to 99.3)	75.5 (52.9 to 102.5)	73.5 (52.7 to 98.5)	51.9 (38.9 to 67.1)	50 (35.9 to 66.7)	55.5 (40.1 to 72)	-30.4 (-48.5 to -8.6)	-33.8 (-53.2 to -5.5)	-24.6 (-44.6 to 1.7)
		DALYs	1468.7 (1100.4 to 1868.6)	1472.1 (1084.4 to 1917.9)	1466.9 (1065.8 to 1926.5)	979.3 (759.6 to 1210.4)	949.8 (706.6 to 1209.1)	1015.4 (770.5 to 1268.1)	-33.3 (-48.8 to -13.4)	-35.5 (-52.6 to -11.9)	-30.8 (-48.1 to -7.1)
		YLLs	1353.5 (987.7 to 1751)	1335.7 (962.2 to 1767.9)	1377.5 (984.1 to 1828.6)	870.6 (668.4 to 1095.3)	823 (610.7 to 1064.6)	926.8 (699.9 to 1164.7)	-35.7 (-52 to -15)	-38.4 (-56.1 to -13.3)	-32.7 (-50.5 to -7.9)
		YLDs	115.2 (79.3 to 152.5)	136.4 (91.3 to 183.1)	89.5 (61.8 to 119.2)	108.8 (74.9 to 144.8)	126.8 (84.5 to 172.5)	88.6 (60.5 to 120.5)	-5.6 (-18.1 to 9.2)	-7 (-24.7 to 15.6)	-1 (-16 to 17.3)
	High temperature	Deaths	0.5 (-0.5 to 5.4)	0.5 (-0.5 to 5.4)	0.5 (-0.5 to 5.5)	0.6 (-0.3 to 2.9)	0.5 (-0.3 to 2.8)	0.6 (-0.4 to 3.1)	17.7 (-700.7 to 681.5)	19.6 (-671.8 to 665.3)	17.7 (-732.3 to 680.8)
		YLLs	8 (-8.9 to 90)	7.7 (-8.8 to 88.5)	8.4 (-9 to 92)	8.7 (-5.3 to 44.9)	8.4 (-5.2 to 43.2)	9 (-5.4 to 47)	8.1 (-649.3 to 627.1)	9.7 (-623.7 to 617.3)	6.4 (-685.5 to 605.9)
	Household air pollution from solid fuels	Deaths	20.8 (10.5 to 33.1)	22.1 (11.6 to 34.6)	19.3 (8.9 to 31.8)	0.4 (0.2 to 0.8)	0.4 (0.2 to 0.9)	0.3 (0.1 to 0.7)	-98.1 (-99.2 to -96)	-98 (-99.1 to -95.7)	-98.4 (-99.3 to -96.2)
		DALYs	429.4 (217.5 to 674.6)	459.3 (244.8 to 714.7)	394.4 (186.9 to 649.8)	7.8 (3.5 to 15.6)	9.2 (4.3 to 18)	6.1 (2.5 to 13.3)	-98.2 (-99.2 to -96.1)	-98 (-99.1 to -95.8)	-98.4 (-99.4 to -96.3)
		YLLs	391.9 (196.6 to 625.5)	411.8 (216.1 to 644.2)	368.8 (170.8 to 616.1)	6.8 (3 to 13.7)	7.8 (3.6 to 15.3)	5.5 (2.2 to 12.1)	-98.3 (-99.2 to -96.2)	-98.1 (-99.2 to -95.9)	-98.5 (-99.4 to -96.4)
		YLDs	37.5 (19.3 to 59.7)	47.5 (24.8 to 74)	25.7 (12 to 43.3)	1 (0.4 to 2)	1.4 (0.6 to 2.7)	0.6 (0.2 to 1.3)	-97.4 (-98.8 to -94.3)	-97.1 (-98.7 to -93.8)	-97.7 (-99 to -95)
	Kidney dysfunction	Deaths	15.1 (10.1 to 20.5)	15.7 (10.2 to 21.5)	14.4 (9.7 to 19.5)	14 (8.7 to 19.6)	14.2 (8.7 to 20)	14 (8.4 to 19.5)	-7.2 (-28.9 to 19.4)	-9.6 (-32.6 to 17.9)	-3.1 (-29.2 to 29.8)
		DALYs	296.4 (217.1 to 384.4)	313.3 (226 to 411.1)	277 (200 to 364.2)	269.8 (196 to 347)	278.6 (201.8 to 360.1)	260.1 (184.9 to 334.9)	-9 (-29.2 to 14.8)	-11.1 (-31.6 to 14.2)	-6.1 (-28.9 to 23.5)
		YLLs	272 (195.7 to 356.7)	282.7 (201 to 377.2)	260 (185.6 to 346.2)	239.5 (171.6 to 310.4)	241.4 (169.4 to 313.2)	237.5 (166.5 to 309.5)	-11.9 (-32.8 to 13.6)	-14.6 (-36.2 to 13.6)	-8.7 (-31.9 to 22.2)
		YLDs	24.4 (16.7 to 33.1)	30.6 (21.1 to 41.5)	17 (11.5 to 23.2)	30.2 (20.3 to 40.9)	37.1 (24.8 to 50.4)	22.7 (15.4 to 31)	23.9 (13.8 to 35.8)	21.3 (9 to 34.7)	33.1 (17 to 49.5)
	Lead exposure	Deaths	8.5 (4.9 to 12.9)	6.9 (3.6 to 10.9)	10.5 (6.2 to 15.7)	5.1 (2.9 to 7.9)	4.2 (2.1 to 6.8)	6.5 (3.8 to 9.8)	-39.9 (-54.7 to -22.8)	-38.8 (-55.2 to -19.3)	-38 (-53.7 to -17)
		DALYs	159.7 (87.7 to 241.5)	128.6 (65.6 to 206.2)	198.1 (114 to 293.5)	83.3 (43.5 to 130.2)	68.2 (31.2 to 111.6)	102.8 (58.3 to 154.5)	-47.8 (-60.9 to -32.8)	-47 (-62.3 to -30.7)	-48.1 (-62 to -31)
		YLLs	148.5 (81.7 to 226.1)	117.4 (59.7 to 190.1)	186.9 (107.5 to 279.2)	75.6 (40.4 to 117.7)	60.6 (27.8 to 99.2)	95.1 (53.8 to 143.9)	-49.1 (-62.6 to -33.3)	-48.4 (-64.1 to -30.8)	-49.1 (-63.2 to -31.2)
		YLDs	11.2 (5.6 to 18)	11.2 (4.9 to 18.8)	11.2 (6.2 to 17.4)	7.7 (3.4 to 13.2)	7.6 (2.8 to 13.9)	7.7 (3.8 to 12.5)	-31.5 (-44.6 to -22.6)	-31.9 (-50.1 to -19.8)	-31.7 (-44.8 to -21.7)
	Low physical activity	Deaths	9.7 (2.1 to 21.1)	10 (2.4 to 20.9)	9.4 (1.8 to 21)	8.7 (2 to 18.2)	9 (2.2 to 18.3)	8.5 (1.5 to 18.7)	-10.3 (-29.2 to 16.4)	-10.3 (-31 to 20.6)	-9.2 (-30 to 22.6)
		DALYs	140.2 (28.7 to 319.6)	148.3 (32.9 to 324.6)	131.1 (22.4 to 320.6)	122.9 (26.5 to 276.5)	131.5 (30.2 to 282)	113.6 (21 to 264.5)	-12.3 (-30.1 to 12.7)	-11.3 (-31.3 to 17.2)	-13.4 (-33.4 to 15.4)
		YLLs	127.3 (25.8 to 289.5)	132.1 (29.5 to 290.5)	122 (21.2 to 296.1)	109.2 (23.7 to 244.5)	114 (26.6 to 242.4)	104.2 (19.4 to 240.5)	-14.2 (-33.3 to 12.8)	-13.7 (-34.8 to 16.3)	-14.6 (-35.5 to 15.7)
		YLDs	13 (2.4 to 32.5)	16.2 (3.3 to 39.4)	9.1 (1.4 to 24.1)	13.8 (2.6 to 33.4)	17.4 (3.6 to 41.4)	9.4 (1.5 to 24.4)	6.1 (-5.6 to 26.8)	7.9 (-6.1 to 31.5)	3.3 (-12.4 to 25.9)
	Low temperature	Deaths	9.9 (0.8 to 20.8)	9.6 (0.8 to 20.5)	10.4 (0.8 to 21.5)	6.6 (0.6 to 13.3)	6.4 (0.6 to 13)	6.9 (0.6 to 13.9)	-33.9 (-49.8 to -9.1)	-33 (-49.9 to -6)	-33.7 (-50.1 to -10.2)
		YLLs	167 (12.8 to 353.3)	160.9 (12.2 to 343.7)	175 (13.6 to 368)	101.3 (8.8 to 206.4)	98.6 (8.6 to 200.4)	104.9 (9.8 to 214.2)	-39.4 (-54.7 to -16.3)	-38.7 (-54.8 to -13.6)	-40 (-55.4 to -16.6)
	Secondhand smoke	Deaths	6.6 (4.6 to 9.1)	7.5 (5.2 to 10.4)	5.5 (3.8 to 7.8)	4 (2.9 to 5.4)	4.7 (3.4 to 6.3)	3.2 (2.3 to 4.4)	-38.6 (-52.7 to -19.7)	-36.9 (-53 to -17.7)	-41.3 (-56.4 to -21)
		DALYs	146 (102.3 to 203)	172.2 (120.6 to 238.9)	114.9 (77.3 to 162.6)	87 (62.6 to 114.2)	105.7 (77 to 136.5)	66.6 (46 to 89.8)	-40.4 (-54.4 to -22.2)	-38.6 (-53.6 to -19.7)	-42 (-56.7 to -21.2)
		YLLs	137.4 (95 to 192.2)	160 (111 to 223.2)	110.5 (74.1 to 157.3)	79.3 (56.7 to 104.6)	94.3 (68.4 to 123.5)	62.7 (43 to 85.3)	-42.3 (-56.5 to -22.8)	-41 (-56.7 to -20.9)	-43.2 (-58.4 to -22.1)
		YLDs	8.6 (5.6 to 12)	12.3 (8 to 17)	4.4 (2.8 to 6.3)	7.7 (4.9 to 10.6)	11.4 (7.2 to 15.7)	3.9 (2.5 to 5.6)	-11 (-18.1 to -3.4)	-6.9 (-15.5 to 2.4)	-11.4 (-24.5 to 2.2)
	Smoking	Deaths	21 (16.2 to 26.9)	6.2 (4.1 to 9)	39 (30.1 to 49.6)	12.5 (10.2 to 14.9)	3.4 (2.4 to 4.7)	23.7 (19.4 to 27.9)	-40.5 (-55.1 to -18.6)	-44.3 (-65.4 to -9.8)	-39.4 (-54.4 to -18.1)
		DALYs	480.3 (373.3 to 614.3)	141.3 (91.6 to 202.6)	890.1 (691.1 to 1132.7)	292.3 (244.3 to 347.4)	77.7 (55.4 to 105.4)	528.2 (441.5 to 621.2)	-39.2 (-54.6 to -18)	-45 (-65.9 to -11.9)	-40.7 (-55.3 to -19.8)
		YLLs	445.1 (339.4 to 575.7)	126.6 (81.4 to 184.7)	830.3 (635.9 to 1075)	260.7 (214.4 to 312.3)	66 (46.7 to 91)	475.9 (388.9 to 564.9)	-41.4 (-57 to -18.6)	-47.9 (-68.2 to -15.3)	-42.7 (-57.7 to -21)
		YLDs	35.2 (24.9 to 46.6)	14.8 (9.2 to 22)	59.8 (41.8 to 78.7)	31.6 (22.6 to 41.2)	11.7 (7.4 to 16.9)	52.3 (37.5 to 68)	-10.3 (-23.2 to 4.2)	-20.6 (-46.7 to 16.1)	-12.5 (-23.4 to 0.1)
Qatar	All risk factors	Deaths	70.9 (53.4 to 90.6)	74.4 (51.4 to 96.5)	66.5 (50 to 89.5)	44.6 (35.3 to 57.7)	71.4 (55.5 to 90.7)	37.6 (28.4 to 52.1)	-37 (-54.1 to -16.6)	-4.1 (-31.8 to 41)	-43.4 (-60.9 to -19.9)
		DALYs	1405.5 (1146.5 to 1754)	1566 (1203.4 to 1958.8)	1314.9 (1041.7 to 1738.9)	782.5 (640.1 to 988.2)	1145.1 (928.2 to 1401.4)	675.4 (532.9 to 881.2)	-44.3 (-57.4 to -28.6)	-26.9 (-45.2 to -1.5)	-48.6 (-62.2 to -31.1)
		YLLs	1196.5 (933 to 1531.2)	1302.5 (941 to 1692.5)	1137.7 (862 to 1548.6)	623.4 (490.4 to 817.9)	933.9 (735.4 to 1194.4)	532.8 (397.6 to 739.9)	-47.9 (-62.1 to -29.6)	-28.3 (-49.5 to 3.5)	-53.2 (-67.7 to -33.5)
		YLDs	209 (152.5 to 262.4)	263.5 (193.7 to 332.9)	177.2 (128.1 to 223.9)	159.1 (116.3 to 201.1)	211.1 (154 to 268.4)	142.7 (103.3 to 181.4)	-23.9 (-28.3 to -19.4)	-19.9 (-25.6 to -14)	-19.5 (-25.2 to -13.1)
	Alcohol use	Deaths	0.2 (0 to 0.5)	-0.1 (-0.3 to 0.1)	0.5 (0.1 to 0.9)	0 (-0.1 to 0.2)	-0.2 (-0.3 to 0)	0.1 (-0.1 to 0.3)	-83.9 (-269.6 to 11)	45.8 (-388.3 to 614.9)	-79.7 (-142.3 to -47.6)
		DALYs	9.7 (2.4 to 18.5)	-1.5 (-5.3 to 2.7)	15.3 (4.5 to 28.1)	2.5 (-0.8 to 6.3)	-2.5 (-4.9 to 0)	4 (-0.3 to 9)	-74.3 (-116.5 to -43.5)	71.2 (-1117.2 to 1156.5)	-73.9 (-103 to -49.5)
		YLLs	8.8 (2.5 to 16.5)	-0.7 (-3.9 to 2.9)	13.6 (4.2 to 25.4)	2.1 (-0.4 to 5.2)	-1.8 (-3.9 to 0.1)	3.2 (0.1 to 7.3)	-76.4 (-106.8 to -49.5)	158.1 (-1492.1 to 1525.8)	-76.1 (-97.7 to -53.2)

Location	Risk factor	Measure	1990			2019			% Change (1990 to 2019)		
			Both	Female	Male	Both	Female	Male	Both	Female	Male
	Ambient particulate matter pollution	YLDs	0.9 (-0.2 to 2.1)	-0.8 (-1.5 to 0)	1.7 (0.2 to 3.5)	0.4 (-0.5 to 1.4)	-0.7 (-1.3 to -0.1)	0.7 (-0.4 to 2)	-54.4 (-307.7 to 135.7)	-8.9 (-52.2 to 77.7)	-55.3 (-188.2 to 3.3)
		Deaths	26.4 (19.5 to 34.9)	28.1 (19.4 to 37.2)	25 (18.5 to 34.8)	15.5 (11.9 to 20.7)	24.3 (18.8 to 31.4)	13 (9.6 to 18.4)	-41.5 (-57.6 to -21.2)	-13.4 (-39.3 to 25.3)	-48.1 (-64 to -26.1)
		DALYs	592.4 (466.3 to 758)	676 (521.4 to 864.2)	553.3 (427 to 748.5)	311.4 (248 to 397)	445.9 (353.8 to 559.3)	270 (208.1 to 354.9)	-47.4 (-59.4 to -32.2)	-34 (-50.8 to -11.9)	-51.2 (-63.9 to -34.3)
		YLLs	498.6 (373.5 to 654.1)	555.9 (398.2 to 735.4)	473.3 (350.4 to 671.1)	240.7 (183.4 to 321.2)	350.9 (272.3 to 457.4)	206.9 (152.2 to 289.7)	-51.7 (-64.6 to -34.1)	-36.9 (-55.8 to -8.8)	-56.3 (-70.3 to -37.1)
	Diet high in red meat	YLDs	93.7 (66.8 to 120.9)	120.1 (85.4 to 154)	80.1 (56.2 to 103.2)	70.8 (50.5 to 90.1)	95 (67.5 to 122.7)	63.1 (45 to 81.3)	-24.5 (-29.3 to -19.2)	-20.9 (-27 to -14.5)	-21.2 (-27.3 to -14.6)
		Deaths	3.6 (1.7 to 5.6)	3.9 (1.8 to 6.2)	3.4 (1.5 to 5.5)	2.1 (0.9 to 3.3)	3.2 (1.3 to 5.1)	1.8 (0.8 to 2.9)	-42.3 (-61.4 to -17.5)	-18.8 (-50.6 to 30.4)	-47.3 (-66.9 to -19.7)
		DALYs	92.5 (45.9 to 140.7)	109.9 (52.4 to 166.8)	85.3 (40.6 to 134.9)	48.6 (23.2 to 71.8)	66.9 (30.9 to 100.9)	43.2 (20.5 to 64.7)	-47.4 (-62.3 to -29.3)	-39.1 (-60.1 to -10.9)	-49.4 (-65.2 to -26.1)
		YLLs	76.7 (36.7 to 122.3)	89.3 (39.3 to 141.6)	71.8 (33.9 to 117.8)	36 (17 to 55.1)	49.9 (22.3 to 79.5)	31.8 (14.5 to 50.3)	-53.1 (-67.8 to -33.8)	-44.1 (-65.7 to -11.5)	-55.7 (-71.1 to -31.5)
	Diet high in sodium	YLDs	15.7 (6.8 to 23.9)	20.6 (9 to 32)	13.6 (5.8 to 21.2)	12.7 (5.4 to 19.4)	17 (7.1 to 25.7)	11.4 (4.8 to 17.6)	-19.4 (-32.4 to -1.6)	-17.8 (-36.1 to 5.4)	-16.1 (-33.1 to 9.1)
		Deaths	1.3 (0.2 to 5.4)	1 (0.2 to 4.1)	1.6 (0.2 to 6.7)	0.8 (0.1 to 3.1)	0.8 (0.2 to 2.9)	0.8 (0.1 to 3.1)	-39.3 (-65.5 to 15.8)	-20.3 (-55.6 to 91)	-49.4 (-72.5 to 33.8)
		DALYs	31.4 (3.8 to 127.1)	23.5 (4.5 to 99.8)	36.4 (2.9 to 149.1)	17 (2.2 to 65.9)	14.6 (3.5 to 59.6)	17.7 (1.6 to 68.2)	-46 (-66 to -4)	-37.7 (-65 to 51.9)	-51.5 (-69.9 to 0.4)
		YLLs	26.6 (3.2 to 109.3)	19.4 (3.6 to 84.4)	31.2 (2.5 to 129.3)	13 (1.7 to 51.2)	11.5 (2.7 to 46.3)	13.4 (1.2 to 53.1)	-51.1 (-70.3 to -5.5)	-40.5 (-67.1 to 53)	-56.9 (-75.3 to -4.4)
	Diet low in fiber	YLDs	4.8 (0.5 to 19.4)	4.1 (0.7 to 17.3)	5.2 (0.4 to 20.6)	4 (0.4 to 15.2)	3.1 (0.6 to 12.9)	4.2 (0.3 to 16.5)	-18.1 (-51.2 to 28.3)	-24.5 (-61.6 to 61.3)	-18.6 (-47.3 to 47.9)
		Deaths	0.4 (0.2 to 0.8)	0.4 (0.2 to 0.8)	0.4 (0.2 to 0.8)	0.2 (0.1 to 0.3)	0.2 (0.2 to 0.5)	0.1 (0.1 to 0.3)	-54.2 (-74.4 to -16.1)	-32.5 (-65.4 to 29.7)	-59.6 (-80.9 to -19.4)
		DALYs	7.4 (3.4 to 15.3)	8.1 (3.7 to 17.9)	7.2 (3.1 to 16)	2.8 (1.7 to 5.2)	3.9 (2.5 to 7.3)	2.5 (1.5 to 5.1)	-61.5 (-77 to -33.4)	-51.4 (-75.5 to -12.2)	-64.6 (-81.2 to -34.5)
		YLLs	6.2 (2.8 to 13)	6.6 (3 to 14.9)	6.1 (2.6 to 13.3)	2.2 (1.4 to 4.2)	3.1 (2 to 5.9)	2 (1.1 to 4)	-63.8 (-79.3 to -34.8)	-52.5 (-76.2 to -11.4)	-67.5 (-83.4 to -37.7)
	Diet low in fruits	YLDs	1.2 (0.5 to 2.6)	1.4 (0.6 to 3.4)	1.1 (0.4 to 2.5)	0.6 (0.3 to 1.2)	0.8 (0.4 to 1.7)	0.6 (0.3 to 1.2)	-49.4 (-69 to -21.4)	-46.5 (-72.4 to -2.4)	-48.1 (-71.5 to -10.8)
		Deaths	1.1 (0.5 to 2)	1.4 (0.6 to 2.6)	1 (0.4 to 2)	0.5 (0.2 to 0.9)	0.8 (0.3 to 1.7)	0.4 (0.2 to 0.8)	-60.1 (-80.2 to -23)	-42.3 (-77 to 24.7)	-62.4 (-84.8 to -14.3)
		DALYs	28.9 (13.5 to 50.5)	38.3 (16.3 to 72.3)	24.8 (10.6 to 47.8)	10.8 (5.2 to 19.3)	16.2 (7.2 to 31.2)	9.2 (4 to 17.8)	-62.8 (-78.3 to -36.6)	-57.7 (-79.7 to -19.8)	-63.1 (-82 to -26.8)
		YLLs	24.3 (11.5 to 42.8)	31.7 (13.7 to 60.8)	21.1 (9 to 40.9)	8.1 (3.9 to 14.7)	12.2 (5.3 to 24.5)	6.9 (3 to 13.2)	-66.9 (-81.6 to -40.3)	-61.5 (-82.6 to -21.9)	-67.5 (-85.5 to -30.7)
	Diet low in vegetables	YLDs	4.6 (1.8 to 8.7)	6.5 (2.5 to 12.6)	3.7 (1.4 to 7.6)	2.7 (1 to 5.2)	4 (1.5 to 8.3)	2.3 (0.8 to 4.9)	-41.4 (-64.4 to -9.2)	-39 (-68.4 to 7.3)	-38.3 (-68.8 to 11.8)
		Deaths	0.4 (0.2 to 0.8)	0.4 (0.2 to 0.8)	0.4 (0.2 to 0.8)	0.1 (0.1 to 0.2)	0.2 (0.1 to 0.4)	0.1 (0.1 to 0.2)	-63.1 (-81.6 to -28.6)	-41.9 (-73.8 to 24.6)	-68.7 (-85.5 to -33)
		DALYs	7 (3.2 to 15.2)	7.2 (3.4 to 16.6)	7 (2.9 to 16)	2.2 (1.6 to 3.6)	3.2 (2.3 to 5.5)	2 (1.3 to 3.3)	-68.2 (-83.4 to -40)	-55.9 (-79.2 to -12.6)	-72.1 (-86.8 to -43.5)
		YLLs	6 (2.6 to 13.3)	6 (2.7 to 14.1)	6.1 (2.5 to 13.9)	1.8 (1.2 to 2.9)	2.6 (1.8 to 4.7)	1.6 (1 to 2.7)	-69.9 (-84.8 to -40.7)	-56.2 (-80.9 to -7.2)	-74.2 (-88.2 to -45.4)
	Diet low in whole grains	YLDs	1 (0.4 to 2.2)	1.2 (0.6 to 2.6)	1 (0.4 to 2.2)	0.4 (0.3 to 0.7)	0.5 (0.4 to 0.9)	0.4 (0.2 to 0.7)	-58.9 (-76.3 to -27.8)	-54.3 (-75.5 to -19.7)	-58.7 (-78.7 to -22.2)
		Deaths	2.7 (1 to 4.2)	2.7 (1 to 4.4)	2.7 (1 to 4.4)	1.7 (0.5 to 2.6)	2.6 (0.8 to 4.1)	1.5 (0.5 to 2.4)	-38 (-56.9 to -17.3)	-3.6 (-39.4 to 46.7)	-45.4 (-63.5 to -21.5)
		DALYs	56.2 (19.8 to 85.2)	59.8 (20.6 to 92.4)	54 (19.4 to 86.1)	31.6 (9.3 to 47.8)	45.3 (13.6 to 68.9)	27.6 (8.2 to 42.9)	-43.8 (-58.3 to -30.2)	-24.3 (-46.6 to -0.5)	-49 (-63.6 to -31.8)
		YLLs	42.4 (15.1 to 66.4)	42.4 (14.6 to 68.3)	42.2 (15.4 to 69.9)	21.6 (6.6 to 33.6)	32 (10 to 50.6)	18.6 (5.4 to 31)	-49.1 (-64.4 to -31.1)	-24.3 (-50.8 to 14.6)	-55.9 (-70.9 to -34.4)
	High body-mass index	YLDs	13.8 (4.7 to 21.5)	17.4 (5.5 to 27.6)	11.9 (4.1 to 18.7)	10 (2.8 to 15.7)	13.2 (3.7 to 21.1)	9 (2.6 to 14.3)	-27.7 (-40.3 to -21.6)	-24.1 (-37.7 to -16.2)	-24.6 (-38.4 to -15.5)
		Deaths	18.7 (11.1 to 27.6)	23 (14.4 to 32.8)	16 (8.9 to 25.3)	11.4 (6.6 to 17.4)	17.6 (9.5 to 27.6)	9.5 (5.4 to 14.7)	-38.9 (-57.4 to -13.9)	-23.4 (-52.3 to 16.8)	-40.6 (-62.2 to -8.3)
		DALYs	565.2 (370.1 to 788.8)	748.2 (529.6 to 1001.9)	483.7 (296.6 to 715.1)	324.2 (225.4 to 435.2)	456 (316.5 to 615.6)	281.7 (191.9 to 385.9)	-42.6 (-56.6 to -24.5)	-39 (-55.8 to -17.9)	-41.8 (-58.9 to -18.1)
		YLLs	471.3 (300 to 679.7)	612.3 (411.7 to 841.1)	408.7 (244 to 626.6)	237.7 (157.4 to 332.1)	334.9 (214.8 to 471.6)	205.7 (134.5 to 299.9)	-49.6 (-64.5 to -28.5)	-45.3 (-63.2 to -19.2)	-49.7 (-67.2 to -23.9)
	High fasting plasma glucose	YLDs	93.8 (58 to 132.8)	135.9 (91.1 to 188.2)	75 (43.8 to 109.2)	86.6 (57.6 to 117.7)	121.1 (82.6 to 161.9)	75.9 (50.1 to 105)	-7.7 (-15.6 to 4.2)	-10.9 (-18.6 to -1.9)	1.3 (-10.9 to 20.3)
		Deaths	26.1 (14.6 to 48.4)	26.4 (14.6 to 48.2)	25.4 (13.8 to 47.1)	15.1 (8.2 to 28.9)	22.5 (12.8 to 43)	13.1 (6.9 to 25.8)	-42.3 (-61.4 to -16.6)	-14.9 (-45 to 31.4)	-48.4 (-67.6 to -20)
		DALYs	496.9 (314.4 to 804.9)	519.3 (323.2 to 823.1)	481 (290 to 801.2)	269.3 (169.1 to 439.4)	369.2 (232.9 to 619.3)	239.5 (143.4 to 405.8)	-45.8 (-61.2 to -25.7)	-28.9 (-51.3 to 3.1)	-50.2 (-66.2 to -27.1)
		YLLs	425.2 (263.3 to 693.1)	432.4 (257.8 to 691.2)	419.2 (252 to 700.7)	213.3 (127.1 to 365.6)	299.4 (183.4 to 522.1)	187.9 (107 to 335.1)	-49.8 (-65.9 to -27.8)	-30.8 (-55.1 to 8.2)	-55.2 (-71.4 to -30.9)
	High LDL cholesterol	YLDs	71.7 (40.3 to 115.9)	86.9 (49.4 to 142)	61.8 (34.5 to 100.7)	56 (32.9 to 87.9)	69.9 (42.1 to 111.1)	51.6 (29.9 to 82.2)	-21.9 (-35.1 to -4.8)	-19.6 (-36.8 to 5.3)	-16.5 (-32.9 to 3.1)
		Deaths	9.4 (2.6 to 22)	9.9 (2.7 to 23.8)	8.5 (2.5 to 19.8)	6.6 (1.4 to 16.4)	11.5 (2.3 to 28.5)	5.4 (1.1 to 13.4)	-29.6 (-55.3 to -4.3)	16.2 (-33.5 to 72.3)	-36.3 (-61.8 to -6.9)
		DALYs	188.1 (92 to 346)	212.5 (111.6 to 392.1)	171.3 (84.9 to 308.7)	119.4 (51.4 to 239.5)	187.4 (73.2 to 378.2)	100.2 (44.3 to 198.2)	-36.5 (-53.2 to -19.2)	-11.8 (-40.7 to 21.2)	-41.5 (-58.5 to -21)
		YLLs	140.8 (61.3 to 277.2)	149.3 (63.1 to 302.8)	132.2 (58.1 to 259.6)	81.8 (25.8 to 183.4)	133.8 (36.4 to 305.5)	67.5 (22.3 to 149.7)	-41.9 (-65.7 to -18.5)	-10.4 (-53.3 to 37)	-49 (-69.9 to -22.9)
	High systolic blood pressure	YLDs	47.3 (27.6 to 77.4)	63.2 (38.1 to 101.8)	39.1 (23.1 to 63.7)	37.6 (22.3 to 60.6)	53.6 (32.1 to 86.1)	32.8 (19 to 53.1)	-20.5 (-27.1 to -13)	-15.1 (-22.9 to -6.3)	-16.2 (-25.7 to -5.7)
		Deaths	41 (29.4 to 56.6)	43.8 (28.9 to 60.5)	37.2 (26.2 to 53.5)	25.5 (18 to 36)	41.3 (28 to 58.7)	21.3 (14.4 to 30.9)	-37.8 (-55.4 to -15.9)	-5.6 (-36.6 to 47.9)	-42.7 (-60.9 to -15.7)
		DALYs	838.2 (644.2 to 1099)	929.7 (669.4 to 1221)	774.3 (580.2 to 1076.6)	465 (351.9 to 607)	655 (479.3 to 884.1)	407.6 (299 to 548.3)	-44.5 (-58 to -27.8)	-29.5 (-50.9 to 1.6)	-47.4 (-61.7 to -26.8)
		YLLs	714.4 (527.4 to 970.1)	776.3 (528.7 to 1037.9)	669.2 (478.8 to 961.6)	369.9 (266.8 to 509.2)	541 (380.2 to 749.2)	319.1 (223.2 to 454.5)	-48.2 (-63.3 to -29.4)	-30.3 (-54 to 6.7)	-52.3 (-67.4 to -30.1)
	High temperature	YLDs	123.8 (86.9 to 164.2)	153.4 (106.3 to 207.5)	105.1 (74.3 to 139.1)	95.1 (66.7 to 124.2)	114 (79.7 to 152.7)	88.5 (61.4 to 116)	-23.2 (-31.8 to -13.7)	-25.7 (-38.8 to -8)	-15.9 (-26.8 to -3)
		Deaths	4.7 (0.6 to 9.8)	4.9 (0.6 to 10.5)	4.4 (0.7 to 9.9)	3.3 (1.2 to 6.2)	5.3 (1.9 to 9.3)	2.8 (1 to 5.4)	-29.6 (-65.3 to -2.1)	8 (-49.2 to 67.5)	-36.8 (-69.7 to -8.7)
		YLLs	78.1 (11.1 to 162.7)	84.5 (11.6 to 176.1)	74.7 (11.3 to 168.8)	45 (15.8 to 81.7)	68 (24.3 to 120.1)	38.4 (13.1 to 75)	-42.4 (-72.1 to -20.9)	-19.4 (-63.6 to 21.9)	-48.7 (-75.3 to -25.6)
	Household air pollution from solid fuels	Deaths	0.1 (0 to 0.2)	0.1 (0 to 0.3)	0.1 (0 to 0.2)	0 (0 to 0)	0 (0 to 0)	0 (0 to 0)	-99 (-99.7 to -97.3)	-98.4 (-99.5 to -95.4)	-99.1 (-99.7 to -97.4)
		DALYs	2.3 (0.8 to 5)	3.2 (1.2 to 7)	1.7 (0.6 to 3.8)	0 (0 to 0.1)	0 (0 to 0.1)	0 (0 to 0)	-99.1 (-99.7 to -97.5)	-98.8 (-99.6 to -96.7)	-99.1 (-99.7 to -97.5)

Location	Risk factor	Measure	1990			2019			% Change (1990 to 2019)		
			Both	Female	Male	Both	Female	Male	Both	Female	Male
	[illegible]	YLLs	1.9 (0.7 to 4.2)	2.7 (1 to 5.8)	1.5 (0.5 to 3.3)	0 (0 to 0)	0 (0 to 0.1)	0 (0 to 0)	-99.2 (-99.7 to -97.7)	-98.8 (-99.7 to -96.8)	-99.2 (-99.8 to -97.8)
		YLDs	0.4 (0.1 to 0.8)	0.6 (0.2 to 1.3)	0.2 (0.1 to 0.6)	0 (0 to 0)	0 (0 to 0)	0 (0 to 0)	-98.7 (-99.6 to -96.5)	-98.6 (-99.5 to -96.2)	-98.6 (-99.5 to -96.1)
	Kidney dysfunction	Deaths	6.8 (4.2 to 10)	7.8 (4.5 to 11.9)	5.9 (3.7 to 8.7)	5.4 (2.8 to 8.1)	8.6 (4.2 to 13.3)	4.4 (2.2 to 6.7)	-21.5 (-48.7 to 9.9)	10.2 (-34.7 to 79)	-25.9 (-54.2 to 8.9)
		DALYs	138.3 (98 to 186.4)	171 (114.8 to 235.8)	117.7 (83.2 to 162.5)	100.2 (65.7 to 137.5)	154.5 (98.8 to 213.6)	82.2 (52.8 to 115.5)	-27.5 (-46.5 to -4.4)	-9.6 (-37 to 31.9)	-30.1 (-50.5 to -3.4)
		YLLs	115.1 (80.1 to 159.8)	137.7 (87 to 199.2)	100.5 (68.3 to 144.5)	77.9 (47.8 to 109.9)	122.2 (73.2 to 177.1)	63.3 (37.9 to 93.8)	-32.3 (-54 to -5.5)	-11.3 (-44.6 to 44.2)	-37 (-59.7 to -6.7)
		YLDs	23.2 (15.7 to 31.8)	33.3 (22.6 to 45.4)	17.2 (11.6 to 23.5)	22.3 (15 to 30.1)	32.4 (22.1 to 43.2)	19 (12.6 to 25.8)	-3.7 (-10.7 to 4.4)	-2.9 (-11.5 to 5.8)	10.4 (-0.2 to 22.8)
	Lead exposure	Deaths	1.5 (0.2 to 3)	1.4 (0.1 to 3)	1.5 (0.3 to 3.1)	1 (0.3 to 2)	1.4 (0.2 to 2.8)	0.9 (0.3 to 1.9)	-31.8 (-50.8 to 36.5)	-2.7 (-32.7 to 151.3)	-39.5 (-59.5 to 11.5)
		DALYs	30.9 (4 to 63.6)	29.5 (1.5 to 65.3)	31.8 (5.3 to 63.8)	16.2 (3.3 to 33.3)	19.6 (2.6 to 42.4)	15.3 (3.6 to 31.2)	-47.6 (-61.2 to -3.7)	-33.5 (-52.3 to 100.4)	-52.1 (-66.3 to -19.2)
		YLLs	26.4 (3.4 to 54.7)	24.7 (1.4 to 55)	27.6 (4.6 to 56)	13.3 (2.9 to 27.6)	16.6 (2.4 to 35.2)	12.3 (3 to 25.7)	-49.9 (-64.1 to -4)	-32.7 (-54 to 110.4)	-55.3 (-70.5 to -20.8)
		YLDs	4.5 (0.5 to 9.7)	4.8 (0.2 to 11.3)	4.2 (0.6 to 8.8)	2.9 (0.5 to 6.6)	3 (0.2 to 7.4)	2.9 (0.5 to 6.3)	-34.4 (-44.5 to -4.6)	-37.6 (-54.6 to 28.2)	-31.2 (-44.2 to -4)
	Low physical activity	Deaths	5.4 (1.3 to 11.1)	5.5 (1.4 to 11.2)	5 (1 to 10.4)	4.2 (1.1 to 8.3)	7.1 (1.9 to 13.7)	3.5 (0.8 to 7)	-21 (-43.3 to 12)	29.2 (-11.3 to 103.2)	-28.9 (-51.7 to 7.2)
		DALYs	79.3 (17.6 to 170.6)	85.4 (20.6 to 179.1)	73.1 (14.3 to 162.2)	57.8 (13.5 to 118.3)	93.4 (23.1 to 183.3)	48 (10.3 to 99.8)	-27.1 (-44.5 to -1.1)	9.4 (-19.7 to 61.2)	-34.4 (-52 to -3.7)
		YLLs	64.7 (14.7 to 141)	66.7 (16.2 to 141.7)	61.4 (12.1 to 134.8)	45.8 (11.4 to 91.3)	76.4 (19.6 to 151)	37.6 (8.6 to 77.2)	-29.1 (-49.6 to 1.1)	14.5 (-21.7 to 80.9)	-38.7 (-58.1 to -2.3)
		YLDs	14.7 (2.8 to 34.1)	18.6 (3.8 to 41.9)	11.8 (2 to 29)	12 (2.3 to 27.9)	17 (3.6 to 38.9)	10.4 (1.7 to 24.5)	-18.4 (-28.1 to -9)	-8.6 (-20.2 to 7.3)	-11.7 (-22.5 to 4.9)
	Low temperature	Deaths	3.4 (-4.7 to 11.5)	3.6 (-4.9 to 11.8)	3.2 (-4.3 to 10.5)	2 (-2.6 to 6.2)	3.2 (-4.1 to 10)	1.7 (-2.2 to 5.3)	-	-	-
		YLLs	57.2 (-77.8 to 190.1)	61.7 (-83.9 to 203.4)	54.9 (-73.3 to 178.9)	27.3 (-35.9 to 85.4)	41.3 (-53.8 to 128.4)	23.3 (-30.9 to 74.1)	-	-	-
	Secondhand smoke	Deaths	2.8 (2 to 3.9)	3.1 (2.1 to 4.3)	2.7 (1.8 to 4)	1.4 (0.9 to 1.9)	2.3 (1.6 to 3.2)	1.1 (0.7 to 1.6)	-50.6 (-64.8 to -30.8)	-25.9 (-49.8 to 10.6)	-58.9 (-73.2 to -37.6)
		DALYs	63.3 (44 to 85.8)	82.9 (56.9 to 112.9)	57 (38.4 to 80.6)	27.2 (18.9 to 36.6)	46 (32.5 to 62.3)	21.4 (14.5 to 30.5)	-57.1 (-68.8 to -42.8)	-44.5 (-59.9 to -21.6)	-62.4 (-74.2 to -45.6)
		YLLs	56.9 (39.5 to 78.1)	73.2 (49 to 101.3)	52 (34 to 75.2)	22.8 (15.4 to 32)	38.3 (26.1 to 53)	18.1 (11.8 to 26.7)	-59.9 (-71.5 to -44.4)	-47.7 (-63.9 to -21.8)	-65.1 (-77.4 to -47.1)
		YLDs	6.4 (4.1 to 8.8)	9.7 (6.3 to 13.3)	5.1 (3.3 to 7.2)	4.3 (2.8 to 5.9)	7.7 (5.1 to 10.6)	3.3 (2.2 to 4.6)	-32.5 (-39.5 to -24.5)	-20.5 (-29.1 to -10.3)	-35.2 (-44.5 to -23.8)
	Smoking	Deaths	6.1 (4.5 to 8.6)	1.4 (0.8 to 2.2)	9.7 (7 to 13.7)	3.1 (2.3 to 4.3)	0.7 (0.4 to 1)	3.9 (2.8 to 5.5)	-49.2 (-66.2 to -25.6)	-52.5 (-71.9 to -16.5)	-59.4 (-72.8 to -39.7)
		DALYs	180.8 (138.4 to 243.1)	44.1 (27.9 to 68.2)	257.4 (194.6 to 346.1)	84.9 (65.5 to 108.6)	18.7 (12.8 to 26.9)	106.5 (81.9 to 136.8)	-53 (-66.5 to -35.3)	-57.7 (-75.1 to -29.6)	-58.6 (-70.6 to -42.2)
		YLLs	151.4 (111.3 to 213.1)	35.7 (21.8 to 57.1)	217.8 (157.1 to 307.3)	62.3 (45.2 to 85.4)	13.4 (8.7 to 20)	78.4 (56.7 to 108.5)	-58.9 (-73.1 to -39.2)	-62.6 (-79 to -33.5)	-64 (-76.3 to -45.9)
		YLDs	29.4 (20.8 to 39.3)	8.4 (5.1 to 12.9)	39.7 (28.1 to 52.6)	22.6 (16.1 to 29.9)	5.3 (3.3 to 7.9)	28.1 (20 to 37.2)	-23.1 (-34.1 to -11.1)	-36.7 (-58.7 to -6.7)	-29.1 (-38.8 to -18.2)
Saudi Arabia	All risk factors	Deaths	125.9 (96.8 to 154.3)	127.7 (97.3 to 161.2)	124.6 (90.2 to 154.6)	89.1 (68.5 to 105.4)	88.8 (68.3 to 110.8)	88.5 (63.7 to 104.8)	-29.3 (-47.1 to -5)	-30.5 (-50.8 to -3.5)	-29 (-46 to -3.2)
		DALYs	2548 (2004.1 to 3138.5)	2674.4 (2098.1 to 3331.7)	2463.4 (1815.8 to 3089.6)	1894.5 (1500.9 to 2230)	1935.8 (1552.6 to 2364.6)	1860.8 (1395.2 to 2201.1)	-25.6 (-44 to -0.7)	-27.6 (-47.7 to -1.8)	-24.5 (-43.4 to 4.1)
		YLLs	2316.3 (1771.2 to 2907.7)	2387.4 (1809.9 to 3046.7)	2271.7 (1625.9 to 2901.7)	1633.7 (1262.5 to 1962.6)	1609 (1232.1 to 2030.2)	1644.6 (1180.7 to 1981.9)	-29.5 (-48.8 to -2.2)	-32.6 (-53.2 to -4.3)	-27.6 (-47.2 to 3.2)
		YLDs	231.7 (168.7 to 303)	287 (205.1 to 381.9)	191.7 (138.1 to 249.8)	260.8 (191.6 to 331.2)	326.8 (239.3 to 416.2)	216.3 (156.1 to 277.4)	12.5 (3 to 22)	13.9 (2 to 26.3)	12.8 (3 to 23.5)
	Alcohol use	Deaths	0.6 (-0.1 to 1.4)	0 (-0.3 to 0.4)	1 (-0.1 to 2.3)	0.1 (-0.1 to 0.4)	-0.1 (-0.2 to 0)	0.2 (-0.1 to 0.8)	-82.2 (-223.3 to 59.1)	-1278.8 (-810.6 to 517.8)	-77.6 (-204.2 to 34.9)
		DALYs	17.2 (-1.5 to 41.1)	1.8 (-4.9 to 11.2)	27.7 (-0.8 to 61.9)	4 (-2.6 to 14.2)	-1.2 (-3.7 to 1.8)	7.4 (-2.9 to 22.7)	-76.6 (-183.4 to 70.1)	-165.5 (-692.1 to 504.8)	-73.2 (-171.9 to -0.8)
		YLLs	16.2 (-1 to 38.1)	1.9 (-4.2 to 10.5)	26 (-0.5 to 58.2)	3.8 (-2.1 to 12.9)	-0.8 (-2.8 to 1.9)	6.8 (-2.4 to 20.9)	-76.5 (-169.4 to 68)	-141.5 (-559.7 to 531.8)	-73.7 (-182.5 to -4.5)
		YLDs	1 (-0.4 to 2.8)	-0.1 (-0.8 to 0.8)	1.8 (-0.3 to 4.2)	0.2 (-0.5 to 1.3)	-0.4 (-0.8 to 0.1)	0.6 (-0.6 to 2.2)	-77.7 (-339.2 to 277.2)	175.4 (-1009 to 763.2)	-65.4 (-271.4 to 147.5)
	Ambient particulate matter pollution	Deaths	23.4 (13 to 36.9)	21.6 (11.4 to 34.6)	25 (13.5 to 39.2)	31.3 (23.8 to 38.5)	31.4 (23.7 to 40.5)	31.1 (22.1 to 38.7)	33.5 (-15.5 to 150.3)	45.3 (-14.1 to 184.2)	24.2 (-20.7 to 126.1)
		DALYs	528 (287.5 to 834.8)	502.4 (260.8 to 819.3)	548.9 (297 to 868.2)	755.2 (585.6 to 932.5)	782.4 (604.3 to 981)	736.2 (544.5 to 911.6)	43 (-10.3 to 165.7)	55.7 (-5.8 to 203.4)	34.1 (-16.6 to 151.1)
		YLLs	478.4 (256 to 767.2)	446.3 (232 to 729.6)	503.9 (267.8 to 808.6)	647.3 (484.3 to 811.1)	645.4 (481.2 to 836)	647.5 (456 to 810)	35.3 (-17.5 to 155.3)	44.6 (-16.3 to 186)	28.5 (-21.7 to 144.8)
		YLDs	49.6 (24.4 to 79.7)	56.1 (26.3 to 94.9)	45 (22.9 to 70.5)	108 (77.6 to 138.7)	137 (98.3 to 177.6)	88.8 (62.8 to 114.4)	117.7 (51.3 to 281.4)	144.3 (63.2 to 352.7)	97.1 (39.2 to 242)
	Diet high in red meat	Deaths	4.1 (1.5 to 6.9)	4.3 (1.5 to 7.1)	4.1 (1.4 to 6.8)	2.7 (0.9 to 4.5)	2.7 (0.9 to 4.6)	2.7 (0.9 to 4.4)	-35 (-54.9 to -7.8)	-37.2 (-58.8 to -8)	-33.9 (-53.8 to -6.9)
		DALYs	106 (36.6 to 175.1)	115.2 (39.6 to 187.3)	100.3 (34.5 to 168.8)	73.2 (25.6 to 120.4)	76.6 (26.6 to 125.9)	71 (25.2 to 117.8)	-30.9 (-52.4 to -2.7)	-33.5 (-55.2 to -3.1)	-29.2 (-52.2 to 1.9)
		YLLs	95.2 (31.7 to 159.3)	101.5 (34.7 to 169.4)	91.4 (30.9 to 155.1)	62.3 (21.3 to 104.5)	62.6 (20.3 to 105.9)	62 (21.9 to 105.5)	-34.6 (-56 to -4.1)	-38.3 (-60 to -5.3)	-32.2 (-55.2 to 1.1)
		YLDs	10.8 (3.7 to 18.2)	13.6 (4.6 to 23.1)	8.9 (3 to 14.9)	11 (3.6 to 18.5)	14 (4.5 to 23.4)	9 (3.1 to 15.2)	1.6 (-12.1 to 16)	2.7 (-13.4 to 20.4)	0.8 (-15.7 to 17.6)
	Diet high in sodium	Deaths	2.5 (0.4 to 10.2)	1.8 (0.4 to 7.2)	3.1 (0.3 to 12.7)	1.8 (0.3 to 7.3)	1.1 (0.3 to 4.8)	2.2 (0.2 to 9.1)	-28.9 (-60 to 30.6)	-35.4 (-69.2 to 43.6)	-28.4 (-64 to 53.1)
		DALYs	59.2 (7.3 to 241.4)	42 (7.8 to 168.9)	71.7 (5.4 to 291.3)	44.4 (5.4 to 176)	28.5 (5.7 to 121.1)	54.8 (4.3 to 215.5)	-25.1 (-57.6 to 38)	-32.2 (-67.9 to 60.7)	-23.5 (-56.3 to 60)
		YLLs	53.9 (6.5 to 223)	37.4 (6.9 to 151.7)	65.8 (5 to 267.3)	38.5 (4.6 to 154.5)	23.5 (4.7 to 100.6)	48.3 (3.8 to 190.3)	-28.6 (-61.1 to 35.2)	-37.1 (-70.9 to 50.5)	-26.7 (-58.7 to 58.2)
		YLDs	5.3 (0.6 to 21.2)	4.6 (0.8 to 19)	5.8 (0.4 to 23.4)	5.9 (0.7 to 23.4)	4.9 (0.9 to 21)	6.5 (0.5 to 25.7)	10.9 (-35 to 91.6)	7.7 (-49.5 to 136.5)	12.1 (-29.8 to 112.5)
	Diet low in fiber	Deaths	2.4 (0.6 to 5.2)	2.4 (0.6 to 5.3)	2.4 (0.5 to 5.1)	1.2 (0.3 to 2.7)	1.2 (0.3 to 2.7)	1.2 (0.3 to 2.6)	-49.3 (-65.1 to -25.2)	-50.4 (-69.2 to -20.5)	-49 (-66.6 to -20.5)
		DALYs	50.1 (10.8 to 108.9)	53.2 (11.3 to 120)	48.3 (10.4 to 103.9)	29.8 (6.9 to 66.9)	30.6 (6.9 to 69.9)	29.2 (6.8 to 64.2)	-40.5 (-58 to -14.1)	-42.4 (-60.6 to -14)	-39.4 (-59.2 to -8.7)
		YLLs	44.9 (9.8 to 99.2)	46.8 (10 to 105.9)	43.9 (9.5 to 95.4)	25.2 (5.8 to 57)	24.9 (5.5 to 57)	25.4 (5.9 to 57.1)	-43.9 (-61.8 to -15.7)	-46.8 (-65.7 to -16.2)	-42.2 (-62.1 to -10.8)
		YLDs	5.2 (1 to 11.7)	6.4 (1.3 to 14.5)	4.3 (0.9 to 9.6)	4.6 (1 to 10.5)	5.8 (1.3 to 13.5)	3.8 (0.9 to 8.9)	-11.1 (-27.2 to 8.3)	-9.8 (-30 to 15.9)	-11.7 (-32.9 to 14.1)
	Diet low in [illegible]	Deaths	5.3 (2.5 to 8.7)	5.6 (2.7 to 9.5)	5 (2.4 to 8.3)	4.4 (1.9 to 7.7)	4.5 (2 to 7.9)	4.3 (1.9 to 7.5)	-16.1 (-43 to 20.6)	-19.8 (-48.5 to 20.9)	-13.7 (-41.9 to 27.1)

Location	Risk factor	Measure	1990			2019			% Change (1990 to 2019)		
			Both	Female	Male	Both	Female	Male	Both	Female	Male
	fruits	DALYs	125.9 (60 to 206.2)	142.4 (68 to 242.2)	115.3 (54.8 to 191.6)	116.7 (51.3 to 200.3)	124.4 (55.5 to 214.8)	111.5 (48.7 to 195.1)	-7.3 (-38.1 to 36.2)	-12.6 (-41.4 to 29)	-3.3 (-36.1 to 45.3)
		YLLs	113.9 (53.8 to 188.5)	126.8 (60.4 to 215.7)	105.6 (49.9 to 177)	100 (44.4 to 175.2)	102.8 (46 to 176.8)	98 (43.2 to 172.5)	-12.2 (-43.1 to 31.2)	-18.9 (-48.9 to 24.5)	-7.2 (-40.7 to 43.6)
		YLDs	12 (4.8 to 21.6)	15.6 (6.3 to 28.4)	9.6 (3.9 to 17.3)	16.7 (6.4 to 30.5)	21.6 (8.3 to 39.8)	13.4 (5.1 to 24.3)	38.6 (12.2 to 73)	38.3 (9.9 to 78.8)	39.6 (10.5 to 77.3)
	Diet low in vegetables	Deaths	3.1 (0.9 to 6)	3.1 (0.9 to 6.2)	3.2 (0.9 to 6.1)	2.2 (0.7 to 4.2)	2.2 (0.6 to 4.1)	2.3 (0.7 to 4.2)	-28.5 (-49.6 to 2.1)	-29.8 (-53.7 to 7.6)	-28.2 (-49.5 to 2.8)
		DALYs	65.8 (17 to 138.3)	67.2 (16.4 to 145.6)	65 (16.3 to 137.5)	55.3 (15.9 to 106.5)	55.5 (15.3 to 108)	55.2 (15.4 to 105.7)	-15.9 (-41.9 to 24.6)	-17.5 (-44.3 to 27.8)	-15 (-42 to 28)
		YLLs	59.6 (15.1 to 125.3)	59.8 (14.2 to 132.7)	59.7 (14.9 to 128.9)	47.5 (13.1 to 92.1)	45.8 (12.3 to 91.6)	48.6 (13.7 to 93.7)	-20.3 (-46.5 to 21.9)	-23.5 (-50.9 to 23.6)	-18.6 (-46.1 to 26.1)
		YLDs	6.1 (1.5 to 12.3)	7.4 (1.6 to 15)	5.2 (1.3 to 10.4)	7.8 (2 to 14.8)	9.7 (2.4 to 18.5)	6.6 (1.7 to 12.5)	27.5 (6.2 to 61.6)	30.5 (6 to 73)	26.1 (3.3 to 65)
	Diet low in whole grains	Deaths	4.1 (1.1 to 6.4)	4.2 (1.1 to 6.6)	4 (1 to 6.4)	3.2 (0.8 to 4.9)	3.2 (0.8 to 5)	3.2 (0.8 to 4.9)	-22 (-42.9 to 3.1)	-22.8 (-46.7 to 7.2)	-21.7 (-43 to 4.8)
		DALYs	84.6 (21.7 to 132.5)	89.6 (23 to 140.5)	81.1 (20.4 to 129.1)	73.3 (18.1 to 113.5)	76.7 (19.3 to 120.1)	70.8 (17.4 to 110.5)	-13.4 (-35.1 to 12.4)	-14.4 (-37.4 to 14.3)	-12.6 (-36.7 to 14.5)
		YLLs	72.5 (18.5 to 116.6)	74.2 (19.5 to 121.1)	71.3 (17.3 to 116.7)	59.5 (14.9 to 94.1)	58.9 (14.4 to 94.1)	59.7 (14.8 to 94.3)	-18 (-42.4 to 11.3)	-20.6 (-46.3 to 12.4)	-16.3 (-42.2 to 14.4)
		YLDs	12.1 (3 to 20.1)	15.5 (3.8 to 26.2)	9.7 (2.4 to 16.1)	13.8 (3.4 to 22.3)	17.8 (4.4 to 29.1)	11.1 (2.6 to 18.1)	14.3 (1.3 to 27.2)	15.3 (-1.5 to 32.2)	14.5 (1 to 29)
	High body-mass index	Deaths	29.2 (17.2 to 44.1)	35.5 (21.4 to 52.8)	24.7 (13.3 to 38.8)	30.3 (19.7 to 42)	32.6 (21.2 to 45.6)	28.6 (17.4 to 39.8)	3.8 (-27.5 to 55.8)	-8 (-38.4 to 40.8)	16.1 (-23.5 to 85.7)
		DALYs	871.5 (540.4 to 1259.6)	1093.5 (695.6 to 1584.3)	724.5 (417 to 1090.5)	921.9 (636.9 to 1223.3)	1023 (726.4 to 1345.5)	855.8 (562.5 to 1167.6)	5.8 (-24.5 to 55.6)	-6.4 (-33.1 to 37)	18.1 (-19.2 to 87.4)
		YLLs	783.3 (474.7 to 1161)	968.2 (596.7 to 1430.6)	660.7 (372.5 to 1023.6)	784 (522.3 to 1062.8)	838.4 (567.3 to 1128.4)	748.3 (476.2 to 1028.1)	0.1 (-31.2 to 54.8)	-13.4 (-41.7 to 35.2)	13.3 (-25.9 to 84.3)
		YLDs	88.3 (53.8 to 131.1)	125.3 (77.4 to 181)	63.7 (37.3 to 97.1)	137.9 (90.1 to 190.3)	184.6 (122.8 to 250.2)	107.5 (68.8 to 149.2)	56.2 (34.6 to 90.7)	47.3 (26.7 to 77.3)	68.6 (38.5 to 120.4)
	High fasting plasma glucose	Deaths	36.5 (21.2 to 62.7)	34.8 (19.2 to 64.4)	37.9 (21.4 to 65.5)	30.4 (18.7 to 50.8)	30.2 (17.7 to 52.8)	30.2 (18.3 to 49.5)	-16.7 (-43 to 22.1)	-13 (-45.3 to 41.1)	-20.4 (-46.8 to 22.9)
		DALYs	668.9 (416.1 to 1065.5)	646.4 (386.7 to 1070.7)	686.7 (412.1 to 1089.4)	631.4 (410.5 to 929.6)	631 (398.7 to 958.1)	629.3 (391 to 933.6)	-5.6 (-34.8 to 38.4)	-2.4 (-37.6 to 51.6)	-8.4 (-39.5 to 41.8)
		YLLs	611.4 (373.3 to 976.8)	580.2 (340.1 to 967)	635.9 (375.8 to 1019.4)	546.7 (346.4 to 802.5)	526.6 (325.7 to 801.2)	557.8 (340.5 to 840.2)	-10.6 (-39.7 to 33.8)	-9.2 (-43.4 to 44.9)	-12.3 (-43.2 to 38.7)
		YLDs	57.5 (33 to 95.2)	66.2 (37 to 113.7)	50.8 (28.5 to 86.9)	84.7 (50.5 to 127.9)	104.4 (62.6 to 168.1)	71.5 (41.8 to 109.3)	47.3 (14.4 to 89.9)	57.8 (13.7 to 116.9)	40.8 (3 to 93.1)
	High LDL cholesterol	Deaths	13.9 (4.7 to 31.3)	13.9 (4.7 to 31.2)	13.8 (4.7 to 30.9)	13.8 (5.3 to 29)	14.2 (5.1 to 30.2)	13.3 (5 to 28.2)	-0.6 (-27.2 to 39.4)	2.3 (-27.2 to 52.1)	-3.6 (-29.7 to 38.3)
		DALYs	282 (145.1 to 506.7)	294.9 (152 to 528.4)	273.2 (138.2 to 496.2)	310.6 (174.2 to 522.4)	329.7 (184.3 to 545.5)	296.7 (163.6 to 504.8)	10.2 (-18 to 52.2)	11.8 (-18.2 to 57.3)	8.6 (-20.2 to 52.6)
		YLLs	241.3 (117.4 to 455.4)	243.2 (119.7 to 461)	240 (113.9 to 450.7)	252.1 (135.7 to 439.7)	253.4 (134.1 to 443.1)	250 (129.9 to 432.1)	4.5 (-26.5 to 53)	4.2 (-29.6 to 57.3)	4.2 (-29.1 to 54.1)
		YLDs	40.7 (22.5 to 68.9)	51.7 (28.2 to 90)	33.2 (18.6 to 56.3)	58.5 (34.8 to 96)	76.3 (45.3 to 123.9)	46.6 (27.5 to 75.9)	43.8 (26.4 to 65.8)	47.6 (24.6 to 75.3)	40.7 (23.3 to 64.2)
	High systolic blood pressure	Deaths	70.8 (50.8 to 93.4)	71 (48.7 to 97.3)	70.8 (47.4 to 95.2)	51.8 (37.5 to 65.5)	48.2 (33.7 to 64.8)	53.8 (36.2 to 67.8)	-26.8 (-46.5 to 0.2)	-32.2 (-54.9 to 0.9)	-23.9 (-45 to 7.9)
		DALYs	1499.7 (1105.4 to 1956.7)	1552.8 (1124.1 to 2088.8)	1467 (1016.2 to 1940.5)	1131.9 (852.2 to 1403.6)	1060.5 (778.5 to 1365.4)	1174.3 (842.9 to 1447.1)	-24.5 (-44.5 to 3.1)	-31.7 (-53 to -3.5)	-20 (-42.9 to 13.4)
		YLLs	1366.2 (976.8 to 1810.3)	1390.6 (980.5 to 1900.8)	1354.2 (912.3 to 1814.6)	981.6 (714.7 to 1225.7)	887 (631.4 to 1175.1)	1039.9 (715.5 to 1306.2)	-28.2 (-49.1 to 1.2)	-36.2 (-57.5 to -5.8)	-23.2 (-46.8 to 11.8)
		YLDs	133.4 (91.7 to 180.5)	162.3 (109.6 to 224.1)	112.8 (77.7 to 153.7)	150.3 (106.6 to 197.8)	173.5 (119.3 to 233.2)	134.4 (92.9 to 177.9)	12.6 (-1 to 28.3)	6.9 (-14.3 to 33.9)	19.1 (3.3 to 39.4)
	High temperature	Deaths	4.9 (0.5 to 10.9)	5.1 (0.5 to 11)	4.9 (0.5 to 10.8)	4.2 (1.5 to 7.8)	4.2 (1.5 to 7.6)	4.1 (1.4 to 7.8)	-15.3 (-56.4 to 41.1)	-16.2 (-60.2 to 44.4)	-15.1 (-58.8 to 38.1)
		YLLs	87.9 (8.9 to 189.9)	91.4 (8.6 to 198)	85.5 (8.4 to 187.1)	74.1 (26.3 to 139.7)	74.1 (26.4 to 137.8)	73.8 (25.8 to 139.5)	-15.7 (-58 to 46.5)	-19 (-63 to 40.4)	-13.7 (-60.3 to 48.6)
	Household air pollution from solid fuels	Deaths	23.3 (12.2 to 37.1)	26.8 (14.7 to 41.9)	20.5 (9.8 to 34.9)	0.1 (0 to 0.2)	0.1 (0 to 0.2)	0.1 (0 to 0.2)	-99.7 (-99.9 to -99.1)	-99.7 (-99.9 to -99)	-99.7 (-99.9 to -99.1)
		DALYs	520.8 (271.1 to 842.8)	622.5 (339.6 to 944.5)	449.1 (216.8 to 759.8)	1.6 (0.4 to 4.4)	2.1 (0.5 to 5.8)	1.3 (0.3 to 3.5)	-99.7 (-99.9 to -99)	-99.7 (-99.9 to -99)	-99.7 (-99.9 to -99.1)
		YLLs	470.7 (241.2 to 772.3)	553.2 (296.7 to 864.2)	412.4 (193.1 to 709.3)	1.4 (0.3 to 3.8)	1.7 (0.4 to 4.8)	1.1 (0.3 to 3.1)	-99.7 (-99.9 to -99.1)	-99.7 (-99.9 to -99)	-99.7 (-99.9 to -99.1)
		YLDs	50.1 (25.7 to 79.5)	69.2 (37.2 to 108)	36.7 (17.6 to 61.1)	0.2 (0.1 to 0.7)	0.4 (0.1 to 1)	0.2 (0 to 0.4)	-99.5 (-99.9 to -98.6)	-99.5 (-99.9 to -98.4)	-99.6 (-99.9 to -98.7)
	Kidney dysfunction	Deaths	13.1 (8.9 to 17.9)	14.9 (9.9 to 20.8)	11.6 (7.6 to 15.9)	12.9 (8.3 to 17.3)	13.2 (8.2 to 18.6)	12.6 (8 to 17.1)	-1.7 (-29.5 to 31.7)	-11.3 (-39.3 to 25.8)	8.3 (-21.5 to 49.4)
		DALYs	272.7 (199.1 to 356.4)	321.4 (230.4 to 427.6)	237.3 (164.2 to 316.2)	285.8 (208 to 363.4)	308.7 (225.3 to 403.1)	270.5 (188.7 to 344.3)	4.8 (-21.4 to 39.7)	-3.9 (-30.3 to 31.2)	14 (-15.4 to 54.4)
		YLLs	246.5 (175.3 to 328.8)	285.2 (197.4 to 387.4)	218.4 (146.3 to 294.7)	244.4 (171.4 to 315.7)	253.4 (174.7 to 337)	238.1 (159.9 to 304.8)	-0.9 (-28.6 to 37.1)	-11.1 (-38.7 to 27.8)	9 (-22.1 to 52.5)
		YLDs	26.1 (17.7 to 36.2)	36.2 (24.3 to 50.8)	18.9 (12.9 to 25.9)	41.4 (28.5 to 55.6)	55.4 (38.4 to 74.7)	32.4 (22.1 to 43.8)	58.5 (41.8 to 76.3)	52.9 (32.9 to 74.1)	70.9 (50.6 to 93.4)
	Lead exposure	Deaths	7.2 (4 to 10.9)	5.3 (2.3 to 8.8)	8.8 (5.1 to 13)	4.2 (2.2 to 6.6)	2.9 (1.1 to 5.2)	5.1 (2.8 to 7.6)	-41.9 (-58.4 to -20.8)	-45 (-63.3 to -23.8)	-41.9 (-58.2 to -18)
		DALYs	159 (88.4 to 241.1)	117 (48.8 to 194.4)	189.6 (112.1 to 282.1)	82.9 (40 to 132.5)	56.8 (18.3 to 104.5)	100.4 (52.2 to 151)	-47.8 (-64.1 to -28.8)	-51.4 (-69.5 to -33.6)	-47.1 (-63.8 to -25.4)
		YLLs	145.2 (80 to 221.1)	104.7 (44 to 177.2)	174.9 (101.2 to 264.7)	72.8 (35.1 to 116.3)	47.9 (15.4 to 87.9)	89.5 (46.4 to 136.7)	-49.9 (-66.3 to -29)	-54.3 (-72.2 to -35.3)	-48.8 (-65.5 to -26.1)
		YLDs	13.7 (7.1 to 21.5)	12.2 (4.5 to 21.7)	14.7 (8.5 to 22.4)	10.1 (4.3 to 17.4)	8.9 (2.5 to 17.3)	10.9 (5.3 to 17.6)	-26.4 (-42.6 to -14.6)	-27.1 (-49.2 to -13)	-26.3 (-41.6 to -13.9)
	Low physical activity	Deaths	10.2 (2.6 to 19.6)	11.1 (3 to 20.4)	9.4 (2.1 to 19.1)	9.2 (2.6 to 16.5)	10.1 (3.1 to 18.1)	8.4 (2.1 to 15.1)	-9.8 (-34 to 32.2)	-8.4 (-36 to 41.2)	-10 (-34.8 to 39.5)
		DALYs	156.3 (36.4 to 316.3)	177.6 (44.9 to 347.8)	139.4 (27.7 to 295.5)	159.9 (40.9 to 298.4)	188.2 (52.7 to 336.9)	140.4 (31.8 to 270.1)	2.3 (-24.2 to 50.2)	6 (-24.6 to 60.5)	0.7 (-28.2 to 52.1)
		YLLs	138.1 (32.6 to 280.2)	152.6 (39.2 to 302.1)	126.2 (25.5 to 271.3)	133.3 (33.7 to 246.3)	149.7 (41 to 271.8)	121.7 (27.4 to 233.5)	-3.5 (-30.9 to 43.8)	-1.9 (-33.5 to 55.6)	-3.6 (-31.7 to 46.6)
		YLDs	18.2 (3.6 to 40.7)	25 (5.3 to 54.1)	13.1 (2.2 to 30.8)	26.7 (5.9 to 53.3)	38.5 (9.6 to 72.7)	18.7 (3.4 to 40.4)	46.2 (18.3 to 108.5)	54.2 (21.9 to 127.1)	42.6 (13.3 to 109.3)
	Low temperature	Deaths	6 (0 to 12.3)	6.1 (0 to 12.6)	5.9 (0 to 12.2)	3.7 (-0.3 to 8)	3.8 (-0.3 to 8.3)	3.7 (-0.3 to 7.9)	-37.1 (-73.4 to 2)	-37.6 (-74.8 to 7.1)	-37.1 (-73.7 to 2.7)
		YLLs	105.7 (0.3 to 221.2)	109.6 (0.3 to 227.4)	103.2 (0.3 to 217.6)	66.3 (-4.6 to 142.4)	66.2 (-4.9 to 145)	66.1 (-4.5 to 143)	-37.3 (-73.4 to 7.3)	-39.6 (-76.1 to 5.8)	-36 (-73.6 to 6.3)
	Secondhand smoke	Deaths	5.2 (3.5 to 7.1)	5.8 (4 to 8.1)	4.8 (3.1 to 6.8)	3.5 (2.4 to 4.6)	4.1 (2.9 to 5.7)	3 (2 to 4.1)	-33.4 (-52.3 to -6.9)	-29.5 (-51.8 to 2.3)	-37.5 (-55.8 to -9.5)
		DALYs	123.1 (82.2 to 169.2)	152.8 (102.4 to 213.3)	105.1 (67.3 to 149.5)	86.4 (59.2 to 115.7)	112.3 (78.7 to 152.8)	69.7 (45.3 to 95.6)	-29.8 (-49.7 to -0.8)	-26.5 (-48.8 to 5.8)	-33.7 (-53.6 to -3.6)

Location	Risk factor	Measure	1990 Both	1990 Female	1990 Male	2019 Both	2019 Female	2019 Male	% Change (1990 to 2019) Both	% Change (1990 to 2019) Female	% Change (1990 to 2019) Male
		YLLs	115.7 (76.1 to 161.5)	141.9 (93.8 to 201.4)	100 (63.2 to 143.3)	78.3 (52.6 to 106.4)	99.4 (67.7 to 138)	64.6 (41.6 to 89.1)	-32.3 (-52.8 to -2.1)	-29.9 (-52.6 to 3.9)	-35.4 (-55.4 to -4.3)
		YLDs	7.4 (4.8 to 10.4)	10.9 (7.1 to 15.5)	5.1 (3.3 to 7.2)	8.1 (5.3 to 11.1)	12.8 (8.2 to 17.8)	5.1 (3.3 to 7.2)	10.2 (-2.7 to 24.5)	17.6 (2.3 to 34.7)	-0.7 (-17.4 to 19.1)
	Smoking	Deaths	10.8 (7.8 to 14.9)	3.7 (2.3 to 5.8)	16.1 (11.2 to 22.2)	8.2 (5.8 to 10.3)	2.2 (1.5 to 3.2)	12.1 (8.5 to 15)	-24.6 (-48.4 to 11)	-39.3 (-66.8 to 7.6)	-24.9 (-48.2 to 10.6)
		DALYs	302.1 (218.7 to 420.8)	103.5 (64.7 to 164.1)	437 (307.3 to 603.7)	242.5 (174.3 to 305.2)	65.6 (43.5 to 94.4)	357.3 (255.2 to 454.1)	-19.7 (-44.4 to 19)	-36.6 (-63.7 to 8.1)	-18.3 (-43.4 to 20.2)
		YLLs	275 (195 to 391)	91.9 (56.4 to 150.2)	399.7 (275.1 to 566.8)	211.1 (146.9 to 270.3)	54 (35.1 to 80.1)	313.1 (216.5 to 403.2)	-23.2 (-48.6 to 17.6)	-41.2 (-67.8 to 4.9)	-21.7 (-47.4 to 20.1)
		YLDs	27.1 (18.6 to 36.6)	11.6 (6.6 to 18.2)	37.4 (26 to 50.7)	31.4 (22.4 to 41.4)	11.6 (7.3 to 17.1)	44.2 (31.4 to 58.2)	15.8 (-4.5 to 40.8)	0 (-35.4 to 57.2)	18.3 (-0.9 to 40.7)
Sudan	All risk factors	Deaths	152.8 (107.9 to 199.6)	158.9 (106.7 to 204.1)	146.6 (102.7 to 208.9)	111.2 (81.4 to 157.5)	119.3 (84.1 to 160.8)	104.2 (71.4 to 162)	-27.2 (-40.6 to -8.8)	-24.9 (-38.6 to -6.2)	-28.9 (-44.5 to -7.8)
		DALYs	3265.7 (2409.1 to 4278.2)	3465.8 (2476.4 to 4432.1)	3072.2 (2180.6 to 4359.6)	2309.3 (1736.6 to 3195.8)	2498.4 (1829.2 to 3293.7)	2147.4 (1506.8 to 3305.8)	-29.3 (-43.2 to -11.8)	-27.9 (-41.6 to -9)	-30.1 (-45.9 to -9.3)
		YLLs	3041.3 (2172.1 to 4025.6)	3194.1 (2187.2 to 4159.9)	2891.9 (2003.6 to 4154.9)	2059.5 (1500.5 to 2954)	2189.5 (1506.8 to 2942.5)	1948.3 (1308.6 to 3083.6)	-32.3 (-46.9 to -13.5)	-31.5 (-45.8 to -11.8)	-32.6 (-49.4 to -10.8)
		YLDs	224.4 (162.3 to 284.5)	271.7 (196.7 to 346.8)	180.2 (129.3 to 229.8)	249.8 (182.7 to 318.7)	308.9 (224.6 to 395.9)	199.1 (143.4 to 252.7)	11.4 (5.3 to 17.4)	13.7 (5.8 to 21.7)	10.5 (2.4 to 18.7)
	Alcohol use	Deaths	1.1 (0.3 to 2.3)	0.3 (-0.1 to 0.8)	2 (0.6 to 3.8)	0 (-0.1 to 0)	0 (-0.1 to 0)	0 (-0.1 to 0.1)	-102.2 (-112.8 to -98.4)	-110.6 (-222.1 to 22)	-101.2 (-110 to -97.2)
		DALYs	34.6 (9.9 to 65.5)	8.5 (-1.1 to 21.5)	59 (18.9 to 113.1)	-0.5 (-1.8 to 0.6)	-0.6 (-1.2 to 0)	-0.5 (-2.7 to 1.7)	-101.5 (-109 to -98.2)	-107 (-197.9 to -16)	-100.8 (-107.1 to -97.2)
		YLLs	33.1 (9.5 to 63.1)	8.3 (-0.6 to 20.7)	56.4 (17.9 to 109)	-0.4 (-1.5 to 0.6)	-0.5 (-1 to 0)	-0.3 (-2.4 to 1.6)	-101.3 (-107.9 to -98.2)	-105.9 (-164.5 to 0.1)	-100.6 (-106.3 to -97.4)
		YLDs	1.5 (0.2 to 3)	0.2 (-0.4 to 1)	2.7 (0.7 to 5)	-0.1 (-0.3 to 0)	-0.1 (-0.2 to 0)	-0.1 (-0.4 to 0.2)	-107.4 (-146.4 to -96.9)	-153 (-569.4 to 358.7)	-104.2 (-130.2 to -94.4)
	Ambient particulate matter pollution	Deaths	7.9 (2.6 to 17.6)	6.8 (2.2 to 15.9)	9 (2.7 to 20.5)	24 (13.4 to 38.7)	23.4 (12.4 to 37.7)	24.5 (13 to 43.6)	203 (60.9 to 572.6)	243.9 (84.8 to 661.5)	172.8 (39.2 to 515.8)
		DALYs	182 (59.4 to 403.4)	159.6 (52 to 372.3)	202.5 (61.7 to 467.1)	558.6 (317.3 to 894.1)	552.4 (304.5 to 873.8)	563.9 (313.9 to 977.5)	206.9 (59 to 596.3)	246.1 (82.6 to 691.4)	178.5 (39.7 to 553.4)
		YLLs	169.4 (55 to 378.6)	146.7 (46.9 to 342.4)	190.2 (58 to 439.4)	495.4 (275.2 to 822)	480.1 (260.2 to 776.2)	508.6 (273.3 to 902.2)	192.4 (49.8 to 577.4)	227.2 (71.2 to 655.7)	167.4 (32.3 to 532)
		YLDs	12.6 (4.1 to 28.9)	12.8 (4.2 to 30.1)	12.3 (3.9 to 27.7)	63.2 (33.9 to 97.4)	72.3 (37.5 to 113.6)	55.3 (30.6 to 82.8)	402.5 (167.5 to 1013.4)	462.6 (195.5 to 1148.5)	349.2 (140 to 930)
	Diet high in red meat	Deaths	4.7 (1.6 to 8.1)	4.9 (1.6 to 8.8)	4.5 (1.4 to 8)	3.2 (1.1 to 5.8)	3.4 (1.1 to 5.9)	3 (1 to 5.6)	-32.2 (-58 to 10.5)	-31.1 (-59.1 to 15.1)	-32.8 (-59.5 to 14.4)
		DALYs	125.2 (39.4 to 213)	134.2 (41.8 to 237.2)	116.3 (34.1 to 209.5)	85.3 (29 to 150.8)	91.8 (30.3 to 159.8)	79.4 (26.1 to 147.6)	-31.8 (-58.1 to 15.6)	-31.6 (-60.2 to 21.4)	-31.7 (-59.3 to 19.3)
		YLLs	115.9 (34.7 to 199.3)	123 (35.8 to 219.6)	108.8 (30.7 to 200.2)	74.9 (25.5 to 135.4)	79 (26 to 139.9)	71.1 (23.6 to 134.5)	-35.4 (-60.9 to 13.5)	-35.7 (-62.6 to 17.2)	-34.7 (-61.3 to 17)
		YLDs	9.3 (3.2 to 15.7)	11.2 (3.7 to 19.2)	7.5 (2.5 to 12.6)	10.4 (2.9 to 17.8)	12.8 (3.5 to 22.4)	8.3 (2.3 to 14.6)	12.1 (-18.9 to 39.4)	14 (-20.8 to 52)	10.8 (-23.6 to 47.2)
	Diet high in sodium	Deaths	3.1 (0.4 to 13.5)	2.3 (0.5 to 10.1)	3.9 (0.3 to 17)	2.2 (0.3 to 9.1)	1.6 (0.3 to 6.7)	2.6 (0.2 to 11.5)	-31.4 (-59.5 to 32.7)	-31.5 (-67.8 to 47.6)	-32.5 (-60.3 to 47)
		DALYs	76.6 (8.7 to 327.9)	57.4 (9.8 to 258.2)	94.3 (6.3 to 406.8)	51.6 (6.1 to 210.2)	38.1 (6.8 to 164.4)	63.4 (4.4 to 264.8)	-32.7 (-62 to 30.1)	-33.6 (-69.6 to 50.2)	-32.7 (-58.1 to 44.9)
		YLLs	71.6 (7.9 to 309.7)	52.9 (9 to 238.5)	88.7 (5.9 to 385)	46.1 (5.4 to 190.2)	33.2 (6 to 141.9)	57.3 (3.9 to 244.1)	-35.6 (-63.9 to 27.3)	-37.2 (-71.6 to 43.2)	-35.4 (-60.4 to 40.5)
		YLDs	5.1 (0.6 to 20.6)	4.5 (0.7 to 19.6)	5.6 (0.4 to 21.9)	5.6 (0.7 to 22.7)	4.9 (0.8 to 21.2)	6.2 (0.4 to 24.2)	9.3 (-38.3 to 98.7)	8.3 (-50.5 to 139.8)	9.9 (-28.1 to 119.9)
	Diet low in fiber	Deaths	7.9 (1.8 to 15.2)	8.2 (1.7 to 15.6)	7.6 (1.8 to 14.9)	4.3 (0.9 to 8.6)	4.6 (0.9 to 9.1)	4.1 (0.8 to 8.8)	-45.5 (-61.5 to -27.5)	-44.6 (-63.6 to -22.6)	-46.1 (-64.8 to -22.4)
		DALYs	194.6 (41.6 to 372.4)	208.1 (43.5 to 403.1)	181.1 (40.7 to 356.2)	104.6 (20.7 to 206)	112.1 (22.2 to 222.4)	97.9 (19.1 to 204.6)	-46.2 (-62.7 to -28.3)	-46.1 (-65 to -25.2)	-46 (-63.4 to -20.6)
		YLLs	179.9 (37.9 to 349)	190.5 (39.5 to 373.5)	169.3 (37.3 to 335.8)	92 (18 to 185.2)	96.7 (19.1 to 195.5)	87.7 (16.8 to 188.4)	-48.9 (-65.4 to -29.7)	-49.2 (-67.6 to -27.4)	-48.2 (-65.8 to -22.6)
		YLDs	14.6 (3.3 to 27.1)	17.6 (4 to 33)	11.8 (2.8 to 21.9)	12.6 (2.6 to 23.9)	15.4 (3 to 29.4)	10.1 (2.2 to 19.8)	-13.6 (-34.1 to 3)	-12.6 (-39.6 to 11.3)	-14.1 (-39.8 to 10.8)
	Diet low in fruits	Deaths	10.9 (5.3 to 19.5)	11.5 (5.6 to 20.1)	10.4 (4.8 to 19.6)	6 (2.5 to 11.2)	6.4 (2.7 to 11.7)	5.6 (2.2 to 11.6)	-45.1 (-64.2 to -20.5)	-44 (-64.8 to -16.5)	-45.7 (-65.8 to -18.5)
		DALYs	279.6 (141 to 487.5)	302 (150.6 to 523.6)	257.7 (123.5 to 479.4)	153.1 (65.8 to 282.2)	166 (72.8 to 289.7)	141.7 (57.2 to 283.2)	-45.2 (-64.5 to -19)	-45 (-66.2 to -18.2)	-45 (-65.2 to -16.4)
		YLLs	261.1 (128.2 to 459.4)	279.5 (136.8 to 493.1)	242.9 (116.8 to 457.2)	135.8 (58.7 to 259.9)	144.6 (64.5 to 263.2)	128 (51.8 to 262.9)	-48 (-66.7 to -22.6)	-48.3 (-68.2 to -20.9)	-47.3 (-67.3 to -17.7)
		YLDs	18.5 (8.1 to 32.4)	22.5 (9.7 to 39)	14.8 (6.3 to 26.3)	17.3 (6.4 to 31.8)	21.4 (7.9 to 40)	13.6 (5.1 to 24.8)	-6.6 (-29 to 9.4)	-5 (-30.2 to 14.3)	-7.6 (-32.5 to 11.7)
	Diet low in vegetables	Deaths	7 (2.2 to 12.4)	7.2 (2.2 to 12.5)	6.9 (2.2 to 12.9)	3.6 (1.2 to 6.6)	3.7 (1.2 to 6.6)	3.4 (1.2 to 6.7)	-49.5 (-68.6 to -19)	-48.5 (-68.7 to -14.4)	-50.2 (-69.4 to -19.9)
		DALYs	174.9 (50.6 to 312.4)	182.8 (50.5 to 326.2)	167 (49.1 to 315.8)	86.7 (28.7 to 162.7)	90.8 (28.4 to 165.8)	83.1 (27.1 to 165.4)	-50.4 (-69.1 to -17.3)	-50.3 (-70.3 to -14.2)	-50.3 (-70.1 to -17.5)
		YLLs	163.8 (45.6 to 298.2)	169.7 (45.1 to 308)	157.9 (45.5 to 300.5)	77.2 (25.5 to 146)	79.4 (25.2 to 145.8)	75.2 (24.8 to 152.9)	-52.9 (-70.8 to -20.1)	-53.2 (-72.6 to -16.3)	-52.3 (-71.4 to -20)
		YLDs	11.1 (4 to 19.1)	13.1 (4.6 to 22.9)	9.1 (3.2 to 15.9)	9.5 (2.8 to 17.2)	11.5 (3.3 to 20.9)	7.8 (2.4 to 13.9)	-13.9 (-41.2 to 8.9)	-12.7 (-43 to 12.4)	-14.4 (-42.1 to 9.4)
	Diet low in whole grains	Deaths	7 (3.7 to 10.5)	7.4 (4 to 11)	6.5 (3.4 to 11)	7.4 (3.9 to 11.6)	8 (4.3 to 11.6)	6.8 (3.4 to 12.1)	5.4 (-16.1 to 32)	7 (-16.1 to 37.7)	4.8 (-23.8 to 39)
		DALYs	138.7 (70 to 213.2)	151.1 (75.4 to 224.1)	126.8 (63.5 to 222.2)	154.9 (78.1 to 238.1)	170.4 (86.9 to 253.7)	141.2 (66.6 to 249.6)	11.6 (-11.4 to 41)	12.8 (-11.5 to 43.2)	11.3 (-18 to 50)
		YLLs	120.3 (60.3 to 188.1)	128.3 (63.7 to 197)	112.4 (55 to 202.5)	132.1 (66.1 to 212.8)	141.9 (70.7 to 215.3)	123.4 (57.2 to 229.7)	9.8 (-15.7 to 42.4)	10.6 (-17.2 to 46)	9.8 (-22.6 to 53.7)
		YLDs	18.5 (9.4 to 27.2)	22.8 (11.9 to 33.9)	14.4 (7 to 21.4)	22.8 (11.1 to 33.8)	28.5 (14.1 to 42.2)	17.8 (8.6 to 26.5)	23.4 (14.1 to 32)	24.9 (13.3 to 36.6)	23.5 (11.5 to 36.8)
	High body-mass index	Deaths	22.8 (10.6 to 38.5)	29.9 (15.5 to 48.9)	16.3 (5.8 to 32.5)	25.4 (14.3 to 40.4)	29.7 (16.7 to 46)	21.9 (11 to 39.6)	11.6 (-20.9 to 75.9)	-0.7 (-30.6 to 49.6)	34.5 (-12.8 to 166.2)
		DALYs	732.5 (365.4 to 1197.7)	970.2 (514.3 to 1520.3)	509.7 (190.5 to 993.3)	788.1 (470.6 to 1219.2)	924.7 (560.3 to 1368)	668.6 (345.9 to 1168.2)	7.6 (-24.6 to 73.4)	-4.7 (-34.2 to 44.4)	31.2 (-14.5 to 163.1)
		YLLs	680.5 (333.8 to 1120.4)	895.5 (466.7 to 1418.9)	478.9 (180.7 to 964.4)	692.4 (394.3 to 1115.8)	798.7 (462.3 to 1223)	600.1 (298.6 to 1085.3)	1.7 (-30.8 to 67.3)	-10.8 (-39.9 to 39.7)	25.3 (-20.7 to 154.6)
		YLDs	52 (26 to 83.3)	74.7 (38.6 to 115.2)	30.8 (12.3 to 54.8)	95.6 (58.9 to 137.7)	126 (80.9 to 178.9)	68.5 (38.8 to 102.8)	83.9 (49.2 to 166.3)	68.8 (33.4 to 129.8)	122.8 (65.6 to 309.9)
	High fasting plasma glucose	Deaths	29 (15.6 to 53.4)	29.3 (14.8 to 55.6)	28.6 (14.9 to 54)	40.3 (21.4 to 74.9)	42.7 (22.1 to 79.2)	38.3 (19.9 to 71)	39.1 (-3.2 to 108.5)	45.6 (-3.3 to 120.8)	33.6 (-12.2 to 111)
		DALYs	540.5 (314 to 925.8)	555.6 (313.8 to 972.7)	526 (298.2 to 921.7)	789.9 (460.5 to 1335.5)	841.9 (486.9 to 1443.1)	747 (416.1 to 1280.1)	46.2 (3.8 to 110.5)	51.5 (1.3 to 124.4)	42 (-5.3 to 113.3)
		YLLs	504.7 (290.6 to 866.4)	512.9 (284.5 to 899.9)	496.5 (275 to 877.2)	707.2 (403.4 to 1205.5)	740.6 (421.1 to 1280.8)	679.5 (376.7 to 1196.2)	40.1 (-1.2 to 104.6)	44.4 (-4.4 to 114.1)	36.9 (-10.2 to 107.7)

Location	Risk factor	Measure	1990 Both	1990 Female	1990 Male	2019 Both	2019 Female	2019 Male	% Change (1990 to 2019) Both	% Change (1990 to 2019) Female	% Change (1990 to 2019) Male
		YLDs	35.8 (20.1 to 61.4)	42.7 (23.6 to 75.3)	29.5 (15.8 to 51.3)	82.7 (45.1 to 136.3)	101.3 (54.3 to 170.4)	67.4 (37.1 to 112.6)	131 (81.8 to 197.7)	137.4 (77.2 to 219.4)	128.7 (66.6 to 206.1)
	High LDL cholesterol	Deaths	13 (4.5 to 29.5)	14 (4.4 to 31.7)	12 (4 to 27.4)	15.6 (5.7 to 34.2)	17.1 (6.1 to 36.1)	14.3 (5.2 to 31.9)	20.2 (-4 to 58.5)	22.3 (-5.2 to 61.9)	19.4 (-11.5 to 63.7)
		DALYs	272.8 (139 to 499.5)	301.9 (150.2 to 554.9)	244.6 (122.4 to 472.5)	351.2 (191.4 to 615.1)	391.5 (211 to 682.9)	315.1 (159.5 to 590.1)	28.8 (1.7 to 68.7)	29.7 (0.2 to 70.4)	28.8 (-4.7 to 78.7)
		YLLs	233.2 (112.6 to 450)	252.6 (113.9 to 487.4)	214.2 (102.1 to 421.9)	295.8 (149.1 to 547.9)	321.7 (156.8 to 587.6)	272.6 (130.2 to 541.8)	26.9 (-4.5 to 75)	27.3 (-7.6 to 76.6)	27.2 (-10.5 to 84.6)
		YLDs	39.6 (23.3 to 66.2)	49.3 (29.1 to 82.3)	30.3 (17.7 to 50.6)	55.4 (33.8 to 89.4)	69.8 (42.3 to 113.6)	42.5 (25.9 to 67.6)	40 (29.1 to 52.3)	41.5 (28.2 to 57.4)	40.1 (25.7 to 58.1)
	High systolic blood pressure	Deaths	95.8 (65.2 to 131.6)	105.8 (69.4 to 143)	86 (56.7 to 129.1)	73.4 (50.8 to 105.5)	83.3 (56 to 116.4)	65 (42.3 to 106.4)	-23.4 (-39.3 to -1.1)	-21.2 (-38.8 to 4.1)	-24.4 (-44 to -0.3)
		DALYs	2150.6 (1536.7 to 2919.8)	2404.5 (1649.1 to 3249.9)	1911.2 (1301.9 to 2779.7)	1592.1 (1151.5 to 2261.1)	1800.8 (1287.8 to 2426.3)	1417.1 (945.5 to 2243.5)	-26 (-41.5 to -4.4)	-25.1 (-41.6 to -2.1)	-25.9 (-44.3 to -1)
		YLLs	2003.2 (1397.5 to 2766.7)	2218.3 (1472.1 to 3049)	1799.6 (1195.7 to 2661.5)	1417.8 (983.4 to 2096.3)	1577.7 (1077.4 to 2171.1)	1284.1 (833.1 to 2129.1)	-29.2 (-45.4 to -7)	-28.9 (-45.8 to -5.1)	-28.6 (-47.5 to -2.9)
		YLDs	147.4 (105.8 to 192.6)	186.2 (130.3 to 242.7)	111.6 (78.9 to 147.8)	174.3 (125.3 to 228.3)	223 (159 to 293.6)	133 (94.6 to 172.9)	18.3 (7.1 to 30.8)	19.8 (5 to 38)	19.2 (4.9 to 35.7)
	High temperature	Deaths	6.6 (-1 to 16.1)	6.9 (-1 to 16)	6.4 (-1 to 15.9)	5.5 (1.9 to 12)	5.8 (2 to 12)	5.2 (1.9 to 11.7)	-17.6 (-69.7 to 23.1)	-15.2 (-69.6 to 27.9)	-19.5 (-72.2 to 18.3)
		YLLs	134.7 (-20.9 to 321.4)	142.4 (-21.4 to 334.2)	127.4 (-20.3 to 306.1)	101 (35.3 to 219.9)	107 (37.1 to 217.5)	95.8 (33.8 to 220)	-25.1 (-74.3 to 11)	-24.9 (-73.3 to 12.2)	-24.8 (-76 to 14.3)
	Household air pollution from solid fuels	Deaths	65.4 (42.7 to 96.5)	70.8 (45.1 to 102.8)	60.2 (37.1 to 92.9)	16.2 (8.9 to 26.7)	19.5 (11.2 to 30.3)	13.5 (6.6 to 25.1)	-75.2 (-84.5 to -62)	-72.4 (-82.3 to -59.3)	-77.6 (-87.4 to -64.4)
		DALYs	1494.2 (1018.3 to 2095.6)	1645.9 (1102.8 to 2251.3)	1348.8 (864.2 to 2025.6)	380.3 (213.6 to 621.6)	461.4 (270.5 to 712.3)	310.2 (154.1 to 568)	-74.5 (-84.5 to -61.4)	-72 (-81.8 to -58.5)	-77 (-87.4 to -63)
		YLLs	1388 (915 to 1984.8)	1513.8 (979 to 2103.3)	1267 (790.9 to 1926.4)	336 (183.3 to 560)	401.2 (229.1 to 631.5)	279.9 (134.8 to 524.1)	-75.8 (-85.4 to -63)	-73.5 (-83.3 to -59.6)	-77.9 (-88.1 to -63.7)
		YLDs	106.1 (71.1 to 145.9)	132.1 (88.3 to 182.1)	81.9 (54.1 to 113.9)	44.3 (25.4 to 68.2)	60.2 (36.1 to 89.7)	30.3 (15.7 to 49.9)	-58.3 (-71.8 to -42.6)	-54.4 (-68.4 to -38.3)	-62.9 (-77.1 to -46.5)
	Kidney dysfunction	Deaths	11.8 (7.9 to 16.7)	13.3 (8.4 to 19.3)	10.3 (6.8 to 15.4)	12.2 (7.5 to 18.6)	13.6 (8.3 to 19.9)	11.1 (6.6 to 18.3)	3.6 (-19.7 to 32.8)	2 (-21.4 to 33.7)	7 (-21 to 43)
		DALYs	256.1 (181.2 to 349.8)	296.9 (203.4 to 414.5)	218.3 (148.1 to 320.1)	260.1 (182.5 to 369.4)	296.6 (201.3 to 407.7)	229.2 (153.5 to 353.4)	1.6 (-18.7 to 26.9)	-0.1 (-20.9 to 29.4)	5 (-21.3 to 36.3)
		YLLs	236.4 (164.9 to 326.5)	271.1 (178 to 384.1)	204.1 (136.4 to 303.7)	230.2 (156.5 to 336.8)	257.7 (167 to 363)	206.9 (135.5 to 332.1)	-2.6 (-24.3 to 24.7)	-4.9 (-27.1 to 26)	1.3 (-26.1 to 35.9)
		YLDs	19.7 (13.4 to 26.5)	25.9 (17.7 to 35.1)	14.2 (9.6 to 18.8)	29.9 (20.3 to 40.7)	38.9 (26.4 to 53)	22.3 (15.1 to 30.3)	51.6 (39.2 to 62.8)	50.4 (35.7 to 63.9)	57.6 (40.6 to 75.8)
	Lead exposure	Deaths	16.1 (10.3 to 24.3)	14 (8.3 to 20.8)	18 (11.3 to 28.4)	11.3 (7.1 to 17.4)	10 (5.8 to 15.3)	12.4 (7.6 to 20)	-29.9 (-45.4 to -9.9)	-28.6 (-44.3 to -7.2)	-31.6 (-48.5 to -9.1)
		DALYs	375.8 (243.7 to 547.6)	331.7 (200.7 to 486.7)	416.3 (263.3 to 635.7)	230.7 (144.7 to 348.1)	205.4 (123.8 to 303.5)	252.3 (155 to 402.5)	-38.6 (-52.1 to -21.3)	-38.1 (-51.8 to -20.2)	-39.4 (-54.4 to -20.5)
		YLLs	350.9 (223.7 to 520.4)	306.3 (179.7 to 453.9)	392 (243.1 to 607.7)	207.2 (128.3 to 321.9)	180.9 (104.4 to 274.8)	229.6 (136.4 to 377)	-41 (-54.7 to -23.1)	-40.9 (-55 to -22.4)	-41.4 (-57 to -21.1)
		YLDs	24.9 (15.8 to 35.1)	25.5 (15.7 to 36.5)	24.3 (15.7 to 33.9)	23.6 (14.5 to 33.8)	24.6 (14.5 to 36.7)	22.7 (14.3 to 32)	-5.2 (-14.7 to 3.3)	-3.6 (-15.8 to 7.6)	-6.3 (-17 to 5.3)
	Low physical activity	Deaths	10.7 (3 to 19.7)	12 (3.7 to 21.6)	9.5 (2.3 to 19)	11.7 (3.4 to 21.3)	13.4 (4 to 23.8)	10.2 (2.6 to 20.7)	9 (-11.5 to 35.3)	11.8 (-11.5 to 43.2)	7.5 (-20.2 to 41.7)
		DALYs	175.1 (45.8 to 335.7)	207.2 (57.5 to 387.2)	145 (31.6 to 305.6)	203.9 (52.2 to 391.6)	246 (65 to 444)	167 (37.4 to 359)	16.4 (-5.1 to 43.8)	18.7 (-5.5 to 48.7)	15.2 (-13.9 to 50.6)
		YLLs	154.5 (41.1 to 298.5)	179 (51.1 to 335.7)	131.7 (28.6 to 281.2)	176.7 (45.3 to 345.8)	208.3 (56.5 to 383.6)	149.1 (32.5 to 328.6)	14.4 (-9.1 to 45)	16.4 (-11.5 to 50.9)	13.3 (-18.1 to 52.6)
		YLDs	20.5 (4.5 to 42.2)	28.3 (6.9 to 55.5)	13.3 (2.4 to 30.2)	27.1 (6 to 55)	37.7 (9.2 to 73.3)	17.8 (3.1 to 39.9)	32.1 (21.4 to 47.1)	33.4 (21.5 to 50.7)	34.1 (17.9 to 58.4)
	Low temperature	Deaths	4.4 (-1.3 to 10.6)	4.6 (-1.5 to 10.8)	4.3 (-1.2 to 10.4)	3.2 (-1.7 to 8.1)	3.4 (-1.9 to 8.6)	3 (-1.6 to 8)	-27.4 (-120.4 to 41.1)	-25.1 (-121.9 to 46.1)	-29.3 (-119.3 to 39.6)
		YLLs	89.9 (-27.9 to 212)	94.8 (-30.9 to 220.7)	85.1 (-24.4 to 203.1)	59.2 (-32.7 to 151.3)	62.8 (-34.6 to 157.7)	56.1 (-29.9 to 146.8)	-34.1 (-118.1 to 30.3)	-33.7 (-118.8 to 34.8)	-34.1 (-117.6 to 27.9)
	Secondhand smoke	Deaths	6 (4 to 8.5)	6.5 (4.1 to 9.1)	5.5 (3.4 to 8.1)	3.6 (2.4 to 5.3)	4 (2.6 to 5.7)	3.3 (2 to 5.2)	-39.8 (-52.6 to -21.8)	-38.9 (-52.5 to -20.1)	-40.3 (-55.6 to -19.6)
		DALYs	151.7 (101 to 214.1)	174.1 (111.6 to 246.7)	129.1 (82 to 193.5)	89.4 (59.9 to 131.4)	103.4 (67.8 to 149.9)	76.1 (46.7 to 124.1)	-41.1 (-55.2 to -22.1)	-40.6 (-55.2 to -20)	-41 (-57.2 to -20.2)
		YLLs	145.1 (95.8 to 206.2)	165.5 (103.5 to 234)	124.4 (77.8 to 188.8)	82.9 (54.5 to 122.8)	94.7 (60.1 to 140.4)	71.6 (42.8 to 118.4)	-42.9 (-57.2 to -23.2)	-42.8 (-57.7 to -21.4)	-42.4 (-58.9 to -20.8)
		YLDs	6.6 (4.4 to 9)	8.6 (5.7 to 11.8)	4.7 (3.2 to 6.5)	6.5 (4.3 to 8.9)	8.7 (5.8 to 12)	4.5 (3 to 6.2)	-1.7 (-10.9 to 8.6)	0.9 (-10.2 to 13.4)	-4.2 (-17.9 to 12)
	Smoking	Deaths	18.1 (12.4 to 26.1)	6.3 (3.7 to 9.8)	29.1 (19.5 to 42.6)	11.4 (7.7 to 16.8)	3.7 (2.3 to 5.9)	17.8 (11.8 to 27.1)	-37.4 (-54.1 to -15.7)	-40.6 (-63.3 to -2.9)	-38.8 (-55.9 to -15.8)
		DALYs	492.6 (345.6 to 700.5)	180.9 (109.1 to 282.2)	779.9 (531.3 to 1106.6)	306.3 (210.2 to 445.3)	108.5 (66.8 to 169.7)	478.1 (320.7 to 718)	-37.8 (-54.6 to -15.8)	-40 (-63.6 to -1.1)	-38.7 (-55.8 to -14.8)
		YLLs	459.6 (313.7 to 661.2)	165.8 (96.4 to 260.3)	730.2 (484.1 to 1063.9)	272.9 (179.3 to 409.7)	93.4 (54.5 to 151.3)	428.5 (273.2 to 674.4)	-40.6 (-58 to -17.6)	-43.7 (-66.5 to -4.8)	-41.3 (-59 to -15.8)
		YLDs	33.1 (23 to 44)	15.1 (9 to 23.3)	49.7 (34.9 to 65.5)	33.4 (23.1 to 43.8)	15.1 (9 to 22.7)	49.6 (34.6 to 64.7)	1 (-17.1 to 20)	0.1 (-37.7 to 60.3)	-0.3 (-15.3 to 15.6)
Syrian Arab Republic	All risk factors	Deaths	117.7 (94.7 to 144.7)	124.4 (98.7 to 154.2)	111.9 (87.6 to 143.7)	83.2 (64.5 to 105.4)	93.2 (73 to 115.3)	80.2 (60.1 to 106)	-29.3 (-48.7 to -2.3)	-25.1 (-44.9 to 3.2)	-28.4 (-50 to 1.9)
		DALYs	2608 (2128.1 to 3157.7)	2600.4 (2086.5 to 3184.6)	2620.2 (2094.4 to 3255.5)	1708.5 (1346 to 2186.7)	1718.4 (1375.9 to 2146.7)	1759.1 (1353.2 to 2312.8)	-34.5 (-51.5 to -11)	-33.9 (-50.3 to -10.4)	-32.9 (-51.8 to -4.3)
		YLLs	2377.1 (1916.1 to 2927)	2338.6 (1827.8 to 2905.6)	2417.5 (1882.3 to 3060.5)	1505.9 (1154.5 to 1964)	1488 (1164 to 1909.9)	1582.9 (1185.3 to 2130.3)	-36.6 (-54.9 to -11)	-36.4 (-53.9 to -10)	-34.5 (-54.5 to -3.5)
		YLDs	230.8 (168.5 to 291.3)	261.8 (190.7 to 330.8)	202.8 (147.3 to 255.7)	202.5 (148.9 to 257.4)	230.4 (169.7 to 292.3)	176.2 (128.5 to 225.1)	-12.3 (-16.8 to -7.2)	-12 (-18.1 to -4.6)	-13.1 (-19.1 to -6.1)
	Alcohol use	Deaths	1.4 (0.9 to 2.1)	0.2 (0.1 to 0.4)	2.6 (1.6 to 3.8)	0.5 (0.2 to 0.9)	0 (0 to 0.1)	1 (0.4 to 1.7)	-63.1 (-81.5 to -40.4)	-79.6 (-115.6 to -52.5)	-62 (-80.1 to -38.7)
		DALYs	42.4 (26.6 to 61)	6.6 (2.6 to 12)	75.7 (46.2 to 111.1)	14.7 (7.1 to 24.5)	1.4 (-0.1 to 3.4)	28.1 (14.1 to 46.7)	-65.3 (-81 to -46)	-79.2 (-104.6 to -57.5)	-62.8 (-79.3 to -42.1)
		YLLs	40 (24.7 to 58.4)	6.2 (2.5 to 11.2)	71.4 (43.4 to 105.8)	13.7 (6.6 to 23.1)	1.3 (-0.1 to 3.2)	26.1 (13 to 43.9)	-65.9 (-81.6 to -46.1)	-79.2 (-101.3 to -58.3)	-63.5 (-80 to -42)
		YLDs	2.4 (1.4 to 3.6)	0.4 (0.1 to 0.8)	4.3 (2.4 to 6.4)	1.1 (0.4 to 1.8)	0.1 (-0.1 to 0.3)	2.1 (0.9 to 3.5)	-55.8 (-77.2 to -40.3)	-78.8 (-184.5 to -44.4)	-51.6 (-72.8 to -36.4)
	Ambient particulate matter pollution	Deaths	27.3 (19.2 to 37.3)	27.2 (18.8 to 37.8)	27.4 (19.3 to 37.8)	19.9 (14.1 to 26.8)	21.2 (15.2 to 28.2)	19.8 (13.9 to 27.6)	-26.9 (-48.7 to 4.4)	-22.2 (-44.7 to 14.6)	-27.6 (-50.1 to 6.2)
		DALYs	684.5 (484.5 to 918.3)	650.6 (449.1 to 887.8)	716.9 (510.4 to 968)	470.9 (340.7 to 628)	457.5 (333.8 to 609)	495.9 (354.4 to 673.3)	-31.2 (-50.1 to -2.5)	-29.7 (-49.9 to 1.8)	-30.8 (-51.8 to -0.3)
		YLLs	620.9 (440.4 to 845.4)	580.8 (397.9 to 802.2)	659 (464.2 to 901)	410.9 (286.9 to 558.6)	389.6 (274.8 to 528.5)	443.6 (311.8 to 612.9)	-33.8 (-54.2 to -3.3)	-32.9 (-54.2 to 1.5)	-32.7 (-54.4 to 0.4)
		YLDs	63.6 (41.8 to 87.7)	69.9 (44.7 to 97.3)	57.9 (37.9 to 80)	60 (41 to 79)	67.9 (46.4 to 89.5)	52.3 (35.5 to 69.5)	-5.6 (-18.1 to 14.2)	-2.8 (-17.4 to 21.4)	-9.8 (-21.5 to 8.2)

Location	Risk factor	Measure	1990			2019			% Change (1990 to 2019)		
			Both	Female	Male	Both	Female	Male	Both	Female	Male
	Diet high in red meat	Deaths	5.3 (1.9 to 8.3)	5.3 (1.9 to 8.5)	5.2 (1.9 to 8.3)	3 (1.1 to 4.9)	3.1 (1.1 to 5.2)	3 (1.1 to 5)	-43.5 (-60.6 to -19)	-41.5 (-61.1 to -13.1)	-42.1 (-60.5 to -12.3)
		DALYs	148.6 (56.7 to 230.8)	143.1 (54.8 to 224.7)	154.1 (56.5 to 243.8)	78.1 (30.2 to 127.1)	74 (28.5 to 121.3)	84.1 (30.6 to 139.8)	-47.5 (-62.3 to -26.3)	-48.3 (-64.2 to -26.9)	-45.4 (-61.6 to -19.4)
		YLLs	134.3 (48.6 to 212.3)	127.1 (48 to 203.4)	141.3 (51 to 226)	67.5 (24.5 to 111.9)	62.2 (23.6 to 105)	74.9 (26.4 to 125.8)	-49.7 (-64.9 to -26.3)	-51.1 (-67.4 to -27.1)	-47 (-64 to -18.5)
		YLDs	14.3 (5 to 23)	16 (5.5 to 25.8)	12.8 (4.5 to 20.5)	10.5 (3.3 to 17.2)	11.8 (3.6 to 19.7)	9.2 (3 to 15.2)	-26.4 (-38.6 to -17.7)	-26.1 (-38.9 to -15.5)	-27.8 (-40.6 to -16.7)
	Diet high in sodium	Deaths	2.4 (0.4 to 9.9)	1.7 (0.4 to 7)	3.1 (0.3 to 12.4)	1.6 (0.2 to 6.8)	1.2 (0.3 to 4.8)	2.1 (0.2 to 8.8)	-32.5 (-58.1 to 22.2)	-32.6 (-65.6 to 38.6)	-31.2 (-60.4 to 41.7)
		DALYs	61.7 (7.4 to 246.3)	41 (7.7 to 173.1)	80.8 (5.9 to 315.6)	39 (4.9 to 159.4)	25 (5.1 to 107.4)	53.3 (3.9 to 215)	-36.9 (-61.4 to 12.4)	-38.9 (-69.2 to 35.1)	-34.1 (-60.5 to 31.4)
		YLLs	56.4 (6.7 to 224.3)	36.7 (6.9 to 151.7)	74.5 (5.4 to 292.3)	34.5 (4.3 to 141.2)	21.5 (4.3 to 93.1)	47.9 (3.5 to 196.1)	-38.8 (-63.2 to 10.7)	-41.5 (-70.8 to 28.6)	-35.7 (-62.5 to 30.6)
		YLDs	5.3 (0.6 to 21.5)	4.3 (0.7 to 18.6)	6.3 (0.4 to 25.3)	4.5 (0.5 to 17.9)	3.6 (0.6 to 15.1)	5.4 (0.4 to 21)	-16.2 (-48.3 to 49.8)	-16.4 (-61.6 to 83.9)	-14.6 (-43 to 66.3)
	Diet low in fiber	Deaths	3.9 (0.8 to 7.7)	4 (0.8 to 8)	3.8 (0.8 to 7.6)	2.7 (0.6 to 5.5)	3 (0.6 to 6.1)	2.7 (0.6 to 5.5)	-29.3 (-49.4 to 0)	-24.3 (-49.9 to 12.6)	-28.5 (-50.8 to 5)
		DALYs	98.1 (18.5 to 194.2)	94.7 (18.5 to 190.9)	101.4 (19.7 to 203.8)	63.5 (12.8 to 129.6)	61.9 (12.7 to 122.8)	67.6 (13.5 to 137.1)	-35.2 (-52.9 to -9)	-34.7 (-53.9 to -6.8)	-33.4 (-52.3 to -4.1)
		YLLs	88.6 (16.6 to 178.1)	84.2 (16.5 to 171.4)	92.9 (17.8 to 188.1)	55 (10.9 to 113.2)	52.4 (10.9 to 107.2)	60.1 (12 to 123.8)	-37.9 (-56.6 to -10.2)	-37.8 (-58.3 to -7.3)	-35.3 (-55 to -4.2)
		YLDs	9.5 (1.7 to 19.4)	10.5 (1.9 to 21.6)	8.5 (1.5 to 17.5)	8.5 (1.6 to 17.1)	9.5 (1.8 to 19.5)	7.5 (1.5 to 15.3)	-10.4 (-21.1 to 5.1)	-9.7 (-25.1 to 10.8)	-12.2 (-26.3 to 8.9)
	Diet low in fruits	Deaths	5 (2.5 to 8.1)	5.3 (2.5 to 8.6)	4.8 (2.4 to 8)	3.8 (1.8 to 6.6)	4.1 (1.9 to 7.1)	3.8 (1.7 to 6.6)	-24.4 (-48.5 to 12.1)	-23.3 (-50.2 to 14)	-20.9 (-48.5 to 19.3)
		DALYs	136.2 (65.7 to 222.5)	135.8 (65.9 to 219)	137 (67.1 to 227.7)	96.4 (44.4 to 167.8)	93.6 (42.9 to 163.6)	101.9 (47.6 to 178.9)	-29.3 (-51 to 1.7)	-31.1 (-53.2 to -0.3)	-25.6 (-49.7 to 10.1)
		YLLs	124.5 (61.3 to 205)	122.4 (59.4 to 201.4)	126.8 (61.9 to 214.6)	84.4 (40.1 to 148.8)	80 (37.7 to 142.3)	91.7 (43.8 to 162.6)	-32.1 (-54.4 to -0.7)	-34.6 (-57.3 to -3)	-27.7 (-52.4 to 9.4)
		YLDs	11.8 (4.4 to 20.4)	13.5 (5 to 23.8)	10.2 (4 to 17.5)	11.9 (4.3 to 22)	13.6 (4.8 to 25.5)	10.2 (3.9 to 18.7)	1.3 (-16.7 to 21.1)	0.5 (-18.4 to 21.8)	0.6 (-19.6 to 24.9)
	Diet low in vegetables	Deaths	2.1 (0.5 to 4.9)	2.1 (0.5 to 4.8)	2.1 (0.5 to 5.1)	2.5 (0.7 to 4.6)	2.6 (0.8 to 4.8)	2.6 (0.7 to 4.8)	20.3 (-29.3 to 135.7)	23.9 (-26.8 to 157.4)	23.8 (-30.6 to 175.9)
		DALYs	46 (9.1 to 117.9)	43.1 (8.5 to 106.7)	48.7 (9.4 to 129.9)	59.1 (15.6 to 110.4)	55.2 (15.4 to 101.4)	64.6 (16.9 to 123.5)	28.5 (-31.6 to 222.5)	28.3 (-30.9 to 223.7)	32.5 (-32 to 252)
		YLLs	41.8 (8.3 to 111.6)	38.7 (7.7 to 96.5)	44.8 (8.6 to 122.3)	52 (13.6 to 99.8)	47.5 (12.4 to 89.2)	58.2 (14.4 to 113.5)	24.3 (-34.9 to 217.2)	22.7 (-36.2 to 209.1)	29.7 (-34.7 to 255.4)
		YLDs	4.1 (0.7 to 9.1)	4.4 (0.8 to 9.6)	3.9 (0.7 to 8.8)	7.1 (1.9 to 12.8)	7.8 (2.1 to 14)	6.4 (1.8 to 11.6)	71.6 (1.2 to 316.9)	76.6 (1.2 to 333.4)	64.9 (-0.5 to 297.1)
	Diet low in whole grains	Deaths	5.3 (2.8 to 7.7)	6.1 (3.2 to 8.6)	4.7 (2.2 to 7.3)	4.6 (2.5 to 6.4)	5.5 (3.1 to 7.5)	4.2 (2.2 to 6.1)	-14.1 (-38.1 to 18.1)	-9.9 (-34.5 to 24.7)	-10.3 (-42.2 to 31.5)
		DALYs	115.5 (55.7 to 165.1)	125.5 (62.9 to 178.1)	106.8 (46.9 to 162.6)	95.1 (49.6 to 134.5)	102.7 (55.7 to 141.9)	91.5 (45.6 to 132.6)	-17.7 (-39.7 to 9.8)	-18.1 (-38 to 10)	-14.3 (-42.5 to 23.1)
		YLLs	97.1 (46.4 to 141.5)	103.6 (51.5 to 149.4)	91.4 (40.2 to 142.3)	77.1 (39.5 to 112)	81.7 (43.6 to 115.4)	76.4 (37.1 to 114.6)	-20.6 (-45.2 to 11.5)	-21.1 (-43.9 to 13.6)	-16.4 (-47.2 to 27.6)
		YLDs	18.5 (8.2 to 27.8)	21.9 (10.1 to 32.6)	15.3 (6.5 to 23.3)	18 (9 to 26.5)	21 (10.6 to 30.8)	15.1 (7.4 to 22.6)	-2.5 (-8.6 to 9.2)	-4 (-12.7 to 7.8)	-1.6 (-11.2 to 15.8)
	High body-mass index	Deaths	29 (17.1 to 44.1)	33.4 (19.6 to 50)	25.2 (13.4 to 39.1)	22.1 (12.8 to 33.7)	23.4 (14.1 to 34.9)	21.6 (11.8 to 33.9)	-23.9 (-45.7 to 12.4)	-29.8 (-50.6 to 3.8)	-14.3 (-42.7 to 32.4)
		DALYs	933.9 (571.7 to 1368)	1038.9 (654.7 to 1493.5)	839.6 (465.5 to 1267.6)	680.5 (426 to 986.2)	683.1 (447.9 to 967)	688.1 (405.5 to 1042.4)	-27.1 (-46.5 to 5.9)	-34.2 (-51.7 to -4.7)	-18 (-42.7 to 23.5)
		YLLs	847.2 (511 to 1264.6)	928 (573.1 to 1352.9)	775 (428.1 to 1190.4)	590.6 (356.6 to 871.1)	574.6 (365.6 to 822.9)	617.1 (356.8 to 960.2)	-30.3 (-50.6 to 5.5)	-38.1 (-56.5 to -5.5)	-20.4 (-46.2 to 24.2)
		YLDs	86.8 (50.1 to 130.3)	110.9 (66.6 to 161.4)	64.5 (33.7 to 101.1)	89.9 (55.4 to 128.3)	108.4 (68.5 to 152.4)	71 (42.6 to 104.7)	3.6 (-7 to 20.9)	-2.2 (-11.9 to 14.4)	10 (-6.5 to 41.4)
	High fasting plasma glucose	Deaths	26.2 (15.4 to 46.5)	30 (16.4 to 57.5)	22.9 (13.5 to 40.2)	31.1 (17.6 to 55.6)	34.5 (18.7 to 64.5)	30 (17.1 to 52.1)	18.6 (-19.7 to 75.8)	15.1 (-24.2 to 79.5)	31.1 (-15.9 to 110.5)
		DALYs	505.8 (316.9 to 815.3)	547.2 (326.3 to 908.8)	469.3 (297.4 to 756.1)	589.9 (365.6 to 968.3)	599.9 (361.4 to 987.5)	598.6 (366 to 940.6)	16.6 (-19.6 to 70.1)	9.6 (-26.3 to 68.9)	27.5 (-17.3 to 97.9)
		YLLs	461.4 (290.6 to 740)	493.8 (292.3 to 837.1)	433 (275.9 to 690.1)	522.7 (313.6 to 846.7)	523.2 (308.3 to 879.5)	540.1 (324 to 848.6)	13.3 (-23.5 to 70.7)	6 (-29.9 to 68.4)	24.7 (-21.4 to 99.6)
		YLDs	44.4 (24.7 to 76)	53.5 (28.3 to 94)	36.3 (20.8 to 60.9)	67.1 (37.7 to 112.3)	76.6 (42.5 to 128.7)	58.4 (32.4 to 95.7)	51.1 (20.3 to 92.6)	43.4 (9.4 to 91.8)	60.8 (20.7 to 110.4)
	High LDL cholesterol	Deaths	14.6 (5.8 to 29.5)	16.9 (6.2 to 36.3)	12.6 (5.2 to 24.4)	11.9 (4.3 to 24.9)	14.8 (4.7 to 33.3)	10.6 (4.1 to 21.5)	-18.5 (-42.6 to 9.4)	-12.3 (-40.3 to 21)	-16.3 (-46.2 to 20.7)
		DALYs	333.8 (194.7 to 549.9)	364.5 (204.3 to 611.6)	306.5 (177 to 505.3)	259.5 (141.1 to 442)	285.5 (145.4 to 509.2)	246 (137.8 to 425.7)	-22.2 (-43 to 2.5)	-21.7 (-43 to 5.2)	-19.7 (-44.6 to 14.8)
		YLLs	277.8 (155.2 to 471.5)	297.8 (161 to 526.8)	260.5 (144.6 to 439.8)	207.5 (105 to 372.4)	223.8 (104.2 to 422.3)	203.4 (107.9 to 361)	-25.3 (-48.7 to 5.1)	-24.9 (-50 to 8.2)	-21.9 (-49.6 to 19.4)
		YLDs	56 (34.2 to 89.4)	66.7 (40.3 to 106.5)	46.1 (28.3 to 74.3)	52.1 (31.4 to 83.9)	61.7 (37.1 to 98.2)	42.7 (26.1 to 67.8)	-6.9 (-13.3 to -0.3)	-7.5 (-16 to 0.8)	-7.4 (-16.6 to 3.6)
	High systolic blood pressure	Deaths	69.5 (50.8 to 91.6)	76.5 (55.1 to 103.3)	63.3 (45.4 to 86.6)	48 (34.8 to 64.6)	55.1 (39 to 74.6)	45.3 (32.2 to 62.7)	-30.8 (-51.2 to -2.1)	-28 (-51.6 to 3.2)	-28.4 (-51.2 to 4.5)
		DALYs	1564.3 (1202.9 to 1991.1)	1616.2 (1209.6 to 2106.6)	1521.5 (1121.7 to 2007.7)	1021.4 (756.7 to 1342.5)	1041.5 (763.7 to 1370.1)	1041 (765.4 to 1424.1)	-34.7 (-52.5 to -9.3)	-35.6 (-55.2 to -9.3)	-31.6 (-52.1 to -1)
		YLLs	1425.7 (1081.7 to 1851)	1454.4 (1066.5 to 1944.7)	1404 (1017.7 to 1873.7)	900.4 (655.3 to 1219)	902 (650.6 to 1225.3)	937 (673.3 to 1305.1)	-36.8 (-55.8 to -8.8)	-38 (-58.2 to -10.4)	-33.3 (-54.7 to -0.1)
		YLDs	138.5 (94.9 to 184.1)	161.9 (110.4 to 220.1)	117.5 (80.7 to 156.1)	121 (84.5 to 160.9)	139.5 (96.3 to 186.4)	104 (72.2 to 139.9)	-12.7 (-23.6 to -0.2)	-13.8 (-29.9 to 5.6)	-11.4 (-25.2 to 3.5)
	High temperature	Deaths	0.7 (-0.6 to 3)	0.7 (-0.7 to 3)	0.6 (-0.6 to 2.8)	0.7 (-0.4 to 2.2)	0.9 (-0.4 to 2.5)	0.7 (-0.3 to 2.2)	14.1 (-346 to 359.4)	22.7 (-365.3 to 406.8)	14.4 (-346.9 to 344.9)
		YLLs	14 (-12.6 to 64.5)	13.6 (-12.4 to 62)	14.3 (-13.1 to 64.7)	13.5 (-6.3 to 39.9)	13.5 (-6.4 to 39.7)	14.1 (-6.7 to 43.3)	-3.4 (-303.6 to 263.9)	-1.2 (-315.4 to 282.8)	-1.7 (-314.7 to 278.9)
	Household air pollution from solid fuels	Deaths	4.9 (2 to 10.1)	5.9 (2.5 to 11.9)	3.9 (1.5 to 8.7)	0 (0 to 0.1)	0 (0 to 0.1)	0 (0 to 0.1)	-99.3 (-99.7 to -98.2)	-99.2 (-99.7 to -98)	-99.3 (-99.8 to -98.1)
		DALYs	121.1 (50.7 to 251.2)	141.3 (60.4 to 284.8)	102.8 (40.9 to 228.5)	0.8 (0.3 to 1.9)	1 (0.4 to 2.2)	0.7 (0.2 to 1.6)	-99.3 (-99.8 to -98.3)	-99.3 (-99.8 to -98.2)	-99.3 (-99.8 to -98.2)
		YLLs	109.5 (45.7 to 227.9)	126.2 (53.3 to 253.4)	94.5 (36.8 to 208)	0.7 (0.3 to 1.6)	0.8 (0.3 to 1.9)	0.6 (0.2 to 1.4)	-99.4 (-99.8 to -98.3)	-99.3 (-99.8 to -98.3)	-99.4 (-99.8 to -98.2)
		YLDs	11.6 (4.8 to 24.1)	15.2 (6.5 to 30.6)	8.3 (3.2 to 18.8)	0.1 (0 to 0.2)	0.1 (0.1 to 0.3)	0.1 (0 to 0.2)	-99.1 (-99.6 to -97.6)	-99.1 (-99.6 to -97.6)	-99.1 (-99.7 to -97.7)
	Kidney dysfunction	Deaths	11.2 (7.9 to 15.3)	13.2 (9 to 18.5)	9.5 (6.8 to 12.9)	10.4 (6.6 to 14.6)	11.8 (6.8 to 16.7)	9.5 (6.2 to 13.3)	-7.7 (-35.2 to 29.6)	-10.4 (-39.6 to 27.5)	0.2 (-33.7 to 46.9)
		DALYs	244.9 (186.9 to 317.6)	279 (210.2 to 364.3)	214.2 (161.4 to 286.5)	212.3 (151.8 to 282.2)	229.1 (158.5 to 306.4)	200.4 (141.2 to 273.2)	-13.3 (-37.4 to 19.3)	-17.9 (-40.2 to 13.9)	-6.5 (-35.5 to 35.3)
		YLLs	220.7 (165.7 to 292.3)	248.3 (181 to 329.4)	196 (145.9 to 264.4)	185.2 (128.8 to 250.8)	196.2 (131.9 to 267.6)	179 (121.7 to 247.3)	-16.1 (-41.6 to 20.1)	-21 (-45.8 to 14.4)	-8.7 (-39.5 to 36.2)

Location	Risk factor	Measure	1990			2019			% Change (1990 to 2019)		
			Both	Female	Male	Both	Female	Male	Both	Female	Male
		YLDs	24.2 (16.8 to 32.1)	30.7 (20.9 to 41.1)	18.2 (12.4 to 24.4)	27.1 (18.6 to 36.6)	32.9 (22.5 to 44.6)	21.4 (14.7 to 29.1)	11.9 (3.3 to 21)	7.1 (-2.4 to 17.4)	17.2 (4.8 to 30.8)
	Lead exposure	Deaths	8.2 (4.8 to 12.1)	6.9 (3.7 to 10.9)	9.4 (5.7 to 13.9)	5.4 (3.1 to 8.3)	4.9 (2.5 to 7.7)	6 (3.6 to 9.2)	-34.6 (-53.1 to -10.1)	-30 (-49.1 to -2)	-35.7 (-55.8 to -7.3)
		DALYs	193.5 (114 to 284.7)	152.1 (80.1 to 232.1)	231.8 (141.9 to 341)	102.2 (57.3 to 157)	82.7 (41.1 to 133.5)	123 (70.1 to 187.1)	-47.2 (-62.5 to -27.4)	-45.7 (-60.3 to -24.7)	-47 (-63.1 to -24.6)
		YLLs	176.8 (103.5 to 261.9)	136.8 (69.8 to 210.7)	213.9 (129.3 to 319.4)	91.3 (51 to 142.6)	72.5 (36.1 to 118.4)	111.3 (62.8 to 173.9)	-48.4 (-64.4 to -26.7)	-47 (-62.5 to -23.6)	-48 (-64.8 to -23.4)
		YLDs	16.6 (9.4 to 25.4)	15.3 (7.5 to 24.5)	17.9 (10.9 to 26.8)	10.9 (5.6 to 17.5)	10.1 (4.6 to 17.2)	11.7 (6.4 to 18)	-34.5 (-44.3 to -27)	-33.7 (-45.7 to -25.2)	-34.7 (-45.1 to -25.5)
	Low physical activity	Deaths	6.5 (1.4 to 14.1)	7.6 (1.9 to 15.9)	5.6 (1.1 to 12.5)	5.6 (1.3 to 11.4)	7.2 (1.8 to 14.5)	4.8 (0.9 to 10)	-14.7 (-38.6 to 16.2)	-5.6 (-34.4 to 34)	-14 (-43.5 to 25.6)
		DALYs	103.9 (21.9 to 240.2)	116.4 (27.1 to 260.6)	92.7 (15.9 to 223.9)	86.1 (17.5 to 195.8)	101.2 (23.3 to 217)	77.6 (13.5 to 179.6)	-17.1 (-39 to 11.1)	-13.1 (-35.5 to 20)	-16.3 (-43.4 to 20.2)
		YLLs	90.4 (19 to 209.4)	100.3 (23.8 to 224.1)	81.7 (14.6 to 201.1)	73.2 (15.5 to 161.8)	85.8 (20.2 to 181)	67 (11.9 to 155.5)	-19.1 (-43.8 to 13.8)	-14.5 (-39.4 to 24.4)	-17.9 (-47.6 to 23.7)
		YLDs	13.4 (2.4 to 34)	16.1 (3.2 to 39.2)	11 (1.7 to 29.1)	12.9 (2.3 to 32.2)	15.4 (3.1 to 37.5)	10.6 (1.7 to 26.8)	-4 (-12.4 to 9.1)	-3.9 (-14.2 to 12.9)	-4.1 (-15 to 13.2)
	Low temperature	Deaths	10.3 (4.3 to 17.8)	10.9 (4.4 to 19.1)	9.8 (4.1 to 16.8)	6.9 (2.9 to 11.4)	7.8 (3.4 to 13)	6.5 (2.7 to 11.1)	-33.3 (-50.3 to -8.7)	-28.5 (-47 to -1.6)	-32.9 (-52.7 to -4.9)
		YLLs	218.5 (91.7 to 373.7)	213.7 (88.1 to 374.2)	223.4 (93.9 to 375.3)	123.7 (50.9 to 208.1)	123.5 (52.4 to 206.6)	128.9 (52.6 to 219)	-43.4 (-59 to -20.6)	-42.2 (-58 to -18.9)	-42.3 (-59.6 to -17.9)
	Secondhand smoke	Deaths	5.1 (3.5 to 7)	5.9 (4 to 8)	4.4 (2.9 to 6.2)	3.1 (2.1 to 4.3)	3.5 (2.5 to 4.8)	2.7 (1.8 to 4)	-40.2 (-57.3 to -15.3)	-40.3 (-57.7 to -14.8)	-37.5 (-57.6 to -7.4)
		DALYs	134.6 (93.4 to 186.3)	158.7 (109.2 to 219.5)	111.8 (74.1 to 156.9)	76.3 (52.2 to 107)	86.3 (59.6 to 120)	67 (44.5 to 96.9)	-43.3 (-59.6 to -21.5)	-45.6 (-61.5 to -22)	-40 (-58.5 to -12.3)
		YLLs	126.6 (87.2 to 176.7)	147.9 (101.1 to 205.9)	106.5 (70.2 to 152.2)	69.8 (46.7 to 99.4)	77.5 (52.7 to 109.1)	62.9 (41 to 92.4)	-44.9 (-61.4 to -21.6)	-47.6 (-64.4 to -22.4)	-41 (-60 to -11.8)
		YLDs	8 (5.1 to 11.1)	10.8 (6.9 to 15)	5.3 (3.4 to 7.6)	6.5 (4.2 to 9)	8.8 (5.7 to 12.2)	4.2 (2.7 to 5.8)	-17.9 (-24.7 to -10.1)	-18.9 (-26.7 to -9.4)	-21 (-32.8 to -6)
	Smoking	Deaths	23.4 (18.5 to 29.6)	9.9 (6.8 to 13.5)	35.7 (27.8 to 45.8)	11.9 (8.8 to 16.2)	3.9 (2.7 to 5.6)	19.3 (14 to 26.2)	-49 (-65.1 to -24.1)	-60.2 (-74.5 to -35.5)	-45.9 (-62.7 to -18.3)
		DALYs	645.5 (514.1 to 804.2)	281.5 (196.9 to 380.2)	980.8 (775.8 to 1221.8)	319 (236.7 to 425.8)	108.1 (75.8 to 149.5)	525.9 (387.5 to 708.2)	-50.6 (-65.2 to -28.3)	-61.6 (-75.1 to -39.5)	-46.4 (-62.1 to -19.7)
		YLLs	587.5 (461.7 to 747.8)	249.3 (169.8 to 340.2)	899 (690.1 to 1139)	281.8 (204.7 to 387)	90.5 (61.9 to 129.9)	468.9 (337.6 to 646.8)	-52 (-67.5 to -27.3)	-63.7 (-77.3 to -40.4)	-47.8 (-64.3 to -19.1)
		YLDs	58 (41.6 to 74.1)	32.1 (21.3 to 44.7)	81.9 (59.7 to 105.2)	37.3 (26.8 to 48.4)	17.6 (11.7 to 25)	57 (40.4 to 74.5)	-35.7 (-42.9 to -27.9)	-45.2 (-60.1 to -24.8)	-30.4 (-37.3 to -22.2)
Tunisia	All risk factors	Deaths	85.1 (69.7 to 102.6)	82.3 (65.2 to 102.2)	88.1 (69.6 to 107.8)	65.8 (48.1 to 85.4)	61.3 (45.4 to 80.1)	70.9 (50.4 to 94.2)	-22.7 (-43.5 to 3)	-25.5 (-46 to 0.2)	-19.6 (-43.7 to 9.7)
		DALYs	1597.9 (1317.3 to 1901)	1576 (1286.5 to 1913)	1620.7 (1298 to 1991.7)	1263 (963 to 1624.7)	1199.8 (921.8 to 1525.2)	1331.2 (987 to 1741.2)	-21 (-41.6 to 5.3)	-23.9 (-42.3 to 1.3)	-17.9 (-40.8 to 11.6)
		YLLs	1461.5 (1179 to 1759.6)	1420 (1129.6 to 1756.7)	1503.2 (1175.9 to 1860.4)	1092.5 (794.7 to 1437.7)	1004.5 (740.3 to 1311.7)	1187 (850.5 to 1596)	-25.2 (-46.5 to 2.4)	-29.3 (-48.6 to -2.4)	-21 (-45.5 to 10.6)
		YLDs	136.4 (99.7 to 173)	156 (114.6 to 199.2)	117.6 (85.1 to 148.8)	170.6 (124.7 to 216.1)	195.4 (141.3 to 248.9)	144.2 (104.6 to 183.5)	25 (17.4 to 33.2)	25.2 (14.8 to 36.5)	22.6 (12.2 to 34)
	Alcohol use	Deaths	0.3 (0 to 0.7)	-0.1 (-0.3 to 0.1)	0.7 (0.1 to 1.4)	0.4 (0.1 to 0.8)	-0.1 (-0.3 to 0.1)	1 (0.4 to 1.8)	39.4 (-225.4 to 801.6)	-12.5 (-494.6 to 604.8)	40.3 (-33.3 to 449.6)
		DALYs	11.6 (3.3 to 21.2)	-0.3 (-5.3 to 6)	23.2 (8 to 40.1)	15 (6.7 to 24.8)	0.2 (-5.2 to 6.5)	30.6 (15.2 to 49.2)	29.4 (-29.7 to 211.4)	-177.1 (-1194.9 to 1085)	31.7 (-22.2 to 176.4)
		YLLs	11.4 (3.4 to 20.4)	0.3 (-4.3 to 6.1)	22.1 (7.5 to 38.5)	13.7 (6.2 to 22.9)	0.6 (-4 to 5.7)	27.5 (13.7 to 45.4)	20.9 (-34 to 183.6)	112.6 (-817.8 to 920)	24.5 (-27.7 to 161.7)
		YLDs	0.3 (-0.4 to 1)	-0.6 (-1.2 to 0.1)	1.1 (0 to 2.3)	1.3 (0.2 to 2.5)	-0.4 (-1.4 to 0.7)	3 (1.2 to 5)	401.9 (-2280.8 to 4328.8)	-40.5 (-508.4 to 462.2)	176.9 (44.4 to 1462.5)
	Ambient particulate matter pollution	Deaths	16.7 (10.7 to 23.9)	15.6 (9.7 to 22.4)	17.8 (11.1 to 25.5)	15.4 (10.1 to 21.9)	14.4 (9.6 to 20)	16.4 (10.6 to 24.1)	-7.8 (-34 to 31.8)	-7.6 (-34.4 to 36.6)	-7.7 (-36.3 to 36.3)
		DALYs	354.3 (231.4 to 499.6)	338.1 (215.4 to 484.8)	370 (236.7 to 524.8)	336.9 (232.4 to 471.7)	323.2 (224.3 to 444)	351.4 (238.5 to 501.2)	-4.9 (-31.2 to 34.7)	-4.4 (-31 to 38.9)	-5 (-32.4 to 39.1)
		YLLs	322 (206.6 to 460.1)	302.3 (189.4 to 436.2)	341.1 (212.4 to 488.6)	287.6 (190.7 to 413.2)	266.2 (175.4 to 378.8)	310.2 (205.1 to 457.5)	-10.7 (-37.8 to 30.9)	-11.9 (-39.2 to 32.4)	-9.1 (-38.1 to 37.7)
		YLDs	32.3 (19.6 to 47.2)	35.8 (21.3 to 53.3)	28.9 (17.2 to 41.8)	49.3 (32.3 to 69.4)	57 (37.3 to 80)	41.2 (27.1 to 57.9)	52.6 (31.4 to 94.2)	59 (32.6 to 109.1)	42.6 (23.7 to 79.3)
	Diet high in red meat	Deaths	2.9 (1 to 4.7)	2.9 (1 to 4.7)	3 (1.1 to 5)	2 (0.6 to 3.6)	1.9 (0.6 to 3.3)	2.2 (0.7 to 3.9)	-30.6 (-58.6 to 4.5)	-34 (-61.3 to 3.4)	-27 (-57.9 to 14.9)
		DALYs	68.8 (24.1 to 109.7)	69.1 (24.6 to 111.2)	68.4 (24.3 to 111.4)	49 (16.6 to 83.4)	46.9 (15.8 to 79.9)	51.3 (16.8 to 90.8)	-28.7 (-56.4 to 10)	-32.1 (-58.6 to 6.3)	-25.1 (-55.2 to 17.4)
		YLLs	62 (21 to 100.7)	61.3 (20.7 to 101.1)	62.8 (21.5 to 102.9)	41.4 (14.1 to 72.3)	38.1 (13.4 to 66.7)	44.9 (15.2 to 81.9)	-33.3 (-59.6 to 7.7)	-37.9 (-62.5 to 3.3)	-28.5 (-58.9 to 17.9)
		YLDs	6.7 (1.9 to 11.2)	7.8 (2.2 to 13.1)	5.7 (1.6 to 9.6)	7.7 (2 to 13)	8.9 (2.3 to 15.1)	6.4 (1.6 to 10.8)	13.9 (-1.5 to 26.7)	13.6 (-6 to 30.2)	12.7 (-4.3 to 28.6)
	Diet high in sodium	Deaths	1.7 (0.3 to 6.7)	1.2 (0.3 to 4.7)	2.2 (0.2 to 9)	1.2 (0.2 to 4.9)	0.8 (0.2 to 3.3)	1.7 (0.2 to 6.5)	-27 (-57 to 37.2)	-31 (-66.2 to 44.3)	-22.8 (-59.2 to 65.6)
		DALYs	35.8 (4.6 to 145.9)	25 (4.7 to 102.8)	46.3 (3.8 to 192.2)	27 (3.6 to 109.9)	17.9 (3.6 to 76.7)	36.7 (3 to 146.2)	-24.7 (-53.9 to 40.3)	-28.4 (-67.3 to 56.5)	-20.7 (-52.7 to 59.1)
		YLLs	32.8 (4.2 to 133.7)	22.4 (4.3 to 93.3)	42.9 (3.5 to 176.9)	23.4 (3 to 92.9)	14.8 (3 to 62.9)	32.5 (2.6 to 129.8)	-28.9 (-58.4 to 35.4)	-34 (-69.9 to 45.5)	-24.2 (-55.6 to 58.1)
		YLDs	3 (0.4 to 12.1)	2.5 (0.4 to 10.3)	3.5 (0.3 to 13.8)	3.6 (0.5 to 14.4)	3.1 (0.6 to 12.9)	4.2 (0.3 to 16.5)	21.2 (-27.7 to 121.7)	22.1 (-44.3 to 169.1)	22.2 (-18.7 to 123)
	Diet low in fiber	Deaths	1.2 (0.3 to 2.6)	1.2 (0.3 to 2.5)	1.3 (0.3 to 2.7)	0.6 (0.2 to 1.3)	0.5 (0.2 to 1.2)	0.7 (0.2 to 1.5)	-52.5 (-68.6 to -23.6)	-54.4 (-73.8 to -21.5)	-50.3 (-69 to -15.2)
		DALYs	23.6 (5.7 to 50.3)	22.9 (5.5 to 50)	24.3 (5.9 to 51)	11.4 (3.6 to 25.3)	10.5 (3.3 to 22.9)	12.4 (3.6 to 28.5)	-51.7 (-65.7 to -26.7)	-54.3 (-69.2 to -27)	-49 (-66.2 to -17.6)
		YLLs	21.3 (5.2 to 45.6)	20.3 (4.9 to 44.9)	22.4 (5.4 to 47.1)	9.7 (3 to 21.8)	8.6 (2.8 to 19)	10.9 (3.2 to 25.1)	-54.5 (-69.1 to -28.8)	-57.7 (-72.7 to -30.3)	-51.1 (-68.6 to -19)
		YLDs	2.2 (0.5 to 5)	2.5 (0.6 to 5.9)	2 (0.5 to 4.4)	1.7 (0.5 to 3.8)	1.9 (0.5 to 4.5)	1.5 (0.4 to 3.5)	-25.6 (-40.2 to 3.7)	-26.6 (-45.5 to 4.1)	-25.1 (-44.2 to 6)
	Diet low in fruits	Deaths	4.5 (2 to 7.8)	4.5 (2 to 7.8)	4.5 (2 to 7.9)	2.2 (0.9 to 4)	2.1 (0.8 to 3.8)	2.3 (0.9 to 4.2)	-51.7 (-70.4 to -26.4)	-53.4 (-72.2 to -28)	-49.9 (-71.8 to -19.3)
		DALYs	100.2 (45 to 174.1)	102.6 (46.2 to 178.8)	97.8 (44 to 174.3)	51.6 (20.9 to 89.7)	51 (20.3 to 90.4)	52.1 (21 to 92.2)	-48.5 (-67.7 to -23)	-50.3 (-69.4 to -25.9)	-46.7 (-67.8 to -17.2)
		YLLs	91.5 (41.4 to 161)	92.4 (41.5 to 161)	90.7 (41.2 to 163.7)	43.8 (18.2 to 77.4)	41.8 (16.8 to 75.9)	46 (19 to 81.7)	-52.1 (-70.7 to -25.3)	-54.7 (-72.7 to -29.6)	-49.3 (-70.3 to -18.2)
		YLDs	8.7 (2.9 to 16.4)	10.2 (3.5 to 19.4)	7.1 (2.3 to 13.6)	7.7 (2.5 to 14.7)	9.2 (3 to 17.3)	6.2 (2 to 12.1)	-10.9 (-29.8 to 8.6)	-10.4 (-31.1 to 10)	-13.3 (-34 to 9.5)
	Diet low in vegetables	Deaths	1.9 (0.6 to 3.8)	1.8 (0.5 to 3.6)	2.1 (0.6 to 4)	0.5 (0.2 to 1.1)	0.5 (0.2 to 1)	0.6 (0.2 to 1.3)	-71.8 (-84.7 to -43.1)	-73.8 (-86.9 to -44.9)	-69.5 (-85 to -38.7)
		DALYs	39.4 (10.1 to 80.1)	37.7 (9.5 to 78.6)	41 (10.3 to 83.9)	9 (3.2 to 19.6)	7.9 (3 to 16.8)	10.3 (3.2 to 22.4)	-77.1 (-87.6 to -49.3)	-79.1 (-89.1 to -51.5)	-75 (-87.6 to -45.7)

Location	Risk factor	Measure	1990			2019			% Change (1990 to 2019)		
			Both	Female	Male	Both	Female	Male	Both	Female	Male
		YLLs	35.9 (9 to 75)	33.8 (8.6 to 71.7)	38 (9.3 to 78.9)	7.9 (2.7 to 16.8)	6.7 (2.5 to 14.3)	9.2 (2.8 to 20.4)	-78 (-88.4 to -49.8)	-80.3 (-89.9 to -52.1)	-75.7 (-88.4 to -45.5)
	Diet low in whole grains	YLDs	3.5 (0.7 to 6.7)	3.9 (0.8 to 7.6)	3.1 (0.7 to 5.8)	1.1 (0.4 to 2.5)	1.2 (0.4 to 2.7)	1.1 (0.3 to 2.5)	-67.5 (-82 to -37)	-69.4 (-83.3 to -35.3)	-65.6 (-82.8 to -34.8)
		Deaths	4.4 (2.3 to 6.2)	4.5 (2.5 to 6.4)	4.3 (2.1 to 6.2)	4.3 (2.1 to 6.4)	4.1 (2 to 6.1)	4.5 (2.1 to 7)	-2.5 (-31.3 to 29.4)	-8.9 (-36.1 to 24.6)	4.9 (-29.4 to 49)
		DALYs	79.7 (39.4 to 113.5)	82.1 (42 to 117.3)	77.5 (36.3 to 112.5)	84.9 (40 to 125.7)	82.8 (39.3 to 122.5)	87.1 (38.9 to 135.7)	6.5 (-20.9 to 38.6)	0.9 (-25.5 to 31.9)	12.4 (-20 to 56)
		YLLs	67.7 (34.1 to 96.9)	68 (35.5 to 100.2)	67.4 (31.6 to 98.9)	70 (33.2 to 107)	65.5 (30.9 to 99.1)	74.7 (33.4 to 118.5)	3.4 (-27.4 to 41.7)	-3.6 (-33.9 to 33.5)	10.8 (-26 to 60.3)
	High body-mass index	YLDs	12.1 (5.5 to 18)	14.1 (6.6 to 21.3)	10.1 (4.4 to 15.3)	14.9 (6.5 to 22.4)	17.3 (7.6 to 26.2)	12.4 (5.3 to 18.5)	23.6 (13.4 to 33.5)	22.2 (7.5 to 36.3)	23 (7.8 to 39.4)
		Deaths	16.8 (9.5 to 25.5)	19.4 (11.6 to 29.1)	14.3 (7.2 to 23.2)	15.6 (8.8 to 24.7)	15.9 (9.3 to 24.6)	15.2 (7.9 to 25.3)	-7.1 (-35.8 to 34.9)	-18.2 (-43.5 to 18.1)	6.3 (-31.4 to 69.4)
		DALYs	481.6 (297.4 to 694.6)	561.7 (365.2 to 796.1)	405.9 (220.9 to 630.9)	450 (280.8 to 668)	466.7 (300.3 to 674)	432 (247.5 to 667.5)	-6.6 (-32.8 to 31.4)	-16.9 (-39.9 to 13.5)	6.4 (-29.5 to 63.9)
		YLLs	435.4 (267.6 to 635.2)	501.2 (324.6 to 719)	373.3 (202.2 to 585)	377.6 (224.5 to 578.9)	377.6 (231.3 to 567.5)	377 (206.6 to 596.7)	-13.3 (-40.5 to 26.6)	-24.7 (-48.2 to 8.3)	1 (-35.8 to 61.6)
	High fasting plasma glucose	YLDs	46.2 (27.1 to 69.3)	60.5 (37 to 89.5)	32.6 (17.3 to 51.4)	72.3 (45.4 to 105.2)	89.1 (56.5 to 127.4)	55 (32.6 to 81.8)	56.5 (38.2 to 85.2)	47.2 (28.9 to 72.2)	68.6 (41.2 to 124.5)
		Deaths	24.6 (13.3 to 47.8)	20.7 (10.4 to 42.3)	28.8 (15.6 to 54.1)	25.9 (13.6 to 48.4)	24 (12.3 to 46)	28 (14.3 to 53)	4.9 (-28.2 to 52.7)	15.8 (-25.9 to 77.1)	-2.8 (-36.7 to 45.2)
		DALYs	401.2 (240.1 to 694.5)	337.5 (187.6 to 609.4)	461.8 (270 to 764.1)	470.3 (273.5 to 802.5)	438.1 (249.1 to 768.9)	504.6 (288.5 to 875.3)	17.2 (-18.8 to 69.6)	29.8 (-15.6 to 96.3)	9.3 (-27.8 to 65.9)
		YLLs	370.6 (219 to 642.4)	307.1 (168.7 to 558.4)	431.2 (250.1 to 709.8)	410.1 (241 to 704)	370.9 (208.5 to 648.8)	451.8 (254.3 to 784.2)	10.6 (-25.8 to 63.7)	20.8 (-23.2 to 87.9)	4.8 (-33 to 60.7)
	High LDL cholesterol	YLDs	30.6 (16.2 to 54.4)	30.4 (16.2 to 56.4)	30.6 (15.9 to 54.6)	60.3 (32.4 to 102.3)	67.2 (35.2 to 117.9)	52.8 (28.6 to 87.5)	96.8 (58.8 to 146.2)	121 (60.8 to 206.9)	72.2 (33.9 to 126.8)
		Deaths	11.9 (3.6 to 26.4)	11.9 (3.5 to 26.9)	12 (3.8 to 27.7)	12.6 (3.9 to 28.5)	12 (3.8 to 26.9)	13.2 (4.2 to 30.1)	5.6 (-23.6 to 45.4)	0.9 (-27.6 to 39.1)	10.6 (-22.6 to 58.6)
		DALYs	219.1 (106.6 to 398.9)	221.7 (107 to 409.1)	217 (105.7 to 407.9)	254.4 (128.5 to 469.8)	248 (128.7 to 452.5)	261.2 (128 to 484.3)	16.1 (-12.4 to 56)	11.9 (-15.3 to 50.6)	20.3 (-13.9 to 71.2)
		YLLs	183.8 (82.9 to 355.4)	180.6 (79.1 to 353.1)	187.2 (81.6 to 370.3)	207.7 (96.3 to 401.4)	193.9 (85 to 371)	222.3 (102.2 to 433)	13 (-21.1 to 60.2)	7.3 (-24.3 to 56.1)	18.7 (-20.1 to 78.3)
	High systolic blood pressure	YLDs	35.4 (20.6 to 57.4)	41 (23.9 to 66.3)	29.8 (17.1 to 49.1)	46.7 (27.9 to 75.9)	54.1 (32 to 88.2)	38.9 (22.6 to 62.6)	32.1 (21.9 to 43.1)	31.8 (19.2 to 47.1)	30.4 (15.9 to 48)
		Deaths	46.9 (34.1 to 61)	49.5 (35.5 to 67.3)	44.2 (30.6 to 59.2)	35.5 (23.8 to 49.7)	34.9 (22.8 to 49.3)	36 (23.1 to 52)	-24.2 (-46.5 to 5.4)	-29.4 (-53.3 to 3.5)	-18.5 (-45.3 to 18.3)
		DALYs	925.2 (706.1 to 1166.4)	990.4 (738.3 to 1280.2)	863.9 (626.4 to 1119.1)	723.1 (510.8 to 965.7)	721.3 (508.5 to 957)	723.9 (490.4 to 1007.1)	-21.8 (-42.9 to 6.4)	-27.2 (-49.6 to 1.5)	-16.2 (-43.4 to 20.8)
		YLLs	845.9 (633.3 to 1075.2)	893.3 (660.7 to 1165.5)	801.4 (568.5 to 1051.9)	622.9 (427.1 to 853.7)	602.1 (409.4 to 826.1)	643.8 (422.5 to 920.1)	-26.4 (-47.8 to 3.8)	-32.6 (-55.7 to -2.9)	-19.7 (-47.6 to 19.9)
	High temperature	YLDs	79.3 (55 to 106)	97.1 (67 to 132.8)	62.5 (42 to 84.8)	100.2 (68.5 to 132.3)	119.2 (78.8 to 159.4)	80.1 (55.4 to 106.3)	26.4 (9.7 to 46.5)	22.7 (-0.9 to 52.6)	28.3 (8.9 to 53.4)
		Deaths	0.9 (-0.7 to 4.6)	0.8 (-0.7 to 4.5)	0.9 (-0.7 to 4.7)	0.8 (-0.3 to 2.7)	0.7 (-0.3 to 2.7)	0.8 (-0.3 to 2.9)	-9.6 (-292.5 to 252.9)	-13.1 (-278.5 to 240.7)	-5.9 (-311.9 to 263.4)
		YLLs	14.5 (-11.7 to 77.3)	14.3 (-11 to 76.9)	14.7 (-12 to 79.4)	12.4 (-4.5 to 44)	11.6 (-4.1 to 42.5)	13.3 (-5.1 to 47.4)	-14.3 (-289.3 to 235.2)	-19.2 (-267.9 to 211.6)	-9.2 (-308.6 to 250.2)
	Household air pollution from solid fuels	Deaths	5.3 (2.5 to 9.5)	6.1 (2.9 to 10.6)	4.6 (2 to 8.7)	0 (0 to 0.1)	0.1 (0 to 0.1)	0 (0 to 0.1)	-99.1 (-99.7 to -97.7)	-99.1 (-99.7 to -97.8)	-99.2 (-99.7 to -97.7)
		DALYs	113 (53.4 to 200.9)	131.7 (63.4 to 227.3)	95.1 (41.1 to 183.5)	1 (0.3 to 2.3)	1.2 (0.4 to 2.7)	0.8 (0.3 to 1.9)	-99.1 (-99.6 to -97.7)	-99.1 (-99.6 to -97.7)	-99.1 (-99.7 to -97.6)
		YLLs	102.4 (47.9 to 183.9)	117.7 (56.5 to 205.5)	87.7 (37.9 to 171.3)	0.9 (0.3 to 1.9)	1 (0.3 to 2.2)	0.7 (0.2 to 1.7)	-99.2 (-99.7 to -97.8)	-99.2 (-99.7 to -97.9)	-99.2 (-99.7 to -97.6)
	Kidney dysfunction	YLDs	10.6 (4.9 to 19)	13.9 (6.6 to 24.2)	7.4 (3.2 to 13.9)	0.2 (0.1 to 0.3)	0.2 (0.1 to 0.5)	0.1 (0 to 0.2)	-98.6 (-99.4 to -96.5)	-98.5 (-99.4 to -96.5)	-98.7 (-99.5 to -96.8)
		Deaths	7.8 (5.1 to 10.8)	8.3 (5.4 to 11.8)	7.2 (4.7 to 10)	8.4 (5 to 12.4)	8.2 (4.7 to 12.3)	8.7 (5 to 13.1)	8.9 (-24.4 to 48.7)	-1.4 (-32.3 to 37.6)	20.6 (-21.8 to 70.3)
		DALYs	148.2 (111.3 to 192.6)	162.5 (118.8 to 215.1)	135 (98 to 176.5)	169.5 (117.6 to 232.5)	170.4 (117.8 to 232.5)	168.6 (112.6 to 236.4)	14.4 (-17.3 to 53.7)	4.9 (-23.8 to 41.2)	24.9 (-14.1 to 73.1)
		YLLs	134.2 (99.2 to 176.8)	144.9 (103.9 to 196.5)	124.3 (88.3 to 165)	145.5 (96.2 to 203.5)	141.6 (93.3 to 196.9)	149.8 (95.7 to 213.7)	8.4 (-24.9 to 51.8)	-2.3 (-31.9 to 37.7)	20.4 (-20.6 to 72.2)
	Lead exposure	YLDs	14 (9.5 to 19)	17.6 (11.9 to 23.8)	10.6 (7.2 to 14.6)	24 (16.4 to 32.6)	28.9 (19.6 to 39)	18.9 (12.8 to 25.8)	71.5 (57.5 to 86.6)	63.8 (49.4 to 80.4)	77.4 (57.8 to 99.3)
		Deaths	5.1 (2.9 to 7.7)	4.2 (2.1 to 6.6)	6.1 (3.6 to 9)	3.4 (1.8 to 5.5)	2.6 (1.2 to 4.5)	4.3 (2.3 to 6.9)	-33.2 (-53.4 to -9.6)	-36.7 (-55.9 to -13.1)	-29.1 (-52.6 to -0.4)
		DALYs	102.3 (59.2 to 151.7)	83.6 (41.2 to 132.2)	120.4 (75.1 to 176.1)	60.6 (29.7 to 97.9)	46.9 (19.8 to 80.4)	75.5 (39.3 to 120.5)	-40.8 (-59.1 to -20)	-43.9 (-61.5 to -24.6)	-37.3 (-57.5 to -12.4)
		YLLs	93.9 (54 to 140.6)	75.5 (37.1 to 120.4)	111.8 (68.9 to 164.5)	53.3 (26.3 to 86.7)	39.9 (16.7 to 69.4)	67.9 (34.1 to 110.1)	-43.2 (-61.5 to -21.3)	-47.2 (-64.9 to -26.1)	-39.2 (-59.9 to -12.7)
	Low physical activity	YLDs	8.4 (4.4 to 13.1)	8.1 (3.7 to 13.4)	8.6 (5 to 13)	7.3 (3.3 to 12.2)	7 (2.7 to 12.5)	7.6 (3.8 to 12.2)	-13 (-29.2 to -1.3)	-13.6 (-34.8 to 0.7)	-12.2 (-28 to 1.6)
		Deaths	2.5 (0.4 to 7)	2 (0.4 to 5.8)	3.1 (0.4 to 9)	2.8 (0.5 to 7.7)	2.2 (0.4 to 6.4)	3.6 (0.5 to 9.9)	13.2 (-18.6 to 57.2)	8.2 (-25.3 to 58.7)	18.1 (-20.7 to 76.9)
		DALYs	34.1 (5.5 to 98)	28.4 (5.4 to 84.8)	40.3 (5.5 to 120.4)	40.6 (6.1 to 115.4)	32.8 (5.6 to 96.7)	49.9 (6.8 to 143.1)	19.1 (-13 to 59.9)	15.5 (-15.8 to 59.5)	23.8 (-14.1 to 78.6)
		YLLs	30.4 (5 to 86.4)	24.7 (4.7 to 73.4)	36.6 (4.8 to 110.3)	35.4 (5.4 to 100.8)	27.5 (4.7 to 82.3)	44.6 (6.1 to 128.2)	16.2 (-19.2 to 60.9)	11.2 (-23.5 to 57.4)	21.9 (-17.8 to 80.6)
	Low temperature	YLDs	3.7 (0.5 to 11.9)	3.7 (0.6 to 12.3)	3.7 (0.4 to 11.2)	5.3 (0.7 to 16.2)	5.3 (0.8 to 16.9)	5.2 (0.6 to 15.4)	43.3 (24.5 to 72.8)	44.7 (22.7 to 82)	42.3 (17.5 to 82.2)
		Deaths	6.1 (1.9 to 11.1)	6 (1.8 to 11)	6.3 (1.9 to 11.5)	4.8 (1.2 to 9)	4.5 (1.1 to 8.4)	5.1 (1.3 to 9.9)	-22.1 (-47.5 to 3.2)	-25.1 (-50.2 to 1.6)	-18.9 (-47.1 to 11.2)
		YLLs	103.2 (31.1 to 186)	101.7 (30.3 to 185.1)	104.9 (31.9 to 192.5)	76.3 (19 to 147.7)	70.9 (17.2 to 133.9)	82.1 (20.4 to 161.4)	-26.1 (-51 to 0.2)	-30.3 (-54.3 to -5.3)	-21.8 (-49.5 to 9.7)
	Secondhand smoke	Deaths	4.6 (3.2 to 6)	5.2 (3.6 to 7)	3.9 (2.7 to 5.3)	2.8 (1.8 to 4)	3 (1.9 to 4.3)	2.6 (1.6 to 3.8)	-38.9 (-56 to -16.1)	-42.6 (-58.8 to -19.7)	-34.7 (-55.6 to -4.6)
		DALYs	98.8 (70.4 to 130.1)	119.5 (84.2 to 158.9)	78.5 (54.4 to 107)	62.1 (41.4 to 87.8)	71 (47 to 101.3)	52.8 (34.5 to 78.6)	-37.1 (-53.9 to -14.5)	-40.6 (-56.6 to -19.1)	-32.7 (-53.9 to -1.8)
		YLLs	93 (66.5 to 123.4)	111.4 (78.1 to 149.9)	74.9 (51.4 to 102.2)	56 (36.3 to 81.5)	62.6 (40.2 to 90.6)	49.1 (31.4 to 73.8)	-39.8 (-57.1 to -15.8)	-43.9 (-60.7 to -21.3)	-34.5 (-56.4 to -2.5)
	Smoking	YLDs	5.8 (3.8 to 8)	8.1 (5.3 to 11.1)	3.6 (2.3 to 4.9)	6.1 (4 to 8.4)	8.4 (5.5 to 11.7)	3.7 (2.4 to 5.2)	5.3 (-4.3 to 14.6)	4 (-7.1 to 15.7)	3.6 (-11.6 to 22)
		Deaths	14.4 (11.4 to 17.7)	3.5 (2.4 to 5.1)	25 (19.3 to 31)	9.3 (6.7 to 12.4)	1.8 (1.2 to 2.7)	17.7 (12.4 to 23.6)	-35.2 (-55.7 to -8.2)	-47.9 (-68 to -16.9)	-29.4 (-51.8 to 0.2)
		DALYs	330.4 (262 to 404.4)	87.4 (60.1 to 123.6)	560.8 (441.4 to 686.8)	222.5 (164.3 to 293.1)	47.8 (32.7 to 67.5)	407.9 (298.7 to 539.4)	-32.7 (-53 to -6.1)	-45.3 (-65.1 to -13.7)	-27.3 (-49.6 to 2.5)
		YLLs	302.8 (233.9 to 375.5)	77.9 (52.9 to 111.2)	516.1 (395 to 639.5)	194 (137.5 to 263.7)	39.1 (25.6 to 56.9)	358.8 (251.3 to 487.3)	-35.9 (-56.9 to -8.1)	-49.8 (-69.4 to -20.5)	-30.5 (-53.8 to 0.4)

Location	Risk factor	Measure	1990			2019			% Change (1990 to 2019)		
			Both	Female	Male	Both	Female	Male	Both	Female	Male
		YLDs	27.6 (19.7 to 35)	9.5 (6.1 to 13.8)	44.7 (32 to 58)	28.5 (20.6 to 36.4)	8.7 (5.6 to 12.5)	49.1 (35.4 to 63.2)	3.3 (-7.8 to 16)	-8.1 (-36 to 32.1)	9.9 (-0.9 to 23.2)
Turkey	All risk factors	Deaths	59.4 (46.8 to 78.8)	58.6 (43.9 to 75.1)	59.7 (47.5 to 90.8)	49.9 (39.8 to 61.1)	49.2 (38.4 to 60.7)	50.1 (39.9 to 62)	-15.9 (-39.3 to 7.7)	-16 (-39.5 to 11.2)	-16 (-46.1 to 14.9)
		DALYs	1263.7 (1058 to 1637.7)	1249.9 (1012.3 to 1533.6)	1271.1 (1029.4 to 1959.3)	991 (816.3 to 1187.1)	956.3 (787 to 1141.2)	1020 (827.5 to 1248.5)	-21.6 (-42.2 to -2.1)	-23.5 (-41.5 to -4.3)	-19.8 (-47.7 to 9.2)
		YLLs	1088.1 (888.8 to 1459.4)	1033 (798.9 to 1294)	1140.1 (900.6 to 1832.1)	829.3 (662.6 to 1019.9)	770.2 (606.6 to 948.1)	885.4 (697.5 to 1107.6)	-23.8 (-46.2 to -1.3)	-25.4 (-46.8 to -1.8)	-22.3 (-51.5 to 10.1)
		YLDs	175.6 (129.4 to 221.9)	216.9 (158.8 to 274.9)	130.9 (95.9 to 167.4)	161.7 (118.7 to 204.4)	186.1 (136.1 to 237.3)	134.6 (98.3 to 170.7)	-7.9 (-13.1 to -2.6)	-14.2 (-20.5 to -7.4)	2.8 (-5.2 to 11.3)
	Alcohol use	Deaths	0.5 (0.2 to 1)	0.1 (-0.2 to 0.5)	1 (0.5 to 1.8)	0.4 (0.1 to 0.8)	0 (-0.2 to 0.4)	0.8 (0.4 to 1.4)	-23.3 (-66.3 to 55.2)	-59.1 (-636.5 to 331)	-17.8 (-55.4 to 39)
		DALYs	18.7 (8.7 to 30.7)	5.5 (-3.3 to 15.4)	32.5 (17 to 53.7)	13.8 (6 to 22.7)	2.8 (-3.5 to 10.3)	25.5 (13.3 to 40.6)	-26.1 (-59 to 29.8)	-49.3 (-310.5 to 294.4)	-21.3 (-54.3 to 26.6)
		YLLs	17.6 (8.9 to 28.9)	5.7 (-1.7 to 14.4)	30 (15.7 to 50.5)	12.5 (5.7 to 20.5)	3 (-2 to 9.1)	22.7 (11.8 to 36.7)	-28.7 (-60.5 to 23.5)	-46.9 (-218.4 to 138.4)	-24.4 (-57.6 to 25.3)
		YLDs	1.1 (-0.1 to 2.3)	-0.2 (-1.9 to 1.5)	2.5 (1.1 to 4.1)	1.3 (0.1 to 2.7)	-0.2 (-1.7 to 1.5)	2.9 (1.2 to 4.8)	16.3 (-151.3 to 249.1)	13.2 (-576.3 to 701.8)	15.9 (-22.2 to 74.1)
	Ambient particulate matter pollution	Deaths	10.6 (7.6 to 14.8)	9.9 (6.5 to 13.3)	11.4 (8.3 to 17.3)	10.3 (7.7 to 13.5)	10 (7.4 to 12.9)	10.6 (7.9 to 14.1)	-2.8 (-32.8 to 34.3)	1.1 (-28.7 to 47.9)	-7.1 (-40.1 to 33.9)
		DALYs	260.5 (190.6 to 348)	243.8 (168.1 to 320)	277.4 (202.5 to 424.6)	236.5 (182 to 300.9)	224.5 (172.9 to 283.2)	247.6 (188.1 to 321.8)	-9.2 (-34.9 to 22.8)	-7.9 (-32.6 to 30.4)	-10.8 (-41.9 to 27.4)
		YLLs	222.1 (161.8 to 302.2)	197.8 (132.4 to 267.2)	247.2 (177.1 to 397)	194.3 (146 to 255.2)	175.8 (130.4 to 227)	212.6 (156.1 to 285.1)	-12.5 (-40.2 to 22)	-11.1 (-38.5 to 29.1)	-14 (-46.3 to 27.7)
		YLDs	38.4 (25.1 to 52.2)	46 (29.8 to 63.4)	30.3 (20.2 to 40.8)	42.1 (29.4 to 55.5)	48.7 (34 to 64.4)	35 (24.4 to 46)	9.8 (-5.7 to 39.8)	5.9 (-10.9 to 38.3)	15.6 (-1.3 to 43.6)
	Diet high in red meat	Deaths	2.2 (0.8 to 3.5)	2.1 (0.7 to 3.5)	2.3 (0.8 to 3.8)	1.7 (0.6 to 2.8)	1.6 (0.6 to 2.7)	1.8 (0.7 to 3)	-22.8 (-45.9 to 4.7)	-24.1 (-49.2 to 6.4)	-21.7 (-50.2 to 11.8)
		DALYs	59.7 (21.2 to 93.5)	58.4 (20.2 to 92.8)	60.9 (20.8 to 99.2)	43.3 (16.1 to 68.8)	40.4 (15.2 to 64.6)	46 (17.5 to 74.1)	-27.6 (-47.8 to -6.2)	-30.8 (-50.1 to -8.8)	-24.5 (-51 to 4.6)
		YLLs	50.3 (17.1 to 80.3)	46.7 (15.8 to 76.6)	53.9 (18 to 89.5)	34.9 (12.8 to 56.5)	30.7 (11.3 to 50.4)	39.1 (14.5 to 64)	-30.7 (-52.6 to -5.2)	-34.2 (-55.4 to -8)	-27.6 (-54.9 to 5)
		YLDs	9.4 (3.2 to 15.4)	11.7 (4 to 19.2)	7 (2.3 to 11.5)	8.4 (2.7 to 13.7)	9.7 (3.1 to 16)	6.9 (2.3 to 11.4)	-10.9 (-19.5 to -1.5)	-16.9 (-28.3 to -4.4)	-0.6 (-12.6 to 13)
	Diet high in sodium	Deaths	0.8 (0.2 to 3.7)	0.6 (0.2 to 2.4)	1.1 (0.1 to 5)	0.7 (0.2 to 2.8)	0.5 (0.2 to 1.8)	0.9 (0.1 to 4.1)	-21.2 (-54.8 to 42.4)	-21.5 (-60.7 to 61.4)	-20.8 (-61.7 to 76.7)
		DALYs	20.4 (4.1 to 88)	14.3 (4.3 to 58.9)	26.8 (2.9 to 121)	15.2 (3.2 to 65)	10.2 (3.4 to 38.9)	20.5 (2.4 to 94.3)	-25.5 (-57.5 to 36.6)	-28.5 (-64.2 to 55.3)	-23.4 (-63.9 to 77)
		YLLs	17.8 (3.5 to 76.9)	11.8 (3.5 to 48.4)	24.1 (2.6 to 108.9)	12.8 (2.6 to 55)	8.2 (2.6 to 31.2)	17.8 (2 to 82.9)	-27.9 (-59.7 to 35.2)	-30.5 (-66.7 to 50.5)	-26.2 (-65.9 to 75.7)
		YLDs	2.6 (0.5 to 10.8)	2.5 (0.7 to 10)	2.7 (0.3 to 12.1)	2.4 (0.5 to 10.1)	2 (0.6 to 7.9)	2.7 (0.3 to 12.6)	-8.6 (-46.9 to 55.5)	-18.9 (-57.3 to 74.7)	1.6 (-48.4 to 101.1)
	Diet low in fiber	Deaths	0.4 (0.2 to 0.7)	0.3 (0.1 to 0.7)	0.4 (0.2 to 0.8)	0.3 (0.1 to 0.6)	0.3 (0.1 to 0.7)	0.3 (0.1 to 0.6)	-15.5 (-43.5 to 28)	-10 (-48.7 to 58.1)	-22.2 (-53.9 to 27.5)
		DALYs	7.2 (3.2 to 15.5)	6.8 (3.1 to 14.2)	7.5 (3.3 to 17.7)	5.6 (2.5 to 11.5)	5.4 (2.5 to 11.1)	5.7 (2.5 to 12.1)	-22.4 (-44.4 to 7.3)	-21 (-46.8 to 15.3)	-24.3 (-52.5 to 12.5)
		YLLs	6.1 (2.7 to 13.2)	5.5 (2.5 to 11.6)	6.7 (2.9 to 15.5)	4.6 (2.1 to 9.4)	4.3 (1.9 to 8.9)	4.9 (2.2 to 10.5)	-24.8 (-48.4 to 7.4)	-23.1 (-50 to 17.6)	-27 (-57 to 12.5)
		YLDs	1.1 (0.4 to 2.3)	1.3 (0.5 to 2.7)	0.9 (0.3 to 2)	1 (0.4 to 2.1)	1.1 (0.5 to 2.4)	0.8 (0.3 to 1.9)	-8.7 (-24.9 to 14)	-11.8 (-34.1 to 18.1)	-4.3 (-29.4 to 29)
	Diet low in fruits	Deaths	1.4 (0.8 to 2.3)	1.5 (0.8 to 2.5)	1.3 (0.7 to 2.3)	0.6 (0.3 to 1)	0.6 (0.3 to 1.1)	0.5 (0.2 to 0.9)	-61 (-76.3 to -39)	-60 (-79 to -30.2)	-62.5 (-80.1 to -32.8)
		DALYs	38.6 (20.5 to 60.4)	40.9 (21.7 to 64.9)	36 (18.8 to 58.2)	13.8 (7.4 to 22.9)	14.6 (7.7 to 24.9)	12.8 (6.2 to 21.1)	-64.1 (-75.5 to -49.2)	-64.2 (-77 to -45.9)	-64.4 (-79.5 to -44.4)
		YLLs	32.7 (17.8 to 52.3)	33.1 (17.9 to 52.9)	32 (16.5 to 52.5)	11 (5.8 to 18.1)	11 (5.6 to 19.2)	10.8 (5.2 to 18.5)	-66.3 (-77.7 to -50.3)	-66.7 (-79.9 to -46.5)	-66.2 (-81.5 to -45)
		YLDs	5.9 (2.4 to 10.3)	7.7 (3.2 to 13.3)	4 (1.7 to 7)	2.8 (1.2 to 4.9)	3.6 (1.5 to 6.7)	2 (0.9 to 3.8)	-52.4 (-64.5 to -39.7)	-53.6 (-68.2 to -37.6)	-49.9 (-65.9 to -31.3)
	Diet low in vegetables	Deaths	0.2 (0.1 to 0.2)	0.2 (0.1 to 0.2)	0.2 (0.1 to 0.3)	0.1 (0.1 to 0.2)	0.1 (0.1 to 0.2)	0.1 (0.1 to 0.2)	-23.3 (-48.4 to 4.9)	-21.5 (-49.3 to 10.8)	-25.9 (-55.5 to 8.5)
		DALYs	3.1 (2.6 to 4.1)	3.1 (2.5 to 4)	3.2 (2.5 to 4.8)	2.4 (1.9 to 2.8)	2.3 (1.9 to 2.8)	2.4 (1.9 to 2.9)	-25.1 (-44.8 to -4.3)	-25.6 (-46 to -4.1)	-25 (-52.4 to 4.6)
		YLLs	2.7 (2.2 to 3.7)	2.6 (2 to 3.4)	2.9 (2.2 to 4.5)	2 (1.6 to 2.4)	1.9 (1.5 to 2.3)	2.1 (1.7 to 2.6)	-27.2 (-48.3 to -3.1)	-27.4 (-50.2 to -1.2)	-27.4 (-55.4 to 5.9)
		YLDs	0.4 (0.3 to 0.5)	0.5 (0.4 to 0.7)	0.3 (0.2 to 0.4)	0.4 (0.3 to 0.5)	0.4 (0.3 to 0.5)	0.3 (0.2 to 0.4)	-12.1 (-22.5 to -4.9)	-16.8 (-26.9 to -8.8)	-4.5 (-20.2 to 7.5)
	Diet low in whole grains	Deaths	3.3 (2 to 4.7)	3.3 (1.9 to 4.7)	3.3 (1.9 to 5)	3.1 (1.9 to 4.4)	3.2 (1.9 to 4.4)	3.1 (1.8 to 4.3)	-4.7 (-34 to 24.8)	-3 (-32.3 to 34.9)	-6.9 (-44.5 to 30.8)
		DALYs	66.3 (36.7 to 92.6)	67.5 (37.4 to 96.1)	64.8 (35.8 to 99.4)	60 (34.1 to 82.5)	60.5 (34.3 to 82.8)	59.1 (32.8 to 82)	-9.5 (-33.7 to 11.7)	-10.4 (-31.2 to 12.2)	-8.9 (-43.8 to 22.9)
		YLLs	50.6 (28.5 to 74.6)	48.2 (27 to 70)	53 (29.2 to 86.6)	45.2 (25.9 to 64.6)	43.3 (24.9 to 61.5)	46.9 (26 to 67.9)	-10.6 (-40.4 to 17.6)	-10 (-37 to 24.4)	-11.4 (-50.4 to 28.2)
		YLDs	15.7 (8.1 to 22.8)	19.3 (10 to 28)	11.9 (6 to 17.5)	14.8 (7.7 to 21.5)	17.2 (8.9 to 25.3)	12.2 (6.3 to 17.7)	-6.1 (-12.3 to 0.7)	-11.1 (-19.1 to -2.4)	2.6 (-7.7 to 13.9)
	High body-mass index	Deaths	14.8 (8.7 to 22)	16.3 (10 to 23.8)	12.9 (7 to 21.5)	14.1 (8.6 to 20.7)	14 (8.7 to 20.5)	14 (8.2 to 20.9)	-4.3 (-33.3 to 28.6)	-13.7 (-38.4 to 17.2)	8 (-35.1 to 64.1)
		DALYs	464 (299.2 to 658)	519.2 (347.5 to 706.5)	403.5 (233.3 to 645.4)	413 (275.9 to 564.1)	409.8 (283.7 to 547.1)	412.4 (262.4 to 586.4)	-11 (-35.2 to 14.4)	-21.1 (-39.2 to 0.2)	2.2 (-36 to 49.2)
		YLLs	392.2 (245.7 to 559)	419.2 (275.8 to 591.7)	360.9 (204.6 to 591.6)	334.5 (216.2 to 470.8)	313.6 (208.3 to 436)	352.7 (217 to 512.4)	-14.7 (-41.3 to 14.3)	-25.2 (-46.9 to 0.6)	-2.3 (-41.4 to 48)
		YLDs	71.9 (44.5 to 106)	100.1 (62.9 to 142.8)	42.6 (23.3 to 66.9)	78.5 (50.1 to 110.6)	96.3 (62.6 to 133.7)	59.7 (35.8 to 85.9)	9.2 (-1.6 to 23.9)	-3.8 (-13.5 to 9.3)	40.1 (17.2 to 81.3)
	High fasting plasma glucose	Deaths	10.5 (6.2 to 19.3)	11 (6.1 to 21.6)	9.9 (5.9 to 17.7)	14.4 (7.7 to 26.7)	14.3 (7.4 to 27.2)	14.4 (7.5 to 27.5)	37.4 (-10.7 to 99.2)	31 (-17.8 to 107.2)	46.6 (-14 to 122.1)
		DALYs	206.4 (132.6 to 344.3)	214.1 (135.1 to 356.8)	195.8 (122.9 to 330.1)	259 (153.1 to 425.8)	255.5 (147.4 to 418.4)	261.1 (154.4 to 447.9)	25.4 (-15.5 to 76.3)	19.4 (-20.8 to 77)	33.3 (-18.1 to 103.3)
		YLLs	178.3 (113.5 to 298.4)	178.6 (108.8 to 299.4)	175.9 (109.7 to 297.4)	220.3 (127.8 to 364)	211.1 (119.1 to 356.4)	229 (130.5 to 391.5)	23.6 (-19.6 to 78.1)	18.2 (-25.3 to 82.5)	30.2 (-23.4 to 102.2)
		YLDs	28.2 (16.6 to 46)	35.5 (20.3 to 58.2)	20 (11.7 to 31.9)	38.7 (21.6 to 65.5)	44.4 (24.7 to 74.9)	32.1 (17.6 to 56.5)	37.4 (10.3 to 76.7)	25.2 (-3.5 to 71.1)	60.8 (22.9 to 121.7)
	High LDL cholesterol	Deaths	7.7 (2.4 to 17.5)	7.9 (2.2 to 18.2)	7.3 (2.4 to 16.2)	6.6 (1.8 to 15.8)	7.1 (1.9 to 17.1)	5.9 (1.8 to 13.2)	-13.7 (-43.1 to 12.3)	-9.7 (-39 to 23.3)	-19.3 (-51.7 to 11.5)
		DALYs	164.3 (86.1 to 289.7)	172.2 (89.5 to 314.5)	154.5 (79.1 to 282.4)	133.3 (65.4 to 251.6)	142.7 (68.8 to 277.6)	121.4 (61.5 to 224.7)	-18.9 (-41.5 to 0.4)	-17.2 (-36.6 to 3)	-21.4 (-51.1 to 4.8)
		YLLs	121.8 (55.4 to 228.4)	118.6 (51.7 to 242.1)	123.8 (58.7 to 237.3)	96.7 (40.7 to 196.6)	97.8 (37.5 to 208.8)	93.6 (42.2 to 183.1)	-20.6 (-48.3 to 4.8)	-17.5 (-46.2 to 13.7)	-24.4 (-57.3 to 9.7)
		YLDs	42.5 (25.4 to 69.1)	53.7 (32.2 to 86.1)	30.7 (18.3 to 50.5)	36.6 (21.6 to 59.5)	44.8 (26.7 to 74.6)	27.8 (16.3 to 45.3)	-13.9 (-21.2 to -6.4)	-16.5 (-25.6 to -7)	-9.4 (-20.7 to 2.3)

Location	Risk factor	Measure	1990			2019			% Change (1990 to 2019)		
			Both	Female	Male	Both	Female	Male	Both	Female	Male
	High systolic blood pressure	Deaths	37 (27.9 to 50.1)	38.2 (27.3 to 51.2)	35.2 (25.5 to 52.1)	29.8 (22 to 38.6)	30.3 (22 to 40.1)	28.7 (21.2 to 37.5)	-19.5 (-41.7 to 3.7)	-20.7 (-42.9 to 8.5)	-18.3 (-47.3 to 15)
		DALYs	807.5 (636.1 to 1066.8)	824.9 (628 to 1064.3)	780.1 (595.4 to 1214.6)	614.5 (476.4 to 763.7)	602.8 (466.9 to 758.1)	618.3 (467.7 to 783.5)	-23.9 (-44.4 to -4.9)	-26.9 (-44.8 to -5.4)	-20.7 (-48.4 to 9.6)
		YLLs	695.8 (540.5 to 965.6)	683.3 (503.2 to 905.6)	701.1 (521.1 to 1132.9)	515.2 (389.9 to 653.9)	487.7 (360.9 to 626.1)	537.1 (395 to 693.6)	-26 (-48.2 to -4)	-28.6 (-49.1 to -3.3)	-23.4 (-52 to 10.6)
		YLDs	111.7 (77.5 to 145.7)	141.5 (97.7 to 185.6)	79 (55.3 to 105.3)	99.3 (69.5 to 131)	115.1 (79.3 to 153.5)	81.2 (57.2 to 107.7)	-11.1 (-19.4 to -1.5)	-18.7 (-29.1 to -7.2)	2.8 (-9.7 to 16.8)
	High temperature	Deaths	0 (-0.1 to 0.1)	0 (-0.1 to 0.1)	0 (-0.1 to 0.1)	0 (-0.1 to 0.1)	0 (-0.1 to 0.1)	0 (-0.1 to 0.1)	51.1 (-363.7 to 270.1)	52.1 (-361.6 to 288)	49.4 (-355.6 to 281.5)
		YLLs	0.2 (-1 to 1.5)	0.2 (-1 to 1.4)	0.2 (-1.1 to 1.6)	0.3 (-0.9 to 1.7)	0.3 (-0.9 to 1.6)	0.3 (-1 to 1.8)	34.5 (-322.8 to 234.2)	32.4 (-322.4 to 233.7)	36 (-328.8 to 250.3)
	Household air pollution from solid fuels	Deaths	3.6 (1.8 to 6.6)	4.1 (2 to 7.1)	3.1 (1.3 to 6.3)	0.1 (0 to 0.2)	0.1 (0 to 0.2)	0 (0 to 0.1)	-98.3 (-99.4 to -95.8)	-98.3 (-99.4 to -95.6)	-98.4 (-99.5 to -95.8)
		DALYs	88.6 (44.6 to 159.2)	100.3 (51 to 175.6)	75.2 (33 to 148)	1.4 (0.4 to 3.6)	1.6 (0.5 to 3.9)	1.1 (0.3 to 3)	-98.4 (-99.4 to -96.1)	-98.4 (-99.5 to -96)	-98.5 (-99.5 to -96)
		YLLs	74.9 (36.7 to 136.3)	81.4 (41.2 to 143.2)	67 (29.1 to 133.4)	1.1 (0.4 to 2.9)	1.2 (0.4 to 3)	1 (0.3 to 2.5)	-98.5 (-99.5 to -96.3)	-98.5 (-99.5 to -96.1)	-98.5 (-99.5 to -96.1)
		YLDs	13.7 (6.4 to 24.7)	18.9 (9.1 to 32.6)	8.2 (3.5 to 15.7)	0.3 (0.1 to 0.7)	0.3 (0.1 to 0.9)	0.2 (0 to 0.4)	-98.1 (-99.3 to -95.5)	-98.2 (-99.3 to -95.6)	-98 (-99.3 to -95.1)
	Kidney dysfunction	Deaths	4.8 (3.2 to 6.8)	4.9 (3.2 to 6.9)	4.6 (3.1 to 7.2)	6.1 (3.7 to 8.7)	6.1 (3.6 to 9)	6 (3.7 to 8.5)	27.6 (-13.1 to 68.5)	24.5 (-16.5 to 74)	31.5 (-18.9 to 84.1)
		DALYs	102.6 (78.3 to 138.2)	106.8 (78.6 to 140.2)	97.4 (72.5 to 153)	121.6 (86.7 to 160.1)	121.2 (85.5 to 161)	121.1 (87.1 to 159.7)	18.5 (-14.6 to 52)	13.5 (-16.5 to 49)	24.4 (-19.5 to 70.2)
		YLLs	86.6 (64.1 to 119)	86.4 (60.6 to 117.8)	86.3 (62.6 to 140.7)	100.7 (70.7 to 135.9)	96.9 (65.6 to 132.2)	104.3 (73.1 to 139.2)	16.3 (-20.6 to 55.7)	12.2 (-23.5 to 56.6)	20.8 (-26.5 to 72.6)
		YLDs	15.9 (10.9 to 21.4)	20.4 (14 to 27.4)	11.1 (7.6 to 14.9)	20.8 (14 to 28.4)	24.4 (16.4 to 33.2)	16.9 (11.4 to 22.8)	30.8 (17.9 to 43.8)	19.3 (6.3 to 32.8)	52.7 (34.1 to 72.5)
	Lead exposure	Deaths	1.9 (0.7 to 3.4)	1.7 (0.5 to 3.2)	2.2 (0.8 to 3.7)	1.3 (0.4 to 2.5)	1.1 (0.3 to 2.2)	1.5 (0.5 to 2.8)	-31.3 (-53.6 to -11.7)	-32.2 (-54.4 to -7.5)	-30 (-56 to -2.4)
		DALYs	40.8 (12.9 to 75.2)	34.9 (8.3 to 67.3)	47 (16.1 to 83.6)	22.7 (5.5 to 45.3)	18.6 (3.4 to 39.4)	27.2 (7.8 to 52.1)	-44.4 (-64.6 to -27.8)	-46.5 (-66.9 to -29.7)	-42.2 (-64.7 to -20.4)
		YLLs	35.6 (11.2 to 65.3)	29.1 (6.9 to 56.1)	42.4 (14.4 to 75.5)	19.6 (4.9 to 39.3)	15.6 (2.9 to 32.9)	24 (7 to 46.3)	-45 (-65.4 to -26.6)	-46.2 (-66.9 to -26.6)	-43.4 (-67 to -19.5)
		YLDs	5.2 (1.4 to 10.1)	5.8 (1.2 to 11.7)	4.6 (1.5 to 8.4)	3.1 (0.6 to 6.8)	3 (0.4 to 7.1)	3.2 (0.8 to 6.5)	-40.5 (-62.1 to -29.8)	-47.8 (-72.2 to -36)	-30.7 (-53.9 to -18)
	Low physical activity	Deaths	2.9 (0.6 to 6.8)	2.8 (0.6 to 6.5)	3.1 (0.6 to 7)	3.2 (0.7 to 7)	3.2 (0.7 to 6.8)	3.2 (0.6 to 7.1)	8.4 (-23.7 to 50.9)	12.7 (-21.8 to 73.1)	3 (-33.2 to 52)
		DALYs	43.7 (8.1 to 106.5)	43 (8.3 to 104.6)	44.6 (7.7 to 109.1)	46.4 (9.1 to 110.3)	46.3 (10.6 to 109.5)	46.2 (8.6 to 111)	6.1 (-24 to 42.7)	7.8 (-20.2 to 52.3)	3.6 (-32.4 to 47.1)
		YLLs	35.6 (6.6 to 85.3)	33.4 (6.7 to 81.1)	38.1 (6.5 to 93.8)	37.6 (7.6 to 88.9)	36.5 (8.1 to 84)	38.7 (7.2 to 90.8)	5.7 (-28.8 to 49.4)	9.1 (-25.1 to 66.8)	1.7 (-36.6 to 53.2)
		YLDs	8.1 (1.4 to 21.9)	9.5 (1.7 to 25.7)	6.5 (1 to 17.4)	8.7 (1.6 to 22.7)	9.8 (1.9 to 25.4)	7.5 (1.2 to 19.9)	7.6 (-7.1 to 28.6)	3 (-11.9 to 27.2)	14.8 (-3.2 to 40.3)
	Low temperature	Deaths	8.5 (5.8 to 11.8)	8.4 (5.8 to 11.8)	8.6 (5.8 to 12.6)	6.7 (4.6 to 9)	6.6 (4.5 to 9)	6.6 (4.6 to 9)	-22 (-43.7 to 0.1)	-21.8 (-43.1 to 2.9)	-22.4 (-49.2 to 6.2)
		YLLs	154.1 (106.3 to 212.6)	147.4 (101.5 to 204)	160.4 (109.4 to 250)	106.6 (72.6 to 145.2)	100 (68.1 to 136.3)	112.8 (76.3 to 155)	-30.8 (-50.9 to -11.1)	-32.2 (-50.4 to -11.8)	-29.7 (-55.1 to -1.2)
	Secondhand smoke	Deaths	3.3 (2.3 to 4.5)	4 (2.7 to 5.4)	2.5 (1.7 to 3.9)	1.9 (1.3 to 2.5)	2.1 (1.5 to 2.8)	1.6 (1.1 to 2.2)	-43.8 (-59.5 to -26.2)	-47.3 (-63.4 to -29.8)	-38 (-61.7 to -12.1)
		DALYs	76.1 (54.4 to 101.1)	94.9 (68.3 to 125.7)	55.9 (37.4 to 83.4)	40.8 (29 to 54.9)	46.7 (33.1 to 61.4)	34.3 (23 to 48.1)	-46.4 (-60 to -30.8)	-50.8 (-63.7 to -36.5)	-38.7 (-61.8 to -14.8)
		YLLs	68.4 (48.3 to 90.9)	83.3 (58.8 to 112)	52.3 (35 to 80.7)	35.9 (24.9 to 48.9)	39.8 (27.6 to 54.1)	31.4 (20.8 to 44.4)	-47.6 (-62 to -30.7)	-52.2 (-66.2 to -36.2)	-40 (-63.6 to -14.9)
		YLDs	7.7 (5 to 10.6)	11.7 (7.6 to 16)	3.5 (2.3 to 4.9)	4.9 (3.2 to 6.9)	6.9 (4.5 to 9.8)	2.9 (1.9 to 4.1)	-35.6 (-41.1 to -29.6)	-40.4 (-46.1 to -33.7)	-18.9 (-31.1 to -5.4)
	Smoking	Deaths	12.3 (9.9 to 17.9)	6.6 (4.7 to 9)	19 (14.8 to 29.8)	7.4 (5.9 to 9.2)	3.8 (2.9 to 4.8)	11.7 (9.2 to 14.8)	-39.8 (-59.9 to -18.7)	-42.8 (-61.4 to -16.5)	-38.2 (-61.4 to -11.7)
		DALYs	360.6 (294 to 504.2)	224.7 (169.8 to 291.5)	509 (402.9 to 789.4)	215.4 (175.1 to 260.6)	127.8 (100 to 161.1)	312.8 (249.5 to 385.2)	-40.3 (-59 to -21.3)	-43.1 (-58.2 to -23.2)	-38.5 (-61.3 to -14.6)
		YLLs	310.2 (250 to 452.4)	177.9 (131.5 to 238.4)	454.1 (349.6 to 734.8)	176.9 (139.9 to 221.6)	94.9 (71.8 to 124)	267.7 (207.7 to 340.9)	-43 (-63 to -21.7)	-46.7 (-63.4 to -23.8)	-41 (-64.1 to -14.3)
		YLDs	50.4 (36.3 to 64.4)	46.7 (32.1 to 62)	54.9 (39.6 to 70.6)	38.5 (27.6 to 49.4)	32.9 (22.8 to 43.7)	45.1 (32.4 to 57.9)	-23.5 (-31.9 to -14.4)	-29.6 (-43 to -13.4)	-17.9 (-26 to -9.1)
United Arab Emirates	All risk factors	Deaths	159.1 (125.3 to 220.2)	169.7 (129.4 to 228.9)	152.5 (110.7 to 213.6)	79.2 (59.8 to 103.4)	76.9 (58.5 to 102.4)	80.3 (59 to 105.4)	-50.2 (-62.3 to -35.4)	-54.6 (-66.2 to -37)	-47.3 (-61.8 to -26.6)
		DALYs	3155.9 (2501.8 to 4229.8)	3385.5 (2661.6 to 4488.6)	3050.5 (2259 to 4213.7)	1719.6 (1350.3 to 2192.7)	1739.9 (1392.7 to 2175)	1717.7 (1304.2 to 2220.6)	-45.5 (-58.5 to -29.9)	-48.6 (-60.8 to -31)	-43.7 (-59.1 to -22.8)
		YLLs	2822.9 (2184.7 to 3920.6)	2980.4 (2277.7 to 4025.4)	2758 (1966.7 to 3903.6)	1422.4 (1057.8 to 1910.2)	1364.1 (1037.5 to 1792.5)	1450.7 (1035.6 to 1952.3)	-49.6 (-63.2 to -32.4)	-54.2 (-67 to -35)	-47.4 (-63.1 to -24.5)
		YLDs	333 (242.7 to 424.7)	405.2 (296.6 to 518.7)	292.6 (213.1 to 374.1)	297.2 (218.7 to 377.1)	375.8 (277.2 to 476.3)	267.1 (194.6 to 342)	-10.8 (-15.8 to -6.1)	-7.2 (-13.4 to -0.8)	-8.7 (-15.3 to -2)
	Alcohol use	Deaths	3.4 (1.6 to 5.7)	0.1 (-1.1 to 1.3)	5.6 (2.8 to 9.5)	0.3 (-0.4 to 1)	-0.4 (-0.8 to 0)	0.5 (-0.4 to 1.6)	-91.6 (-119.8 to -72.4)	-835.8 (-1088.1 to 994.9)	-90.4 (-110.4 to -75.1)
		DALYs	97.9 (51.4 to 160.5)	12.3 (-17.1 to 47.3)	147 (79.6 to 240.7)	12.1 (-4.9 to 31.8)	-10.1 (-19.7 to 0.2)	19.7 (-3.3 to 46.6)	-87.6 (-108.7 to -68.6)	-182.3 (-1160.8 to 903)	-86.6 (-103.2 to -70.4)
		YLLs	91.5 (48.2 to 152.7)	13.7 (-13 to 43.9)	136.3 (72.8 to 224.6)	11.5 (-3 to 28.8)	-6.8 (-14.9 to 0.9)	17.7 (-1.7 to 40.8)	-87.4 (-104.8 to -69.1)	-149.9 (-1031.3 to 684)	-87 (-101.6 to -70.9)
		YLDs	6.4 (2.3 to 11.3)	-1.4 (-5.1 to 2.3)	10.7 (4.7 to 17.5)	0.6 (-2.4 to 3.9)	-3.3 (-6 to -0.7)	2 (-1.9 to 6.3)	-90.4 (-184.9 to -53.5)	124.8 (-1198.6 to 1650.2)	-81.7 (-130.6 to -53.4)
	Ambient particulate matter pollution	Deaths	46.7 (34 to 69.4)	49.6 (35.7 to 70.2)	45.1 (30.5 to 67.3)	23.5 (16.3 to 32.9)	22.8 (16.4 to 31.5)	23.9 (15.9 to 34.1)	-49.6 (-62.2 to -33.6)	-54.1 (-66.4 to -36.1)	-46.9 (-61.8 to -24.8)
		DALYs	1038.6 (763.5 to 1456.5)	1123.9 (824 to 1554.3)	1004.4 (697.8 to 1484.9)	581.5 (421.7 to 780.3)	594.6 (440.2 to 783.5)	579.5 (406.7 to 794.7)	-44 (-58 to -26.5)	-47.1 (-60.4 to -28)	-42.3 (-58.7 to -19.5)
		YLLs	923.6 (660.1 to 1331)	982.2 (708.8 to 1379.3)	903.1 (604 to 1373.5)	477 (328.3 to 674.5)	460.7 (326.2 to 632.8)	485.5 (322.2 to 693)	-48.4 (-62.8 to -28.9)	-53.1 (-66.4 to -32.2)	-46.2 (-63 to -21.2)
		YLDs	115 (81.7 to 154.1)	141.7 (100.7 to 189.4)	101.3 (71.8 to 136.2)	104.5 (74.2 to 139.7)	133.9 (95.2 to 180.5)	94 (67.4 to 126.5)	-9.1 (-15 to -3.6)	-5.5 (-12.4 to 1.9)	-7.2 (-14.6 to 0.2)
	Diet high in red meat	Deaths	14.1 (8.3 to 20.9)	14.9 (8.6 to 22.5)	13.7 (7.7 to 20.8)	4.1 (1.7 to 6.4)	3.9 (1.6 to 6.2)	4.1 (1.8 to 6.8)	-71.2 (-83.4 to -58.2)	-73.7 (-84.9 to -60.2)	-70 (-83.4 to -53.6)
		DALYs	358.3 (217.6 to 526.5)	393.9 (243.5 to 574.5)	345.9 (200.7 to 515.6)	112 (49.7 to 173.2)	117.2 (51.8 to 182)	110.8 (48.9 to 177.1)	-68.7 (-82.3 to -54)	-70.3 (-83.1 to -56.1)	-68 (-82.1 to -51.2)
		YLLs	316.3 (188.5 to 472.4)	341.1 (204.7 to 508.9)	308.8 (176.2 to 469.9)	91.3 (41.2 to 146.6)	90 (40.7 to 142.6)	92.2 (41.8 to 154.8)	-71.1 (-84 to -55.8)	-73.6 (-84.9 to -59.2)	-70.1 (-84.2 to -51.9)
		YLDs	42 (24.2 to 59.9)	52.8 (30.4 to 75.8)	37 (21.3 to 52.9)	20.7 (7.8 to 32.8)	27.2 (10 to 43.1)	18.6 (6.8 to 29.2)	-50.6 (-72.3 to -37.8)	-48.6 (-71.6 to -35.4)	-49.7 (-72 to -35.4)
	Diet high in [illegible]	Deaths	3.3 (0.4 to 14)	2.2 (0.5 to 9.3)	4 (0.3 to 17.1)	1.7 (0.2 to 6.6)	0.9 (0.2 to 3.8)	2 (0.2 to 8)	-48.6 (-72.3 to -5.5)	-57.4 (-80.2 to -12.9)	-50.3 (-72.4 to 13.7)

Location	Risk factor	Measure	1990			2019			% Change (1990 to 2019)		
			Both	Female	Male	Both	Female	Male	Both	Female	Male
	sodium	DALYs	76.2 (8.2 to 313.8)	51.2 (9.3 to 221.5)	91.6 (6.2 to 384.2)	42.7 (4.5 to 164.6)	24.9 (5 to 100.9)	49.4 (3.9 to 194)	-44 (-68.5 to 0)	-51.4 (-79.1 to 6)	-46 (-67.1 to 17.8)
		YLLs	68.2 (7.4 to 285.6)	44.9 (8.3 to 195.5)	82.7 (5.5 to 345.9)	35.4 (3.7 to 138.7)	19.3 (3.8 to 78.8)	41.5 (3.2 to 165)	-48.2 (-71.8 to -5.2)	-57 (-81.5 to -5)	-49.8 (-70.5 to 11.7)
		YLDs	8 (0.8 to 31.6)	6.4 (1.1 to 26.2)	8.9 (0.6 to 36.1)	7.3 (0.7 to 28.1)	5.6 (1 to 22.8)	7.9 (0.6 to 31)	-8.8 (-46.5 to 48.8)	-12 (-63.8 to 99.4)	-11.3 (-42.7 to 76.4)
	Diet low in fiber	Deaths	2.1 (0.5 to 4.8)	2.2 (0.6 to 5.3)	2 (0.5 to 4.7)	1.3 (0.3 to 3.1)	1.3 (0.3 to 3)	1.4 (0.3 to 3.1)	-35.5 (-55.4 to -4.8)	-41.4 (-62.2 to -6.6)	-32 (-56.4 to 7.3)
		DALYs	43.2 (10.7 to 104.6)	47.2 (10.8 to 112.9)	41.8 (10.2 to 99.5)	31.7 (7 to 75.3)	32.8 (7.1 to 77.5)	31.5 (7 to 74.8)	-26.6 (-48.2 to 6.6)	-30.5 (-53.1 to 8.3)	-24.6 (-50 to 11.9)
		YLLs	38.3 (9.3 to 93.9)	41.1 (9.2 to 99)	37.3 (9 to 90.7)	26 (5.7 to 62.6)	25.5 (5.4 to 61.8)	26.3 (5.8 to 64.5)	-32.1 (-54.8 to 2.2)	-37.9 (-60 to 0.6)	-29.5 (-54.8 to 9.8)
		YLDs	5 (1.1 to 11.1)	6.1 (1.4 to 14.3)	4.5 (1 to 10.1)	5.7 (1.2 to 12.5)	7.3 (1.5 to 16.8)	5.2 (1.1 to 11.4)	15.2 (-5.3 to 42.7)	19.9 (-6.3 to 59.2)	15.9 (-8.5 to 51.2)
	Diet low in fruits	Deaths	1.8 (0.9 to 3.2)	2.4 (1.2 to 4.3)	1.5 (0.7 to 2.8)	2.4 (1 to 4.4)	2.5 (1 to 4.8)	2.4 (1 to 4.4)	33.1 (-22 to 127.9)	6.6 (-41.6 to 95.4)	59.7 (-16.2 to 215.4)
		DALYs	42.9 (22.6 to 74.2)	60.2 (31.7 to 99.9)	35.1 (17.7 to 62.4)	66.8 (27 to 118.5)	75.1 (30.7 to 135.8)	64.2 (26.2 to 117.2)	55.7 (-13.8 to 151.5)	24.8 (-32.9 to 116)	82.8 (-3.5 to 228.4)
		YLLs	38 (20.1 to 66.2)	52.7 (27.3 to 87.3)	31.3 (15.4 to 57.3)	55 (23 to 100.4)	58.7 (24.1 to 108.3)	53.9 (22.1 to 99.5)	44.7 (-21.7 to 144.8)	11.4 (-40.5 to 103.1)	72.3 (-11.3 to 223.8)
		YLDs	4.9 (2.1 to 8.7)	7.5 (3.1 to 13.2)	3.8 (1.7 to 6.7)	11.8 (3.9 to 21.9)	16.4 (5.2 to 30.8)	10.3 (3.3 to 19.2)	140.5 (56.7 to 236.2)	120 (42.6 to 215)	169.7 (70.2 to 303.5)
	Diet low in vegetables	Deaths	0.6 (0.3 to 1)	0.6 (0.4 to 1.2)	0.6 (0.3 to 1)	1.5 (0.4 to 2.9)	1.4 (0.4 to 2.7)	1.6 (0.4 to 3.1)	165.3 (-3.6 to 356.5)	134 (-17.9 to 361.5)	182.2 (-0.8 to 439.8)
		DALYs	8.9 (6.3 to 13.4)	9.4 (6.6 to 15.4)	8.6 (5.6 to 13.4)	38.3 (9.4 to 72.7)	37.4 (8.9 to 73.2)	38.8 (9.3 to 75.7)	330.3 (24.2 to 650.5)	297 (8.6 to 652.2)	348.7 (29.5 to 725.8)
		YLLs	8 (5.5 to 12.4)	8.4 (5.6 to 14)	7.9 (4.9 to 12.6)	31.5 (7.8 to 62.5)	29 (7.1 to 58.5)	32.5 (8 to 65.3)	290.9 (11.3 to 595.5)	245.2 (-5.8 to 568.4)	311.9 (17.3 to 684.6)
		YLDs	0.8 (0.6 to 1.2)	1 (0.7 to 1.4)	0.8 (0.5 to 1.1)	6.8 (1.4 to 13.2)	8.4 (1.6 to 16.9)	6.3 (1.3 to 11.9)	704.7 (88.2 to 1259.9)	727.3 (79.6 to 1365.7)	735.9 (98.2 to 1319.8)
	Diet low in whole grains	Deaths	5.9 (1.6 to 9.6)	6.3 (1.8 to 10.3)	5.7 (1.6 to 9.8)	3.6 (1 to 5.6)	3.5 (1 to 5.6)	3.6 (1.1 to 5.8)	-39.8 (-55.2 to -20.9)	-44.5 (-59.1 to -23.5)	-36.5 (-54.9 to -10)
		DALYs	122.8 (31.9 to 198)	129.8 (35.5 to 211.3)	119.1 (31.9 to 200.6)	85.7 (23.2 to 133)	89.9 (24 to 139.5)	84.6 (24.1 to 133)	-30.2 (-47.4 to -9.1)	-30.7 (-47.6 to -9)	-29 (-49.8 to -0.5)
		YLLs	102.7 (26.8 to 168.7)	105.1 (28.2 to 176.2)	101.3 (26.6 to 175.5)	66.7 (18.5 to 106.5)	65.4 (16.9 to 105)	67.5 (19.2 to 109.9)	-35 (-53.9 to -9.8)	-37.8 (-55.9 to -11.4)	-33.3 (-55.7 to -0.2)
		YLDs	20.1 (5.8 to 32.6)	24.7 (6.9 to 39.6)	17.8 (5.1 to 28.9)	19 (5 to 30.4)	24.5 (6.5 to 39.7)	17.1 (4.4 to 27.3)	-5.7 (-14.3 to 0.7)	-0.7 (-11.1 to 8.4)	-4.2 (-14.4 to 3.9)
	High body-mass index	Deaths	40.1 (24.6 to 62.4)	46 (29 to 69.4)	36.8 (20.5 to 60.7)	26.7 (16.8 to 39)	26.1 (16.9 to 37.3)	27 (16.5 to 40.6)	-33.4 (-52.5 to -5.3)	-43.4 (-59.9 to -15.4)	-26.5 (-51.2 to 13.7)
		DALYs	1163.3 (751.2 to 1722.1)	1393.7 (934.7 to 1979.9)	1058.1 (617.8 to 1677.7)	828.5 (569.3 to 1144.4)	874.6 (633.4 to 1167.2)	815.1 (546.8 to 1146.9)	-28.8 (-48.8 to -0.7)	-37.2 (-54.2 to -11.5)	-23 (-47.9 to 14.5)
		YLLs	1030.8 (651.1 to 1546.2)	1213.1 (799.4 to 1778.9)	949.5 (530.7 to 1543.3)	673.9 (446 to 966.8)	668.6 (452.6 to 917.8)	678.9 (432.2 to 1008.9)	-34.6 (-55 to -4.2)	-44.9 (-61.8 to -17.1)	-28.5 (-53.9 to 11.2)
		YLDs	132.5 (83.8 to 190.8)	180.6 (118 to 250.9)	108.6 (66.3 to 160.3)	154.6 (102.2 to 208.8)	206 (139.6 to 275.8)	136.2 (89.2 to 186.9)	16.7 (4.8 to 34.2)	14.1 (3.4 to 29.5)	25.4 (8.6 to 49.8)
	High fasting plasma glucose	Deaths	56.2 (30.9 to 99)	60 (32.2 to 115.8)	53.6 (28.6 to 90.2)	27.3 (14.6 to 46.7)	24.8 (13.7 to 45.2)	28.4 (14.9 to 49.2)	-51.4 (-65.1 to -33.6)	-58.7 (-71.6 to -37.2)	-47 (-64.3 to -20.6)
		DALYs	1073.4 (641.3 to 1704.4)	1139 (679.9 to 1924.4)	1036.6 (597.3 to 1626.8)	588.3 (353.8 to 922.6)	551.6 (336.7 to 878.1)	604.8 (350.8 to 968.7)	-45.2 (-60.5 to -26)	-51.6 (-66.7 to -27.4)	-41.7 (-60.8 to -12.6)
		YLLs	960.5 (567.7 to 1543.6)	1002.9 (585.1 to 1754.6)	938.7 (532 to 1500.3)	486.4 (287.4 to 776.2)	430.3 (259 to 705.5)	510.3 (287.7 to 834)	-49.4 (-64.8 to -29.4)	-57.1 (-71.4 to -33.3)	-45.6 (-64.2 to -15.7)
		YLDs	112.8 (62 to 183)	136 (74.8 to 221.4)	97.9 (52.6 to 165.4)	101.9 (58.4 to 161.2)	121.3 (69.8 to 192.3)	94.5 (53.1 to 150.3)	-9.7 (-26.2 to 10.3)	-10.8 (-31.9 to 18.9)	-3.5 (-26.4 to 24)
	High LDL cholesterol	Deaths	24.9 (8.8 to 51)	27.9 (9.3 to 62.6)	22.6 (8.4 to 46.9)	17.1 (6.6 to 34.1)	17.7 (6.6 to 36.1)	16.8 (6.6 to 33.9)	-31.5 (-48.3 to -4.4)	-36.6 (-53.1 to -7.2)	-25.8 (-48 to 8.5)
		DALYs	502.6 (266.5 to 875.2)	559.5 (294 to 1008)	468.5 (241 to 846.4)	403.7 (232.6 to 670.1)	442 (250.9 to 715.8)	390.9 (222.7 to 649.3)	-19.7 (-39.3 to 9.6)	-21 (-40.4 to 9.4)	-16.6 (-41.3 to 21.1)
		YLLs	419.8 (212.9 to 765.2)	453.7 (217.1 to 866.5)	397.1 (191.9 to 730.1)	314.5 (171.8 to 542.2)	322.5 (169.4 to 549.1)	312.1 (164.5 to 541)	-25.1 (-46.7 to 10.3)	-28.9 (-49.6 to 7.8)	-21.4 (-47.7 to 24.5)
		YLDs	82.8 (46.9 to 137.3)	105.8 (61.6 to 174.8)	71.4 (40.5 to 117.5)	89.2 (53 to 142.8)	119.5 (73 to 190.2)	78.8 (46.1 to 127.4)	7.8 (-0.4 to 17.2)	13 (3 to 25.6)	10.5 (0.6 to 22.4)
	High systolic blood pressure	Deaths	103.2 (77.9 to 150.6)	110.2 (80.1 to 158.9)	98.9 (69.1 to 147.2)	44.1 (30.5 to 61.3)	44.1 (30.4 to 62.9)	44.2 (29.7 to 62.6)	-57.2 (-68.2 to -43.2)	-60 (-71.6 to -42.6)	-55.3 (-68.5 to -36.7)
		DALYs	2147 (1655.1 to 3002.8)	2294.2 (1756.4 to 3194.9)	2079 (1480.7 to 2989.1)	1008.2 (732.4 to 1350.4)	1026.8 (755.5 to 1330.9)	1001.2 (719.5 to 1374.9)	-53 (-65 to -38.2)	-55.2 (-67 to -38.2)	-51.8 (-66.2 to -32.2)
		YLLs	1920.9 (1446.6 to 2793.5)	2021.1 (1494.8 to 2849.9)	1879.7 (1309.9 to 2786.9)	833.3 (581 to 1175.1)	804.5 (570.6 to 1092.9)	844.9 (579.2 to 1214)	-56.6 (-69.2 to -41.2)	-60.2 (-71.9 to -41.7)	-55.1 (-69.2 to -33.9)
		YLDs	226.1 (162.2 to 296)	273.1 (192.2 to 356.9)	199.3 (142.3 to 260.9)	174.9 (123.8 to 233.1)	222.3 (152 to 301.9)	156.3 (109 to 209.7)	-22.7 (-29.8 to -15)	-18.6 (-29.6 to -6.7)	-21.6 (-30.5 to -12.1)
	High temperature	Deaths	11 (-4.7 to 28.5)	11.8 (-5.3 to 31.4)	10.5 (-4.3 to 28.1)	6.5 (1.9 to 13.9)	6.3 (1.8 to 13.1)	6.5 (1.9 to 13.9)	-41.1 (-82.8 to -19.5)	-46.5 (-82.7 to -20.6)	-37.4 (-83.5 to -10.9)
		YLLs	191.6 (-80.2 to 497.7)	202.4 (-91.8 to 536.7)	187.1 (-76.4 to 510.8)	112.3 (33 to 237.2)	108.1 (30.8 to 223.4)	114.5 (32.1 to 239.6)	-41.4 (-82.9 to -15.8)	-46.6 (-83.7 to -19)	-38.8 (-83.8 to -6.8)
	Household air pollution from solid fuels	Deaths	0.3 (0.1 to 0.7)	0.4 (0.1 to 0.9)	0.2 (0.1 to 0.5)	0 (0 to 0)	0 (0 to 0)	0 (0 to 0)	-99.6 (-99.9 to -99)	-99.6 (-99.9 to -99.1)	-99.6 (-99.9 to -98.9)
		DALYs	6.3 (2.3 to 14.5)	8.7 (3.1 to 20.5)	5.1 (1.7 to 11.9)	0 (0 to 0.1)	0 (0 to 0.1)	0 (0 to 0.1)	-99.6 (-99.9 to -98.9)	-99.6 (-99.9 to -98.9)	-99.6 (-99.9 to -98.8)
		YLLs	5.6 (2 to 13)	7.6 (2.7 to 18)	4.6 (1.5 to 11)	0 (0 to 0.1)	0 (0 to 0.1)	0 (0 to 0.1)	-99.6 (-99.9 to -99)	-99.6 (-99.9 to -99)	-99.6 (-99.9 to -98.9)
		YLDs	0.7 (0.3 to 1.5)	1.1 (0.4 to 2.4)	0.5 (0.2 to 1.2)	0 (0 to 0)	0 (0 to 0)	0 (0 to 0)	-99.3 (-99.8 to -98.3)	-99.3 (-99.8 to -98.1)	-99.3 (-99.8 to -98.2)
	Kidney dysfunction	Deaths	16.9 (11.1 to 25.6)	19.4 (12.4 to 30.1)	15.2 (9.6 to 22.9)	11 (6.6 to 16.3)	11.1 (6.3 to 16.7)	11 (6.7 to 16.3)	-34.7 (-52.5 to -12.2)	-42.9 (-59.7 to -19.2)	-27.3 (-50.4 to 2.7)
		DALYs	349.6 (256.9 to 498.1)	409.6 (289.1 to 589.7)	314 (215.1 to 448.3)	251.4 (174.6 to 342.6)	270.5 (190.7 to 372)	245.2 (166 to 335.5)	-28.1 (-45.5 to -6.6)	-33.9 (-50.5 to -11.1)	-21.9 (-43.9 to 7.6)
		YLLs	309 (222.4 to 451.6)	355.2 (241.8 to 525.2)	281.9 (186.8 to 412.3)	204.4 (137.1 to 290)	206.9 (139 to 292.5)	204.8 (133 to 289.5)	-33.8 (-52.7 to -10)	-41.8 (-58.7 to -16.5)	-27.3 (-50 to 5.4)
		YLDs	40.6 (27.6 to 55.3)	54.4 (37.3 to 74.1)	32.1 (21.8 to 43.9)	47 (31.9 to 63.4)	63.7 (43.5 to 86.6)	40.4 (27.4 to 55.3)	15.7 (8 to 23.8)	17.1 (7.9 to 26.9)	25.9 (13.8 to 38.5)
	Lead exposure	Deaths	4.8 (1.7 to 8.9)	4.6 (1.3 to 8.9)	5 (1.9 to 9.3)	1.8 (0.5 to 3.6)	1.5 (0.3 to 3.1)	1.9 (0.5 to 3.8)	-63.6 (-77.8 to -50.3)	-68 (-82.6 to -54.9)	-62.3 (-78.3 to -45.6)
		DALYs	100.8 (34 to 185.1)	94.5 (23.2 to 180.4)	105.1 (38.4 to 197.4)	32.4 (5.9 to 69.5)	27.4 (3.4 to 62.7)	34.5 (6.7 to 73.6)	-67.9 (-84.3 to -54.1)	-71 (-87.8 to -58.4)	-67.1 (-84.8 to -51.2)
		YLLs	90.5 (30.8 to 170.8)	83.4 (20.8 to 161.5)	95.3 (34.4 to 182.8)	27.2 (5.2 to 58.7)	21.9 (2.8 to 49.8)	29.5 (5.8 to 64.2)	-69.9 (-85.2 to -56)	-73.8 (-88.6 to -61.5)	-69 (-85.6 to -52.5)
		YLDs	10.3 (3.2 to 19)	11.1 (2.6 to 21.9)	9.8 (3.4 to 17.8)	5.2 (0.8 to 11.5)	5.5 (0.5 to 13.1)	5 (0.9 to 10.8)	-50 (-77.9 to -36.4)	-50.1 (-83.1 to -35.1)	-49.1 (-77.1 to -34.3)

Location	Risk factor	Measure	1990			2019			% Change (1990 to 2019)		
			Both	Female	Male	Both	Female	Male	Both	Female	Male
	Low physical activity	Deaths	13.6 (3.5 to 26.5)	13.8 (3.4 to 27.4)	13.4 (3.3 to 26.2)	8.5 (2.1 to 16.1)	7.8 (2 to 15.5)	8.7 (2.2 to 16.9)	-37.7 (-52.6 to -18.9)	-43.3 (-57.9 to -19.3)	-35.2 (-53 to -9.4)
		DALYs	218.6 (49.6 to 443.7)	213.7 (47.5 to 441.8)	220.9 (48.4 to 443.7)	153.3 (34.2 to 310.3)	144.1 (32.2 to 299.1)	156.8 (33.7 to 316.1)	-29.9 (-46.5 to -9.7)	-32.6 (-49.3 to -9.7)	-29 (-47.7 to -3.4)
		YLLs	189 (44 to 385.2)	182.1 (42 to 382.4)	192.7 (41.2 to 396.7)	123.8 (29.4 to 253)	110.7 (24.9 to 234.2)	128.9 (27.9 to 264.5)	-34.5 (-51.9 to -11.6)	-39.2 (-55.8 to -13.9)	-33.1 (-53 to -3.9)
		YLDs	29.6 (5.6 to 64.9)	31.6 (6.3 to 73.3)	28.2 (5.1 to 60.4)	29.5 (5.6 to 64)	33.4 (6.5 to 75)	27.9 (5 to 59.8)	-0.5 (-8.4 to 9.3)	5.7 (-6 to 22.2)	-1.1 (-10.8 to 8.9)
	Low temperature	Deaths	8 (-13.2 to 28.1)	8.6 (-13.6 to 30.2)	7.6 (-12.9 to 27.7)	3.7 (-6.3 to 12.7)	3.7 (-6.2 to 12.3)	3.8 (-6.3 to 13.3)	-	-	-
		YLLs	140.2 (-227.3 to 488.8)	147.9 (-236.3 to 525.7)	137 (-236 to 484.1)	65.2 (-109.2 to 220.9)	63 (-100.5 to 210.5)	66.3 (-110.7 to 229.1)	-	-	-
	Secondhand smoke	Deaths	6.7 (4.5 to 9.8)	7.7 (5.1 to 11.4)	6.3 (3.8 to 9.5)	3.6 (2.4 to 5)	3.8 (2.6 to 5.3)	3.5 (2.3 to 5.1)	-47.1 (-61.5 to -28.2)	-51 (-65.3 to -28.9)	-43.9 (-61 to -17.1)
		DALYs	148.4 (99 to 216.3)	185.8 (124.7 to 267.9)	134.3 (84.3 to 201.1)	84.4 (57.1 to 118.7)	101.7 (70.6 to 140.9)	79.8 (52.2 to 115.1)	-43.1 (-58.5 to -22.5)	-45.3 (-61.1 to -22.4)	-40.6 (-58.9 to -12.4)
		YLLs	138.3 (91.4 to 203.9)	171 (113.8 to 250.9)	126.3 (77.2 to 193.4)	74.6 (49.6 to 106.7)	86.6 (58.3 to 122.8)	71.8 (45.6 to 104.6)	-46 (-62 to -24.4)	-49.4 (-64.7 to -25.5)	-43.1 (-62 to -13.3)
		YLDs	10.1 (6.5 to 14.2)	14.7 (9.4 to 20.4)	8 (5.2 to 11.5)	9.7 (6.5 to 13.8)	15.1 (9.9 to 21.3)	8 (5.3 to 11.4)	-3.7 (-12.9 to 7)	2.6 (-8.1 to 14.6)	-0.6 (-14.7 to 16.3)
	Smoking	Deaths	15.7 (11.2 to 22.3)	7.2 (4.7 to 11.4)	21.1 (14.5 to 30.5)	8.7 (6.2 to 11.8)	3.4 (2.3 to 5)	10.9 (7.6 to 15)	-44.4 (-61.5 to -20.7)	-53 (-71.7 to -20.8)	-48.4 (-65.4 to -23.9)
		DALYs	421.5 (305.5 to 587.3)	201.3 (134.3 to 300.7)	543.7 (382.1 to 771.3)	255.1 (187 to 339.5)	114 (78.3 to 159)	307 (220.7 to 415.9)	-39.5 (-57.9 to -14.7)	-43.4 (-64.9 to -7.1)	-43.5 (-61.4 to -18.3)
		YLLs	375.3 (261.8 to 540.9)	174.9 (113.2 to 273.3)	487.3 (326.6 to 713.7)	210.3 (144.5 to 290)	86.9 (57.7 to 126.3)	255.8 (174.3 to 358.3)	-44 (-63.2 to -17.2)	-50.3 (-70.8 to -15.2)	-47.5 (-66.3 to -19.8)
		YLDs	46.2 (32.8 to 60.7)	26.4 (16.6 to 37.4)	56.4 (39.5 to 74.8)	44.8 (31.4 to 57.7)	27.1 (17.4 to 38.9)	51.2 (35.2 to 66.4)	-3 (-17.2 to 13.4)	2.6 (-29.5 to 50.5)	-9.2 (-22.3 to 5.7)
Yemen	All risk factors	Deaths	146.5 (99.2 to 191.4)	145.3 (94.9 to 195)	148 (102.3 to 195.4)	114.7 (86.2 to 148.7)	117.6 (84.3 to 153.5)	111.4 (82.3 to 150.1)	-21.7 (-38.7 to 2.7)	-19.1 (-37.1 to 10.1)	-24.7 (-42 to 0.2)
		DALYs	3108.9 (2192.1 to 4101.1)	3127.3 (2150.8 to 4168)	3093.4 (2195.4 to 4125.7)	2391.7 (1853.8 to 3075.2)	2479.3 (1884.5 to 3225.3)	2300.6 (1725.6 to 3107.5)	-23.1 (-40.5 to 1.9)	-20.7 (-39 to 8.5)	-25.6 (-43.8 to 0.5)
		YLLs	2898.7 (1992 to 3877.6)	2888.1 (1916.9 to 3958.9)	2916.9 (2039.1 to 3958.7)	2176.4 (1644 to 2871.7)	2225.7 (1628.5 to 2951.8)	2124.8 (1549.9 to 2919.7)	-24.9 (-42.8 to 1.5)	-22.9 (-41.9 to 8.3)	-27.2 (-45.9 to 0.6)
		YLDs	210.2 (152.8 to 266)	239.1 (173.3 to 302.9)	176.5 (126.5 to 225.5)	215.2 (157.4 to 272.4)	253.6 (185.6 to 323.8)	175.7 (128.2 to 221.8)	2.4 (-3.7 to 9.1)	6.1 (-1.6 to 14.9)	-0.4 (-8 to 7.8)
	Alcohol use	Deaths	2.1 (1.2 to 3.2)	0.8 (0.3 to 1.4)	3.6 (2 to 5.5)	0.4 (0.1 to 0.7)	0 (-0.1 to 0.2)	0.8 (0.3 to 1.4)	-80.9 (-92.9 to -65.1)	-94.2 (-117.6 to -73.9)	-78.8 (-90.7 to -63.2)
		DALYs	58.8 (34.9 to 88.8)	20 (8.5 to 36.4)	98.6 (57.2 to 151.9)	12.6 (4.8 to 22.4)	1.9 (-1.1 to 5.8)	23.4 (9.8 to 41.5)	-78.6 (-90.1 to -62.2)	-90.3 (-109.8 to -72)	-76.3 (-88.1 to -59.7)
		YLLs	56 (32.4 to 85.3)	19.1 (7.9 to 35.1)	94.1 (53.6 to 146.1)	11.8 (4.6 to 21.3)	1.9 (-0.9 to 5.6)	21.9 (9.3 to 39.4)	-78.9 (-90 to -62.4)	-90 (-108.1 to -72)	-76.7 (-88.4 to -60.1)
		YLDs	2.7 (1.7 to 3.9)	0.9 (0.4 to 1.7)	4.5 (2.7 to 6.7)	0.8 (0.2 to 1.4)	0 (-0.3 to 0.4)	1.5 (0.6 to 2.5)	-72.5 (-90 to -55.3)	-96.8 (-151.7 to -67.3)	-67.3 (-84.2 to -50.6)
	Ambient particulate matter pollution	Deaths	7.2 (1.9 to 18.4)	6.1 (1.6 to 16.3)	8.7 (2.2 to 22.2)	21.7 (10.5 to 35.1)	20.2 (9.5 to 33.7)	23.3 (11 to 37.8)	201.5 (63.6 to 602.8)	232.3 (75 to 696.8)	166.3 (42.9 to 537.5)
		DALYs	166 (43 to 428)	140.1 (37.1 to 370.8)	196.1 (49.2 to 503.9)	504.6 (240.8 to 806.7)	476.2 (224.7 to 790.5)	534.1 (256.8 to 849.3)	204.1 (60.2 to 642.4)	239.9 (76.1 to 719.5)	172.4 (40.3 to 580.2)
		YLLs	154.7 (40 to 404.7)	129.1 (32.9 to 344.7)	184.5 (46.1 to 475.2)	457.7 (216.2 to 737.5)	425.1 (198.4 to 706.2)	491.3 (229.9 to 797.2)	195.9 (55.1 to 637.1)	229.3 (70 to 709.3)	166.3 (37.5 to 572.7)
		YLDs	11.3 (2.9 to 30.2)	11 (2.7 to 29.7)	11.6 (2.8 to 30)	47 (23.4 to 76.8)	51 (24.1 to 86.1)	42.9 (20.7 to 69.2)	316.3 (120.5 to 900.8)	364.3 (151.8 to 983.2)	270.9 (96.2 to 816.6)
	Diet high in red meat	Deaths	3.5 (1.1 to 6.3)	3.5 (1 to 6.3)	3.6 (1.1 to 6.4)	2.7 (0.9 to 4.8)	2.8 (0.9 to 4.9)	2.7 (0.9 to 4.7)	-23.1 (-52 to 28)	-21.1 (-52.5 to 34.5)	-25.7 (-54.6 to 19.5)
		DALYs	92.4 (25 to 164.8)	93.8 (25.4 to 167.1)	91.3 (25.5 to 164.4)	72.2 (23.1 to 125.3)	75.1 (25 to 131.3)	69.3 (23 to 121.6)	-21.9 (-52 to 34.6)	-20 (-52.9 to 44.9)	-24.1 (-54 to 29.5)
		YLLs	85.7 (22.7 to 155.3)	86.1 (22.1 to 156.2)	85.6 (23.6 to 157)	64.9 (20.9 to 114.6)	66.5 (21.1 to 118.6)	63.3 (20.4 to 113.4)	-24.2 (-53.7 to 35.2)	-22.7 (-55.3 to 47.6)	-26 (-55.1 to 30.3)
		YLDs	6.7 (2.1 to 11.7)	7.7 (2.4 to 13.3)	5.7 (1.8 to 9.8)	7.3 (2 to 12.7)	8.5 (2.3 to 14.9)	6 (1.6 to 10.6)	7.9 (-22 to 30.6)	10.8 (-22.9 to 36.8)	4.6 (-22 to 28.5)
	Diet high in sodium	Deaths	2.8 (0.4 to 11.9)	2.1 (0.4 to 8.7)	3.8 (0.3 to 16.7)	2.2 (0.4 to 8.8)	1.6 (0.3 to 6.5)	2.8 (0.3 to 11.3)	-24.1 (-55.1 to 35.7)	-23.8 (-63 to 51.1)	-27.9 (-59.4 to 56.8)
		DALYs	69.7 (8.2 to 284.5)	49.9 (8.9 to 210.2)	91.7 (6.5 to 391)	51.8 (6.9 to 205.3)	37.7 (6.9 to 157)	66.2 (5.1 to 260.9)	-25.7 (-57.2 to 39.8)	-24.4 (-65.5 to 70.8)	-27.8 (-59.1 to 53.1)
		YLLs	65.1 (7.5 to 267.6)	46.1 (7.9 to 196.6)	86.3 (6.1 to 369.3)	47.2 (6.2 to 187.4)	33.8 (6.1 to 140.3)	61 (4.6 to 242.5)	-27.5 (-58.3 to 37.9)	-26.6 (-66.4 to 67.3)	-29.4 (-60.4 to 49.9)
		YLDs	4.6 (0.6 to 18.2)	3.8 (0.7 to 15.4)	5.4 (0.4 to 22.2)	4.6 (0.6 to 18.1)	3.9 (0.7 to 16.3)	5.2 (0.4 to 20.5)	0.1 (-41.2 to 82.3)	3.2 (-50.5 to 121.4)	-2.3 (-36.5 to 102.8)
	Diet low in fiber	Deaths	7.6 (1.6 to 14.6)	7.6 (1.5 to 14.7)	7.7 (1.6 to 14.4)	6.3 (1.5 to 11.9)	6.5 (1.5 to 12.2)	6.2 (1.4 to 11.5)	-16.8 (-35.6 to 15.6)	-14.5 (-35 to 21.9)	-19.6 (-40.3 to 12.2)
		DALYs	180.4 (36.1 to 347.9)	183.2 (35.4 to 356.6)	178 (36.4 to 341.8)	152 (34.9 to 282.4)	158.1 (35.4 to 293.8)	145.8 (32.9 to 274.2)	-15.7 (-36.3 to 18.7)	-13.7 (-36.3 to 25.9)	-18.1 (-40.1 to 15.9)
		YLLs	167 (33.3 to 325.8)	167.9 (32.3 to 330.8)	166.7 (33.4 to 322.6)	136.9 (31.1 to 257.2)	140.3 (31.4 to 265.4)	133.4 (29.9 to 253.7)	-18 (-39.5 to 18.7)	-16.4 (-39.6 to 26.3)	-20 (-42.9 to 15.6)
		YLDs	13.4 (2.8 to 24.9)	15.4 (3.1 to 28.5)	11.3 (2.4 to 20.7)	15.1 (3.5 to 27.3)	17.8 (4 to 32.4)	12.4 (2.9 to 22.2)	12.7 (2.8 to 32.4)	15.8 (2.9 to 40.5)	9.8 (-2.8 to 30.1)
	Diet low in fruits	Deaths	13 (6.8 to 21.8)	13 (6.6 to 22)	13 (6.7 to 22)	8.8 (4.2 to 14.5)	9 (4.2 to 15)	8.5 (4 to 14.2)	-32.5 (-53.6 to -2.3)	-30.9 (-54 to 5.5)	-34.5 (-55.5 to -5.4)
		DALYs	325.1 (175 to 544.1)	333.4 (169.4 to 562)	317.7 (165.9 to 533)	220.3 (112.5 to 360.6)	230.3 (115.1 to 378.9)	210.2 (103.7 to 347.6)	-32.2 (-53.6 to -0.6)	-30.9 (-53.7 to 6.8)	-33.9 (-55 to -3)
		YLLs	304.3 (158.5 to 520.4)	309.4 (156.3 to 531.4)	300.3 (154 to 510.2)	200.4 (103.1 to 331.3)	206.8 (103.7 to 344.8)	193.9 (95.7 to 327)	-34.2 (-55.5 to -0.5)	-33.2 (-56.1 to 6.8)	-35.4 (-56.7 to -2.8)
		YLDs	20.8 (9.8 to 34.7)	24 (11.3 to 40.2)	17.4 (8.1 to 29.3)	20 (8.5 to 34.9)	23.6 (10.1 to 41.2)	16.3 (7 to 28.2)	-4.1 (-22.8 to 9)	-1.8 (-22.8 to 13.5)	-6.6 (-23.7 to 7.2)
	Diet low in vegetables	Deaths	8.9 (3.3 to 15.4)	8.7 (3.1 to 15.3)	9.1 (3.4 to 15.7)	6.1 (2.6 to 10)	6.1 (2.6 to 10.1)	6 (2.6 to 9.9)	-31.5 (-52.1 to 6.6)	-29.9 (-52.8 to 11.6)	-33.8 (-54.1 to 1.1)
		DALYs	217.9 (75 to 385.9)	219.3 (75.1 to 385.2)	217.5 (75.5 to 387.5)	150.9 (63.4 to 249.5)	155 (65.1 to 260.2)	146.8 (63.7 to 245.7)	-30.7 (-52 to 8.6)	-29.3 (-52.6 to 13.8)	-32.5 (-53.2 to 6.5)
		YLLs	204.6 (68.2 to 365.5)	204.3 (66.8 to 363.4)	206.2 (69.6 to 371.8)	137.8 (58.9 to 229.2)	139.7 (58.9 to 237.5)	135.9 (58.2 to 230.2)	-32.7 (-53.7 to 8.3)	-31.6 (-54.6 to 14.4)	-34.1 (-54.6 to 6.5)
		YLDs	13.3 (5.4 to 22)	15.1 (6.1 to 25)	11.3 (4.6 to 19.1)	13.1 (4.7 to 22.7)	15.3 (5.3 to 26.8)	11 (4 to 18.7)	-0.9 (-23.3 to 16.7)	1.4 (-23.3 to 22.4)	-3.3 (-23.7 to 14.1)
	Diet low in whole grains	Deaths	6.7 (3.8 to 9.8)	6.5 (3.6 to 9.5)	6.9 (3.8 to 10.7)	8.2 (4.7 to 11.9)	8.4 (4.9 to 12.1)	7.9 (4.5 to 12)	22.3 (-3.5 to 57.2)	29.7 (2.3 to 73.3)	14.5 (-14.5 to 54)
		DALYs	131.9 (72.1 to 194.8)	128.9 (67.6 to 189.8)	134.8 (73.5 to 208)	168.1 (91.8 to 240.2)	175.7 (94 to 250.8)	160.3 (87 to 240.5)	27.5 (0.4 to 63.4)	36.3 (7.5 to 81)	18.9 (-11.8 to 58.1)
		YLLs	114.1 (62.7 to 173)	108.7 (57 to 165.2)	119.7 (63.9 to 187.7)	146.7 (79.2 to 216.5)	150.3 (79.4 to 221.4)	142.9 (77 to 218.9)	28.6 (-2.4 to 72)	38.3 (5.5 to 92.4)	19.4 (-14.6 to 66)

Location	Risk factor	Measure	1990			2019			% Change (1990 to 2019)		
			Both	Female	Male	Both	Female	Male	Both	Female	Male
		YLDs	17.8 (9 to 25.9)	20.1 (10.1 to 29.8)	15 (7.6 to 22.2)	21.4 (11.2 to 31.1)	25.3 (13.3 to 36.9)	17.4 (9.1 to 25.3)	20.3 (12.5 to 29.6)	25.9 (16.1 to 38.1)	15.3 (5 to 26.5)
	High body-mass index	Deaths	15 (5.6 to 28)	18.8 (7.6 to 33.3)	11 (3 to 23)	17.8 (9.1 to 29.7)	21.2 (10.9 to 34.2)	14.4 (6.6 to 25)	18.6 (-19 to 105.8)	12.3 (-24.3 to 95.7)	31.2 (-14.7 to 183.2)
		DALYs	469.4 (178.8 to 859.5)	595.6 (250.1 to 1030.5)	343.8 (97.7 to 704.1)	564 (299.1 to 912.1)	677.3 (363.7 to 1058.7)	449.1 (215.8 to 764.7)	20.1 (-19.5 to 108.1)	13.7 (-23.5 to 99.9)	30.6 (-15.4 to 174.7)
		YLLs	436.7 (163.5 to 803.6)	550.6 (224.4 to 957.4)	323.2 (89 to 668.7)	508.8 (262.7 to 836.3)	604.1 (316.7 to 962.7)	412.1 (192.7 to 710.2)	16.5 (-23.6 to 105.1)	9.7 (-27.9 to 98.2)	27.5 (-18.9 to 175.5)
		YLDs	32.7 (13.8 to 59.7)	45 (20.2 to 76.8)	20.5 (6.1 to 41.6)	55.2 (29.7 to 84.2)	73.2 (40.9 to 110.1)	36.9 (17.8 to 59.9)	68.5 (32.7 to 156.3)	62.5 (26.8 to 144.1)	79.8 (33.1 to 234.4)
	High fasting plasma glucose	Deaths	20.7 (12.2 to 34.9)	20 (11.2 to 34.2)	22.2 (13.3 to 37.1)	32.6 (17.2 to 59.1)	34.4 (17.6 to 63.6)	30.7 (15.4 to 54.3)	57.4 (8 to 131.6)	71.9 (12.5 to 156.7)	38 (-13.5 to 106.1)
		DALYs	396.6 (247.3 to 610.7)	389.5 (231.4 to 604.4)	410.7 (251.6 to 631)	626.9 (351 to 1071.4)	659.4 (356.5 to 1142)	592.3 (307.2 to 1029.6)	58.1 (6.2 to 130.7)	69.3 (9.1 to 154.3)	44.2 (-11.6 to 120.4)
		YLLs	370.7 (228 to 573.9)	360.5 (207.4 to 568)	388.5 (236.5 to 606.4)	572.8 (314.3 to 978.1)	594.9 (320.1 to 1039.9)	549 (279.8 to 953.8)	54.5 (2.6 to 128.7)	65 (5.1 to 151.8)	41.3 (-14.9 to 117.5)
		YLDs	25.9 (15 to 43.2)	29 (16.7 to 48.3)	22.3 (12.7 to 36.6)	54.1 (27.3 to 95.1)	64.5 (31.2 to 116.5)	43.3 (21.2 to 78)	108.8 (52.3 to 175.8)	122.6 (53.7 to 206.9)	94.5 (33.6 to 163.1)
	High LDL cholesterol	Deaths	14.4 (4.7 to 32.9)	14.4 (4.5 to 34.5)	14 (4.9 to 29.2)	18.2 (7.1 to 40.1)	19.3 (7.7 to 42.1)	16.9 (6.5 to 35.7)	26.7 (-1.1 to 72.2)	34.1 (4.5 to 90.7)	21.1 (-10.4 to 68.8)
		DALYs	296.2 (149.5 to 530.2)	300.6 (150.8 to 568.4)	287.3 (141.1 to 510.6)	399.1 (221.3 to 709.5)	431.6 (244.8 to 765.9)	365.2 (198.4 to 641.5)	34.7 (4.7 to 80.1)	43.6 (10.9 to 99.3)	27.1 (-7.4 to 78.2)
		YLLs	252.8 (119.7 to 462)	249.7 (113.6 to 490.7)	252.3 (116.3 to 457.8)	344.5 (185.6 to 630.6)	365.3 (196.2 to 665)	322.7 (166.5 to 575.7)	36.3 (2.1 to 91.2)	46.3 (8.3 to 117.3)	27.9 (-10.7 to 88.1)
		YLDs	43.4 (25.3 to 71.3)	50.9 (30.1 to 84.1)	35 (20.5 to 57.5)	54.6 (33.4 to 87.1)	66.3 (40.3 to 106.3)	42.5 (25.9 to 68.1)	25.7 (16.2 to 38.2)	30.2 (18.7 to 44.8)	21.5 (8.9 to 37.3)
	High systolic blood pressure	Deaths	78.3 (49.6 to 109.4)	83 (50.8 to 118.1)	71.5 (47.1 to 102.9)	61.6 (42.7 to 85.1)	67.1 (44.9 to 93)	55.6 (37.6 to 80.5)	-21.4 (-41.3 to 8)	-19.1 (-43.2 to 18.1)	-22.3 (-44.7 to 8.7)
		DALYs	1711.7 (1138 to 2347)	1844.2 (1153 to 2602)	1558.1 (1037.8 to 2217.8)	1344.8 (963 to 1816.7)	1480.5 (1023.9 to 2029.1)	1204.2 (833.8 to 1701.9)	-21.4 (-42 to 7.3)	-19.7 (-43.5 to 16)	-22.7 (-45.3 to 7.8)
		YLLs	1598.4 (1035.7 to 2223.1)	1707.6 (1042.6 to 2427.9)	1471.3 (959.9 to 2137.4)	1223.5 (861 to 1702)	1330.3 (895.2 to 1847)	1112.6 (749.4 to 1598.3)	-23.5 (-44.3 to 6.6)	-22.1 (-46.6 to 16.5)	-24.4 (-47.6 to 7.6)
		YLDs	113.2 (76.2 to 152.7)	136.6 (90.2 to 186.5)	86.8 (58.4 to 118.6)	121.3 (84.6 to 162)	150.3 (102.8 to 202.7)	91.6 (63 to 122)	7.2 (-7.6 to 27.1)	10 (-11.1 to 38.6)	5.6 (-13.2 to 28.8)
	High temperature	Deaths	5.9 (-2.3 to 14.5)	5.9 (-2.3 to 14)	6 (-2.4 to 15.6)	4.8 (1.6 to 10.4)	4.9 (1.6 to 10)	4.7 (1.6 to 10.1)	-18.5 (-94.9 to 22.6)	-16.1 (-95 to 27.6)	-21.7 (-94.9 to 16.2)
		YLLs	115.9 (-41.9 to 287.8)	115.9 (-42.8 to 278.7)	116.5 (-41.6 to 297.8)	89.3 (29.1 to 188.2)	91.1 (30.2 to 181.1)	87.4 (29.3 to 181.1)	-23 (-95.2 to 17)	-21.4 (-95.4 to 22.4)	-25 (-95.2 to 11.1)
	Household air pollution from solid fuels	Deaths	62.8 (38.9 to 94)	64.5 (39.8 to 96.4)	60.1 (36.5 to 90.1)	18.5 (10.5 to 28.3)	20.9 (12.2 to 32.1)	15.8 (8.3 to 25.9)	-70.6 (-80.4 to -57.2)	-67.6 (-78.3 to -52.1)	-73.6 (-83.5 to -60.6)
		DALYs	1407.3 (914 to 2022.7)	1470.2 (954.5 to 2112.5)	1334.8 (835.7 to 1956.8)	429.4 (244.7 to 650)	493.4 (287.7 to 747.3)	363.5 (194.5 to 598.1)	-69.5 (-80 to -55.2)	-66.4 (-77.8 to -50.1)	-72.8 (-83 to -59.3)
		YLLs	1309.3 (826.6 to 1908.3)	1355.2 (842.5 to 1971.3)	1256.3 (764.8 to 1864.7)	388.1 (218.6 to 599.1)	440.5 (254.3 to 675.5)	334.2 (176.7 to 551.6)	-70.4 (-80.8 to -55.3)	-67.5 (-78.6 to -50.5)	-73.4 (-83.7 to -59.5)
		YLDs	97.9 (64.3 to 137.1)	115 (76.1 to 159.3)	78.6 (51.1 to 110.8)	41.2 (23 to 62.4)	52.9 (30.7 to 78.7)	29.3 (15.4 to 46.7)	-57.9 (-69.9 to -45.3)	-54 (-66.8 to -41.5)	-62.7 (-75.4 to -49.3)
	Kidney dysfunction	Deaths	11.5 (7.4 to 16.1)	12.1 (7.4 to 17.5)	10.6 (7.2 to 14.9)	12.4 (7.9 to 17.7)	13.1 (8.2 to 19)	11.5 (7.4 to 16.7)	7.6 (-17.8 to 43.9)	8.7 (-19.4 to 54.9)	8.4 (-20 to 44)
		DALYs	245.6 (166.2 to 338.4)	264.8 (171.8 to 373.9)	224.2 (157 to 309.1)	260.8 (184.9 to 351.9)	283.5 (201 to 388.5)	237.4 (166.8 to 327.7)	6.2 (-18.3 to 40.9)	7.1 (-18.7 to 48.5)	5.9 (-20.8 to 39.5)
		YLLs	227.2 (150.3 to 318.5)	242.4 (150.9 to 347.1)	210.2 (144.2 to 294.2)	235.6 (163.5 to 324.4)	252.4 (173.9 to 352.6)	218.3 (149 to 306.3)	3.7 (-21.6 to 41.1)	4.1 (-22.7 to 50)	3.9 (-23.6 to 39.6)
		YLDs	18.4 (12.6 to 24.7)	22.4 (15.3 to 30.2)	14 (9.5 to 19)	25.2 (17.1 to 34.5)	31.1 (21.1 to 42.8)	19.1 (12.9 to 26)	36.7 (25.4 to 50.2)	39 (24.4 to 58.2)	36.4 (22.2 to 52.5)
	Lead exposure	Deaths	17.7 (10.7 to 25.4)	14.6 (8.5 to 21.4)	21.8 (13.4 to 31.8)	14.5 (9.4 to 20.9)	11.9 (7.3 to 17.4)	17.2 (11.2 to 25.4)	-18 (-37.9 to 10.2)	-18.3 (-38.1 to 12.6)	-21.1 (-41.3 to 6.5)
		DALYs	416.6 (262.3 to 601.6)	342.5 (199.1 to 492.5)	500.8 (321.2 to 723.7)	301.9 (197.4 to 425.5)	248.8 (152.3 to 360.9)	357.1 (237 to 516.6)	-27.5 (-46 to -2.3)	-27.4 (-46 to 0.8)	-28.7 (-47.5 to -2.5)
		YLLs	389.7 (238.1 to 569.3)	317 (181.1 to 461.9)	472.5 (295.8 to 693.3)	276.5 (179.4 to 397.4)	224.3 (135.9 to 332.7)	330.7 (213.5 to 485.4)	-29 (-47.9 to -1.6)	-29.2 (-48.6 to 0.8)	-30 (-49.1 to -2.3)
		YLDs	26.9 (17.5 to 37.6)	25.5 (16.2 to 36.9)	28.3 (18.5 to 38.9)	25.4 (16.3 to 36.1)	24.4 (15 to 35.5)	26.4 (17.3 to 36.9)	-5.7 (-15 to 2.9)	-4.3 (-16.2 to 7.2)	-6.9 (-17 to 4.8)
	Low physical activity	Deaths	6.5 (1.4 to 14.4)	6.4 (1.5 to 14.2)	6.6 (1.2 to 14.4)	7.6 (1.7 to 16.8)	7.9 (1.8 to 17.3)	7.3 (1.4 to 16.2)	18.1 (-7.9 to 52)	23.6 (-9 to 64.3)	11.6 (-16.4 to 49.4)
		DALYs	96.6 (19.7 to 227.1)	96.7 (21.1 to 220.6)	96.4 (16.7 to 230.5)	117.4 (23.3 to 274.9)	123.8 (27.1 to 289.2)	110.6 (19.5 to 268)	21.4 (-4.2 to 54.6)	28 (-3.5 to 67.2)	14.7 (-14.3 to 52.3)
		YLLs	86.2 (17.4 to 202.3)	84.7 (18.7 to 198.2)	88 (15.7 to 208.6)	105.2 (20.7 to 243.7)	109.2 (23.5 to 254.6)	101 (17.9 to 243.3)	22.1 (-6.2 to 60.8)	28.9 (-5.5 to 74.3)	14.8 (-16 to 56.8)
		YLDs	10.4 (2 to 26.6)	12 (2.3 to 30.1)	8.4 (1.3 to 22.4)	12.2 (2.1 to 31.1)	14.6 (2.7 to 36.2)	9.6 (1.5 to 25.8)	16.3 (2.1 to 28.5)	22 (4.5 to 38.4)	14.2 (-2 to 33.2)
	Low temperature	Deaths	4.8 (-0.7 to 11.2)	4.8 (-0.7 to 11)	4.9 (-0.7 to 11.2)	3.3 (-0.3 to 6.8)	3.4 (-0.4 to 7.1)	3.2 (-0.3 to 6.8)	-32 (-68.8 to 18.8)	-29.8 (-67.6 to 23.7)	-35 (-68.6 to 17.8)
		YLLs	95 (-13.8 to 219.4)	94.7 (-13.6 to 218.1)	95.9 (-14.2 to 217)	60.9 (-6.2 to 127.1)	62.3 (-6.4 to 130.1)	59.6 (-6.2 to 126)	-35.9 (-71.3 to 14.8)	-34.2 (-71.6 to 19)	-37.9 (-70.9 to 12.3)
	Secondhand smoke	Deaths	6.7 (4.2 to 9.6)	7.5 (4.5 to 10.8)	5.8 (3.7 to 8.4)	5.2 (3.4 to 7.3)	5.7 (3.8 to 8.2)	4.6 (3 to 6.8)	-22.2 (-40.6 to 4.1)	-22.9 (-42 to 6.6)	-21 (-41.2 to 8.1)
		DALYs	160.9 (102.8 to 231.8)	190.6 (119.7 to 275.8)	131.8 (82.8 to 192.4)	123.9 (82.1 to 177.5)	144.1 (95.9 to 207.4)	103.4 (67.5 to 152)	-23 (-43.2 to 6)	-24.4 (-44.7 to 7.9)	-21.6 (-43.3 to 9.1)
		YLLs	153.9 (96.8 to 224.1)	181.3 (111.8 to 264.9)	127.2 (78.8 to 187.3)	116.5 (77.4 to 169.7)	134.4 (88.3 to 196)	98.5 (63.4 to 144.9)	-24.3 (-44.9 to 6)	-25.9 (-46.9 to 8.2)	-22.6 (-44.6 to 9.1)
		YLDs	7 (4.5 to 9.6)	9.3 (6 to 12.8)	4.6 (3 to 6.4)	7.3 (4.7 to 10.1)	9.7 (6.2 to 13.5)	4.9 (3.2 to 6.9)	5.1 (-2.7 to 13.4)	4.3 (-5.2 to 14.8)	6.9 (-5.7 to 21.2)
	Smoking	Deaths	26 (18 to 35.4)	16.4 (10.2 to 24.8)	38.2 (26.4 to 51.4)	18.7 (13.7 to 24.9)	12 (8.4 to 16.7)	25.7 (18.6 to 34.8)	-28.2 (-47.1 to -0.7)	-26.9 (-51.2 to 13.5)	-32.8 (-51.2 to -6.6)
		DALYs	717.1 (498 to 985.3)	465 (292.8 to 699.7)	993.7 (685.1 to 1358.3)	500.7 (373.3 to 659.1)	338.8 (240.6 to 464.2)	666.3 (490.7 to 912.1)	-30.2 (-49.1 to -1.4)	-27.1 (-51.9 to 12.4)	-32.9 (-51.1 to -5.8)
		YLLs	668.6 (455.7 to 931)	427.7 (261.2 to 661.3)	933.3 (630.7 to 1288.7)	454.5 (329.2 to 612.4)	301.3 (207.9 to 426.1)	611.3 (438.1 to 853.4)	-32 (-51.5 to -1.6)	-29.6 (-54.5 to 10.7)	-34.5 (-53.7 to -5.6)
		YLDs	48.5 (35 to 63.7)	37.3 (24.9 to 53)	60.4 (42.8 to 77.8)	46.2 (33 to 59.4)	37.6 (26 to 50.3)	55 (39.4 to 70.1)	-4.7 (-17.6 to 11.9)	0.7 (-24.4 to 37.4)	-9 (-18.8 to 2.9)
