# Supplementary material for: Burden of stroke in North Africa and Middle East, 1990 to 2019: a systematic analysis for the global burden of disease study 2019
Source: BMC Neurol. 2022 Jul 27;22:279. doi: 10.1186/s12883-022-02793-0 (PMC9327376; doi:10.1186/s12883-022-02793-0)
Supplement: Supplementary file 5 — Additional file 5: Supplementary Table 4. Decomposition of the predictors of expected new cases in 2019 by sex at the super-region and its 21 countries [file 12883_2022_2793_MOESM5_ESM.pdf]

Location		Sex	New cases		Expected new cases in 2019		% 1990 - 2019 new cases change cause			% 1990 - 2019 new cases overall change
			1990	2019	Population growth	Population growth + Aging	Population growth	Age structure change	Incidence rate change	
**North Africa and Middle East**		Female	191319	430334	332540	458953	73.8%	66.1%	-15%	124.9%
		Male	168353	399469	301199	415476	78.9%	67.9%	-9.5%	137.3%
Country	Afghanistan	Female	9347	20456	30193	21722	223%	-90.6%	-13.5%	118.9%
		Male	8024	16329	27897	16330	247.7%	-144.2%	0%	103.5%
	Algeria	Female	15131	32148	24996	38892	65.2%	91.8%	-44.6%	112.5%
		Male	12856	28904	21320	34699	65.8%	104.1%	-45.1%	124.8%
	Bahrain	Female	178	519	455	774	155.2%	178.9%	-142.8%	191.3%
		Male	185	717	564	1030	204.8%	251.9%	-169.1%	287.7%
	Egypt	Female	34321	79014	60217	67198	75.5%	20.3%	34.4%	130.2%
		Male	29602	73688	53335	67829	80.2%	49%	19.8%	148.9%
	Iran (Islamic Republic of)	Female	23725	52562	34389	62938	44.9%	120.3%	-43.7%	121.5%
		Male	24549	50216	35128	62645	43.1%	112.1%	-50.6%	104.6%
	Iraq	Female	12476	31249	29747	36014	138.4%	50.2%	-38.2%	150.5%
		Male	11344	29154	27255	33296	140.3%	53.3%	-36.5%	157%
	Jordan	Female	2404	7566	7249	10814	201.6%	148.3%	-135.1%	214.8%
		Male	2170	7891	6826	10368	214.6%	163.2%	-114.1%	263.7%
	Kuwait	Female	468	1630	1279	1966	173.6%	146.9%	-71.9%	248.6%
		Male	612	2375	1440	2170	135.2%	119.4%	33.4%	288%
	Lebanon	Female	2250	4840	3651	5399	62.2%	77.7%	-24.8%	115.1%
		Male	1428	2960	2199	2946	54%	52.3%	1%	107.3%
	Libya	Female	1868	5562	3009	5000	61.1%	106.6%	30.1%	197.8%
		Male	1465	3796	2301	3510	57%	82.5%	19.5%	159.1%
	Morocco	Female	16749	35310	23591	35797	40.9%	72.9%	-2.9%	110.8%
		Male	14086	28382	20200	30457	43.4%	72.8%	-14.7%	101.5%
	Oman	Female	727	1501	1463	1794	101.3%	45.5%	-40.3%	106.5%
		Male	915	2200	2385	2758	160.6%	40.8%	-61%	140.4%
	Palestine	Female	1132	2654	2679	2889	136.7%	18.5%	-20.7%	134.6%
		Male	820	2091	1986	2319	142.1%	40.6%	-27.8%	154.9%
	Qatar	Female	113	511	558	714	392.2%	137.7%	-178.4%	351.4%
		Male	208	1377	1497	1840	618.7%	164.5%	-222%	561.3%

Location	Sex	New cases		Expected new cases in 2019		% 1990 - 2019 new cases change cause			% 1990 - 2019 new cases overall change
		1990	2019	Population growth	Population growth + Aging	Population growth	Age structure change	Incidence rate change	
Saudi Arabia	Female	5205	17995	10944	14939	110.2%	76.8%	58.7%	245.7%
	Male	6366	23065	14801	19695	132.5%	76.9%	52.9%	262.3%
Sudan	Female	12723	25520	25611	25967	101.3%	2.8%	-3.5%	100.6%
	Male	11456	23075	23230	24232	102.8%	8.7%	-10.1%	101.4%
Syrian Arab Republic	Female	6673	11090	7874	14064	18%	92.8%	-44.6%	66.2%
	Male	7058	11075	7552	13782	7%	88.3%	-38.4%	56.9%
Tunisia	Female	4291	10707	5991	10726	39.6%	110.3%	-0.4%	149.5%
	Male	4032	8952	5430	9001	34.7%	88.6%	-1.2%	122%
Turkey	Female	33864	68061	46269	76703	36.6%	89.9%	-25.5%	101%
	Male	23786	57284	32257	53175	35.6%	87.9%	17.3%	140.8%
United Arab Emirates	Female	618	3827	2368	4082	282.9%	277.3%	-41.2%	519%
	Male	1169	10655	6474	11410	454.1%	422.4%	-64.7%	811.8%
Yemen	Female	6926	17173	16016	18462	131.2%	35.3%	-18.6%	148%
	Male	6108	14878	13910	16686	127.7%	45.4%	-29.6%	143.6%
