# Supplementary material for: Burden of stroke in North Africa and Middle East, 1990 to 2019: a systematic analysis for the global burden of disease study 2019
Source: BMC Neurol. 2022 Jul 27;22:279. doi: 10.1186/s12883-022-02793-0 (PMC9327376; doi:10.1186/s12883-022-02793-0)
Supplement: Supplementary file 6 — Additional file 6: Supplementary File 1. GBD 2019 cause list mapped to ICD-10 codes used in hospital/claim analyses and causes of death due to stroke and its subtypes [file 12883_2022_2793_MOESM6_ESM.pdf]

New cases

Stroke: G45-G46.8, I60-I62, I62.9-I64, I64.1, I65-I69.998, Z82.3

Ischemic stroke: G45-G46.8, I63-I63.9, I65-I66.9, I67.2-I67.848, I69.3-I69.4

Intracerebral hemorrhage: I61-I62, I62.9, I69.0-I69.298

Subarachnoid hemorrhage: I60-I60.9, I67.0-I67.1

Deaths

Stroke: G45-G46.8, I60-I63.9, I65-I66.9, I67.0-I67.3, I67.5-I67.6, I68.1-I68.2, I69.0-I69.3

Ischemic stroke: G45-G46.8, I63-I63.9, I65-I66.9, I67.2-I67.3, I67.5-I67.6, I69.3

Intracerebral hemorrhage: I61-I62, I62.1-I62.9, I68.1-I68.2, I69.1-I69.2

Subarachnoid hemorrhage: I60-I60.9, I62.0, I67.0-I67.1, I69.0
